# Cone *p*-aminocalix[4]arenes enriched with ‘clickable’ alkyne or azide functionalities

**DOI:** 10.3762/bjoc.22.28

**Published:** 2026-03-09

**Authors:** Ilia Korniltsev, Vasily Bazhenov, Alexander Gorbunov, Dmitry Cheshkov, Stanislav Bezzubov, Vladimir Kovalev, Ivan Vatsouro

**Affiliations:** 1 Department of Chemistry, M. V. Lomonosov Moscow State University, Lenin’s Hills 1, 119991 Moscow, Russia; 2 State Research Institute for Chemistry and Technology of Organoelement Compounds, Sh. Entuziastov 38, 105118 Moscow, Russia; 3 Kurnakov Institute of General and Inorganic Chemistry, Russian Academy of Sciences, Leninskii pr. 31, 119991 Moscow, Russia

**Keywords:** calixarene amines, dimeric capsules, functionalization, tetraureacalixarenes, triazoles

## Abstract

Efficient approaches have been developed for the synthesis of heteromultifunctional cone calix[4]arenes containing four amino groups at the wide rim and one, two or four propargyl or 2-azidoethyl groups at the narrow rim of the macrocycle, which can be used for expanding functionalization of the calixarene core in the well-known amine acylation (or similar reactions) and CuAAC ‘click’ reactions. Two different strategies were implemented to obtain propargylated and 2-azidoethylated *p*-aminocalixarenes. In the case of propargylated calixarenes, sterically crowding silyl protection was introduced into the alkyne groups of *p-tert*-butylcalix[4]arene (multiple) propargyl ethers, and the resulting compounds were *ipso*-nitrated followed by reduction of the nitro groups. To prepare 2-azidoethylated macrocycles, the *ipso*-nitration/reduction sequence was applied to *p-tert*-butylcalix[4]arenes containing 2-tosyloxyethyl groups at the narrow rims followed by replacement of the tosyloxy groups with azide ones. In all cases, *p*-aminocalix[4]arenes were obtained as the readily cleavable *tert*-butoxycarbonyl (Boc) derivatives, which was crucial for certain transformation and purification steps. To confirm the functionalization capabilities of the five obtained multifunctional calixarenes, they were reacted with excess benzyl azide or phenylacetylene, taken as representatives, under copper(I) catalysis, resulting in the narrow-rim triazolated macrocycles. By removing the Boc protecting groups and involving the free amino groups in reactions with *p*-tolyl isocyanate, a series of narrow-rim triazolated tetraureacalix[4]arenes was obtained. Examination of the ^1^H NMR spectra of the tetraureas in CDCl_3_ showed that in most cases triazole heterocycles do not intervene the formation of homo- and heterodimeric capsules by these compounds. Thus, considering the synthetic value of CuAAC and amine transformations, *p*-aminocalix[4]arenes enriched with alkyne or azide functionalities can be readily used as multifunctional platforms to obtain even higher functionalized macrocycles. As an example, they can be used for the preparation of sophisticated supramolecular assemblies with homo- or heterodimeric calixarene cores and virtually any functional units attached to them via triazole groups.

## Introduction

Considered generally as multifunctional molecular cores, calixarenes in their native forms as cyclic oligophenols actually possess just limited functionalization capabilities unless synthetically more valuable groups are introduced into their structures. Due to the synthetic approaches well-developed in the first decades of calixarene chemistry, these groups can be arranged in a certain number and order at the phenolic oxygen atoms and/or in the aromatic *p*-positions to them, which form respectively the narrow (lower) and wide (upper) rims of the calixarene macrocycle when it possesses a cone shape. Both the core functionalization and shape control strategies are most developed for calix[4]arene cores, and that is exactly why these cyclic tetramers may be regarded as virtually universal molecular platforms for the construction of unique multifunctional structures capable of diverse supramolecular interactions [1–4].

Amino groups attached directly to all four positions of the wide rim of the cores are among the most important functionalities which can be introduced into calix[4]arene structures either through a wide-rim exhaustive nitration followed by reduction of the nitro groups [5–13], or through azo coupling followed by cleavage of the calixarene azo compounds thus formed [14–17]. (Multi)calix[4]arenes having (dialkyl)amino groups at the wide rims are soluble in aqueous media at physiological pHs and may be used in protein sensing [18], DNA binding/recognition [19–23], and cell transfection [24–26]. Their water-soluble guanidinium derivatives are also biocompatible compounds capable of interacting with proteins, nucleic acids, and polysaccharides [27–31], and may also mimic phosphodiesterase functions [32–35]. Beyond biorelevant structures, *p*-aminocalix[4]arenes serve as preorganizing platforms that may graft together four functional/receptor units, thus drastically improving their supramolecular interaction capabilities. This property of *p*-aminocalix[4]arenes has been widely utilized in constructing *f*-element-targeted extractants for nuclear waste treatment, having four carbamoylmethylphosphine oxide groups introduced to the macrocyclic core through simple acylation of *p*-aminocalix[4]arenes with the respective phosphorous-containing activated esters. Such grafting of well-known receptor units onto a common platform has led to substantial amplification of the efficiency of lanthanide and actinide extraction from acidic media by carbamoylmethylphosphine oxides along with enrichment of the extraction selectivity [36–45]. Even more impressive is the effect from grafting of four urea groups at the wide rims of calix[4]arene macrocycles achieved by reacting *p*-aminocalix[4]arenes with aryl- or arylsulfonyl isocyanates. In aprotic media, these compounds, referred to as ‘tetraureacalix[4]arenes’, assemble into well-defined homo- or heterodimeric capsules, which are held together by cyclic belts of hydrogen bonds between interpenetrated urea groups from two molecules and are able to include a single neutral molecule or a trialkylammonium cation inside the joint cavity formed by two cone calix[4]arene macrocycles [46–54]. This phenomenon has been thoroughly investigated, including the effects from substituents in the urea groups and/or at the calixarene narrow rim upon the thermodynamic and kinetic stability of the capsules and the guest exchange processes [55–60]. This has made dimerization of tetraureacalix[4]arenes one of the most explored self-organization processes in calixarene supramolecular chemistry, which has been used in the template synthesis of calixarene-based multi(macrocycles) and multi(catenanes) having impressive topology [61–69].

Over the past two decades, azide and alkyne groups have also appeared among the most synthetically valuable functionalities introduced into calixarene cores due to the ease of their conversion into diverse 1,4-disubstituted 1,2,3-triazole units under CuAAC (copper(I)-catalyzed azide–alkyne cycloaddition) ‘click’ conditions [70–73]. The first implementation of the CuAAC approach for calixarene modification was published shortly after its introduction into chemistry in general, and used cone calix[4]arenes having four propargyl or 2-azidoethyl groups at their narrow rims as CuAAC substrates [74]. Since then, hundreds of publications have reported the application of the CuAAC reaction for the introduction of diverse biorelevant, receptor or sensory functionalities into the structures of calixarenes and related macrocycles [75–78]. Many of them relied on the introduction of propargyl or azidoalkyl groups to the phenolic oxygen atoms for synthesizing the CuAAC-ready calixarene cores. Within this area of research, our studies on the CuAAC reactions applied to cone propargylated and 2-azidoethylated calix[4]arenes revealed their enhanced efficiency, resulting in the preferential formation of exhaustively triazolated macrocycles over mixed triazolated/propargylated or triazolated/2-azidoethylated ones [79–80]. Furthermore, it has been proved that triazolated calixarenes derived from propargylated and 2-azidoethylated precursors possess drastically different complexation abilities towards transition-metal cations [81–84] including the formation of unique inherently dinuclear iridium(III) complexes with cone calix[4]arenes having pairs of 2-(4-aryltriazol-1-yl)ethyl groups at their narrow rims [85]. Regarding the synthetic capabilities of propargyl and 2-azidoethyl groups residing the calixarene core, we have established a series of triazolated calix[4]semitubes in which two or three calix[4]arene macrocycles are connected to each other by pairs of triazole units formed via two-fold CuAAC macrocyclizations [86–87]. We have also developed a straightforward approach to the functionalization of water-soluble *p*-sulfonatocalix[4]arenes by their enrichment with propargyl groups at the narrow rims followed by CuAAC reactions with various azides [88].

**Figure 1:**
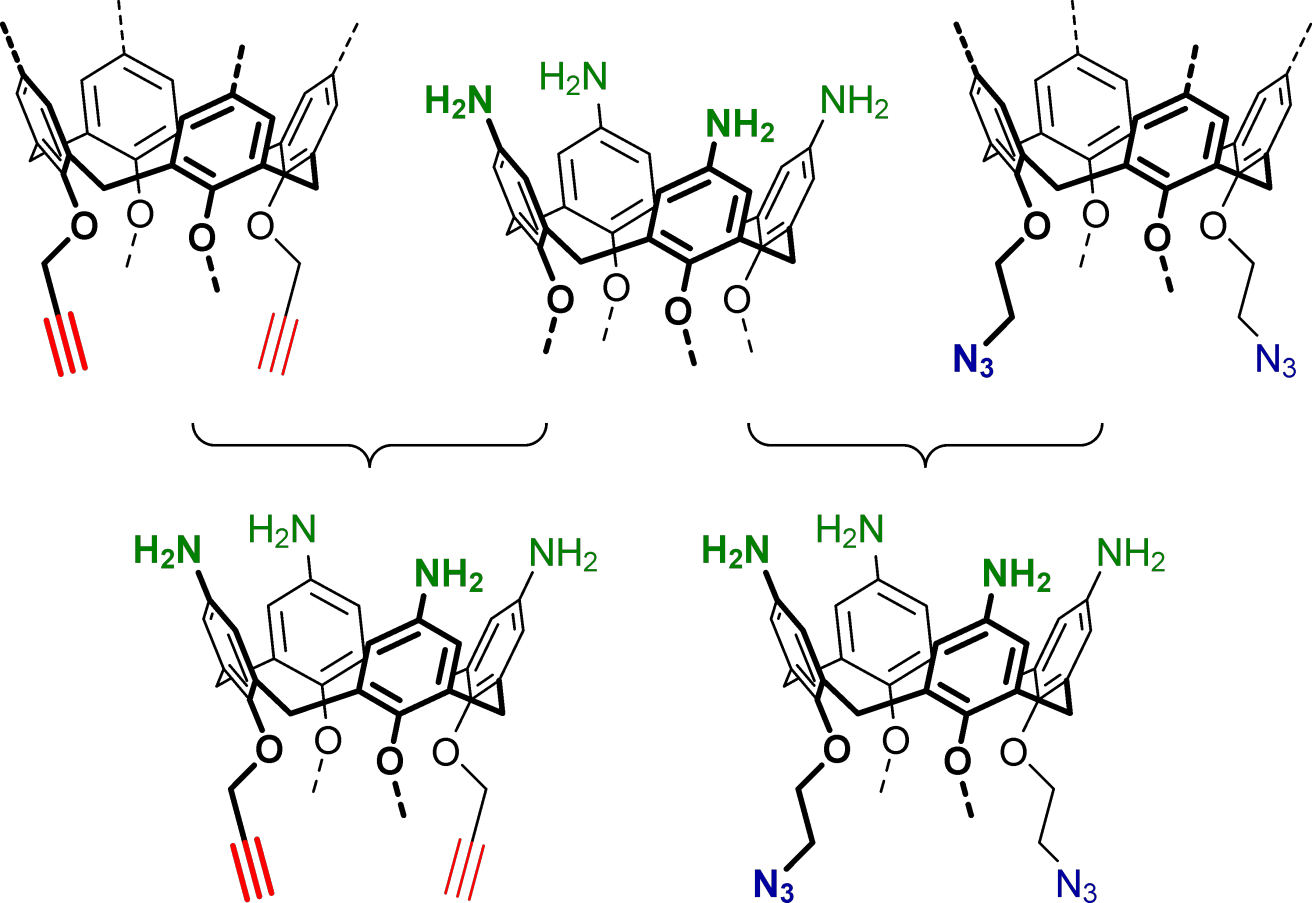
General concept of building multifunctional calix[4]arenes by joining propargyl/2-azidoethyl and *p*-amino groups on a common platform.

Inspired by the above benefits brought to the calixarene structures by amino groups, the power of CuAAC reactions in building multifunctional architectures, and following the pioneering work on bifunctional “Janus” calix[4]arenes joining propargyl groups and nitro/amino/azide groups in the structures [9], we herein report on the synthesis approaches to a series of cone *p*-aminocalix[4]arenes having propargyl or 2-azidoethyl groups at the narrow rims (Figure 1). The newly synthesized compounds may be thus treated as novel building blocks in calixarene chemistry capable of step-wise orthogonal transformations into various multifunctional structures and sophisticated supramolecular systems.

## Results and Discussion

In the majority of cases, the introduction of four amino groups into the calix[4]arene wide rim requires exhaustive (*ipso*-)nitration of the core followed by reduction of the resulting *p*-nitrocalix[4]arene. Notably, the alternative strategy of utilizing an azo coupling/cleavage sequence has only been applied thus far for the wide-rim modification of narrow-rim unsubstituted calixarenes. Since the exhaustive nitration and reduction steps proceed under relatively drastic conditions, which are poorly compatible with propargyl or 2-azidoethyl functionalities residing at the calixarene narrow rims, these ‘simple’ transformations are not applicable to the available propargylated/2-azidoethylated *p-tert*-butyl- or *p*-H-calix[4]arenes. Therefore, the multistep reaction sequences presented below have been developed for the preparation of the targeted multifunctionalized calixarenes.

### Propargylated *p*-aminocalix[4]arenes

Following the published example [9], for the preparation of propargylated *p*-aminocalix[4]arenes a three-step synthesis strategy was selected, which involved protection of the calixarene propargyl ethers by *tert*-butyldimethyl-silylation followed by the calixarene wide-rim exhaustive nitration and reduction of the nitro groups. The readily available cone calix[4]arenes **1**–**5** [89–92] bearing up to four propargyl groups at their narrow rims and *n*-propyl groups completing the substitution patterns were selected as the starting materials to enable further preparation of the respective mono-, di- and tetrapropargylated calix[4]arene tetraamines and their derivatives.

**Scheme 1:**
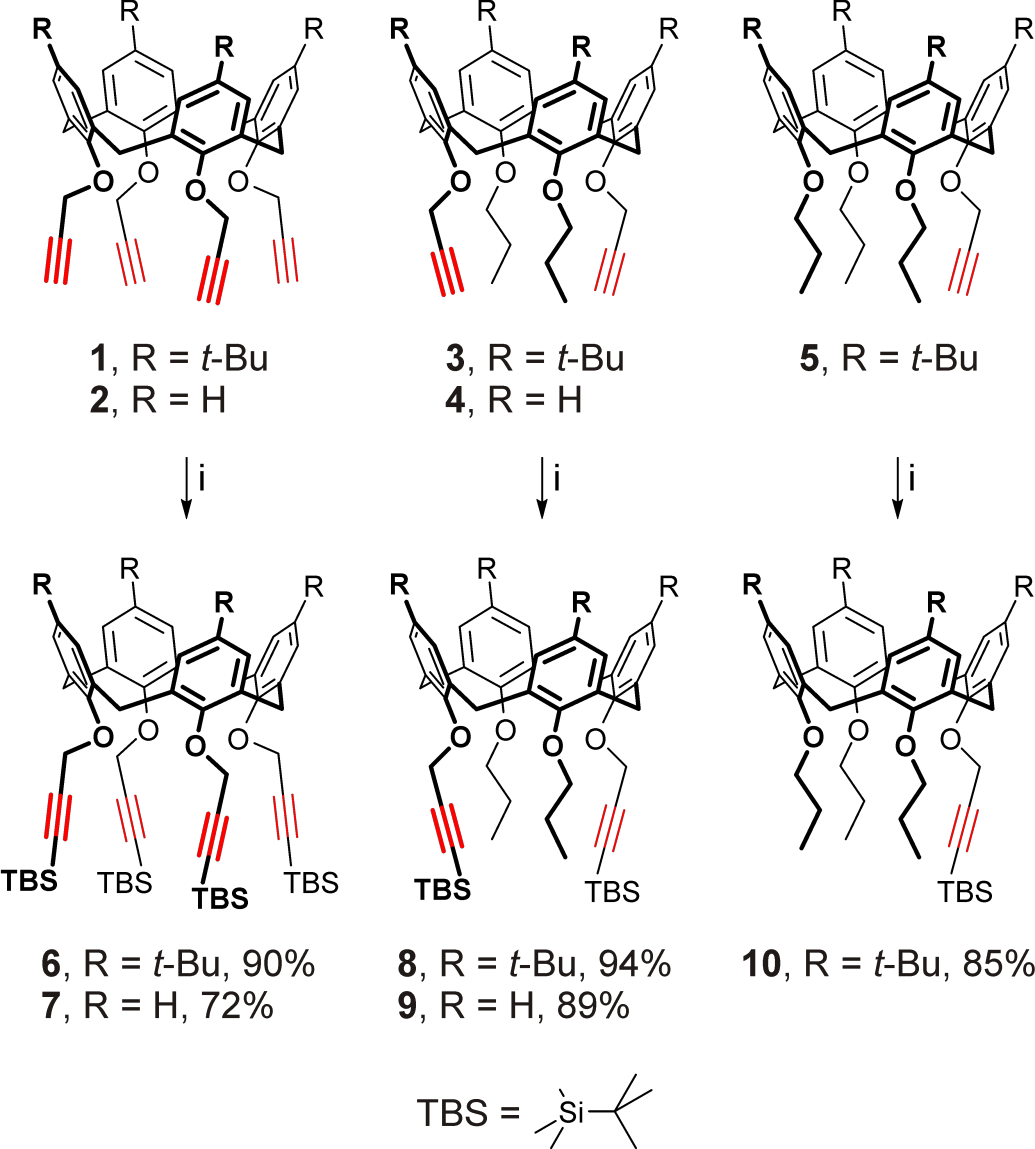
Silylation of the calixarene propargyl ethers. Conditions: i) LiHMDS (HMDS + *n-*BuLi), TBSCl, THF, −95 °C → rt.

The silylation of propargyl ethers **1**–**5** was first attempted using *tert*-butyldimethylsilyl chloride (TBSCl) and the commercially available lithium bis(trimethylsilyl)amide (LiHMDS), but the latter was found inconvenient for routine syntheses due to high moisture sensitivity which prevented reproducibility of the reaction conditions. To overcome this difficulty, the LiHMDS solution was instead prepared in situ by reacting bis(trimethylsilyl)amine (HMDS) with *n*-butyllithium in THF immediately before the addition of a propargylated calixarene followed by TBSCl. This allowed us to improve the yield of the known calix[4]arene **6** [9] having four silylated propargyl groups at the narrow rim, and to obtain the respective persilylated propargyl ethers **7**–**10** from precursors **2**–**5** (Scheme 1).

**Scheme 2:**
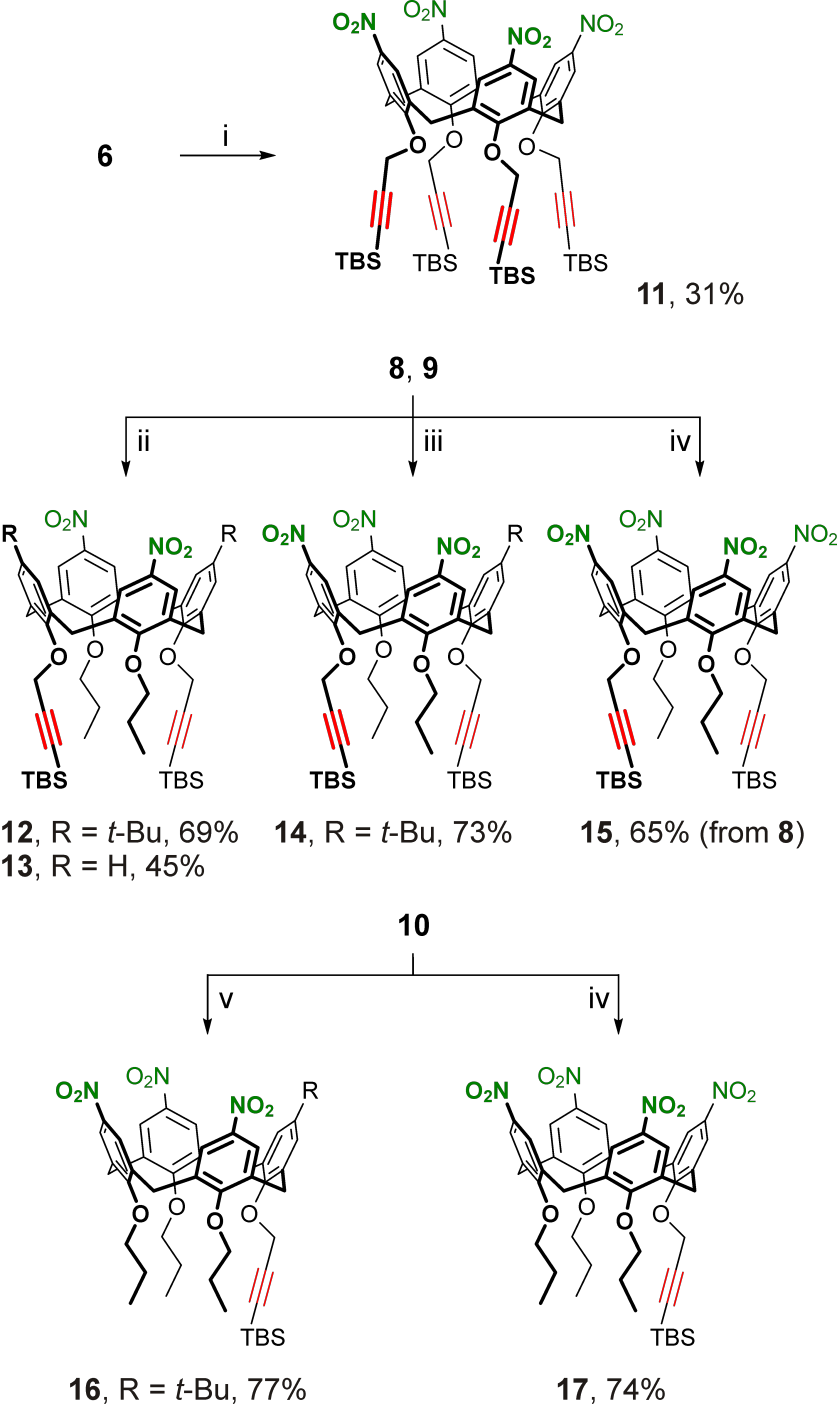
Nitration of calixarenes **6**, **8**–**10**. Conditions: i) HNO_3_ (fuming, 10 equiv per calixarene aromatic unit, *c* ≈ 1 M), AcOH, dichloromethane, rt, 72 h; ii) HNO_3_ (fuming, 2.5 equiv per calixarene aromatic unit, *c* ≈ 0.2 M), AcOH, dichloromethane, rt, 12 h; iii) HNO_3_ (fuming, 2.5 equiv per calixarene aromatic unit, *c* ≈ 1 M), AcOH, dichloromethane, rt, 12 h; iv) HNO_3_ (fuming, 10 equiv per calixarene aromatic unit, *c* ≈ 2.5 M), AcOH, dichloromethane, rt, 48 h; v) HNO_3_ (fuming, 2.5 equiv per calixarene aromatic unit, *c* ≈ 1 M), AcOH, dichloromethane, rt, 48 h.

Expectedly, (*ipso*-)nitration of calixarenes **6**–**10** by mixtures of fuming nitric acid and glacial acetic acid in dichloromethane solutions could potentially be complicated by both the incomplete conversion of the starting materials and also by formation of side products, due to acid-promoted cleavage and undesired transformations of the TBS-protected propargyl groups and/or due to unwanted oxidation/nitration of the calixarene core [93]. Indeed, nitration of calixarene **6** has been reported to furnish the desired wide-rim exhaustively nitrated calix[4]arene **11** having four TBS-protected propargyl groups at the narrow rim in only moderate yield of 37% [9]. In our hands, according to the NMR spectra of the reaction mixture, under the reported conditions (>30 equiv of fuming HNO_3_ per calixarene aromatic unit in a 1:1 dichloromethane/acetic acid mixture, from 0 °C → rt, overnight) the nitration of calixarene **6** resulted in a mixture of exhaustively nitrated product **11**, partially nitrated calixarenes having nitro and *tert*-butyl groups at the wide rims, and a large amount of other calixarene side product(s) having broadened and non-interpretable NMR spectra. Upon tuning the reaction conditions, it was found that a more complete nitration of calixarene **6** along with some suppression of the undesired side reactions could be achieved by decreasing the nitric acid excess (to ≈10 equiv per calixarene aromatic unit) and its concentration in dichloromethane (to ≈1 M), reducing the content of acetic acid in the mixture (to 1 mol per mol of HNO_3_) and extending the room-temperature reaction time to 72 h. Yet, under these conditions, the desired calix[4]arene **11** was obtained in only 31% yield (Scheme 2), and further variations in reagent excess/concentration and reaction time did not significantly improve the yield. Also an attempt to obtain calixarene **11** by nitration of the silylated *p*-H-calix[4]arene tetrapropargyl ether **7** failed, as no complete conversion of the starting calixarene could be achieved likely due to a slower nitration of unsubstituted calixarene aromatic units in comparison with the *ipso*-nitration of the corresponding *tert*-butylated ones. Even larger amounts of side products were formed, thus confirming the involvement of the tightly arranged narrow-rim substituents in calixarene **6** and **7** in undesired side processes under nitration conditions.

In line with this, compounds **8**–**10** having less sterically hindered narrow-rim substitution patterns were found to be more resistant to side reactions during wide-rim nitration as well as to the target exhaustive nitration itself. These reactions required a higher nitric acid concentration of ≈2.5 M to complete the process and to obtain the *p*-nitrocalix[4]arenes **15** and **17** in good yield (Scheme 2). Upon further fine-tuning the reaction conditions it was found that some of the partially nitrated products could also be successfully prepared by using smaller amounts of the nitrating agent. Thus, the room-temperature nitration of the silylated *p-tert*-butylcalix[4]arene ether **8** using 2.5 equiv of HNO_3_ per calixarene aromatic unit resulted in di- or trinitrated macrocycles **12** and **14** as the major products, when ≈0.2 and ≈1 M nitric acid concentrations were used. Similarly, the wide-rim unsubstituted calixarene **9** was successfully converted into the dinitro-derivative **13** using the dichloromethane-diluted nitric acid/acetic acid mixture. Also, it was found that calixarene **10** could be converted to the trinitrated product **16** under the same conditions as for the synthesis of calixarene **14**, with only the reaction time increased (Scheme 2).

**Figure 2:**
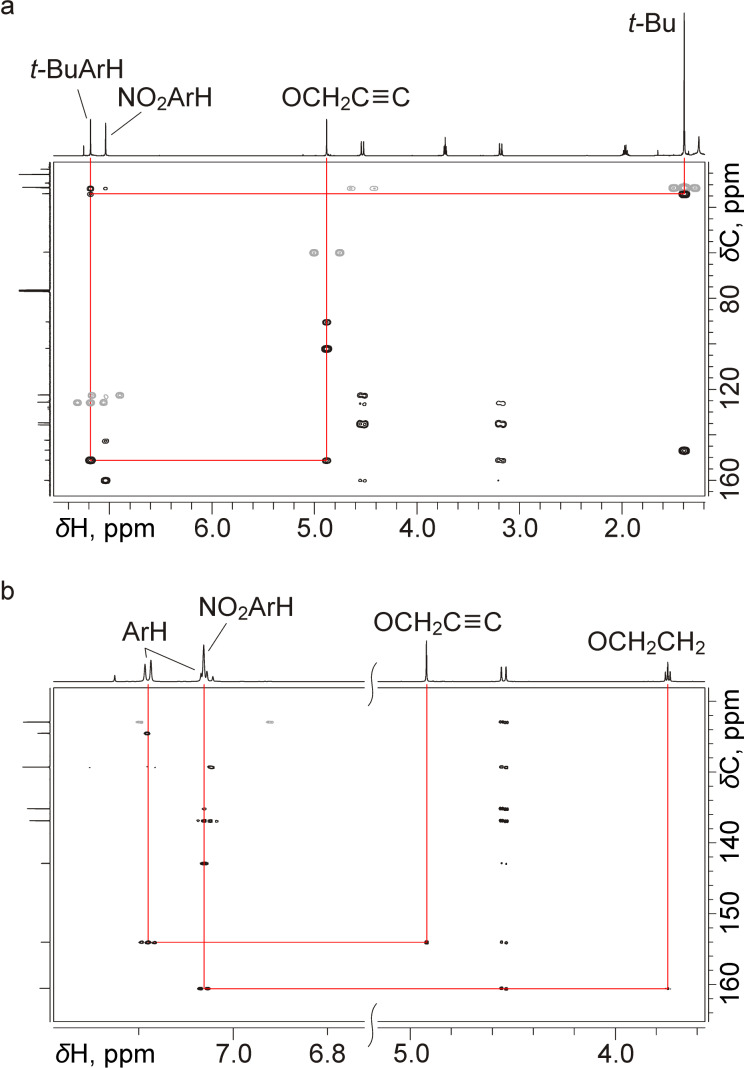
Parts of ^1^H,^13^C HMBC spectra of calixarenes **12** (a) and **13** (b) recorded in CDCl_3_ solutions at 600 MHz. Red lines show the key correlations between signals from the calixarene narrow-rim substituents and aromatic units.

The 1D NMR spectra for the dinitrocalix[4]arenes **12** and **13** provided no definitive data on the substitution patterns at their wide-rims except for the presence of two nitro groups in the distal aromatic units. To localize the nitro groups in the structures of calixarenes **12** and **13**, ^1^H,^13^C HMBC spectra were acquired (Figure 2), which clearly showed that the nitro groups are attached to the propylated aromatic units of the macrocycle in both cases. Notably, the correlations showed that the signals from the nitrated calixarene aromatic units appeared upfield-shifted in the ^1^H NMR spectra with respect to those from the aromatic units bearing no electron-withdrawing groups. This uncommon signal position may indicate a specific time-averaged conformation of the calixarene core in compounds **12** and **13** due to the presence of the bulky TBS-protected propargyl groups in their structures, in which the nitrated aromatic units experience an additional shielding. This may be also responsible for the selectivity of the two-fold nitration of compounds **8** and **9** leading to calixarenes **12** and **13** rather than to their isomers containing nitro groups in the propargylated aromatic units of the macrocycles.

There was no doubt in establishing the structure of the trinitrated calix[4]arene **14**, as its NMR spectra contained a single set of resonances from propyl groups and two sets of those from the TBS-protected propargyl groups, which indicated clearly the symmetry plane passing through the propargylated aromatic units of the calixarene. Assuming a step-wise nitration of calixarene **8**, the dinitrated calixarene **12** must be the immediate precursor of compound **14**. The presence of the nitro groups in both of the propylated aromatic units of this calixarene can only be derived from calixarene **12**, which is additional evidence for its structure.

**Figure 3:**
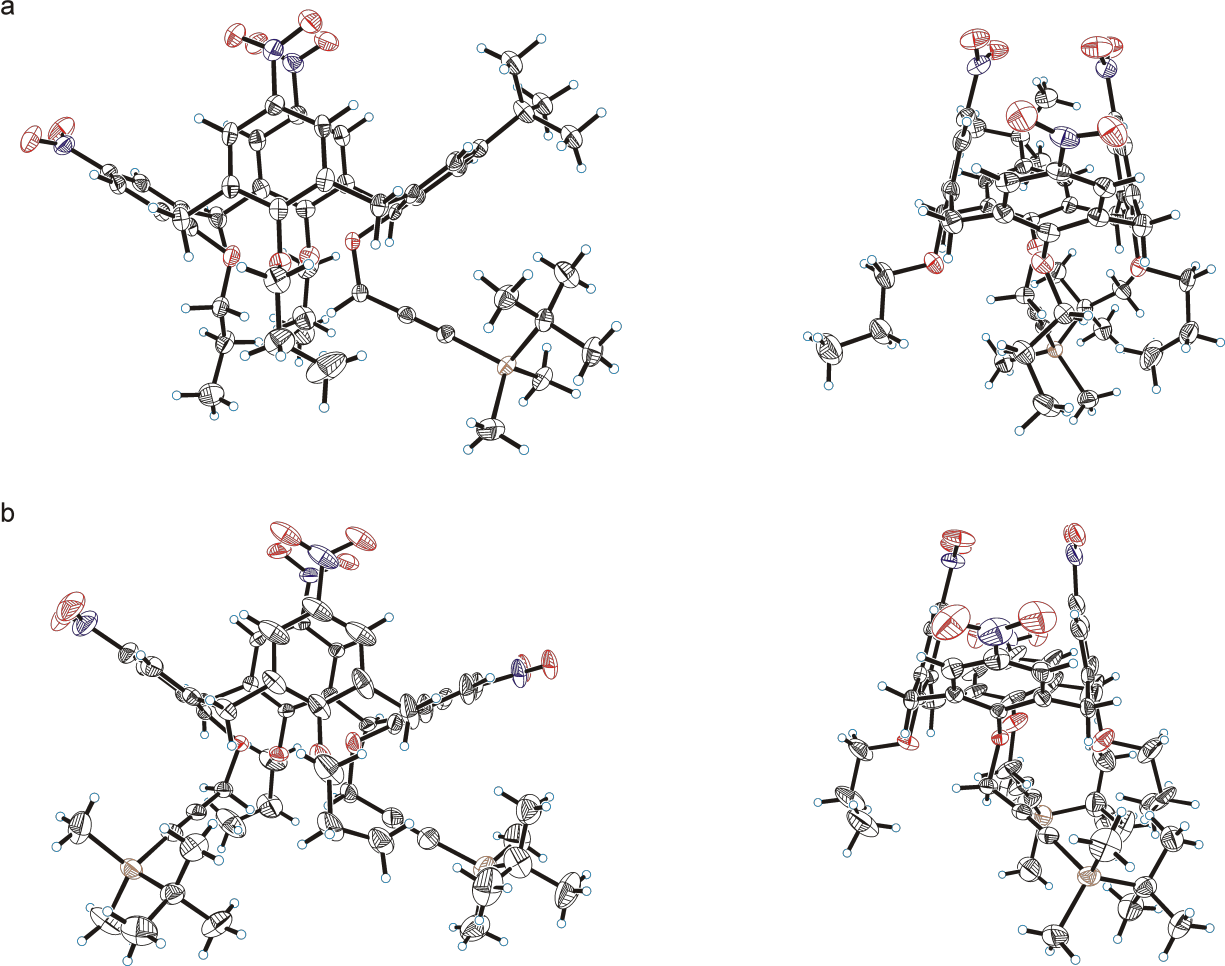
Molecular structures of partially nitrated calixarene **16** (a) and exhaustively nitrated calixarene **15** (b). Two projections shown in each case and thermal ellipsoids are drawn at a 50% probability level.

The structure of trinitrated calix[4]arene **16** having a single TBS-protected propargyl group at the narrow rim was unambiguously established from X-ray diffraction data. Suitable crystals were collected upon slow evaporation of a dichloromethane/methanol solution of compound **16**. Similarly, single crystals of the exhaustively nitrated calix[4]arene **15** having two TBS-protected propargyl groups at the narrow rim were collected, and the molecular structure of this compound was also established (Figure 3) [94]. The results showed clearly, that in calixarene **16** all three propylated aromatic units were nitrated, and the propargylated one still contained a *tert*-butyl group. Thus, within a step-wise nitration paradigm, the narrow-rim propargylated aromatic units are more stable against nitration when compared to the propylated aromatic units of the calixarene core. This correlates well with the above selectivity of nitration observed for calixarenes **8** and **9** and may be tentatively explained by an electron-withdrawing effect of the propargyl groups.

However, in the molecular structures of calixarenes **15** and **16**, another feature was observed: in both cases the calixarene aromatic units containing the TBS-protected propargyl groups (and the opposed one in the case of compound **16**) are extremely flattened, while the other aromatic units are pinched and bring the two nitro groups into very close contact. Similar effects from the TBS-protected propargyl groups may be also expected in calixarenes **6**–**10** and their partially nitrated products. Thus complex conformational transitions in the calixarene cores may also interfere with the reaction, and, in particular, may hamper the exhaustive nitration of calixarenes **6** and **7** each bearing four TBS-protected propargyl groups at their narrow rims.

The formation of the partially nitrated calix[4]arenes **12**–**14** and **16** as major reaction products described above was quite unexpected, and these compounds were not used further in this study which was more concerned with calix[4]arenes having four equal substituents at the wide rims. But surely, the partially nitrated calix[4]arenes bearing protected propargyl groups in their structures may be further explored as multifunctional cores capable of other potential applications in calixarene chemistry.

**Scheme 3:**
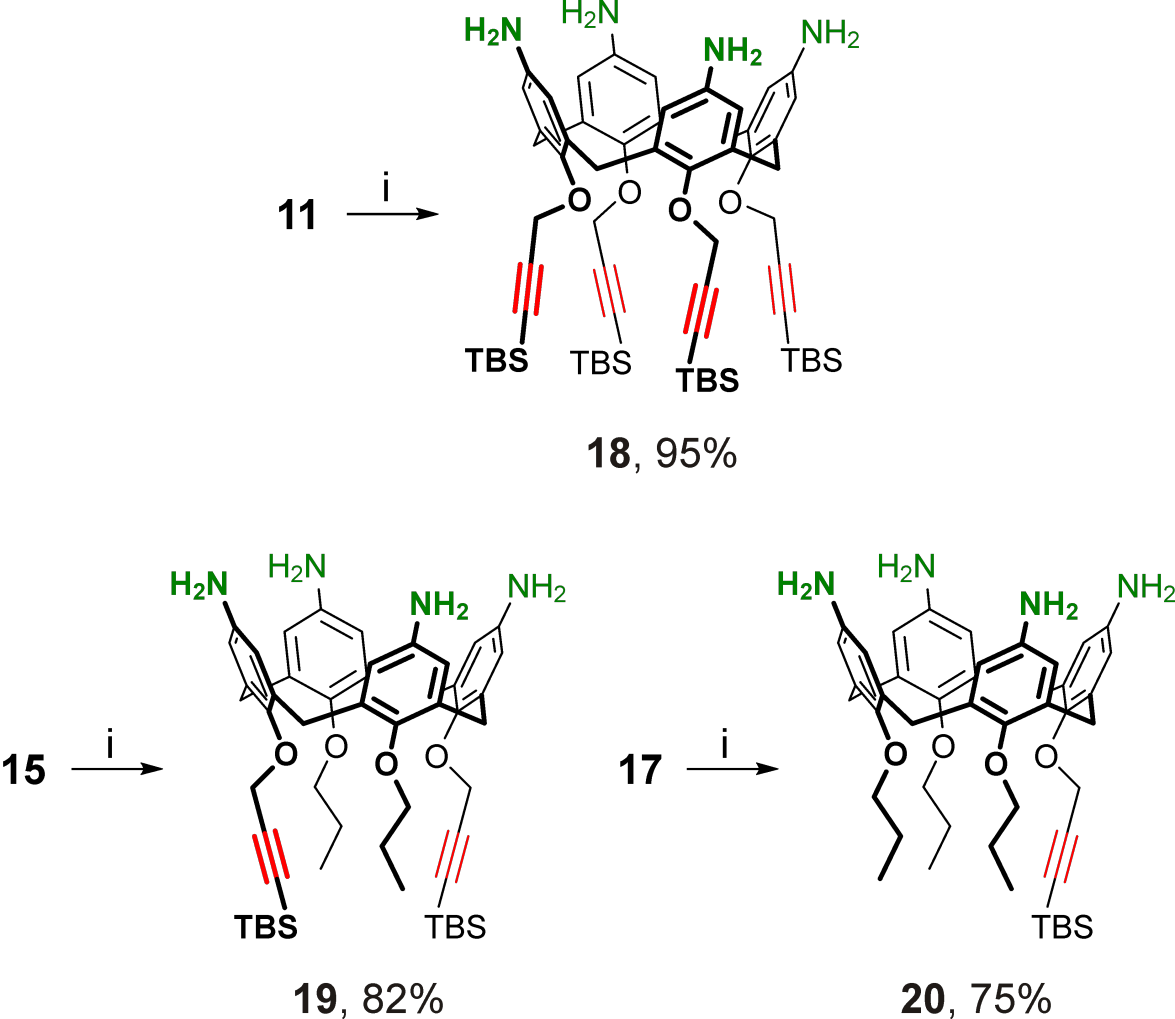
Reduction of calixarenes **11**, **15** and **17**. Conditions: i) SnCl_2_·2H_2_O, HCl, EtOH, H_2_O, 70 °C, then KOH, H_2_O.

TBS units attached to the propargyl groups of calixarenes appeared to be not bulky enough to protect the alkyne from a Raney-Ni-catalyzed reduction by gaseous hydrogen, as a trial hydrogenation of the nitrated/propargylated calix[4]arene **11** resulted in only trace amounts of the desired *p*-aminocalix[4]arene **18** among numerous other reaction products. Due to this, a homogeneous reduction using tin(II) chloride in ethanol was used after fine-tuning of the published reaction conditions [9]: the reduction was conducted by gentle heating of a mixture of calixarene **11**, SnCl_2_·2H_2_O, aqueous HCl and ethanol at 70 °C (instead of prolonged boiling of the reaction mixture) in a closed vessel to prevent the product oxidation. At the work-up step sodium hydroxide was replaced with potassium hydroxide which provided better water solubility of inorganic salts for their extractive removal. As a result, tetraamine **18** having four TBS-protected propargyl groups at the narrow rim was obtained in substantially improved yield and the nitrated calixarenes **15** and **17** were similarly reduced to the corresponding tetraamines **19** and **20** (Scheme 3).

**Scheme 4:**
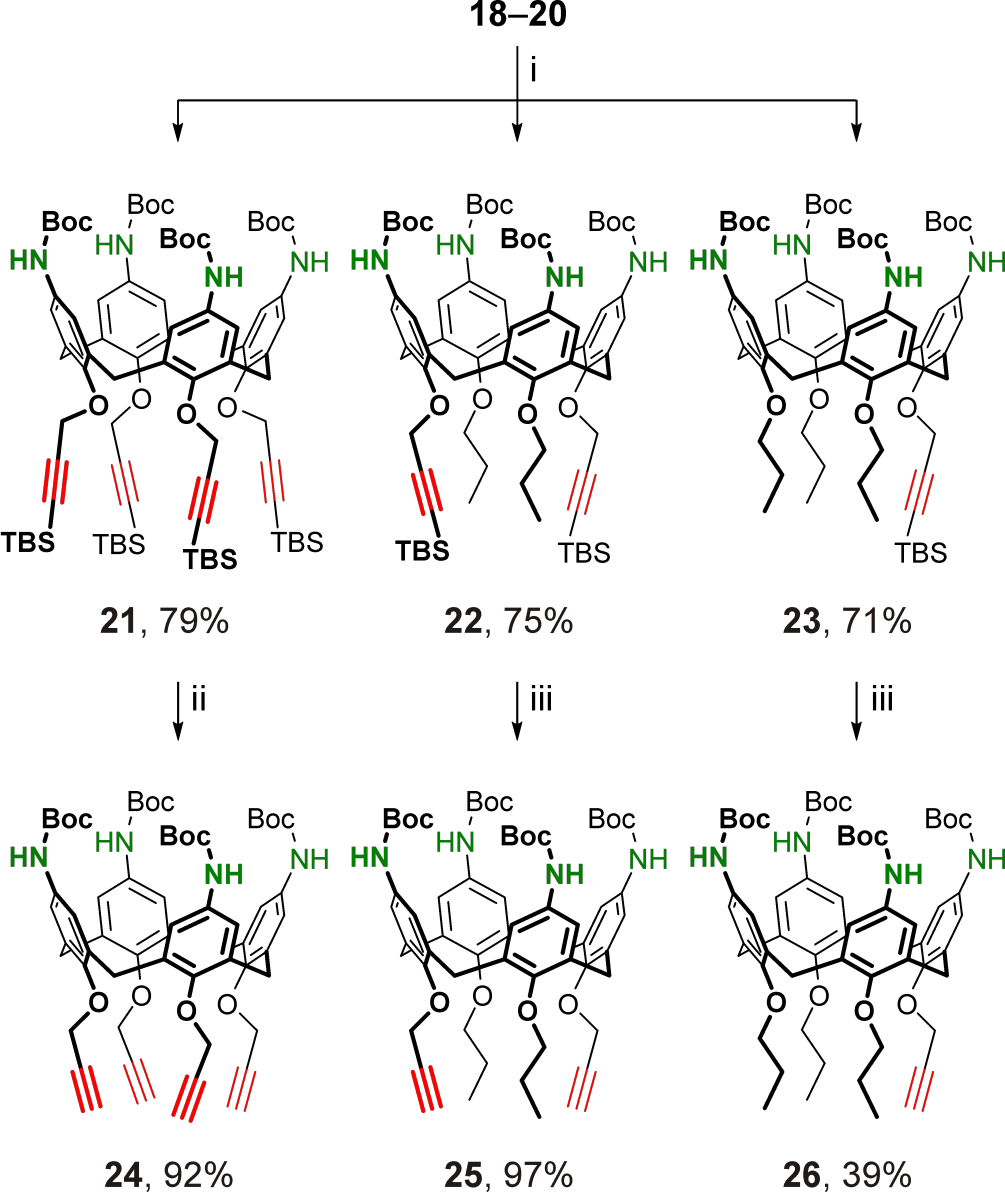
Protecting group replacement in propargylated *p*-aminocalix[4]arenes. Conditions: i) Boc_2_O, Et_3_N, dichloromethane, rt; ii) H_2_O, *n-*Bu_4_NF·3H_2_O, THF, rt; iii) H_2_O, *n-*Bu_4_NF·3H_2_O, THF, 50 °C.

Chemical modifications of propargylated *p*-aminocalix[4]arenes may be performed by the amine acylation followed by the CuAAC involving the propargyl groups, or vice versa. Of these two possibilities, the triazole synthesis conducted prior to the target acylation of amines seems to be more convenient, as the acylation may proceed under milder conditions than the CuAAC reaction. Even more important, the amine-derived functional units may interfere or affect the cycloaddition by, for instance, binding copper cations or counter anions, and thus their presence in the structures of calixarenes at the CuAAC step must be avoided. On the other hand, the unmodified amines may also contribute to undesired Cu(I) stabilization and complicate the CuAAC work up in acidic conditions, so the amino groups in calixarenes **18**–**20** must be deactivated prior to the cycloaddition. Following the above reasons, a two-step replacement of protecting groups in calixarenes **18**–**20** was performed. First, the amino groups were acylated with di-*tert*-butyl dicarbonate (Boc_2_O) and next the TBS-protecting groups were removed from the acetylene units in the Boc-protected tetraamines **21**–**23** (Scheme 4).

For the removal of the TBS groups, instead of using an equivalent amount of *n-*Bu_4_NF, it was used as a catalyst in a water/THF mixture, which showed excellent efficiency for the room-temperature preparation of the tetrapropargyl ether **24** from its silylated precursor **21**. Surprisingly, attempts to apply these conditions to a complete removal of TBS groups in the less sterically hindered propargyl ethers **22** and **23** failed, and large amounts of the starting materials (as well as the partially desilylated product in the case of calixarene **22**) remained in the reaction mixtures even after their stirring at room temperature for 72 h. In the case of calixarene **22**, heating of the mixture at 50 °C for 48 h was enough to complete the process and *p*-aminocalix[4]arene **25** having two propargyl groups at the narrow rim was obtained in 97% yield. Still, the deprotection of calixarene **23** to the desired propargyl ether **26** was far from complete even under heating, which significantly reduced the yield of this compound (Scheme 4).

The difference in the efficiency of catalytic removal of the TBS groups observed for calixarenes **21**–**23** may be due to a reaction rate enhancement upon increasing the number of TBS-protected propargyl groups attached to the same calixarene core. Tentatively, this rate enhancement may be due to entrapment of the catalytic F^–^ and/or water molecule(s) inside a pocket formed by the four TBS-protected propargyl groups in calixarene **21**, which thus may be responsible for an autocatalytic process. Of course, this phenomenon needs to be studied in further detail.

### 2-Azidoethylated *p*-aminocalix[4]arenes

**Scheme 5:**
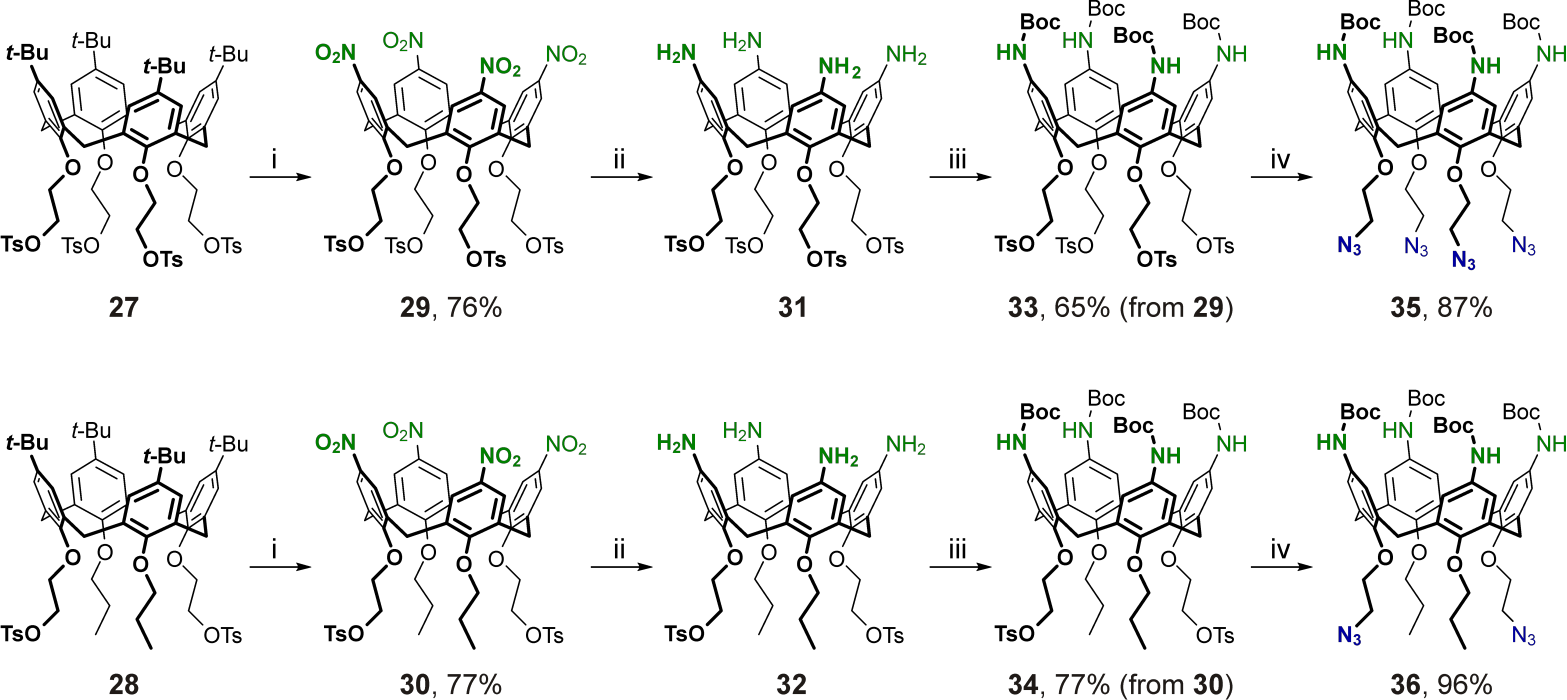
Synthesis of 2-azidoethylated *p*-aminocalix[4]arenes. Conditions: i) HNO_3_ (fuming, 10 equiv per calixarene aromatic unit, *c* ≈ 2.5 M), AcOH, dichloromethane, rt, 48 h; ii) FeSO_4_·7H_2_O, NH_4_Cl, Zn, EtOH, THF, H_2_O, 50 °C; iii) Boc_2_O, Et_3_N, dichloromethane, rt; iv) NaN_3_, DMF, 60 °C.

Due to the instability of azide groups in the presence of reducing agents, a synthesis strategy similar to that described above for the preparation of calixarenes **24**–**26** involving nitration of calix[4]arenes followed by selective reduction of the nitro groups could not be implemented for the preparation of *p*-aminocalix[4]arenes bearing 2-azidoethyl groups at their narrow rims. To overcome this, calixarenes **27** [95–96] and **28** [97] decorated with four or two 2-tosyloxyethyl groups which are precursors to the desired 2-azidoethyl groups, were subjected to a nitration/reduction sequence as shown in Scheme 5.

The *ipso*-nitration step proceeded smoothly in both cases under the optimized conditions, and the wide-rim pernitrated tetra- and ditosylates **29** and **30** were obtained in good yield. However, the subsequent reduction step was expectedly complicated. On the one hand, even partial reduction of the tosyl groups in calixarenes **29** and **30** might result in poisoning of a metal catalyst, thus hampering or preventing a catalytic hydrogenation of these compounds. On the other hand, in calixarenes **31** and **32**, which are immediate products of the selective reduction of the nitro groups in compounds **29** and **30** under neutral or basic conditions, the free amino groups may be alkylated by a neighboring tosylate-containing molecule, resulting in undesired polymeric/oligomeric substances. To avoid this, the reduction must be conducted in acidic medium, and no strong base must be applied during the work-up step. Thus, reduction of calixarenes **29** and **30** with tin(II) chloride, similar to the preparation of propargylated calix[4]arene tetraamines **18**–**20**, proceeds under acidic conditions, but it has limited applicability as a complete removal of a large amount of inorganic by-products could only be achieved using alkaline solutions. This causes the unwanted self-conversions of free tetraamines **31** and **32**. Reduction of nitro compounds with activated iron generated from Zn and FeSO_4_ in the presence of NH_4_Cl [98] was found to be a good alternative to the above tin(II) chloride reduction. Under these conditions, *p*-aminocalixarenes **31** and **32** were successfully prepared from the nitrated precursors **29** and **30** using simple filtration to remove the inorganic by-product components from the reaction mixtures. Although the free amines **31** and **32** were pure enough according to their ^1^H NMR data, these compounds were not fully characterized in order to avoid the risk of self-alkylation of the amines during their storage. Thus, the freshly prepared amines **31** and **32** were immediately treated with Boc_2_O and the resultant Boc-protected calix[4]arene tetraamines **33** and **34** bearing four or two 2-tosyloxyethyl groups at the narrow rims were obtained in good yields. By replacement of the tosyloxy groups with the azide ones, the desired tetraazidoethyl and diazidoethyl products **35** and **36** were prepared, thus completing the series of bifunctionalized cone calix[4]arenes having four protected amino groups at the wide rims and a CuAAC-ready propargyl (compounds **24**–**26**) or 2-azidoethyl (compounds **35**, **36**) groups at the narrow rims of the macrocycles.

### Some transformations of propargylated/2-azidoethylated *p*-aminocalix[4]arenes

**Scheme 6:**
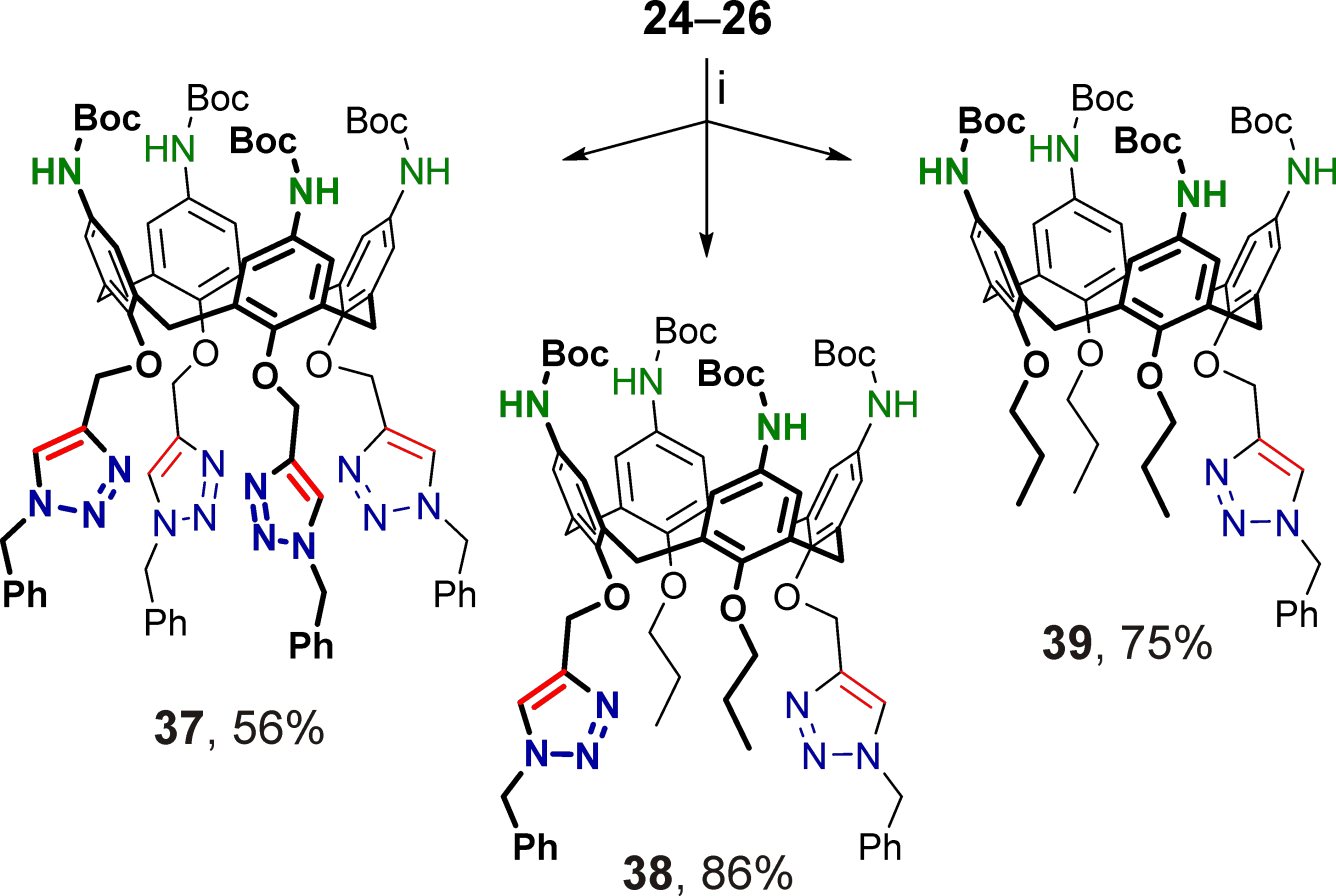
Synthesis of triazolated *p*-aminocalix[4]arenes **37**–**39** from the propargylated calixarene precursors **24**–**26**. Conditions: i) benzyl azide, CuI, Et_3_N, toluene, rt.

**Scheme 7:**
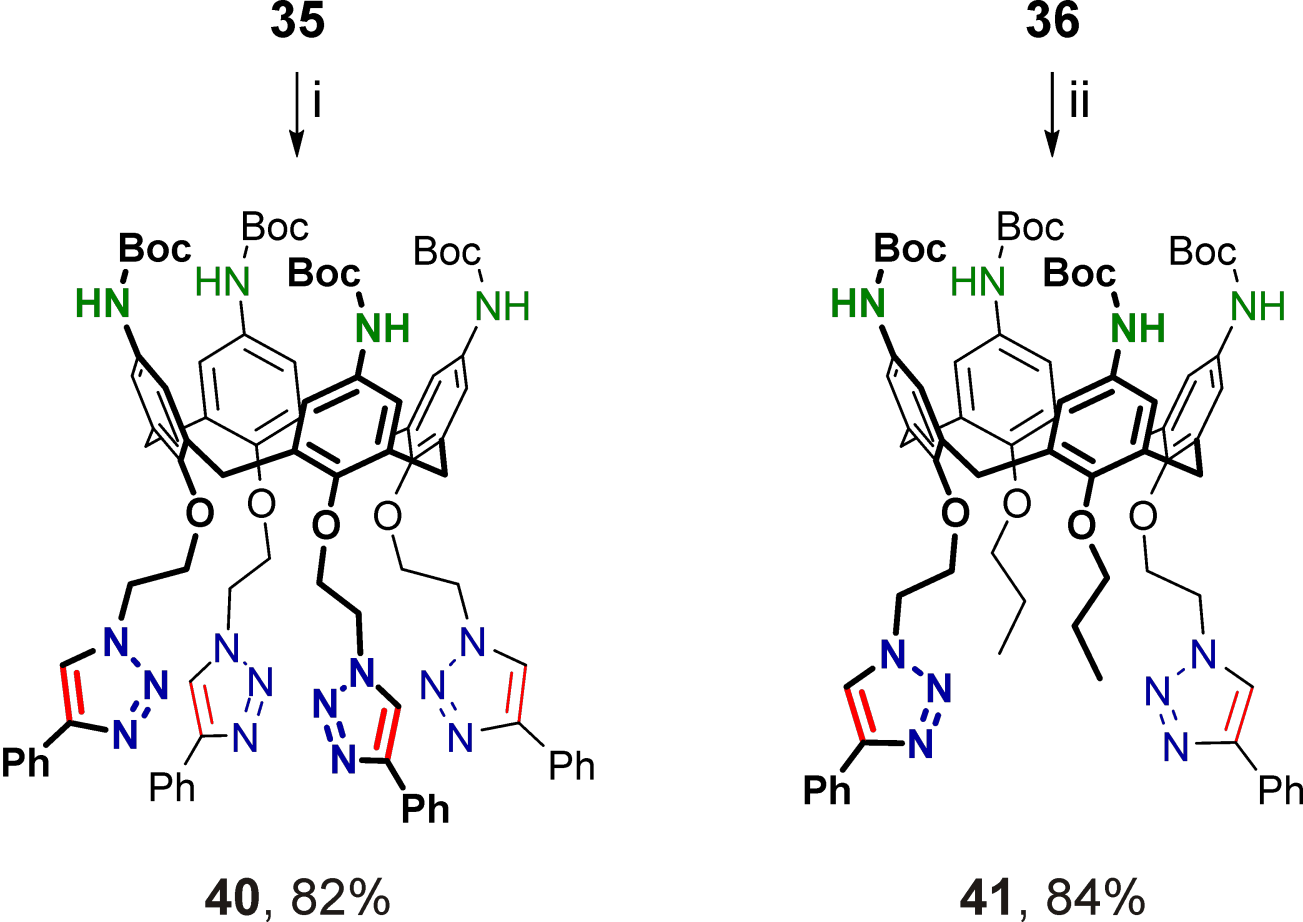
Synthesis of triazolated *p*-aminocalix[4]arenes **40** and **41** from the 2-azidoethylated calixarene precursors **35** and **36**. Conditions: i) phenylacetylene, CuI·P(OEt)_3_, toluene, 100 °C; ii) phenylacetylene, CuI, toluene/Et_3_N, rt.

**Scheme 8:**
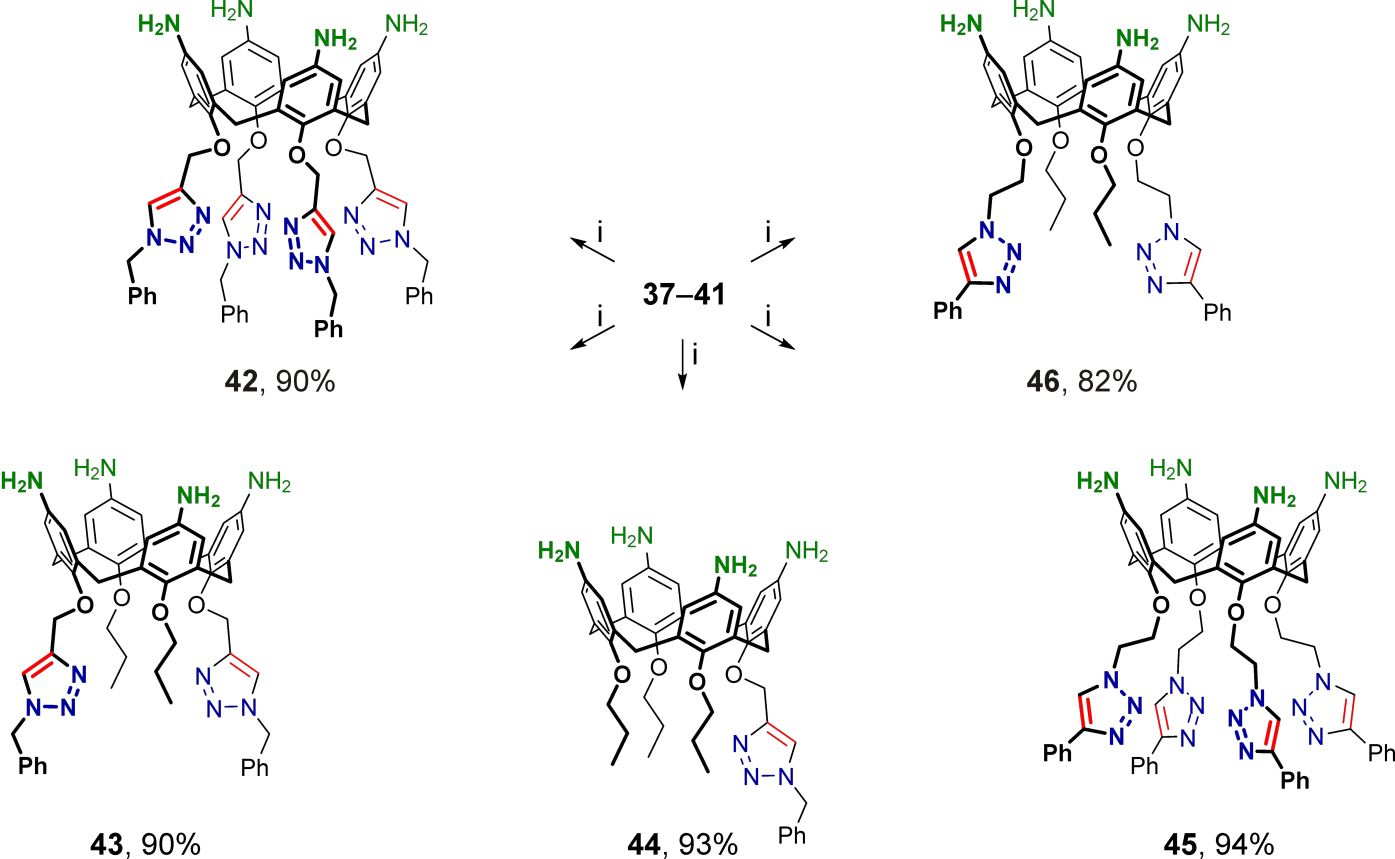
Removal of Boc protection in calixarenes **37**–**41**; (i) CF_3_CO_2_H, dichloromethane, rt, then NaHCO_3_/H_2_O.

**Scheme 9:**
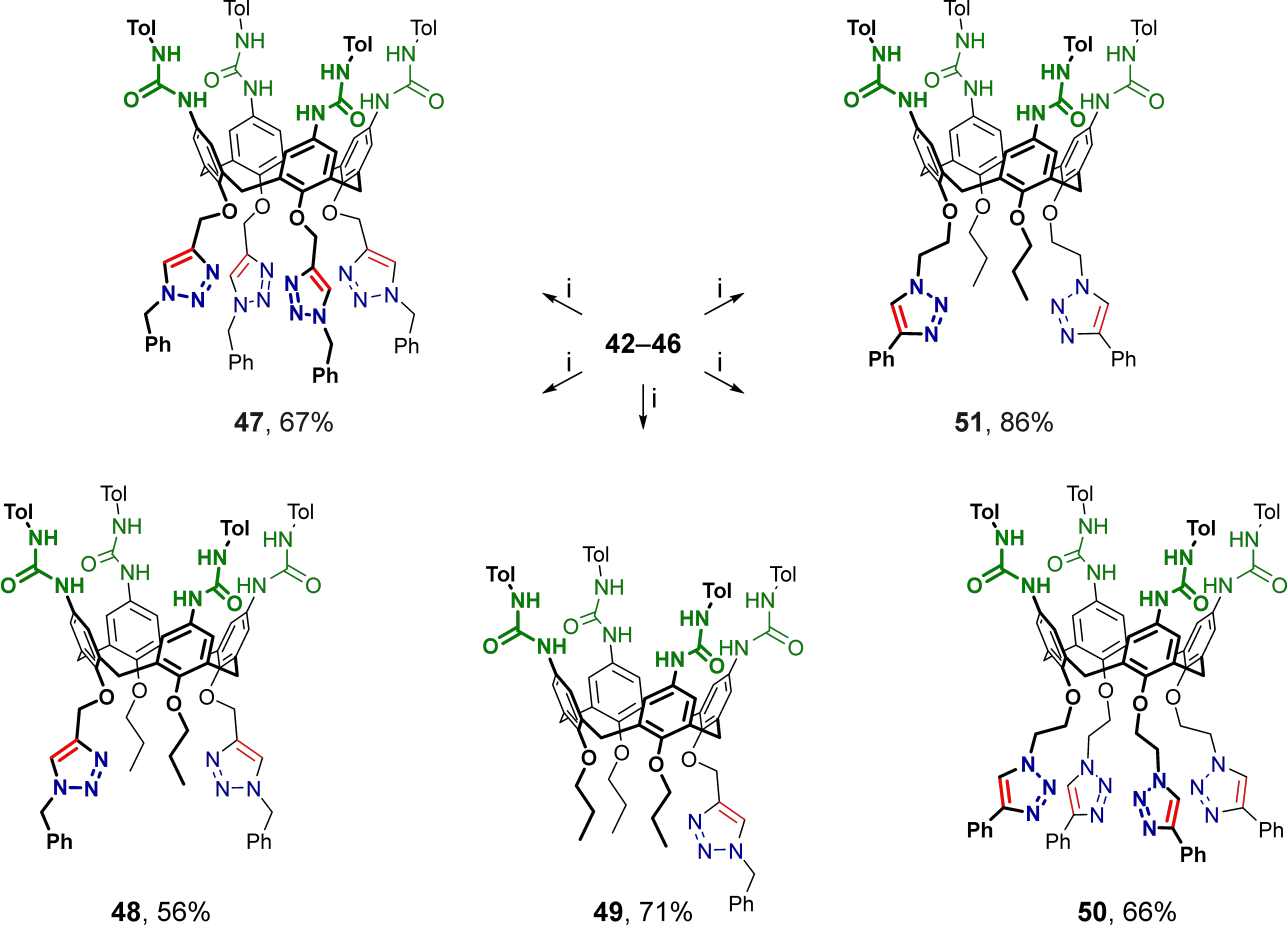
Synthesis of triazolated tetraureacalix[4]arenes **47**–**51**. Conditions: (i) *p*-tolyl isocyanate, toluene, 60 °C.

To evaluate the synthetic potential of the obtained heterobifunctionalized calix[4]arenes, they were subjected to three-step chemical transformations, including the synthesis of triazoles under CuAAC conditions, followed by deprotection of the amino groups, and their conversion to urea moieties (Schemes 6–9).

The propargylated calix[4]arenes **24**–**26** were reacted with benzyl azide in the presence of CuI activated with triethylamine (20 equiv per Cu, Scheme 6). Under these conditions all three reactions were completed in 72 h at room temperature, and the desired Boc-protected *p*-aminocalix[4]arenes **37**, **38**, and **39** having four, two distal or one 1-benzyltriazol-4-ylmethyl moiety at their narrow rims were obtained. The lowered isolated yield of tetratriazole **37** was most likely due to incomplete destruction of its copper complex (although an extraction procedure for complex destruction using Na_2_S_2_O_3_ was applied), which was difficult to separate from the relatively polar free calixarene **37** using column chromatography. In line with this, when toluene-soluble CuI·P(OEt)_3_ was used as a catalyst (in this case heating was required to complete the cycloaddition), an even lower isolated yield of calixarene **37** (≈35%) was observed, indicating that a large portion of Cu^+^ was complexed with the calixarene product when no excess competing ligand such as triethylamine was present in the reaction mixture.

Following our previous findings on the reactivity of 2-azidoethylated calix[4]arenes with arylacetylenes under CuAAC conditions [81], calixarene **35** bearing four 2-azidoethyl groups at the narrow rim was reacted with phenylacetylene under heating using CuI·P(OEt)_3_ as a catalyst (Scheme 7). In contrast to the synthesis of calixarene **37**, there were no problems encountered with the copper complex destruction and the yield of the isomeric calixarene **40** having four 2-(4-phenyltriazol-1-yl)ethyl moieties at the narrow rim was much higher, indicating a weaker binding in the **40**/Cu^+^ system than in the **37**/Cu^+^ one, due to the outer arrangement of the triazole N(3)-atoms relative to the calixarene core in compound **40**.

It was established previously, that *p-tert*-butylcalix[4]arene having two distal 2-azidoethyl groups at the narrow rim could not efficiently react with phenylacetylene under copper(I) catalysis unless a huge excess of Cu^+^-competing triethylamine is added to the system. This is most likely due to specific interactions of the diazide with polymeric/oligomeric copper(I) phenylacetylide hindering the cycloaddition [81]. To avoid similar issues, the Boc-protected *p*-aminocalix[4]arene **36** was reacted with phenylacetylene in the presence of CuI using a mixture of toluene and triethylamine (4:1) as solvent. Under these conditions, the reaction proceeded smoothly at room temperature and the desired ditriazole **41** was obtained in high yield (Scheme 7).

The *tert*-butoxycarbonyl groups in calix[4]arenes **37**–**41** were cleaved under standard conditions using trifluoroacetic acid, and after basification, free *p*-aminocalix[4]arenes **42**–**46** substituted at the narrow rims with either 1-benzyltriazol-4-ylmethyl or 2-(4-phenyltriazol-1-yl)ethyl groups were obtained (Scheme 8). Then, amines **42**–**46** were reacted with excess *p*-tolyl isocyanate to afford the corresponding tetraureacalix[4]arenes **47**–**51** (Scheme 9), the structures of which were unambiguously confirmed by their ^1^H NMR and ^13^C NMR spectra obtained from solutions in polar DMSO-*d*_6_.

Being calix[4]arene tetraureas, compounds **47**–**51** should form homodimeric capsules in H-bond non-competing solvents (e.g., chloroform) with the inclusion of one of the solvent molecules into the capsules. However, triazole heterocycles attached to the calixarene core can serve as weak hydrogen-bond acceptors [99] and thus may interfere with capsule formation, including the exclusive formation of heterodimeric capsules from the calixarene tetraaryl- and tetraarylsulfonylureas if co-dissolved in a 1:1 molar ratio [51–53]. Furthermore, tetraureas **47**–**51** contain relatively bulky substituents at the phenolic oxygen atoms, which may influence the conformational behavior of the macrocycle (e.g., pinched cone-to-pinched cone conformational interconversions), thereby favoring or disfavoring the *C*_4v_-symmetric ‘ideal’ cone conformation of the calixarene core required for capsule formation [59]. To check this, ^1^H NMR spectra from the CDCl_3_ solutions of multifunctionalized calixarenes **47**–**51** and their 1:1 mixtures with the known *O*-pentylated tetratosylureacalix[4]arene **52** [51] were acquired and analyzed.

**Figure 4:**
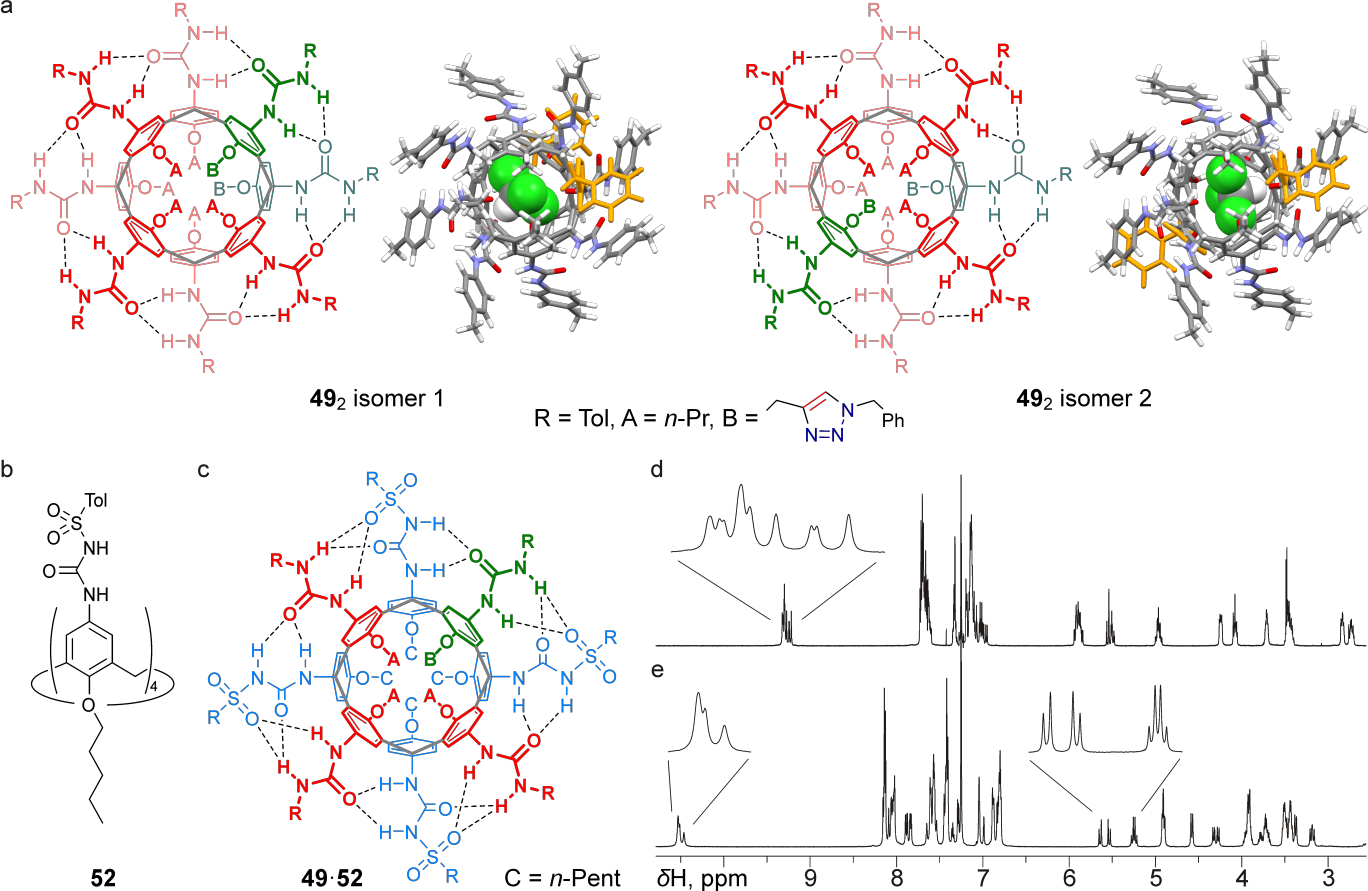
Planar and energy-minimized structures (with CHCl_3_ molecule included and triazole groups highlighted) of isomeric homodimers of tetraurea **49** (a); structure of tetratosylureacalix[4]arene **52** (b); planar structure of heterodimer **49**·**52** (c); fragment of the ^1^H NMR spectrum of calixarene **49** in CDCl_3_ (d); fragment of the ^1^H NMR spectrum of an equimolar mixture of calixarenes **49** and **52** in CDCl_3_ (e). All spectra were acquired using a 600 MHz NMR spectrometer.

In terms of time-averaged molecular symmetry, tetraureacalix[4]arenes of three types were prepared in this work: *C*_s_-symmetric calixarene **49** with one triazole heterocycle at the narrow rim, *C*_2v_-symmetric calixarenes **48** and **51** with pairs of distal triazole groups, and *C*_4v_-symmetric tetraureas **47** and **50**. Due to the phenomenon of supramolecular chirality caused by the direction of hydrogen bonds in the capsules [100], tetraureacalixarenes of the first type should form two regioisomeric homodimers possessing *C*_1_-symmetry or a single heterodimer with a *C*_4v_-symmetric tetratosylureacalix[4]arene **52** (each as a pair of enantiomers). Similarly, the *C*_2v_-symmetric tetraureacalixarenes of the second type should furnish *C*_2_-symmetric homo- or heterodimers as single regioisomers (each as a pair of enantiomers), whereas homo- and heterodimers formed by *C*_4v_-symmetric tetraureas of the third type are expected to have *S*_8_ (achiral meso) and *C*_4_ (as two enantiomers) symmetry, respectively. These features must immediately affect the signal patterns in the ^1^H NMR spectra of triazolated tetraureacalix[4]arenes and may also complicate their interpretation. Indeed, the ^1^H NMR spectrum of the triazolated tetraurea **49** obtained from its solution in CDCl_3_ turned out to be overcomplicated (see Figure 4). The spectral pattern apparently arises from the superposition of signals from two asymmetric homodimers, **49**_2_ isomer 1 and **49**_2_ isomer 2 (Figure 4a), which are present in a ratio of ≈1:1. The ratio of the isomers cannot be easily obtained from the spectrum except by analyzing the group of the most downfield-shifted signals corresponding to the half of the urea NHs involved in hydrogen bonds. According to the symmetry features discussed above, this group of signals should consist of eight singlets from each of the isomeric homodimers, thus giving sixteen partially overlapping signals (see insert in Figure 4d). Integration of the resolved signals of this group showed that their relative intensities are almost exactly multiples of 1, which is most likely in the case where the entire group is formed by sixteen signals of the same intensity.

In support of this, the energy-minimized structures of two regioisomeric capsules, **49**_2_ isomer 1 and **49**_2_ isomer 2, each containing a chloroform molecule (Figure 4a, PBE0/def2-SVP, gCP, D4, SMD (chloroform), ORCA 6.0.1 package [101–109]), were obtained and the calculated difference in their full-electron energies was found to be less than 0.1 kcal mol^−1^, which is well below the error threshold. Thus, in contrast to tetraureas containing several different substituents in the urea groups [100], the presence of one bulky moiety at the narrow rim of the tetraureacalix[4]arene does not lead to a significant difference in the stability of the homodimers formed by compound **49**, at least in a chloroform solution. To further confirm the ability of calixarene **49** to form capsules, the ^1^H NMR spectrum of its equimolar mixture with tetratosylureacalix[4]arene **52** in CDCl_3_ was also analyzed (Figure 4e). Despite the presence of a second calixarene molecule, the ^1^H NMR spectrum turned out to be simplified compared to the spectrum of homodimers **49**_2_, which is due to the formation of only one heterodimer **49**·**52**. The spectrum clearly shows even more downfield-shifted resonances from the four NH groups involved in hydrogen bonding with the sulfonyl groups, as well as clear signals from the two AB spin systems of the methylene groups attached to the single triazole moiety, in which the protons have become diastereotopic due to the supramolecular chirality of the heterodimeric capsule (see inserts in Figure 4e).

**Figure 5:**
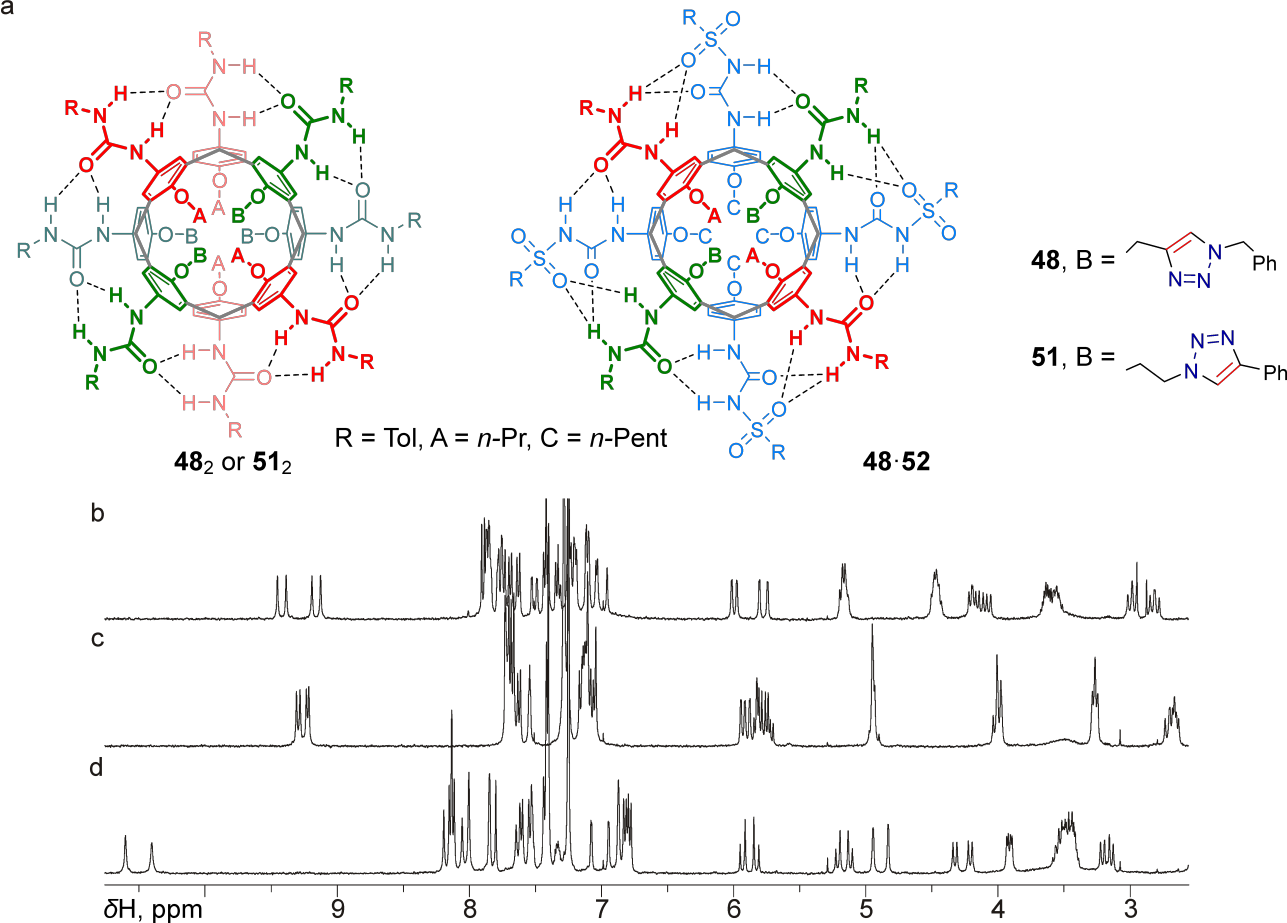
Planar structures of homodimers **48**_2_, **51**_2_, and heterodimer **48**·**52** (a); fragment of the ^1^H NMR spectrum of calixarene **51** in CDCl_3_ (b); fragment of the ^1^H NMR spectrum of calixarene **48** in CDCl_3_ (c); fragment of the ^1^H NMR spectrum of an equimolar mixture of calixarenes **48** and **52** in CDCl_3_ (d). All spectra were acquired using a 400 MHz NMR spectrometer.

Due to the higher symmetry of both homo- and heterodimeric capsules formed by ditriazolated tetraureas **48** and **51** (Figure 5), the ^1^H NMR spectra acquired from solutions of these compounds or their mixtures with tetrasulfonylurea **52** in CDCl_3_ turned out to be much more interpretable. In particular, the spectra of the homodimers (Figure 5b,c) contained four clearly recognizable downfield-shifted singlets from hydrogen-bonded NH groups, and all other signals appearing in the spectra corresponded to chiral *C*_2_-symmetric homodimeric capsules in both cases. The ^1^H NMR spectrum of the equimolar mixture **48**/**52** (taken as an example) contained one set of signals from the *C*_2_-symmetric heterodimer **48**·CDCl_3_·**52**, but not from the homodimers **48**·CDCl_3_·**48** and **52**·CDCl_3_·**52**, and thus reflected the expected exclusive formation of heterocapsular assemblies, which was thus not hindered by the two triazole heterocycles arranged at the narrow rim of the calixarene core.

**Figure 6:**
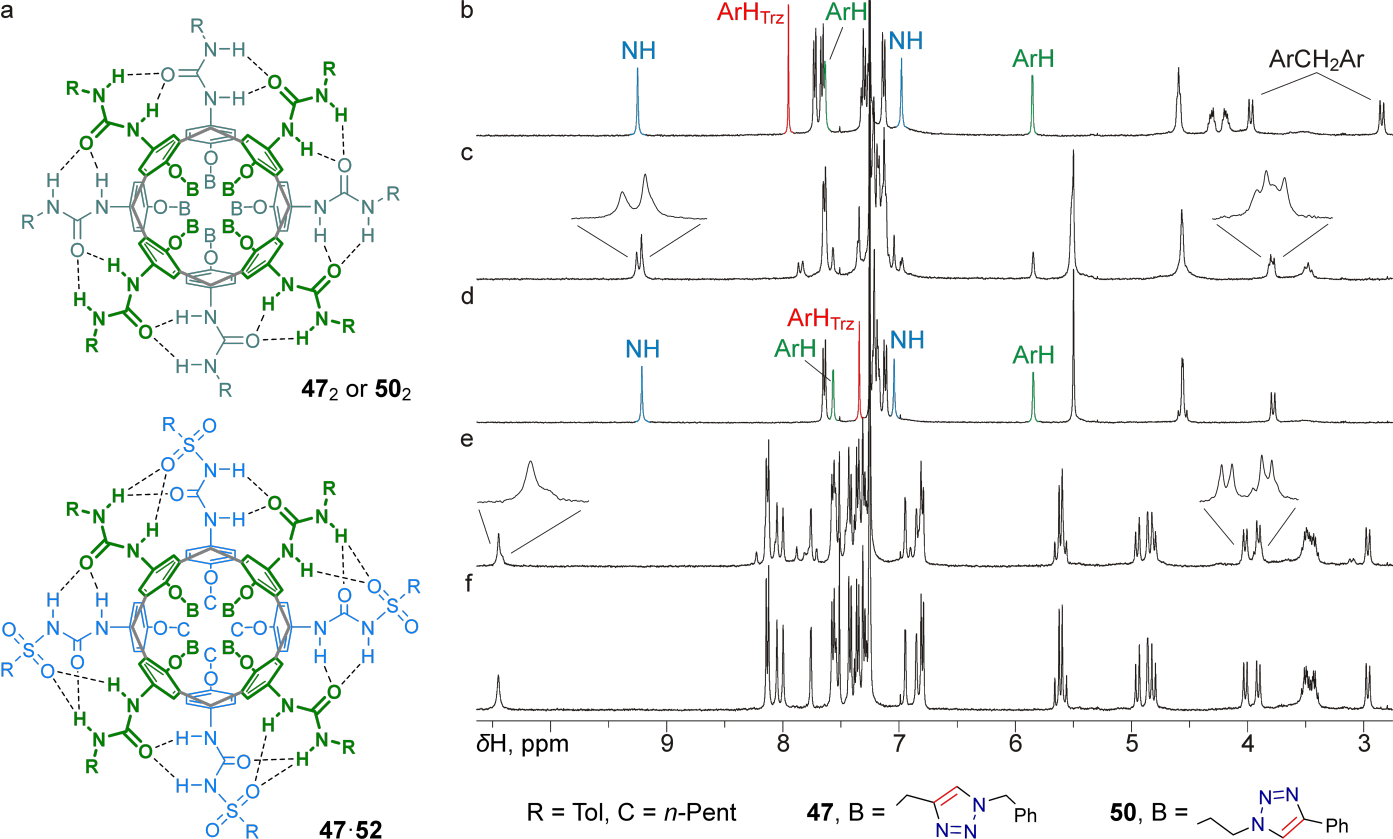
Planar structures of homodimers **47**_2_, **50**_2_, and heterodimer **47**·**52** (a); fragment of the ^1^H NMR spectrum of calixarene **50** in CDCl_3_ (b); fragment of the ^1^H NMR spectrum of calixarene **47** in CDCl_3_ (c); fragment of the ^1^H NMR spectrum of the same sample washed with water (d); fragment of the ^1^H NMR spectrum of an equimolar mixture of calixarenes **47** and **52** in CDCl_3_ (e); fragment of the ^1^H NMR spectrum of the same sample washed with water (f). All spectra were acquired using a 400 MHz NMR spectrometer.

Except for a slight broadening of the signals caused by the limited solubility of the compound, the ^1^H NMR spectrum obtained for a CDCl_3_ solution of tetraureacalixarene **50** having four 2-(4-phenyltriazol-1-yl)ethyl groups at the narrow rim was in good agreement with the expected spectrum of homodimer **50**_2_ possessing *S*_8_ symmetry (Figure 6a). Indeed, the spectrum contained two ^4^*J*-coupled doublets from the diastereotopic aromatic protons of the calixarene core and just one pair of ^2^*J*-coupled doublets from its methylene bridges, two well-distant singlets from the urea NHs participating in the hydrogen-bond belt, one resonance from the triazole protons, as well as only one set of signals from the tolyl and phenyl groups, which confirms the formation of homodimer **50**_2_ (Figure 6b).

Quite surprisingly, such a clear pattern was not observed in the ^1^H NMR spectrum of a CDCl_3_ solution of tetraurea **47**, which is an isomer of calixarene **50**, having 1-benzyltriazol-4-ylmethyl groups replacing 2-(4-phenyltriazol-1-yl)ethyl groups, and thus possessing less flexible linkers between the calixarene core and the differently arranged triazole heterocycles. In addition to what could be interpreted as a set of signals from homodimer **47**_2_, a set of broadened resonances was observed in the spectrum (Figure 6c). Neither prolonged heating of the sample at 50 °C before the spectrum acquisition, nor measuring the spectrum at elevated temperature resulted in a simplification of the spectral pattern, indicating no significant contribution of any slow rotational/conformational motions to the complexity of the spectrum. Similar additional signals were observed in the ^1^H NMR spectrum of an equimolar mixture of calixarenes **47** and **52** in CDCl_3_ (Figure 6e). Reasonably, additional non-covalent interactions possibly occurred between two or more calixarene molecules, partially destroying the dimeric capsules and promoting the formation of more complex assemblies, which was characteristic exclusively of calixarene **47** (of the five calixarene tetraureas studied). Since nothing other than hydrogen bonds involving triazole heterocycles could be proposed for such non-covalent interactions, a drop of methanol-*d*_4_ was added to a solution of tetraurea **47** in CDCl_3_ to break these additional bonds. However, the effect of adding this polar solvent turned out to be too strong, and the resulting spectrum only contained signals from the tetraurea monomer **47**, indicating the disruption of intermolecular hydrogen bonds between the urea moieties as well.

Still, it was discovered by chance that simply washing of a solution of calixarene **47** in CDCl_3_ with water solved the issue. Indeed, the ^1^H NMR spectrum of the sample obtained after separation of the aqueous phase turned out to be easily interpretable (Figure 6d) and contained a set of signals from homodimer **47**_2_ similar to those in the spectrum of homodimer **50**_2_. A drastic simplification of the spectrum was also observed for a water-washed solution of a mixture of calixarenes **47** and **52** in CDCl_3_, and only clear resonances from the heterodimer **47**·**52** were detected there (Figure 6f). In a separate series of experiments, the ^1^H NMR spectra of unstable emulsions obtained by shaking solutions of calixarenes **47**–**51** in CDCl_3_ with water were rapidly collected. Although lowered-resolution NMR spectra were obtained, in all cases only resonances of the respective homodimers were detected, despite the presence in the spectra of a large signal from the aqueous phase (at ≈4.8 ppm) along with a signal from dissolved water (1.56 ppm). Considering the low solubility of water in chloroform, the stability of the capsules in water-saturated CDCl_3_ seems reasonable, because the dimers are held together by at least 16 hydrogen bonds between the urea groups. On the other hand, this water content is apparently sufficient to disrupt weaker intermolecular contacts involving triazole heterocycles as hydrogen-bond acceptors. Since the structures of the above mentioned non-capsular aggregate(s) formed by calixarene **47** cannot be drawn based on the available data, it is unclear why only four 1-benzyltriazol-4-ylmethyl groups attached to the narrow rim of the tetraurea calixarene are responsible for the unwanted aggregation, whereas one or two 1-benzyltriazol-4-ylmethyl groups (calixarenes **48**, **49**) or two or four 2-(4-phenyltriazol-1-yl)ethyl groups (calixarenes **50**, **51**) do not interfere with the capsule formation.

Due to the mentioned limited solubility of calixarene **50** in CDCl_3_, it was not possible to conduct an accurate experiment to study any preferential heterodimerization between isomeric tetraureas **47** and **50**, which possess a set of narrow rim substituents of different flexibility. However, preliminary NMR data showed that a turbid CDCl_3_ solution of both calixarenes, taken in a more or less equimolar ratio, contained signals from both homodimers **47**_2_ and **50**_2_, a set of broadened signals from non-capsular aggregates (readily removed from the spectrum by washing the solution with water) and a set of signals that could be interpreted as those from heterodimer **47**·**50**. Thus, the difference in the spatial preorganization of the four urea groups in calixarenes **47** and **50**, caused by the different constraints related to their narrow-rim substituents, is apparently not sufficient to provide any significant selectivity for the formation of homo/heterocapsules.

Finally, since the triazole groups arranged at the narrow rim of the calix[4]arene macrocycle are capable of complexation with transition-metal cations, showing different complexation preferences depending on the triazole attachment mode [81], the attractive idea of combining complexation and capsule formation using triazolated tetraureas was explored. For preliminary NMR complexation experiments, zinc and silver cations were chosen to target 1-benzyltriazol-4-ylmethyl- and 2-(4-phenyltriazol-1-yl)ethyl-decorated calix[4]arenes, respectively, but unfortunately this study was unsuccessful due to solubility issues. Indeed, CD_3_CN or its mixtures with CDCl_3_, in which Zn(ClO_4_)_2_·6H_2_O is soluble, turned out to be solvents which were too polar and destroyed dimers **47**_2_ or **48**_2_, although some changes in the ^1^H NMR spectra of monomers **47** and **48** could be detected upon addition of the salt. Attempts to conduct the experiment using solid–liquid extraction in neat CDCl_3_ were also unsuccessful, since no changes in the ^1^H NMR spectrum of capsule **47**_2_ were detected upon the addition of excess solid Zn(ClO_4_)_2_·6H_2_O, even after prolonged storing of the mixture at room temperature or under moderate (55 °C) heating. Although anhydrous AgPF_6_, which is soluble in non-polar solvents, was used for trial binding experiments with 2-(4-phenyltriazol-1-yl)ethyl-containing capsules **50**_2_ and **51**_2_, no data on the complexation processes were obtained in these cases either; adding excess salt to solutions of these calixarenes in CDCl_3_ or in CDCl_3_/benzene-*d*_6_ mixtures resulted in the formation of insoluble precipitates containing the entire portions of the hosts, thus indicating a complexation process but the concentrations of dissolved compounds was too low for NMR analysis. Thus, additional experiments need to be designed to reveal whether the triazolated tetraureacalix[4]arenes can be used to include pairs of triazole-bound cations into supramolecules via intermolecular hydrogen bonding between the urea groups.

## Conclusion

We have expanded the functionalization capabilities of cone calix[4]arenes by combining orthogonally transformable functional groups of two types within a single macrocyclic core. Both wide and narrow rims of the calixarene macrocycles were used to introduce four amino groups along with propargyl or 2-azidoethyl groups into the molecules. This allowed for further step-wise functionalization of the core using CuAAC ‘click’ reactions involving alkyne or azide moieties and amine acylation or similar reactions. Five heteromultifunctional *N*-protected *p*-aminocalix[4]arenes comprising one/two/four propargyl groups or two/four 2-azidoethyl groups at the narrow rims of the macrocycles were prepared using synthetic strategies that involved wide rim *ipso*-nitration of prefunctionalized macrocycles (containing either silyl-protected propargyl groups or the synthetic precursors of 2-azidoethyl groups) followed by reduction of the exhaustively nitrated products. During thorough fine-tuning of the nitration conditions, several partially nitrated calix[4]arene propargyl ethers bearing *tert*-butyldimethylsilyl protecting groups in the alkyne moieties were also obtained and their exact structures were determined using 2D NMR and X-ray diffraction data, contributing to a general understanding of the selectivity of nitration when applied to calixarenes containing bulky narrow-rim substituents.

The functionalization capabilities of the propargylated/2-azidoethylated *p*-aminocalix[4]arenes were demonstrated by their facile conversion into the narrow-rim triazolated macrocycles of two types (differing in the mutual arrangement of the calixarene core and the triazole heterocycles) and then into the wide-rim tetraurea derivatives. A preliminary study of the aggregation of triazolated calix[4]arene tetraureas showed that the triazole groups, despite their relatively large size and hydrogen-bond accepting ability, have only a minor effect on the formation of homo/heterodimeric capsules by these compounds in chloroform. Thus, a variety of functional units can be introduced into the narrow rims of the developed propargylated/2-azidoethylated calixarenes using CuAAC reactions, and the resulting triazolated calixarenes can be further functionalized at their amino groups, including the preparation of self-assembled capsular aggregates of virtually unlimited complexity.

## Supporting Information

File 1 Synthesis details, copies of ^1^H and ^13^C NMR spectra of novel compounds, details of X-ray diffraction measurements and crystal structure data.

File 2 Crystallographic information file for compound **16**.

File 3 Crystallographic information file for compound **15**.

## Data Availability

All data that supports the findings of this study is available in the published article and/or the supporting information of this article.
