# Supplementary material for: Cone p-aminocalix[4]arenes enriched with ‘clickable’ alkyne or azide functionalities
Source: Beilstein J Org Chem. 2026 Mar 9;22:399–415. doi: 10.3762/bjoc.22.28 (PMC12990471; doi:10.3762/bjoc.22.28)
Supplement: File 1 — Synthesis details, copies of 1H and 13C NMR spectra of novel compounds, details of X-ray diffraction measurements and crystal structure data. [file Beilstein_J_Org_Chem-22-399-s001.pdf]

BEILSTEIN JOURNAL OF ORGANIC CHEMISTRY

Supporting Information

for

Cone *p*-aminocalix[4]arenes enriched with ‘clickable’ alkyne or azide functionalities

Ilia Korniltsev, Vasily Bazhenov, Alexander Gorbunov, Dmitry Cheshkov, Stanislav Bezzubov, Vladimir Kovalev and Ivan Vatsouro

Beilstein J. Org. Chem. **2026,** *22,* 399–415. doi:10.3762/bjoc.22.28

Synthesis details, copies of ^{1}H and ^{13}C NMR spectra of novel compounds, details of X-ray diffraction measurements and crystal structure data

License and Terms: This is a supporting information file under the terms of the Creative Commons Attribution License (https://creativecommons.org/licenses/by/4.0). Please note that the reuse, redistribution and reproduction in particular requires that the author(s) and source are credited and that individual graphics may be subject to special legal provisions.
The license is subject to the *Beilstein Journal of Organic Chemistry* terms and conditions: (https://www.beilstein-journals.org/bjoc/terms)

Table of contents

Synthesis and characterization of novel compounds S2

NMR spectra of novel compounds S22

Details of X-ray diffraction measurements S61

References S63

Synthesis and characterization of novel compounds

General experimental methods: ^{1}H and ^{13}C NMR spectra were acquired on Bruker Avance 400 and Avance 600 instruments at 20 ºC and chemical shifts are reported as ppm referenced to solvent signals. ESI mass spectra were obtained from Sciex TripleTOF 5600+. Chemicals received from commercial sources were used without further purification. Calixarenes **1**,[S1] **2**,[S2] **3**,[S3] **4**,[S4] **5**,[S3] **27**,[S5, S6] and **28**[S7] were prepared according to the published procedures.

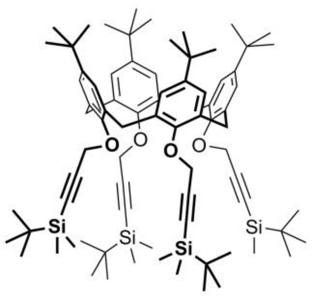

*Silylated tetrapropargyl ether **6**.*[S8] Under argon, a hexane solution of *n*-BuLi (2.5 M, 7.20 mL, 18.00 mmol) was added dropwise to a cooled (−95 °C) solution of bis(trimethylsilyl)amine (4.15 g, 19.86 mmol) in dry THF (20 mL) at stirring. The mixture was slowly warmed to room temperature, stirred for 1 h, and then cooled again (−95 °C). A solution of calixarene **1** (2.40 g, 3.00 mmol) in dry THF (40 mL) was added dropwise with stirring, and the mixture was stirred at cooling for 30 min. A solution of *tert*-butyldimethylsilyl chloride (2.71 g, 18.00 mmol) in dry THF (10 mL) was added, the mixture was warmed to room temperature and stirred overnight. Saturated solution of NH_4Cl (100 mL) was added to the reaction mixture, the organic phase was separated, the aqueous phase was washed with dichloromethane. The combined organic phases were washed with water, dried and evaporated to dryness. The residue was recrystallized from a dichloromethane/methanol mixture. Yield 3.40 g (90%), white crystals. ^{1}H NMR spectrum of the obtained compound was identical to the previously published one.[S8]

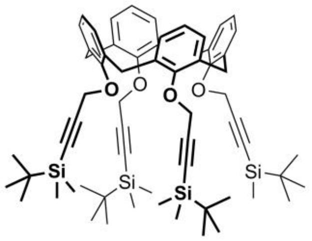

*Silylated tetrapropargyl ether **7*** was prepared as described for compound **6** from calixarene **2** (1.21 g, 2.10 mmol), *n*-BuLi solution (2.5 M in hexane, 5.05 mL, 12.63 mmol), bis(trimethylsilyl)amine (2.24 g, 13.88 mmol) and *tert*-butyldimethylsilyl chloride (1.90 g, 12.61 mmol) in dry THF (95 mL). Yield 1.57 g (72%), beige crystals. M.p. 166–168 °C. ^{1}H NMR (400 MHz, $CDCl_3$): δ = 6.71–6.66 (m, 8H; ArH), 6.64–6.58 (m, 4H; ArH), 4.79 (s, 8H; OCH_2C), 4.57 (d, 4H, ^2J = 13.3 Hz; $ArCH_2Ar$), 3.17 (d, 4H, ^2J = 13.3 Hz; $ArCH_2Ar$), 0.88 (s, 36H; $C(CH_3)_3$), 0.07 (s, 24H; $Si(CH_3)_2$) ppm. ^{13}C NMR (100 MHz, $CDCl_3$): δ = 154.61, 135.62 (C_{Ar}), 128.01, 122.95 (CH_{Ar}), 102.82 ($OCH_2\underline{C}C$), 89.75 ($OCH_2C\underline{C}$), 61.50 ($O\underline{C}H_2CC$), 32.07 ($ArCH_2Ar$), 26.05 ($C(\underline{C}H_3)_3$), 16.40 ($\underline{C}(CH_3)_3$), –4.68 ($Si(CH_3)_2$) ppm. ESI-MS *m*/*z*: 1033.5822 $[M+H]^+$ for $C_{64}H_{89}O_4Si_4$ (1033.5832).

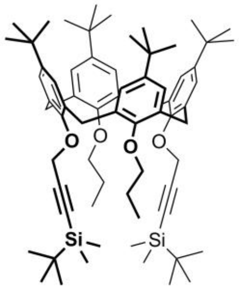

*Silylated dipropargyl ether **8*** was prepared as described for compound **6** from calix[4]arene **3** (2.30 g, 2.84 mmol), *n*-BuLi solution (2.5 M in hexane, 3.41 mL, 8.53 mmol), bis(trimethylsilyl)amine (1.53 g, 9.48 mmol) and *tert*-butyldimethylsilyl chloride (1.29 g, 8.53 mmol) in dry THF (120 mL). Yield 2.77 g (94%), white crystals. M.p. 245–247 °C. ^{1}H NMR (400 MHz, $CDCl_3$): δ = 7.06 (s, 4H; ArH), 6.41 (s, 4H; ArH), 5.04 (s, 4H; OCH_2C), 4.47 (d, 4H, ^{2}J = 12.7 Hz; $ArCH_2Ar$), 3.70–3.61 (m. 4H; $OC\underline{H}_2CH_2$), 3.10 (d, 4H, ^{2}J = 12.7 Hz; $ArCH_2Ar$), 2.06–1.93 (m, 4H; $OCH_2C\underline{H}_2$), 1.31 (s, 18H; $C(CH_3)_3$), 1.07 (t, 6H, ^{3}J = 7.5 Hz; $CH_2C\underline{H}_3$), 0.81 (s, 18H; $C(CH_3)_3$), 0.79 (s, 18H; $C(CH_3)_3$), 0.00 (s, 12H; $Si(CH_3)_2$) ppm. ^{13}C NMR (100 MHz, $CDCl_3$): δ = 153.03, 151.47, 145.44, 144.04, 136.87, 132.00 (C_{Ar}), 125.01, 124.26 (CH_{Ar}), 103.92 ($OCH_2\underline{C}C$), 89.67 ($OCH_2C\underline{C}$), 77.34 ($O\underline{C}H_2CH_2$), 59.39 ($O\underline{C}H_2CC$), 34.06, 33.50 ($\underline{C}(CH_3)_3$), 32.15 ($ArCH_2Ar$), 31.69, 31.08 ($ArC(\underline{C}H_3)_3$), 25.97 ($SiC(\underline{C}H_3)_3$), 23.81 ($\underline{C}H_2CH_3$), 16.39 ($Si\underline{C}(CH_3)_3$), 10.79 ($CH_2\underline{C}H_3$), –4.76 ($Si(CH_3)_2$) ppm. ESI-MS *m*/*z*: 1037.7230 $[M+H]^+$ for $C_{68}H_{101}O_4Si_2$ (1037.7233).

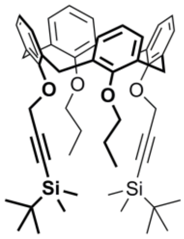

*Silylated dipropargyl ether **9*** was prepared as described for compound **6** from calix[4]arene **4** (5.18 g, 8.87 mmol), *n*-BuLi solution (2.5 M in hexane, 10.64 mL, 26.60 mmol), bis(trimethylsilyl)amine (4.72 g, 29.26 mmol) and *tert*-butyldimethylsilyl chloride (4.01 g, 26.60 mmol) in dry THF (100 mL). Yield 6.39 g (89%), beige crystals. M.p. 183–185 °C. ^{1}H NMR (400 MHz, $CDCl_3$): δ = 7.09 (d, 4H, ^{3}J = 7.5 Hz; ArH), 6.92 (t, 2H, ^{3}J = 7.5 Hz; ArH), 6.31–6.23 (m, 6H; ArH), 5.01 (s, 4H; OCH_2C), 4.51 (d, 4H, ^{2}J = 13.3 Hz; $ArCH_2Ar$), 3.71–3.64 (m, 4H; $OC\underline{H}_2CH_2$), 3.14 (d, 4H, ^{2}J = 13.3 Hz; $ArCH_2Ar$), 2.03–1.91 (m, 4H; $OCH_2C\underline{H}_2$), 1.09 (t, 6H, ^{3}J = 7.5 Hz; $CH_2C\underline{H}_3$), 0.83 (s, 18H; $C(CH_3)_3$), 0.01 (s, 12H; $Si(CH_3)_2$) ppm. ^{13}C NMR (100 MHz, $CDCl_3$): δ = 155.31, 154.52, 138.00, 133.18 (C_{Ar}), 128.43, 127.44, 123.00, 122.16 (CH_{Ar}), 103.17 ($OCH_2\underline{C}C$), 90.01 ($OCH_2C\underline{C}$), 77.12 ($O\underline{C}H_2CH_2$), 59.84 ($O\underline{C}H_2CC$), 31.51 ($ArCH_2Ar$), 25.92 ($C(\underline{C}H_3)_3$), 23.70 ($\underline{C}H_2CH_3$), 16.35 ($\underline{C}(CH_3)_3$), 10.83 ($CH_2\underline{C}H_3$), –4.79 ($Si(CH_3)_2$) ppm. ESI-MS *m*/*z*: 813.4723 $[M+H]^+$ for $C_{52}H_{69}O_4Si_2$ (813.4729).

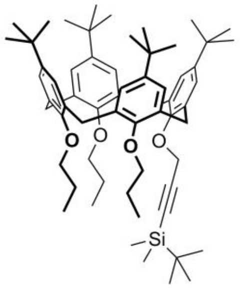

*Silylated propargyl ether **10*** was prepared as described for compound **6** from calix[4]arene **5** (7.02 g, 8.65 mmol), *n*-BuLi solution (2.5 M in hexane, 5.27 mL, 13.18 mmol), bis(trimethylsilyl)amine (2.38 g, 14.74 mmol) and *tert*-butyldimethylsilyl chloride (1.99 g, 13.18 mmol) in dry THF (120 mL). Yield 6.77 g (85%), white crystals. M.p. 174–176 °C. ^{1}H NMR (400 MHz, $CDCl_3$): δ = 7.05 (s, 2H; ArH), 6.99 (s, 2H; ArH), 6.51 (d, 2H, ^{4}J = 2.5 Hz; ArH), 6.45 (d, 2H, ^{4}J = 2.5 Hz; ArH), 5.05 (s, 2H; $OC\underline{H}_2CC$), 4.45 (d, 2H, ^{2}J = 12.8 Hz;

$ArCH_2Ar$), 4.40 (d, 2H, ^2J = 12.4 Hz; $ArCH_2Ar$), 3.93–3.87 (m, 2H; $OC\underline{H}_2CH_2$), 3.73–3.61 (m, 4H; $OC\underline{H}_2CH_2$), 3.11 (d, 2H, ^2J = 12.4 Hz; $ArCH_2Ar$), 3.09 (d, 2H, ^2J = 12.8 Hz; $ArCH_2Ar$), 2.17–2.04 (m, 2H; $OCH_2C\underline{H}_2$), 2.03–1.86 (m, 4H; $OCH_2C\underline{H}_2$), 1.28 (s, 9H; $C(CH_3)_3$), 1.27 (s, 9H; $C(CH_3)_3$), 1.05 (t, 6H, ^3J = 7.5 Hz; $CH_2C\underline{H}_3$), 0.95 (t, 3H, ^3J = 7.5 Hz; $CH_2C\underline{H}_3$), 0.84 (s, 18H; $SiC(CH_3)_3$), 0.80 (s, 9H; $C(CH_3)_3$), 0.00 (s, 6H; $Si(CH_3)_2$) ppm. ^{13}C NMR (100 MHz, $CDCl_3$): δ = 154.40, 153.07, 151.58, 145.18, 144.59, 143.98, 136.54, 135.42, 132.47, 132.11 (C_{Ar}), 125.30, 124.95, 124.49, 124.31 (CH_{Ar}), 104.15 ($OCH_2\underline{C}C$), 89.58 ($OCH_2C\underline{C}$), 77.38, 76.71 ($O\underline{C}H_2CH_2$), 59.32 ($O\underline{C}H_2CC$), 34.02, 34.01, 33.58 ($Ar\underline{C}(CH_3)_3$), 32.22 ($ArCH_2Ar$), 31.71, 31.65, 31.19 ($ArC(\underline{C}H_3)_3$), 30.96 ($ArCH_2Ar$), 25.98 ($SiC(\underline{C}H_3)_3$), 23.62, 23.48 ($\underline{C}H_2CH_3$), 16.40 ($Si\underline{C}(CH_3)_3$), 10.74, 10.06 ($CH_2\underline{C}H_3$), –4.73 ($Si(CH_3)_2$) ppm. ESI-MS *m*/*z*: 927.6678 $[M+H]^+$ for $C_{62}H_{91}O_4Si$ (927.6681).

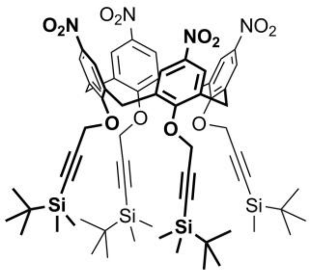

Exhaustively nitrated silylated tetrapropargyl ether ***11***.[S8] To a cooled (0–5 °C) solution of calixarene **6** (3.10 g, 2.47 mmol) in dry dichloromethane (100 mL) glacial acetic acid (5.68 mL, 99.84 mmol) was added under stirring followed by fuming nitric acid (4.16 mL, 99.84 mmol). The mixture was stirred at room temperature for 72 h, water (50 mL) was added, and the reaction products were extracted with dichloromethane. The combined organic fractions were washed with water, dried, and the solvent was evaporated. The residue was recrystallized twice from methanol. Yield 0.93 g (31%), yellowish crystals. ^{1}H NMR spectrum of the obtained compound was identical to the previously published one.[S8]

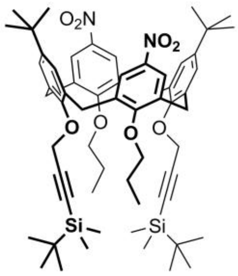

Dinitrated silylated dipropargyl ether ***12*** was prepared as described for compound **11** from calixarene **8** (0.048 g, 0.046 mmol), fuming nitric acid (0.02 mL, 0.46 mmol) and glacial acetic acid (0.03 mL, 0.46 mmol) in dry dichloromethane (2.3 ml). The reaction time was shortened to 12 h, and a single recrystallization was enough to purify the substance. Yield 0.032 g (69%), white crystals. M.p. 190–192 °C. ^{1}H NMR (600 MHz, $CDCl_3$): δ = 7.18 (s, 4H; ArH), 7.04 (s, 4H; ArH), 4.88 (s, 4H; OCH_2CC), 4.53 (d, 4H, ^2J = 13.6 Hz; $ArCH_2Ar$), 3.75–3.70 (m, 4H; $OC\underline{H}_2CH_2$), 3.18 (d, 4H, ^2J = 13.6 Hz; $ArCH_2Ar$), 2.00–1.93 (m, 4H; $OCH_2C\underline{H}_2$), 1.39 (s, 18H; $ArC(CH_3)_3$), 1.11 (t, 6H, ^3J = 7.4 Hz; $CH_2C\underline{H}_3$), 0.83 (s, 18H; $SiC(CH_3)_3$), 0.02 (s, 12H; $Si(CH_3)_2$) ppm. ^{13}C NMR (150 MHz, $CDCl_3$): δ = 160.40, 151.51, 147.11, 142.77, 136.02, 135.10 (C_{Ar}), 126.03, 122.81 (CH_{Ar}), 102.34 ($OCH_2\underline{C}C$), 90.73 ($OCH_2C\underline{C}$), 77.43 ($O\underline{C}H_2CH_2$), 60.05 ($O\underline{C}H_2CC$), 34.33 ($Ar\underline{C}(CH_3)_3$), 31.78 ($ArCH_2Ar$), 31.59 ($ArC(\underline{C}H_3)_3$), 25.91 ($SiC(\underline{C}H_3)_3$), 23.57 ($\underline{C}H_2CH_3$), 16.36 ($Si\underline{C}(CH_3)_3$), 10.73 ($CH_2\underline{C}H_3$), –4.81 ($Si(CH_3)_2$) ppm. ESI-MS *m*/*z*: 1015.5682 $[M+H]^+$ for $C_{60}H_{83}N_2O_8Si_2$ (1015.5682).

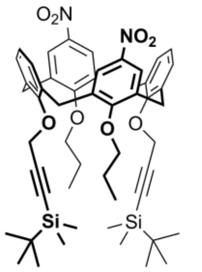

Dinitrated silylated dipropargyl ether ***13*** was prepared as described for compound **12** from calixarene **9** (6.39 g, 7.87 mmol), fuming nitric acid (3.28 mL, 78.74 mmol) and glacial acetic acid (4.50 mL, 78.74 mmol) in dry dichloromethane (394 mL). Yield 3.20 g (45%), yellow solid. M.p. 141–143 °C. ^{1}H NMR (600 MHz, CDCl$_3$): δ = 7.18 (d, 4H, ^{3}J = 7.5 Hz; ArH), 7.06 (s, 4H; ArH), 7.05 (t, 2H, ^{3}J = 7.5 Hz; ArH), 4.92 (s, 4H; OCH$_2$CC), 4.54 (d, 4H, ^{2}J = 13.7 Hz; ArCH$_2$Ar), 3.76–3.72 (m, 4H; OC$\underline{H}_2$CH$_2$), 3.21 (d, 4H, ^{2}J = 13.7 Hz; ArCH$_2$Ar), 2.01–1.93 (m, 4H; OCH$_2$C$\underline{H}_2$), 1.11 (t, 6H, ^{3}J = 7.4 Hz; CH$_2$C$\underline{H}_3$), 0.83 (s, 18H; C(CH$_3$)$_3$), 0.02 (s, 12H; Si(CH$_3$)$_2$) ppm. ^{13}C NMR (150 MHz, CDCl$_3$): δ = 160.51, 153.99, 142.86, 136.84, 135.14 (C$_{Ar}$), 129.27, 124.49, 122.91 (CH$_{Ar}$), 101.94 (OCH$_2\underline{C}$C), 91.19 (OCH$_2$C$\underline{C}$), 77.52 (O$\underline{C}$H$_2$CH$_2$), 60.24 (O$\underline{C}$H$_2$CC), 31.47 (ArCH$_2$Ar), 25.88 (C($\underline{C}$H$_3$)$_3$), 23.56 ($\underline{C}$H$_2$CH$_3$), 16.32 ($\underline{C}$(CH$_3$)$_3$), 10.67 (CH$_2\underline{C}$H$_3$), –4.83 (Si(CH$_3$)$_2$) ppm. ESI-MS *m/z*: 903.4439 [M+H]$^+$ for $C_{52}H_{67}N_2O_8Si_2$ (903.4430).

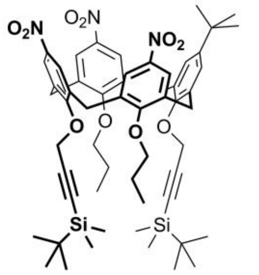

Trinitrated silylated dipropargyl ether ***14*** was prepared as described for compound **12** from calixarene **8** (3.00 g, 2.90 mmol), fuming nitric acid (1.20 mL, 28.96 mmol) and glacial acetic acid (1.65 mL, 28.96 mmol) in dry dichloromethane (30 mL). Yield 2.16 g (73%), white solid. M.p. 260–262 °C. ^{1}H NMR (400 MHz, CDCl$_3$): δ = 8.15 (s, 2H; ArH), 7.20 (s, 2H; ArH), 7.14 (d, 2H, ^{4}J = 2.7 Hz; ArH), 7.06 (d, 2H, ^{4}J = 2.7 Hz; ArH), 5.11 (s, 2H; OCH$_2$CC), 4.84 (s, 2H; OCH$_2$CC), 4.56 (d, 2H, ^{2}J = 14.0 Hz; ArCH$_2$Ar), 4.54 (d, 2H, ^{2}J = 13.5 Hz; ArCH$_2$Ar), 3.82–3.68 (m, 4H; OC$\underline{H}_2$CH$_2$), 3.36 (d, 2H, ^{2}J = 14.0 Hz; ArCH$_2$Ar), 3.23 (d, 2H, ^{2}J = 13.5 Hz; ArCH$_2$Ar), 2.04–1.92 (m, 4H, OCH$_2$C$\underline{H}_2$), 1.39 (s, 9H; ArC(CH$_3$)$_3$), 1.12 (t, 6H, ^{3}J = 7.4 Hz; CH$_2$C$\underline{H}_3$), 0.84 (s, 9H; SiC(CH$_3$)$_3$), 0.78 (s, 9H; SiC(CH$_3$)$_3$), 0.04 (s, 6H; Si(CH$_3$)$_2$), –0.02 (s, 6H; Si(CH$_3$)$_2$) ppm. ^{13}C NMR (100 MHz, CDCl$_3$): δ = 160.40, 159.92, 151.48, 147.66, 143.54, 142.89, 138.64, 135.59, 135.41, 133.51 (C$_{Ar}$), 126.22, 124.30, 123.70, 122.35 (CH$_{Ar}$), 101.72, 100.20 (OCH$_2\underline{C}$C), 93.07, 91.24 (OCH$_2$C$\underline{C}$), 77.77 (bs, O$\underline{C}$H$_2$CH$_2$), 60.58, 60.55 (O$\underline{C}$H$_2$CC), 34.35 (Ar$\underline{C}$(CH$_3$)$_3$), 31.65 (ArCH$_2$Ar), 31.52 (ArC($\underline{C}$H$_3$)$_3$), 31.48 (ArCH$_2$Ar), 25.89, 25.69 (SiC($\underline{C}$H$_3$)$_3$), 23.55 ($\underline{C}$H$_2$CH$_3$), 16.36, 16.19 (Si$\underline{C}$(CH$_3$)$_3$), 10.67 (CH$_2\underline{C}$H$_3$), –4.83, –4.97 (Si(CH$_3$)$_2$) ppm. ESI-MS *m/z*: 1004.4906 [M+H]$^+$ for $C_{56}H_{74}N_3O_{10}Si_2$ (1004.4907).

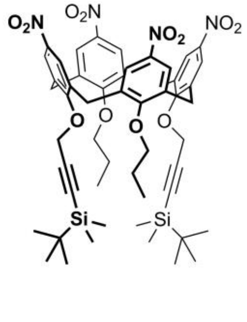

Exhaustively nitrated silylated dipropargyl ether ***15*** was prepared as described for compound **11** from calixarene **8** (3.12 g, 3.01 mmol), fuming nitric acid (5.0 mL, 120.4 mmol) and glacial acetic acid (6.9 mL, 120.4 mmol) in dry dichloromethane (48 mL). The reaction time was

shortened to 48 h, and a single recrystallization was enough to purify the substance. Yield 1.97 g (65%), white crystals. M.p. 254–256 °C. ^{1}H NMR (400 MHz, $CDCl_3$): δ = 8.17 (s, 4H; ArH), 7.14 (s, 4H; ArH), 5.08 (s, 4H; OCH_2CC), 4.58 (d, 4H, ^{2}J = 13.8 Hz; $ArCH_2Ar$), 3.82–3.73 (m, 4H; $OC\underline{H}_2CH_2$), 3.41 (d, 4H, ^{2}J = 13.8 Hz; $ArCH_2Ar$), 2.06–1.93 (m, 4H; $OCH_2C\underline{H}_2$), 1.12 (t, 6H, ^{3}J = 7.4 Hz; $CH_2C\underline{H}_3$), 0.80 (s, 18H; $SiC(CH_3)_3$), 0.00 (s, 12H; $Si(CH_3)_2$) ppm. ^{13}C NMR (100 MHz, $CDCl_3$): δ = 160.44, 159.68, 143.83, 143.05, 138.29, 133.77 (C_{Ar}), 124.44, 123.38 (CH_{Ar}), 99.64 ($OCH_2\underline{C}C$), 93.64 ($OCH_2C\underline{C}$), 78.17 ($O\underline{C}H_2CH_2$), 60.91 ($O\underline{C}H_2CC$), 31.43 ($ArCH_2Ar$), 25.70 ($SiC(\underline{C}H_3)_3$), 23.57 ($\underline{C}H_2CH_3$), 16.21 ($Si\underline{C}(CH_3)_3$), 10.58 ($CH_2\underline{C}H_3$), –4.97 ($Si(CH_3)_2$) ppm. ESI-MS *m/z*: 993.4132 $[M+H]^+$ for $C_{52}H_{65}N_4O_{12}Si_2$ (993.4132).

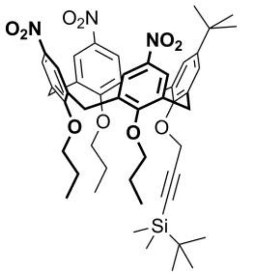

Trinitrated silylated propargyl ether ***16*** was prepared as described for compound **15** from calixarene **10** (3.74 g, 4.04 mmol), fuming nitric acid (1.73 mL, 40.4 mmol) and glacial acetic acid (2.3 mL, 40.4 mmol) in dry dichloromethane (40 mL). Yield 2.76 g (77%), white crystals. M.p. 172–174 °C. ^{1}H NMR (400 MHz, $CDCl_3$): δ = 8.04 (s, 2H; ArH), 7.16 (d, 2H, ^{4}J = 2.7 Hz; ArH), 7.09 (d, 2H, ^{4}J = 2.7 Hz; ArH), 7.07 (s, 2H; ArH), 4.78 (s, 2H; $OC\underline{H}_2CC$), 4.52 (d, 2H, ^{2}J = 13.8 Hz; $ArCH_2Ar$), 4.51 (d, 2H, ^{2}J = 13.7 Hz; $ArCH_2Ar$), 4.14–4.05 (m, 2H; $OC\underline{H}_2CH_2$), 3.86–3.73 (m, 4H; $OC\underline{H}_2CH_2$), 3.35 (d, 2H, ^{2}J = 13.8 Hz; $ArCH_2Ar$), 3.23 (d, 2H, ^{2}J = 13.7 Hz; $ArCH_2Ar$), 2.00–1.83 (m, 6H; $OCH_2C\underline{H}_2$), 1.30 (s, 9H; $ArC(CH_3)_3$), 1.08 (t, 6H, ^{3}J = 7.4 Hz; $CH_2C\underline{H}_3$), 0.94 (t, 3H, ^{3}J = 7.4 Hz; $CH_2C\underline{H}_3$), 0.83 (s, 9H; $SiC(CH_3)_3$), 0.03 (s, 6H; $Si(CH_3)_2$) ppm. ^{13}C NMR (100 MHz, $CDCl_3$): δ = 162.87, 160.65, 151.40, 147.18, 142.72, 142.65, 136.51, 136.11, 135.36, 133.47 (C_{Ar}), 126.05, 124.64, 123.61, 122.40 (CH_{Ar}), 101.83 ($OCH_2\underline{C}C$), 90.92 ($OCH_2C\underline{C}$), 77.60 (bs, $O\underline{C}H_2CH_2$), 60.14 ($O\underline{C}H_2CC$), 34.20 ($Ar\underline{C}(CH_3)_3$), 31.78 ($ArCH_2Ar$), 31.43 ($ArC(\underline{C}H_3)_3$), 30.94 ($ArCH_2Ar$), 25.88 ($SiC(\underline{C}H_3)_3$), 23.38, 23.13 ($\underline{C}H_2CH_3$), 16.34 ($Si\underline{C}(CH_3)_3$), 10.55, 9.88 ($CH_2\underline{C}H_3$), –4.84 ($Si(CH_3)_2$) ppm. ESI-MS *m/z*: 894.4355 $[M+H]^+$ for $C_{50}H_{64}N_3O_{10}Si$ (894.4355).

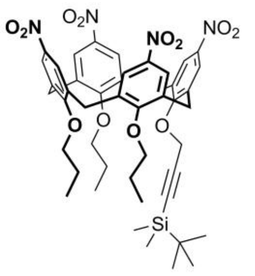

Exhaustively nitrated silylated propargyl ether ***17*** was prepared as described for compound **15** from calixarene **10** (4.71 g, 5.09 mmol), fuming nitric acid (8.5 mL, 204.1 mmol) and glacial acetic acid (11.7 mL, 203.7 mmol) in dry dichloromethane (82 mL). Yield 3.21 g (74%), yellow crystals. M.p. 160–162 °C. ^{1}H NMR (400 MHz, $CDCl_3$): δ = 8.07 (bs, 2H; ArH), 8.06 (bs, 2H; ArH), 7.15 (s, 4H; ArH), 5.00 (s, 2H; $OC\underline{H}_2CC$), 4.55 (d, 2H, ^{2}J = 14.2 Hz; $ArCH_2Ar$), 4.53 (d, 2H, ^{2}J = 13.8 Hz; $ArCH_2Ar$), 4.16–4.07 (m, 2H; $OC\underline{H}_2CH_2$), 3.85–3.75 (m, 4H; $OC\underline{H}_2CH_2$), 3.41 (d, 2H, ^{2}J = 14.2 Hz; $ArCH_2Ar$), 3.40 (d, 2H, ^{2}J = 13.8 Hz; $ArCH_2Ar$), 2.00–1.84 (m, 6H; $OCH_2C\underline{H}_2$), 1.09 (t, 6H, ^{3}J = 7.4 Hz; $CH_2C\underline{H}_3$), 0.96 (t, 3H, ^{3}J = 7.4 Hz; $CH_2C\underline{H}_3$), 0.79 (s, 9H; $C(CH_3)_3$), 0.01 (s, 6H; $Si(CH_3)_2$) ppm. ^{13}C NMR

(100 MHz, $CDCl_3$): δ = 162.65, 160.66, 159.65, 143.59, 142.92 (bs), 138.18, 136.45, 134.45, 133.90 (C_{Ar}), 124.70, 124.32, 123.30, 123.18 (CH_{Ar}), 99.75 ($OCH_2\underline{C}C$), 93.29 ($OCH_2C\underline{C}$), 78.01, 77.86 ($O\underline{C}H_2CH_2$), 60.61 ($O\underline{C}H_2CC$), 31.48, 30.91 ($ArCH_2Ar$), 25.68 ($C(\underline{C}H_3)_3$), 23.38, 23.18 ($\underline{C}H_2CH_3$), 16.21 ($\underline{C}(CH_3)_3$), 10.48, 9.86 ($CH_2\underline{C}H_3$), –4.97 ($Si(CH_3)_2$) ppm. ESI-MS *m*/*z*: 883.3572 $[M+H]^+$ for $C_{46}H_{55}N_4O_{12}Si$ (883.3580).

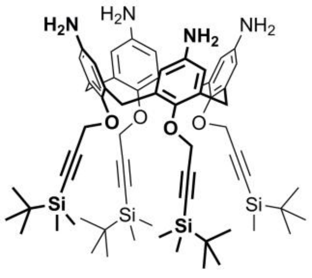

Silylated tetrapropargyl ether of p-aminocalix[4]arene ***18***.[S8] A suspension of $SnCl_2{\cdot}2H_2O$ (8.00 g, 35.39 mmol) in a mixture of ethanol (50 mL) and 10 M HCl (3.5 mL) was added to a stirred suspension of calixarene **11** (1.04 g, 0.858 mmol) in ethanol (50 mL) at 70 °C. The mixture was stirred in a septum-sealed flask at 70 °C for 6 h. The resulting homogeneous solution was cooled to room temperature, ethanol and most of the water were evaporated under reduced pressure, and 2 M aqueous KOH was added to the residue in portions until a pH >7 was achieved. The reaction products were extracted with diethyl ether. The organic fractions were washed with water, dried over anhydrous Na_2SO_4, and the solvent was evaporated under reduced pressure. Yield 0.89 g (95%), yellowish solid. ^{1}H NMR spectrum of the obtained compound was identical to the previously published one.[S8]

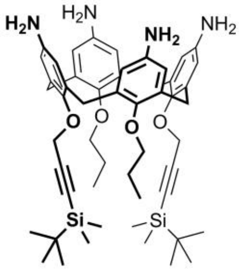

Silylated dipropargyl ether of p-aminocalix[4]arene ***19*** was prepared as described for compound **18** from calixarene **15** (1.97 g, 1.98 mmol), $SnCl_2{\cdot}2H_2O$ (18.0 g, 79.2 mmol) and 10 M HCl (8 mL) in ethanol (200 mL). Yield 1.43 g (82%), yellowish solid. M.p. 182–184 °C (decomp.). ^{1}H NMR (400 MHz, $CDCl_3$): δ = 6.42 (s, 4H; ArH), 5.77 (s, 4H; ArH), 4.87 (s, 4H; OCH_2CC), 4.38 (d, 4H, ^2J = 13.1 Hz; $ArCH_2Ar$), 3.62–3.52 (m, 4H; $OC\underline{H}_2CH_2$), 3.32 (bs, 8H; NH_2), 2.90 (d, 4H, ^2J = 13.1 Hz; $ArCH_2Ar$), 1.99–1.85 (m, 4H; $OCH_2C\underline{H}_2$), 1.03 (t, 6H, ^3J = 7.4 Hz; $CH_2C\underline{H}_3$), 0.84 (s, 18H; $C(CH_3)_3$), 0.02 (s, 12H; $Si(CH_3)_2$) ppm. ^{13}C NMR (100 MHz, $CDCl_3$): δ = 148.89, 147.47, 141.10, 140.52, 138.34, 133.55 (C_{Ar}), 115.38, 115.18 (CH_{Ar}), 104.00 ($OCH_2\underline{C}C$), 89.35 ($OCH_2C\underline{C}$), 77.17 ($O\underline{C}H_2CH_2$), 59.86 ($O\underline{C}H_2CC$), 31.73 ($ArCH_2Ar$), 25.92 ($C(\underline{C}H_3)_3$), 23.60 ($\underline{C}H_2CH_3$), 16.36 ($\underline{C}(CH_3)_3$), 10.76 ($CH_2\underline{C}H_3$), –4.76 ($Si(CH_3)_2$) ppm. ESI-MS *m*/*z*: 873.5146 $[M+H]^+$ for $C_{52}H_{73}N_4O_4Si_2$ (873.5165).

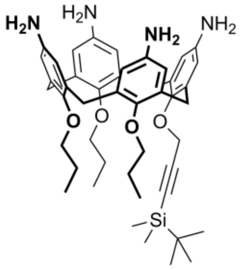

Silylated propargyl ether of p-aminocalix[4]arene ***20*** was prepared as described for compound **18** from calixarene **17** (3.21 g, 3.64 mmol), $SnCl_2{\cdot}2H_2O$ (32.9 g, 145.5 mmol) and 10 M HCl (14.5 mL) in ethanol (300 mL). The precipitate formed upon addition of KOH was collected,

washed repeatedly with water and then parted between water and dichloromethane. The organic fraction was separated, washed with water, dried, and the solvent was evaporated. Yield 2.07 g (75%), yellow solid. M.p. 178–180 °C (decomp.). ^{1}H NMR (400 MHz, $CDCl_3$): δ = 6.36 (s, 2H; ArH), 6.32 (s, 2H; ArH), 5.79 (d, 2H, ^4J = 2.5 Hz; ArH), 5.78 (d, 2H, ^4J = 2.5 Hz; ArH), 4.80 (s, 2H; $OC\underline{H}_2CC$), 4.35 (d, 2H, ^2J = 13.3 Hz; $ArCH_2Ar$), 4.31 (d, 2H, ^2J = 13.0 Hz; $ArCH_2Ar$), 3.88–3.80 (m, 2H; $OC\underline{H}_2CH_2$), 3.65–3.55 (m, 4H; $OC\underline{H}_2CH_2$), 3.18 (bs, 8H; NH_2), 2.91 (d, 4H, ^2J = 13.3 Hz; $ArCH_2Ar$), 1.96–1.76 (m, 6H; $OCH_2C\underline{H}_2$), 1.01 (t, 6H, ^3J = 7.4 Hz; $CH_2C\underline{H}_3$), 0.89 (t, 3H, ^3J = 7.3 Hz; $CH_2C\underline{H}_3$), 0.84 (s, 9H; $C(CH_3)_3$), 0.03 (s, 6H; $Si(CH_3)_2$) ppm. ^{13}C NMR (100 MHz, $CDCl_3$): δ = 150.56, 149.17, 147.66, 140.90, 140.42, 140.27, 138.04, 136.92, 134.34, 133.80 (C_{Ar}), 115.79, 115.44, 115.43, 115.21 (CH_{Ar}), 104.06 ($OCH_2\underline{C}C$), 89.08 ($OCH_2C\underline{C}$), 76.97, 76.75 ($O\underline{C}H_2CH_2$), 59.70 ($O\underline{C}H_2CC$), 31.71, 30.96 ($ArCH_2Ar$), 25.91 ($C(\underline{C}H_3)_3$), 23.38, 22.99 ($\underline{C}H_2CH_3$), 16.37 ($\underline{C}(CH_3)_3$), 10.71, 10.09 ($CH_2\underline{C}H_3$), –4.76 ($Si(CH_3)_2$) ppm. ESI-MS *m*/*z*: 763.4615 $[M+H]^+$ for $C_{46}H_{63}N_4O_4Si$ (763.4613).

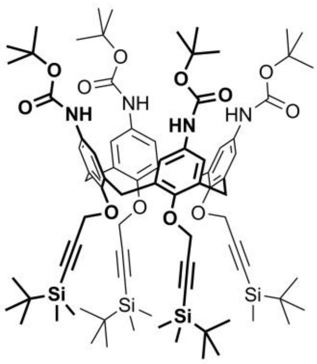

Silylated tetrapropargyl ether of Boc-protected p-aminocalix[4]arene ***21***. Di-*tert*-butyl dicarbonate (Boc_2O, 0.89 g, 4.01 mmol) was added to a stirred solution of calixarene **18** (0.89 g, 0.82 mmol) in dry dichloromethane (25 mL). The stirring was continued at room temperature for 24 h, the solvent was evaporated under reduced pressure and the residue was purified by column chromatography (silica, gradient from dichloromethane to dichloromethane/ethanol 200:1). Yield 0.96 g (79%), white solid. M.p. 242–244 °C. ^{1}H NMR (400 MHz, $CDCl_3$): δ = 6.73 (s, 8H; ArH), 6.15 (s, 4H; NH), 4.74 (s, 8H; OCH_2), 4.50 (d, 4H, ^2J = 13.1 Hz; $ArCH_2Ar$), 3.10 (d, 4H, ^2J = 13.1 Hz; $ArCH_2Ar$), 1.46 (s, 36H; $OC(CH_3)_3$), 0.88 (s, 36H; $SiC(CH_3)_3$), 0.06 (s, 24H; $Si(CH_3)_2$) ppm. ^{13}C NMR (100 MHz, $CDCl_3$): δ = 153.00 (C=O), 150.76, 135.57, 132.94 (C_{Ar}), 119.29 (CH_{Ar}), 102.80 ($OCH_2\underline{C}C$), 89.84 ($OCH_2C\underline{C}$), 79.88 ($O\underline{C}(CH_3)_3$), 61.63 ($O\underline{C}H_2CC$), 32.26 ($ArCH_2Ar$), 28.36 ($OC(\underline{C}H_3)_3$), 26.03 ($SiC(\underline{C}H_3)_3$), 16.37 ($Si\underline{C}(CH_3)_3$), –4.68 ($Si(CH_3)_2$) ppm. ESI-MS *m*/*z*: 1493.8368 $[M+H]^+$ for $C_{84}H_{125}N_4O_{12}Si_4$ (1493.8366).

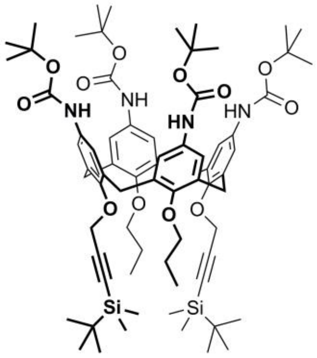

Silylated dipropargyl ether of Boc-protected p-aminocalix[4]arene ***22*** was prepared as described for compound **21** from calixarene **19** (1.43 g, 1.64 mmol) and Boc_2O (1.79 g, 8.20 mmol) in dry dichloromethane (40 mL). Instead of a chromatographic purification, the residue obtained after the solvent removal was dissolved in a minimum amount of dichloromethane, hexane was added, the solid formed was collected, washed with hexane and dried. Yield 1.55 g

(75%), white solid. M.p. 252–254 °C. ^{1}H NMR (400 MHz, $CDCl_3$): δ = 7.10 (s, 4H; ArH), 6.41 (s, 2H; NH), 6.28 (s, 4H; ArH), 5.90 (s, 2H; NH), 4.94 (s, 4H; OCH_2CC), 4.43 (d, 4H, ^{2}J = 13.1 Hz; $ArCH_2Ar$), 3.64–3.54 (m, 4H; $OC\underline{H}_2CH_2$), 3.08 (d, 4H, ^{2}J = 13.1 Hz; $ArCH_2Ar$), 1.99–1.86 (m, 4H; $OCH_2C\underline{H}_2$), 1.53 (s, 18H; $OC(CH_3)_3$), 1.39 (s, 18H; $OC(CH_3)_3$), 1.04 (t, 6H, ^{3}J = 7.4 Hz; $CH_2C\underline{H}_3$), 0.82 (s, 18H; $SiC(CH_3)_3$), 0.00 (s, 12H; $Si(CH_3)_2$) ppm. ^{13}C NMR (100 MHz, $CDCl_3$): δ = 153.35, 152.80 (C=O), 152.02, 150.15, 137.85, 133.32, 133.28, 131.79 (C_{Ar}), 120.29, 118.41 (CH_{Ar}), 103.14 ($OCH_2\underline{C}C$), 90.16 ($OCH_2C\underline{C}$), 80.03, 79.62 ($O\underline{C}(CH_3)_3$), 77.27 ($O\underline{C}H_2CH_2$), 59.96 ($O\underline{C}H_2CC$), 31.69 ($ArCH_2Ar$), 28.41, 28.34 ($OC(\underline{C}H_3)_3$), 25.90 ($SiC(\underline{C}H_3)_3$), 23.58 ($\underline{C}H_2CH_3$), 16.33 ($Si\underline{C}(CH_3)_3$), 10.69 ($CH_2\underline{C}H_3$), –4.79 ($Si(CH_3)_2$) ppm. ESI-MS *m*/*z*: 1273.7268 $[M+H]^+$ for $C_{72}H_{105}N_4O_{12}Si_2$ (1273.7262).

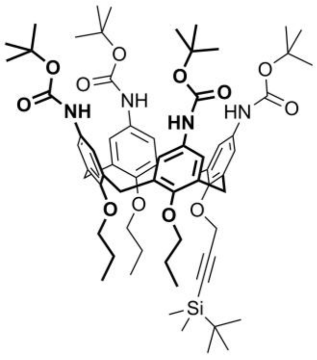

Silylated propargyl ether of Boc-protected p-aminocalix[4]arene ***23*** was prepared as described for compound **21** from calixarene **20** (0.66 g, 0.87 mmol) and Boc_2O (0.95 g, 4.35 mmol) in dry dichloromethane. Yield 0.71 g (71%), white solid. M.p. 178–180 °C. ^{1}H NMR (400 MHz, $CDCl_3$): δ = 7.01 (s, 2H; ArH), 6.99 (s, 2H; ArH), 6.39 (s, 1H; NH), 6.34 (s, 1H; NH), 6.29 (s, 4H; ArH), 5.94 (s, 2H; NH), 4.89 (s, 2H; OCH_2CC), 4.40 (d, 2H, ^{2}J = 13.2 Hz; $ArCH_2Ar$), 4.36 (d, 2H, ^{2}J = 13.2 Hz; $ArCH_2Ar$), 3.94–3.84 (m, 2H; $OC\underline{H}_2CH_2$), 3.67–3.56 (m, 4H; $OC\underline{H}_2CH_2$), 3.08 (d, 4H, ^{2}J = 13.2 Hz; $ArCH_2Ar$), 1.95–1.79 (m, 6H; $OCH_2C\underline{H}_2$), 1.52 (s, 9H; $OC(CH_3)_3$), 1.51 (s, 9H; $OC(CH_3)_3$), 1.42 (s, 18H; $OC(CH_3)_3$), 1.02 (t, 6H, ^{3}J = 7.4 Hz; $CH_2C\underline{H}_3$), 0.88 (t, 3H, ^{3}J = 7.4 Hz; $CH_2C\underline{H}_3$), 0.81 (s, 9H; $SiC(CH_3)_3$), 0.01 (s, 6H; $Si(CH_3)_2$) ppm. ^{13}C NMR (100 MHz, $CDCl_3$): δ = 153.43, 153.15, 153.06, 152.91 (C=O, C_{Ar}), 152.23, 150.35, 137.65, 136.43, 134.00, 133.49, 133.00, 132.31, 131.77 (C_{Ar}), 120.26, 120.09, 119.23, 118.70 (CH_{Ar}), 103.24 ($OCH_2\underline{C}C$), 89.90 ($OCH_2C\underline{C}$), 80.03 (bs), 79.72 ($O\underline{C}(CH_3)_3$), 77.09, 76.77 ($O\underline{C}H_2CH_2$), 59.71 ($O\underline{C}H_2CC$), 31.70, 30.90 ($ArCH_2Ar$), 28.40, 28.37 ($OC(\underline{C}H_3)_3$), 25.88 ($SiC(\underline{C}H_3)_3$), 23.36, 22.96 ($\underline{C}H_2CH_3$), 16.33 ($Si\underline{C}(CH_3)_3$), 10.65, 10.03 ($CH_2\underline{C}H_3$), –4.79 ($Si(CH_3)_2$) ppm. ESI-MS *m*/*z*: 1163.6704 $[M+H]^+$ for $C_{66}H_{95}N_4O_{12}Si$ (1163.6710).

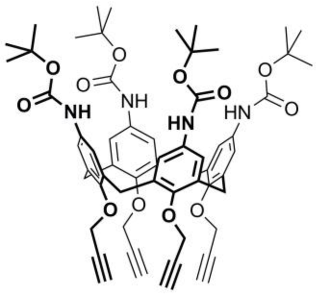

Tetrapropargyl ether of Boc-protected p-aminocalix[4]arene ***24***. To a stirred solution of calixarene **21** (0.552 g, 0.37 mmol) in THF (7.5 mL), tetra(*n*-butyl)ammonium fluoride trihydrate (0.047 g, 0.149 mmol) and water (0.074 mL) were added. The reaction mixture was stirred at room temperature for 72 h, and the solvents were evaporated. The residue was dissolved in a minimum amount of

dichloromethane, hexane was added, and the solid formed was collected, washed with hexane and dried. Yield 0.353 g (92%), white solid. M.p. 165–167 °C. ^{1}H NMR (400 MHz, $CDCl_3$): δ = 6.74 (s, 8H; ArH), 6.18 (s, 4H; NH), 4.70 (d, 8H, ^4J = 2.4 Hz; $C\underline{H}_2CCH$), 4.55 (d, 4H, ^2J = 13.3 Hz; $ArCH_2Ar$), 3.15 (d, 4H, ^2J = 13.3 Hz; $ArCH_2Ar$), 2.43 (t, 4H, ^4J = 2.4 Hz; $CH_2CC\underline{H}$), 1.46 (s, 36H; $OC(CH_3)_3$) ppm. ^{13}C NMR (100 MHz, $CDCl_3$): δ = 153.06 (C=O), 150.91, 135.43, 133.15 (C_{Ar}), 119.44 (CH_{Ar}), 80.38 ($OCH_2\underline{C}C$), 80.00 ($O\underline{C}(CH_3)_3$), 74.80 (CH), 61.17 ($O\underline{C}H_2C$), 32.09 ($ArCH_2Ar$), 28.34 ($C(\underline{C}H_3)_3$) ppm. ESI-MS *m/z*: 1059.4723 $[M+Na]^+$ for $C_{60}H_{68}NaN_4O_{12}$ (1059.4726).

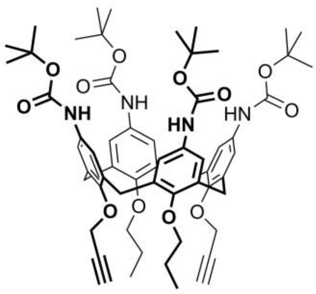

Dipropargyl ether of Boc-protected p-aminocalix[4]arene **25** was prepared as described for compound **24** from calixarene **22** (4.08 g, 3.21 mmol), tetra(*n*-butyl)ammonium fluoride trihydrate (0.20 g, 0.64 mmol) and water (0.35 mL) in THF (36.5 mL). The reacton was conducted at 50 °C for 24 h. The product was purified by column chromatography (silica, gradient from dichloromethane to dichloromethane/ethanol 100:1). Yield 3.25 g (97%), white solid. M.p. 160–162 °C. ^{1}H NMR (400 MHz, $CDCl_3$): δ = 7.17 (s, 4H; ArH), 6.45 (s, 2H; NH), 6.24 (s, 4H; ArH), 5.89 (s, 2H; NH), 4.92 (d, 4H, ^4J = 2.4 Hz; $OC\underline{H}_2CCH$), 4.48 (d, 4H, ^2J = 13.4 Hz; $ArCH_2Ar$), 3.67–3.58 (m, 4H; $OC\underline{H}_2CH_2$), 3.14 (d, 4H, ^2J = 13.4 Hz; $ArCH_2Ar$), 2.36 (t, 2H, ^4J = 2.4 Hz; $CH_2CC\underline{H}$), 1.96–1.83 (m, 4H; $OCH_2C\underline{H}_2$), 1.55 (s, 18H; $C(CH_3)_3$), 1.42 (s, 18H; $C(CH_3)_3$), 1.07 (t, 6H, ^3J = 7.4 Hz; $CH_2C\underline{H}_3$) ppm. ^{13}C NMR (100 MHz, $CDCl_3$): δ = 153.43, 152.88 (C=O), 152.09, 150.73, 137.71, 133.40, 133.20, 131.84 (C_{Ar}), 120.22, 118.69 (CH_{Ar}), 80.66 ($OCH_2\underline{C}CH$), 80.14, 79.69 ($O\underline{C}(CH_3)_3$), 77.22 ($O\underline{C}H_2CH_2$), 74.81 (CH), 59.78 ($O\underline{C}H_2CCH$), 31.44 ($ArCH_2Ar$), 28.37, 28.34 ($C(\underline{C}H_3)_3$), 23.45 ($\underline{C}H_2CH_3$), 10.80 ($CH_2\underline{C}H_3$) ppm. ESI-MS *m/z*: 1062.5806 $[M+NH_4]^+$ for $C_{60}H_{80}N_5O_{12}$ (1062.5798).

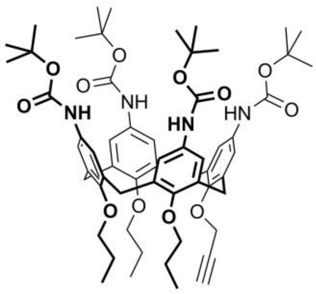

Propargyl ether of Boc-protected p-aminocalix[4]arene **26** was prepared as described for compound **25** from calixarene **23** (1.80 g, 1.55 mmol), tetra(*n*-butyl)ammonium fluoride trihydrate (0.098 g, 0.311 mmol) and water (0.177 mL) in THF (17.5 ml). Yield 0.636 g (39%), white solid. M.p. 166–168 °C. ^{1}H NMR (400 MHz, $CDCl_3$): δ = 7.05 (s, 2H; ArH), 7.03 (s, 2H; ArH), 6.36 (bs, 2H; NH), 6.26 (d, 2H, ^4J = 2.4 Hz; ArH), 6.23 (d, 2H, ^4J = 2.4 Hz; ArH), 5.88 (s, 2H; NH), 4.85 (d, 2H, ^4J = 2.4 Hz; $OC\underline{H}_2CCH$), 4.44 (d, 2H, ^2J = 13.5 Hz; $ArCH_2Ar$), 4.37 (d, 2H, ^2J = 13.1 Hz; $ArCH_2Ar$), 3.96–3.87 (m, 2H; $OC\underline{H}_2CH_2$), 3.67–3.57 (m, 4H; $OC\underline{H}_2CH_2$), 3.11 (d, 2H,

^2J = 13.5 Hz; $ArCH_2Ar$), 3.08 (d, 2H, ^2J = 13.1 Hz; $ArCH_2Ar$), 2.30 (t, 1H, ^4J = 2.4 Hz; $CH_2CC\underline{H}$), 1.96–1.78 (m, 6H; $OCH_2C\underline{H}_2$), 1.52 (s, 9H; $C(CH_3)_3$), 1.51 (s, 9H; $C(CH_3)_3$), 1.42 (s, 18H; $C(CH_3)_3$), 1.02 (t, 6H, ^3J = 7.4 Hz; $CH_2C\underline{H}_3$), 0.89 (t, 3H, ^3J = 7.5 Hz; $CH_2C\underline{H}_3$) ppm. ^{13}C NMR (100 MHz, $CDCl_3$): δ = 153.47 (C_{Ar}), 153.23, 153.04, 152.93 (C=O), 152.20, 150.63, 137.58, 136.48, 133.76, 133.48, 133.15, 132.29, 131.78 (C_{Ar}), 120.20, 120.16, 119.22, 118.83 (CH_{Ar}), 80.78 ($OCH_2\underline{C}CH$), 80.09, 80.06, 79.73 ($O\underline{C}(CH_3)_3$), 77.08, 76.79 ($O\underline{C}H_2CH_2$), 74.51 (CH), 59.35 ($O\underline{C}H_2CCH$), 31.53, 30.88 ($ArCH_2Ar$), 28.38, 28.37, 28.36 ($C(\underline{C}H_3)_3$), 23.33, 22.97 ($\underline{C}H_2CH_3$), 10.67, 9.99 ($CH_2\underline{C}H_3$) ppm. ESI-MS *m*/*z*: 1049.5844 $[M+H]^+$ for $C_{60}H_{81}N_4O_{12}$ (1049.5846).

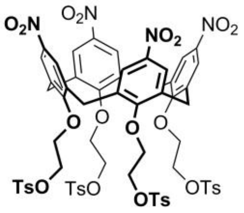

Exhaustively nitrated calix[4]arene tetratosylate ***29*** was prepared as described for calixarene **15** from calixarene **27** (1.22 g, 0.85 mmol), fuming nitric acid (1.41 mL, 34.0 mmol) and glacial acetic acid (1.94 mL, 34.0 mmol) in dry dichloromethane (13.5 mL). Yield 0.90 g (76%), yellow crystals. M.p. 273–275 °C. ^{1}H NMR (400 MHz, $CDCl_3$): δ = 7.77–7.71 (m, 8H; ArH_{Tos}), 7.48 (s, 8H; ArH), 7.37–7.31 (m, 8H; ArH_{Tos}), 4.57 (d, 4H, ^2J = 14.2 Hz; $ArCH_2Ar$), 4.50–4.42 (m, 8H; CH_2CH_2), 3.36–3.28 (m, 8H; CH_2CH_2), 3.34 (d, 4H, ^2J = 14.2 Hz; $ArCH_2Ar$), 2.44 (s, 12H; CH_3) ppm. ^{13}C NMR (100 MHz, $CDCl_3$): δ = 160.32 (C_{Ar}), 145.60 ($C_{Ar\ Tos}$), 143.28, 135.18 (C_{Ar}), 132.16 ($C_{Ar\ Tos}$), 130.08, 127.83 ($CH_{Ar\ Tos}$), 124.13 (CH_{Ar}), 72.95, 69.14 (CH_2CH_2), 30.87 ($ArCH_2Ar$), 21.66 (CH_3) ppm. ESI-MS *m*/*z*: 1419.2372 $[M+Na]^+$ for $C_{64}H_{60}NaN_4O_{24}S_4$ (1419.2373).

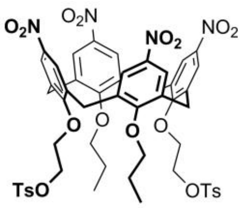

Exhaustively nitrated calix[4]arene ditosylate ***30*** was prepared as described for compound **29** from calixarene **28** (4.76 g, 4.22 mmol), fuming nitric acid (7.0 mL, 168.0 mmol) and glacial acetic acid (9.6 mL, 168.0 mmol) in dry dichloromethane (67.0 mL). Yield 3.53 g (77%), yellow crystals. M.p. 265–267 °C. ^{1}H NMR (400 MHz, $CDCl_3$): δ = 7.71 (s, 4H; ArH), 7.70–7.67 (m, 4H; ArH_{Tos}), 7.36–7.31 (m, 4H; ArH_{Tos}), 7.29 (s, 4H; ArH), 4.44 (d, 4H, ^2J = 14.3 Hz; $ArCH_2Ar$), 4.42–4.38 (m, 4H; OCH_2CH_2O), 4.34–4.28 (m, 4H; OCH_2CH_2O), 3.93–3.85 (m, 4H; $C\underline{H}_2CH_2CH_3$), 3.34 (d, 4H, ^2J = 14.3 Hz; $ArCH_2Ar$), 2.46 (s, 6H; $ArCH_3$), 1.92–1.80 (m, 4H; $C\underline{H}_2CH_3$), 0.99 (t, 6H, ^3J = 7.4 Hz; $CH_2C\underline{H}_3$) ppm. ^{13}C NMR (100 MHz, $CDCl_3+CD_3OD$): δ = 160.99, 160.93 (C_{Ar}), 145.54 ($C_{Ar\ Tos}$), 143.03, 142.65, 135.64, 134.67 (C_{Ar}), 132.21 ($C_{Ar\ Tos}$), 129.89, 127.56 ($CH_{Ar\ Tos}$), 124.15, 123.59 (CH_{Ar}), 78.14, 72.46, 68.28 (OCH_2), 30.82 ($ArCH_2Ar$), 22.98 ($\underline{C}H_2CH_3$), 21.43 ($ArCH_3$), 9.94 ($CH_2\underline{C}H_3$) ppm. ESI-MS *m*/*z*: 1107.2610 $[M+Na]^+$ for $C_{52}H_{52}NaN_4O_{18}S_2$ (1107.2610).

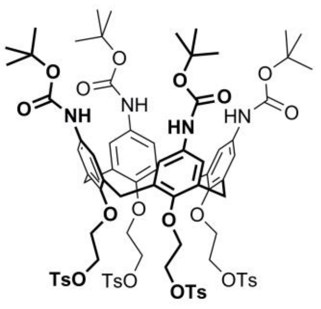

Boc-protected p-aminocalix[4]arene tetratosylate ***33***. To a stirred suspension of calixarene **29** (0.894 g, 0.64 mmol) in a mixture of ethanol (26 mL), THF (13.0 mL) and water (4.6 mL) $FeSO_4 \cdot 7H_2O$ (4.27 g, 15.37 mmol) followed by Zn powder (1.00 g, 15.37 mmol) and NH_4Cl (1.10 g, 20.49 mmol) were added. The mixture was stirred at 50 °C for 8 h. After cooling, the solvents were removed under reduced pressure, and the residue was suspended in a dichloromethane/ethanol (40:1) mixture. The suspension was filtered, the solid was washed with dichloromethane/ethanol (40:1) mixture and discarded. The combined filtrates were evaporated under reduced pressure. The residue was suspended in dry dichloromethane (25 mL), Boc_2O (0.70 g, 3.20 mmol) and Et_3N (0.45 mL, 3.20 mmol) were added, and the mixture was stirred at room temperature for 48 h. The solvent was removed under reduced pressure, and the residue was parted between dichloromethane and 2 M HCl. The organic layer was separated, washed with water, dried and the solvent was evaporated. The residue was purified by column chromatography (silica, gradient from dichloromethane to dichloromethane/ethanol 100:1).Yield 0.70 g (65%), white solid. M.p. 108–110 °C. ^1H NMR (400 MHz, $CDCl_3$) δ = 7.77–7.71 (m, 8H; ArH_{Tos}), 7.36–7.29 (m, 8H; ArH_{Tos}), 6.56 (s, 8H; ArH), 6.10 (s, 4H; NH), 4.39–4.28 (m, 8H; CH_2CH_2), 4.22 (d, 4H, ^2J = 13.4 Hz; $ArCH_2Ar$), 4.09–4.00 (m, 8H; CH_2CH_2), 2.97 (d, 4H, ^2J = 13.4 Hz; $ArCH_2Ar$), 2.42 (s, 12H; $ArCH_3$), 1.47 (s, 36H; $C(CH_3)_3$) ppm. ^{13}C NMR (100 MHz, $CDCl_3$): δ = 153.09 (C=O), 151.14 (C_{Ar}), 144.86 ($C_{Ar\ Tos}$), 134.69 (C_{Ar}), 132.86 ($C_{Ar\ Tos}$), 132.79 (C_{Ar}), 129.96, 127.89 ($CH_{Ar\ Tos}$), 119.70 (CH_{Ar}), 80.09 ($O\underline{C}(CH_3)_3$), 71.71, 69.55 (CH_2CH_2), 30.86 ($ArCH_2Ar$), 28.39 ($OC(\underline{C}H_3)_3$), 21.60 ($ArCH_3$) ppm. ESI-MS *m/z*: 1694.5944 $[M+NH_4]^+$ for $C_{84}H_{104}N_5O_{24}S_4$ (1694.5949).

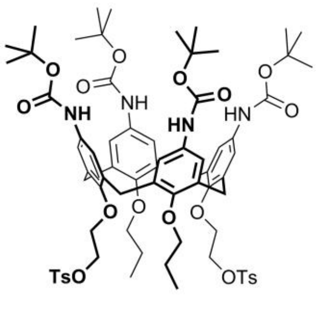

Boc-protected p-aminocalix[4]arene ditosylate ***34*** was prepared as described for compound **33** from calixarene **30** (0.054 g, 0.05 mmol), $FeSO_4 \cdot 7H_2O$ (0.333 g, 1.20 mmol), Zn powder (0.078 g, 1.20 mmol), NH_4Cl (0.086 g, 1.60 mmol) in a mixture of ethanol (2.0 mL), THF (1.0 mL) and water (0.036 mL), and then using Boc_2O (0.048 g, 0.22 mmol) and Et_3N (0.031 mL, 0.22 mmol) in dry dichloromethane (1.3 mL). Yield 0.053 g (77%), white solid. M.p. 149–151 °C. ^1H NMR (400 MHz, $CDCl_3$): δ = 7.73–7.68 (m, 4H; ArH_{Tos}), 7.35–7.30 (m, 4H; ArH_{Tos}), 6.97 (s, 4H; ArH), 6.34 (s, 2H; NH), 6.19 (s, 4H; ArH), 5.84 (s, 2H; NH), 4.47–4.38 (m, 4H; OCH_2), 4.20–4.11 (m, 4H; OCH_2), 4.16 (d, 4H, ^2J = 13.5 Hz; $ArCH_2Ar$), 3.60–3.51 (m, 4H; OCH_2), 3.03 (d, 4H, ^2J = 13.5 Hz; $ArCH_2Ar$), 2.45 (s, 6H; $ArCH_3$), 1.77–1.64 (m, 4H; $C\underline{H_2}CH_3$), 1.53 (s, 18H; $C(CH_3)_3$), 1.42 (s, 18H; $C(CH_3)_3$), 0.90 (t, 6H, ^3J = 7.4 Hz; $CH_2C\underline{H_3}$) ppm. ^{13}C NMR (100 MHz, $CDCl_3$): δ =

153.39, 152.90 (C=O), 152.27, 151.55 (C_{Ar}), 144.69 ($C_{Ar\,Tos}$), 136.01, 133.43 (C_{Ar}), 133.13 ($C_{Ar\,Tos}$), 133.03, 132.03 (C_{Ar}), 129.82, 127.74 ($CH_{Ar\,Tos}$), 120.21, 119.16 (CH_{Ar}), 80.20, 79.87 ($O\underline{C}(CH_3)_3$), 77.34, 71.06, 68.85 (OCH_2), 30.97 ($ArCH_2Ar$), 28.38, 28.35 ($OC(\underline{C}H_3)_3$), 22.99 ($\underline{C}H_2CH_3$), 21.60 ($ArCH_3$), 10.41 ($CH_2\underline{C}H_3$) ppm. ESI-MS *m/z*: 1387.5741 $[M+Na]^+$ for $C_{72}H_{92}NaN_4O_{18}S_2$ (1387.5740).

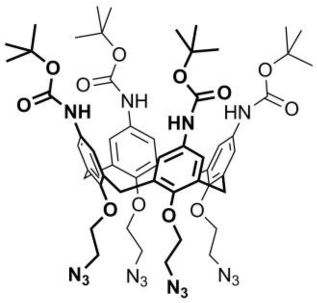

*Boc-protected p-aminocalix[4]arene tetraazide **35***. A mixture of calixarene **33** (0.493 g, 0.29 mmol) and NaN_3 (0.153 g, 2.35 mmol) in dry DMF (10 mL) was stirred at 60 °C for 6 h. The solvent was removed under reduced pressure, water was added to the residue, the solid formed was collected, washed with water and dried. Yield 0.297 g (87%), white solid. M.p. 223–225 °C. ^1H NMR (400 MHz, $CDCl_3$) δ = 6.66 (s, 8H; ArH), 6.17 (s, 4H; NH), 4.33 (d, 4H, ^2J = 13.4 Hz; $ArCH_2Ar$), 4.03–3.96 (m, 8H; OCH_2CH_2), 3.77–3.69 (m, 8H; OCH_2CH_2), 3.16 (d, 4H, ^2J = 13.4 Hz; $ArCH_2Ar$), 1.48 (s, 36H; $C(CH_3)_3$) ppm. ^{13}C NMR (100 MHz, $CDCl_3$): δ = 153.18 (C=O), 151.47 (C_{Ar}), 134.68, 132.97 (C_{Ar}), 119.88 (CH_{Ar}), 80.21 ($O\underline{C}(CH_3)_3$), 72.12 (OCH_2), 51.00 (CH_2N_3), 30.79 ($ArCH_2Ar$), 28.38 ($OC(\underline{C}H_3)_3$) ppm. ESI-MS *m/z*: 1178.5853 $[M+NH_4]^+$ for $C_{56}H_{76}N_{17}O_{12}$ (1178.5854).

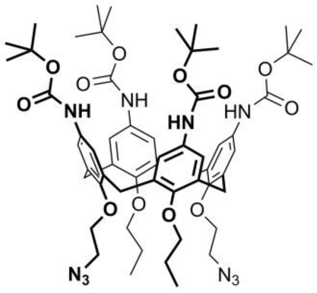

*Boc-protected p-aminocalix[4]arene diazide **36*** was prepared as described for compound **35** from calixarene **34** (0.327 g, 0.24 mmol) and NaN_3 (0.064 g, 0.96 mmol) in dry DMF (6 mL). Yield 0.254 g (96%), white solid. M.p. 147–149 °C. ^1H NMR (400 MHz, $CDCl_3$): δ = 6.97 (s, 4H; ArH), 6.34 (s, 2H; NH), 6.29 (s, 4H; ArH), 5.94 (s, 2H; NH), 4.30 (d, 4H, ^2J = 13.5 Hz; $ArCH_2Ar$), 4.10–4.04 (m, 4H; OCH_2CH_2), 3.79–3.71 (m, 4H; OCH_2CH_2), 3.71–3.62 (m, 4H; OCH_2CH_2), 3.13 (d, 4H, ^2J = 13.5 Hz; $ArCH_2Ar$), 1.91–1.80 (m, 4H; $C\underline{H}_2CH_3$), 1.51 (s, 18H; $C(CH_3)_3$), 1.44 (s, 18H; $C(CH_3)_3$), 1.01 (t, 6H, ^3J = 7.4 Hz; $CH_2C\underline{H}_3$) ppm. ^{13}C NMR (100 MHz, $CDCl_3$): δ = 153.42, 153.02 (C=O), 152.34, 151.75 (C_{Ar}), 135.95, 133.74, 132.96, 132.12 (C_{Ar}), 120.20, 119.42 (CH_{Ar}), 80.23, 79.94 ($O\underline{C}(CH_3)_3$), 77.25, 71.57 (OCH_2), 50.64 (CH_2N_3), 30.92 ($ArCH_2Ar$), 28.38 ($OC(\underline{C}H_3)_3$), 23.25 ($\underline{C}H_2CH_3$), 10.53 ($CH_2\underline{C}H_3$) ppm. ESI-MS *m/z*: 1129.5693 $[M+Na]^+$ for $C_{58}H_{78}NaN_{10}O_{12}$ (1129.5693).

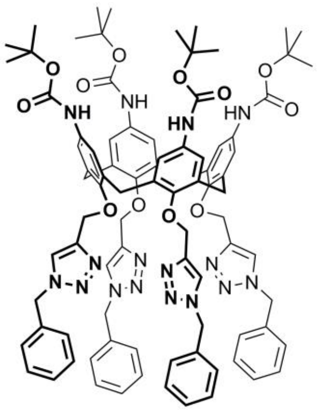

Tetratriazolated Boc-protected p-aminocalix[4]arene ***37***. To a stirred suspension of CuI (0.033 g, 0.17 mmol) in toluene (5 mL) triethylamine (0.488 mL, 3.50 mmol) was added. The resultant solution was added to a solution of calixarene **24** (0.302 g, 0.29 mmol) and benzyl azide (0.186 g, 1.40 mmol) in toluene (20 mL). The reaction mixture was stirred at room temperature in an argon-flushed flask for 72 h. The solvent was removed under reduced pressure, dichloromethane and aqueous $Na_2S_2O_3$ (10%) were added and the mixture was stirred vigorously for 3 h. The organic phase was separated, washed with water, dried and the solvent was evaporated. The residue was purified by column chromatography (silica, gradient from dichloromethane to dichloromethane/ethanol 20:1). Yield 0.257 g (56%), white solid. M.p. 188–190 °C. ^{1}H NMR (400 MHz, $CDCl_3$): δ = 7.69 (s, 4H; ArH_{Trz}), 7.29–7.22 (m, 12H; ArH_{Ph}), 7.19–7.11 (m, 8H; ArH_{Ph}), 6.53 (s, 8H; ArH), 6.08 (s, 4H; NH), 5.44 (s, 8H; CH_2Ph), 4.90 (s, 8H; OCH_2Trz), 4.04 (d, 4H, ^2J = 13.2 Hz; $ArCH_2Ar$), 2.77 (d, 4H, ^2J = 13.2 Hz; $ArCH_2Ar$), 1.47 (s, 36H; $OC(CH_3)_3$) ppm. ^{13}C NMR (100 MHz, $CDCl_3$): δ = 153.00 (C=O), 150.36 (C_{Ar}), 143.93 ($C_{Ar\,Trz}$), 135.18, 134.83, 132.63 (C_{Ar}), 128.82, 128.39, 127.86 (CH_{Ar}), 124.68 ($CH_{Ar\,Trz}$), 119.35 (CH_{Ar}), 79.86 ($O\underline{C}(CH_3)_3$), 65.94 (OCH_2), 53.94 (CH_2Ph), 31.30 ($ArCH_2Ar$), 28.29 ($C(\underline{C}H_3)_3$) ppm. ESI-MS *m*/*z*: 1569.7465 $[M+H]^+$ for $C_{88}H_{97}N_{16}O_{12}$ (1569.7466).

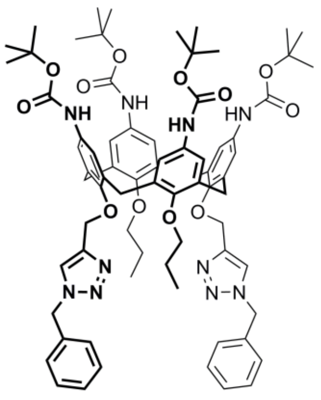

Ditriazolated Boc-protected p-aminocalix[4]arene ***38*** was prepared as described for compound **37** from calixarene **25** (1.04 g, 1.00 mmol), benzyl azide (0.32 g, 2.40 mmol), CuI (0.057 g, 0.30 mmol) and triethylamine (0.836 mL, 6.00 mmol) in toluene (80 mL). The product was additionally purified by recrystallization from hexane. Yield 1.12 g (86%), white solid. M.p. 148–150 °C. ^{1}H NMR (400 MHz, $CDCl_3$): δ = 7.43 (s, 2H; ArH_{Trz}), 7.38–7.29 (m, 6H; ArH_{Ph}), 7.23–7.18 (m, 4H; ArH_{Ph}), 6.81 (s, 4H; ArH), 6.28 (s, 4H; ArH), 6.26 (s, 2H; NH), 5.96 (s, 2H; NH), 5.51 (s, 4H; NCH_2Ph), 5.21 (s, 4H; OCH_2Trz), 4.05 (d, 4H, ^2J = 13.3 Hz; $ArCH_2Ar$), 3.54–3.45 (m, 4H; $OC\underline{H}_2CH_2$), 2.88 (d, 4H, ^2J = 13.3 Hz; $ArCH_2Ar$), 1.71–1.57 (m, 4H; $OCH_2C\underline{H}_2$), 1.52 (s, 18H; $OC(CH_3)_3$), 1.43 (s, 18H; $OC(CH_3)_3$), 0.79 (t, 6H, ^3J = 7.4 Hz; $CH_2C\underline{H}_3$) ppm. ^{13}C NMR (100 MHz, $CDCl_3$): δ = 153.42, 152.96, 152.31, 150.99 (C=O, C_{Ar}), 144.73 ($C_{Ar\,Trz}$), 136.60, 135.03, 133.91, 132.68, 131.73 (C_{Ar}), 128.93, 128.47, 127.79 (CH_{Ar}), 123.74 ($CH_{Ar\,Trz}$), 120.03, 119.09 (CH_{Ar}), 80.04, 79.79 ($\underline{C}(CH_3)_3$), 76.85 ($O\underline{C}H_2CH_2$), 66.00 (OCH_2Trz), 53.83 (NCH_2), 31.27 ($ArCH_2Ar$), 28.38, 28.34 ($C(\underline{C}H_3)_3$), 22.84

($\underline{C}H_2CH_3$), 10.33 ($CH_2\underline{C}H_3$) ppm. ESI-MS *m/z*: 1311.6809 $[M+H]^+$ for $C_{74}H_{91}N_{10}O_{12}$ (1311.6812).

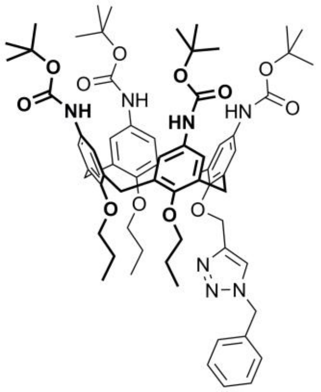

Triazolated Boc-protected p-aminocalix[4]arene ***39*** was prepared as described for compound **37** from calixarene **26** (0.62 g, 0.59 mmol), benzyl azide (0.094 g, 0.71 mmol), CuI (0.017 g, 0.09 mmol) and triethylamine (0.247 mL, 1.77 mmol) in toluene (50 mL). The product was additionally purified by recrystallization from hexane. Yield 0.521 g (75%), white solid. M.p. 156–158 °C. ^{1}H NMR (400 MHz, $CDCl_3$): δ = 7.39–7.31 (m, 3H; ArH_{Ph}), 7.21–7.16 (m, 2H; ArH_{Ph}), 7.19 (s, 1H; ArH_{Trz}), 6.66 (s, 2H; ArH), 6.60 (s, 2H; ArH), 6.54–6.45 (m, 4H; ArH), 6.15 (s, 1H; NH), 6.13 (s, 1H; NH), 6.07 (s, 2H; NH), 5.48 (s, 2H; NCH_2Ph), 5.11 (s, 2H; OCH_2Trz), 4.35 (d, 2H, $^2J = 13.2$ Hz; $ArCH_2Ar$), 4.12 (d, 2H, $^2J = 13.4$ Hz; $ArCH_2Ar$), 3.85–3.77 (m, 2H; $OC\underline{H}_2CH_2$), 3.69–3.58 (m, 4H; $OC\underline{H}_2CH_2$), 3.06 (d, 2H, $^2J = 13.4$ Hz; $ArCH_2Ar$), 2.88 (d, 2H, $^2J = 13.2$ Hz; $ArCH_2Ar$), 1.95–1.82 (m, 2H; $OCH_2C\underline{H}_2$), 1.82–1.70 (m, 4H; $OCH_2C\underline{H}_2$), 1.50 (s, 9H; $C(CH_3)_3$), 1.48 (s, 9H; $C(CH_3)_3$), 1.46 (s, 18H; $C(CH_3)_3$), 0.93 (t, 3H, $^3J = 7.5$ Hz; $CH_2C\underline{H}_3$), 0.85 (t, 6H, $^3J = 7.5$ Hz; $CH_2C\underline{H}_3$) ppm. ^{13}C NMR (100 MHz, $CDCl_3$): δ = 153.35, 153.27, 153.10, 152.85, 152.74, 150.68 (C=O, C_{Ar}), 144.78 ($C_{Ar\ Trz}$), 135.99, 135.46, 134.92, 134.87, 134.74, 132.55, 131.97, 131.87 (C_{Ar}), 129.06, 128.65, 127.84 (CH_{Ar}), 123.14 ($CH_{Ar\ Trz}$), 119.93, 119.83, 119.68, 119.36 (CH_{Ar}), 80.03, 79.99, 73.93 ($\underline{C}(CH_3)_3$), 76.79, 76.73 ($O\underline{C}H_2CH_2$), 66.07 (OCH_2Trz), 53.96 (NCH_2), 31.32, 31.08 ($ArCH_2Ar$), 28.44, 28.42 ($C(\underline{C}H_3)_3$), 23.13, 23.00 ($\underline{C}H_2CH_3$), 10.31 ($CH_2\underline{C}H_3$) ppm. ESI-MS *m/z*: 1182.6489 $[M+H]^+$ for $C_{67}H_{88}N_7O_{12}$ (1182.6485).

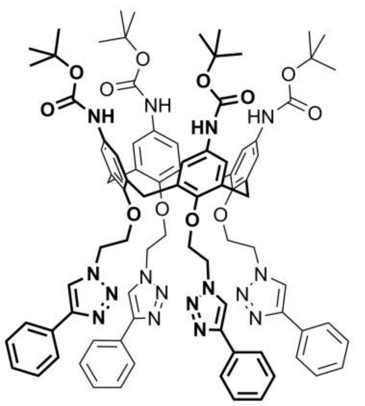

Tetratriazolated Boc-protected p-aminocalix[4]arene ***40***. A mixture of calixarene **35** (0.047 g, 0.04 mmol), phenylacetylene (0.022 mL, 0.20 mmol) and CuI·P(OEt)$_3$ (0.002 g, 0.006 mmol) in toluene (6 mL) was stirred at 100 °C in an argon-flushed flask for 8 h. The solvent was removed under reduced pressure, dichloromethane and aqueous $Na_2S_2O_3$ (10%) were added and the mixture was stirred vigorously for 3 h. The organic phase was separated, washed with water, dried and the solvent was evaporated. The residue was dissolved in a minimum amount of dichloromethane, hexane was added, and the solid formed was collected, washed with hexane and dried, and the product was purified by column chromatography (silica, gradient from dichloromethane to dichloromethane/ethanol 75:1). Yield 0.052 g (82%), white solid. M.p. 168–170 °C. ^{1}H NMR

(400 MHz, $CDCl_3$): δ = 7.82 (s, 4H; ArH_{Trz}), 7.75–7.67 (m, 8H; ArH_{Ph}), 7.32–7.18 (m, 12H; ArH_{Ph}), 6.56 (s, 8H; ArH), 6.11 (s, 4H; NH), 4.69–4.57 (m, 8H; OCH_2CH_2), 4.23–4.10 (m, 8H; OCH_2CH_2), 4.00 (d, 4H, ^2J = 13.6 Hz; $ArCH_2Ar$), 3.03 (d, 4H, ^2J = 13.6 Hz; $ArCH_2Ar$), 1.46 (s, 36H; $C(CH_3)_3$) ppm. ^{13}C NMR (100 MHz, $CDCl_3$): δ = 153.10 (C_{Ar}), 150.97 (C=O), 147.61 ($C_{Ar\ Trz}$), 134.53, 133.14, 130.31 (C_{Ar}), 128.85, 128.10, 125.49 (CH_{Ar}), 120.82 ($CH_{Ar\ Trz}$), 119.90 (CH_{Ar}), 80.24 ($\underline{C}(CH_3)_3$), 72.07 (OCH_2), 50.13 (NCH_2), 30.42 ($ArCH_2Ar$), 28.37 ($C(\underline{C}H_3)_3$) ppm. ESI-MS *m/z*: 1569.7469 $[M+H]^+$ for $C_{88}H_{97}N_{16}O_{12}$ (1569.7466).

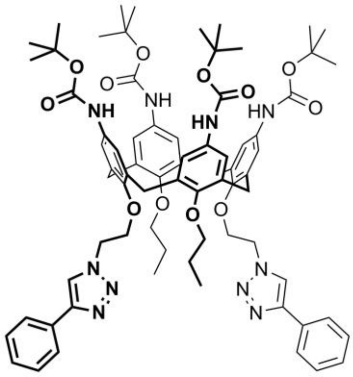

Ditriazolated Boc-protected p-aminocalix[4]arene ***41*** was prepared as described for compound **37** from calixarene **36** (0.089 g, 0.08 mmol), phenylacetylene (0.021 mL, 0.19 mmol), CuI (0.005 g, 0.024 mmol) and triethylamine (1.4 mL) in toluene (5.6 mL). The residue obtained after extraction was dissolved in a minimum amount of dichloromethane, hexane was added, and the solid formed was collected, washed with hexane and dried. Yield 0.088 g (84%), white solid. M.p. 188–190 °C. ^{1}H NMR (400 MHz, $CDCl_3$): δ = 8.13 (s, 2H; ArH_{Trz}), 7.99–7.91 (m, 4H; ArH_{Ph}), 7.46–7.38 (m, 4H; ArH_{Ph}), 7.36–7.29 (m, 2H; ArH_{Ph}), 7.18 (s, 4H; ArH), 6.45 (s, 2H; NH), 6.18 (s, 4H; ArH), 5.84 (s, 2H; NH), 5.04–4.93 (m, 4H; NCH_2CH_2), 4.63–4.51 (m, 4H; NCH_2CH_2), 4.28 (d, 4H, ^2J = 13.6 Hz; $ArCH_2Ar$), 3.67–3.54 (m, 4H; OCH_2), 3.18 (d, 4H, ^2J = 13.6 Hz; $ArCH_2Ar$), 1.81–1.68 (m, 4H; $C\underline{H}_2CH_3$), 1.55 (s, 18H; $C(CH_3)_3$), 1.43 (s, 18H; $C(CH_3)_3$), 0.86 (t, 6H, ^3J = 7.4 Hz; $CH_2C\underline{H}_3$) ppm. ^{13}C NMR (100 MHz, $CDCl_3$): δ = 153.47, 152.93, 152.08, 151.35 (C=O, C_{Ar}), 147.78 ($C_{Ar\ Trz}$), 136.49, 133.60, 133.07, 132.30, 130.66 (C_{Ar}), 128.75, 128.00, 125.74 (CH_{Ar}), 120.64 ($CH_{Ar\ Trz}$), 120.39, 119.50 (CH_{Ar}), 80.39, 79.96 ($\underline{C}(CH_3)_3$), 77.49, 72.15 (OCH_2), 48.84 (NCH_2), 31.10 ($ArCH_2Ar$), 28.39, 28.38 ($C(\underline{C}H_3)_3$), 23.28 ($\underline{C}H_2CH_3$), 10.43 ($CH_2\underline{C}H_3$) ppm. ESI-MS *m/z*: 1311.6814 $[M+H]^+$ for $C_{74}H_{91}N_{10}O_{12}$ (1311.6812).

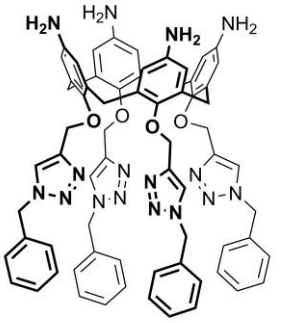

Tetratriazolated p-aminocalix[4]arene ***42***. To a stirred solution of calixarene **37** (0.201 g, 0.13 mmol) in dry dichloromethane (10 mL) trifluoroacetic acid (0.786 mL, 10.26 mmol) was added. The mixture was stirred for 2 h at room temperature and then evaporated under reduced pressure. The residue was dissolved in dichloromethane, the solution was washed with aqueous $NaHCO_3$ (5%), water, dried and the solvent was evaporated. Yield 0.135 g (90%), off-white solid. M.p. 135–137 °C. ^{1}H NMR (400 MHz, $CDCl_3$): δ = 7.71 (s, 4H; ArH_{Trz}), 7.33–7.07 (m, 20H; ArH_{Ph}), 5.92 (s, 8H; ArH), 5.43 (s, 8H; NCH_2Ph), 4.83 (s, 8H; OCH_2Trz), 3.95 (d, 4H, ^2J = 13.2 Hz; $ArCH_2Ar$), 2.97 (bs, 8H; NH_2),

2.61 (d, 4H, ^2J = 13.2 Hz; $ArCH_2Ar$) ppm. ^{13}C NMR (100 MHz, $CDCl_3$): δ = 147.84 (C_{Ar}), 144.90 ($C_{Ar\ Trz}$), 140.80, 135.65, 135.27 (C_{Ar}), 128.79, 128.26, 127.81 (CH_{Ar}), 124.40 ($CH_{Ar\ Trz}$), 115.49 (CH_{Ar}), 66.35 (OCH_2Trz), 53.72 (NCH_2), 31.36 ($ArCH_2Ar$) ppm. ESI-MS *m*/*z*: 1169.5364 $[M+H]^+$ for $C_{68}H_{65}N_{16}O_4$ (1169.5369); 585.2727 $[M+2H]^{2+}$ for $C_{68}H_{66}N_{16}O_4$ (585.2721).

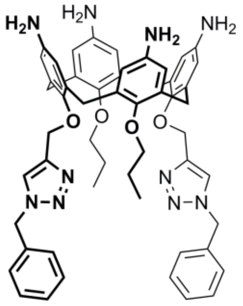

Ditriazolated p-aminocalix[4]arene ***43*** was prepared as described for compound **42** from calixarene **38** (1.03 g, 0.78 mmol) and trifluoroacetic acid (4.8 mL, 62.7 mmol) in dry dichloromethane (60 mL). Yield 0.64 g (90%), off-white solid. M.p. 147–149 °C. ^{1}H NMR (400 MHz, $CDCl_3$): δ = 7.57 (s, 2H; ArH_{Trz}), 7.38–7.28 (m, 6H; ArH_{Ph}), 7.24–7.19 (m, 4H; ArH_{Ph}), 6.15 (s, 4H; ArH), 5.79 (s, 4H; ArH), 5.52 (s, 4H; NCH_2Ph), 5.11 (s, 4H; OCH_2Trz), 4.01 (d, 4H, ^2J = 13.2 Hz; $ArCH_2Ar$), 3.50–3.40 (m, 4H; $OC\underline{H}_2CH_2$), 3.04 (bs, 8H; NH_2), 2.73 (d, 4H, ^2J = 13.2 Hz; $ArCH_2Ar$), 1.68–1.53 (m, 4H; $OCH_2C\underline{H}_2$), 0.76 (t, 6H, ^3J = 7.5 Hz; CH_3) ppm. ^{13}C NMR (100 MHz, $CDCl_3$): δ = 148.48, 148.44 (C_{Ar}), 145.38 ($C_{Ar\ Trz}$), 136.69, 136.65, 135.18, 134.37, 134.35 (C_{Ar}), 128.84, 128.33, 127.67 (CH_{Ar}), 123.71 ($CH_{Ar\ Trz}$), 115.68, 115.44 (CH_{Ar}), 76.68 ($O\underline{C}H_2CH_2$), 66.30 (OCH_2Trz), 53.72 (NCH_2), 31.31 ($ArCH_2Ar$), 22.83 ($\underline{C}H_2CH_3$), 10.33 (CH_3) ppm. ESI-MS *m*/*z*: 911.4713 $[M+H]^+$ for $C_{54}H_{59}N_{10}O_4$ (911.4715).

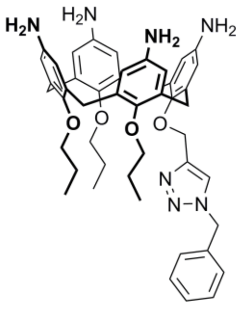

Triazolated p-aminocalix[4]arene ***44*** was prepared as described for compound **42** from calixarene **39** (0.513 g, 0.43 mmol) and trifluoroacetic acid (2.66 mL, 34.7 mmol) in dry dichloromethane (35 mL). Yield 0.317 g (93%), off-white solid. M.p. 156–158 °C. ^{1}H NMR (400 MHz, $CDCl_3$): δ = 7.38 (s, 1H; ArH_{Trz}), 7.37–7.32 (m, 3H; ArH_{Ph}), 7.22–7.18 (m, 2H; ArH_{Ph}), 6.07 (s, 2H; ArH), 6.02–5.98 (m, 4H; ArH), 5.97–5.94 (m, 2H; ArH), 5.50 (s, 2H; NCH_2Ph), 5.04 (s, 2H; OCH_2Trz), 4.28 (d, 2H, ^2J = 13.2 Hz; $ArCH_2Ar$), 4.09 (d, 2H, ^2J = 13.2 Hz; $ArCH_2Ar$), 3.77–3.71 (m, 2H; $OC\underline{H}_2CH_2$), 3.65–3.56 (m, 4H; $OC\underline{H}_2CH_2$), 3.13 (bs, 8H; NH_2), 2.90 (d, 2H, ^2J = 13.2 Hz; $ArCH_2Ar$), 2.74 (d, 2H, ^2J = 13.2 Hz; $ArCH_2Ar$), 1.91–1.82 (m, 2H; $OCH_2C\underline{H}_2$), 1.80–1.69 (m, 4H; $OCH_2C\underline{H}_2$), 0.91 (t, 3H, ^3J = 7.5 Hz; CH_3), 0.83 (t, 6H, ^3J = 7.5 Hz; CH_3) ppm. ^{13}C NMR (100 MHz, $CDCl_3$): δ = 149.85, 149.81, 148.03 (C_{Ar}), 145.55 ($C_{Ar\ Trz}$), 140.26, 135.93, 135.65, 135.36, 135.18, 134.98 (C_{Ar}), 128.94, 128.47, 127.67 (CH_{Ar}), 123.07 ($CH_{Ar\ Trz}$), 115.62 (bs; CH_{Ar}), 76.62 (bs; $O\underline{C}H_2CH_2$), 66.41 (OCH_2Trz), 53.84 (NCH_2), 31.31, 31.08 ($ArCH_2Ar$), 23.12, 22.95 ($\underline{C}H_2CH_3$), 10.33, 10.27 (CH_3) ppm. ESI-MS *m*/*z*: 782.4383 $[M+H]^+$ for $C_{47}H_{56}N_7O_4$ (782.4388), 391.7234 $[M+2H]^{2+}$ for $C_{47}H_{57}N_7O_4$ (391.7231).

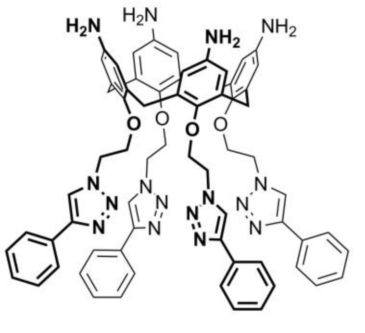

Tetratriazolated p-aminocalix[4]arene ***45*** was prepared as described for compound **42** from calixarene **40** (0.025 g, 0.016 mmol) and trifluoroacetic acid (0.098 mL, 1.275 mmol) in dry dichloromethane (1.5 mL). Yield 0.018 g (94%), off-white solid. M.p. 183–185 °C. ^{1}H NMR (400 MHz, $CDCl_3$): δ = 7.84 (s, 4H; ArH_{Trz}), 7.78–7.65 (m, 8H; ArH_{Ph}), 7.33–7.17 (m, 12H; ArH_{Ph}), 5.95 (s, 8H; ArH), 4.69–4.49 (m, 8H; OCH_2CH_2), 4.18–4.07 (m, 8H; OCH_2CH_2), 3.87 (d, 4H, ^{2}J = 13.6 Hz; $ArCH_2Ar$), 2.85 (d, 4H, ^{2}J = 13.6 Hz; $ArCH_2Ar$), 2.67 (bs, 8H; NH_2) ppm. ^{13}C NMR (100 MHz, $CDCl_3$): δ = 147.48 (C_{Ar}), 141.34 ($C_{Ar\ Trz}$), 135.02 (bs; C_{Ar}), 130.41 (C_{Ar}), 128.83, 128.05, 125.47 (CH_{Ar}), 120.92 ($CH_{Ar\ Trz}$), 115.82 (CH_{Ar}), 71.95 (OCH_2), 50.18 (NCH_2), 30.41 ($ArCH_2Ar$) ppm. ESI-MS *m/z*: 1169.5367 $[M+H]^+$ for $C_{68}H_{65}N_{16}O_4$ (1169.5369).

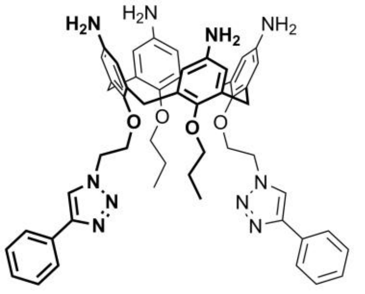

Ditriazolated p-aminocalix[4]arene ***46*** was prepared as described for compound **42** from calixarene **41** (0.088 g, 0.067 mmol) and trifluoroacetic acid (0.411 mL, 5.37 mmol) in dry dichloromethane (5.5 mL). Yield 0.050 g (82%), off-white solid. M.p. 188–190 °C. ^{1}H NMR (400 MHz, $CDCl_3$): δ = 8.14 (s, 2H; ArH_{Trz}), 8.01–7.91 (m, 4H; ArH_{Ph}), 7.47–7.37 (m, 4H; ArH_{Ph}), 7.36–7.28 (m, 2H; ArH_{Ph}), 6.49 (s, 4H; ArH), 5.69 (s, 4H; ArH), 5.08–4.93 (m, 4H; NCH_2CH_2), 4.54–4.42 (m, 4H; NCH_2CH_2), 4.22 (d, 4H, ^{2}J = 13.4 Hz; $ArCH_2Ar$), 3.67–3.50 (m, 4H; OCH_2), 3.29 (bs, 8H; NH_2), 3.01 (d, 4H, ^{2}J = 13.4 Hz; $ArCH_2Ar$), 1.82–1.66 (m, 4H; $C\underline{H}_2CH_3$), 0.88 (t, 6H, ^{3}J = 7.4 Hz; $CH_2C\underline{H}_3$) ppm. ^{13}C NMR (100 MHz, $CDCl_3$): δ = 149.46, 148.14, 147.64, 141.47 (C_{Ar}), 140.89 ($C_{Ar\ Trz}$), 137.12, 133.21, 130.69 (C_{Ar}), 128.70, 127.91, 125.66 (CH_{Ar}), 120.62 ($CH_{Ar\ Trz}$), 116.05, 115.40 (CH_{Ar}), 77.32, 72.11 (OCH_2), 48.88 (NCH_2), 31.06 ($ArCH_2Ar$), 23.30 ($\underline{C}H_2CH_3$), 10.50 ($CH_2\underline{C}H_3$) ppm. ESI-MS *m/z*: 911.4712 $[M+H]^+$ for $C_{54}H_{59}N_{10}O_4$ (911.4715).

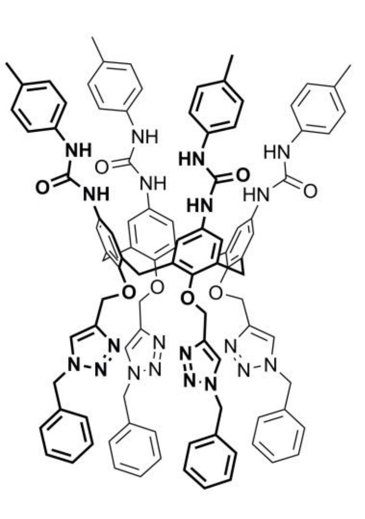

Tetratriazolated tetraureacalix[4]arene ***47***. To a solution of calixarene **42** (0.058 g, 0.05 mmol) in dry toluene (7.5 mL) *p*-tolyl isocyanate (0.050 mL, 0.40 mmol) was added and the reaction mixture was stirred at 60 °C for 12 h. After cooling, the solvent was evaporated under reduced pressure and the residue was purified by column chromatography (silica, gradient from dichloromethane to dichloromethane/ethanol 25:1) followed by recrystallization of the product from hexane. Yield 0.057 g (67%),

off-white solid. M.p. 197–199 °C. ^{1}H NMR (400 MHz, DMSO-d_6): δ = 8.25 (s, 4H; NH), 8.20 (s, 4H; NH), 8.08 (s, 4H; ArH_{Trz}), 7.32–7.25 (m, 12H, ArH_{Ph}), 7.25–7.21 (m, 8H; ArH_{Tol}), 7.19–7.14 (m, 8H; ArH_{Ph}), 7.05–7.00 (m, 8H; ArH_{Tol}), 6.72 (s, 8H; ArH), 5.58 (s, 8H; NCH_2Ph), 4.96 (s, 8H; OCH_2Trz), 3.97 (d, 4H, ^{2}J = 12.8 Hz; $ArCH_2Ar$), 2.64 (d, 4H, ^{2}J = 12.8 Hz; $ArCH_2Ar$), 2.21 (s, 12H; CH_3) ppm. ^{13}C NMR (100 MHz, DMSO-d_6): δ = 152.42 (C=O), 149.25 (C_{Ar}), 143.55 ($C_{Ar\ Trz}$), 137.28, 136.22, 134.86, 133.87, 130.29 (C_{Ar}), 129.11, 128.64, 127.97, 127.53 (CH_{Ar}), 125.18 ($CH_{Ar\ Trz}$), 118.04, 117.68 (CH_{Ar}), 65.66 (OCH_2Trz), 52.63 (NCH_2), 31.45 ($ArCH_2Ar$), 20.34 (CH_3) ppm. ESI-MS *m/z*: 851.8793 $[M+2H]^{2+}$ for $C_{100}H_{94}N_{20}O_8$ (851.8793).

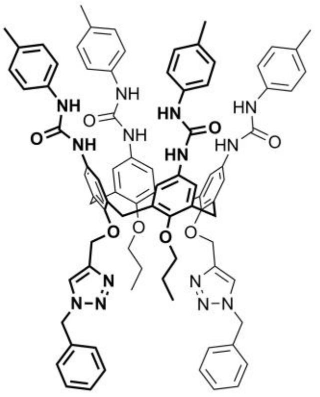

Ditriazolated tetraureacalix[4]arene ***48*** was prepared as described for compound **47** from calixarene **43** (0.137 g, 0.15 mmol) and *p*-tolyl isocyanate (0.151 mL, 1.20 mmol) in dry toluene (15 mL). Instead of column chromatography, the product was purified by recrystallization from methanol. Yield 0.120 g (56%), off-white solid. M.p. 247–249 °C. ^{1}H NMR (400 MHz, DMSO-d_6): δ = 8.22 (s, 4H; NH), 8.18 (s, 2H; NH), 8.17 (s, 2H; NH), 8.04 (s, 2H; ArH_{Trz}), 7.42–7.30 (m, 6H; ArH_{Ph}), 7.26–7.19 (m, 12H; ArH_{Ph}+ArH_{Tol}), 7.06–7.00 (m, 8H; ArH_{Tol}), 6.81 (s, 4H; ArH), 6.78 (s, 4H; ArH), 5.60 (s, 4H; NCH_2Ph), 5.10 (s, 4H; OCH_2Trz), 4.23 (d, 4H, ^{2}J = 12.8 Hz; $ArCH_2Ar$), 3.64–3.56 (m, 4H; $OC\underline{H}_2CH_2$), 2.92 (d, 4H, ^{2}J = 12.8 Hz; $ArCH_2Ar$), 2.21 (s, 12H; $PhCH_3$), 1.75–1.63 (m, 4H; $OCH_2C\underline{H}_2$), 0.70 (t, 6H, ^{3}J = 7.5 Hz; $CH_2C\underline{H}_3$) ppm. ^{13}C NMR (100 MHz, DMSO-d_6): δ = 152.48, 152.42 (C=O), 151.38, 149.18 (C_{Ar}), 143.29 ($C_{Ar\ Trz}$), 137.29, 137.25, 136.22, 134.97, 134.38, 133.84, 133.45, 130.31, 130.26 (C_{Ar}), 129.10, 129.09, 128.68, 128.01, 127.61 (CH_{Ar}), 124.92 ($CH_{Ar\ Trz}$), 118.13, 118.05, 118.02, 117.74 (CH_{Ar}), 76.44 ($O\underline{C}H_2CH_2$), 65.93 (OCH_2Trz), 52.56 (NCH_2), 31.10 ($ArCH_2Ar$), 22.38 ($\underline{C}H_2CH_3$), 20.33 ($PhCH_3$), 10.00 ($CH_2\underline{C}H_3$) ppm. ESI-MS *m/z*: 722.3441 $[M+2H]^{2+}$ for $C_{86}H_{88}N_{14}O_8$ (722.3449).

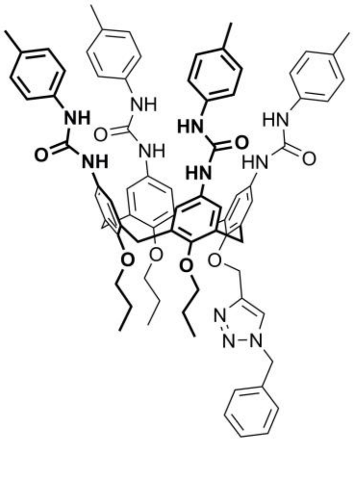

Triazolated tetraureacalix[4]arene ***49*** was prepared as described for compound **47** from calixarene **44** (0.141 g, 0.18 mmol) and *p*-tolyl isocyanate (0.182 mL, 1.44 mmol) in dry toluene (18 mL). Instead of column chromatography, the product was purified by recrystallization from methanol. Yield 0.168 g (71%), off-white solid. M.p. 214–216 °C. ^{1}H NMR (400 MHz, DMSO-d_6): δ = 8.24 (s, 2H; NH), 8.21 (s, 2H; NH), 8.20 (s, 1H; NH), 8.19 (s, 1H; NH), 8.16 (s, 1H; NH), 8.12 (s, 1H; NH), 8.01 (s, 1H; ArH_{Trz}), 7.41–7.29 (m, 3H; ArH_{Ph}), 7.27–7.18 (m, 10H; ArH_{Ph}+ArH_{Tol}), 7.06–6.99 (m,

8H; ArH_{Tol}), 6.88–6.84 (m, 4H; ArH), 6.76 (s, 2H; ArH), 6.75 (s, 2H; ArH), 5.60 (s, 2H; NCH_2Ph), 5.06 (s, 2H; OCH_2Trz), 4.33 (d, 2H, ^2J = 12.7 Hz; $ArCH_2Ar$), 4.25 (d, 2H, ^2J = 12.7 Hz; $ArCH_2Ar$), 3.78–3.72 (m, 2H; $OC\underline{H}_2CH_2$), 3.73–3.67 (m, 4H; $OC\underline{H}_2CH_2$), 3.09 (d, 2H, ^2J = 12.7 Hz; $ArCH_2Ar$), 2.94 (d, 2H, ^2J = 12.7 Hz; $ArCH_2Ar$), 2.22 (s, 9H; $PhCH_3$), 2.21 (s, 3H; $PhCH_3$), 1.99–1.88 (m, 2H; $OCH_2C\underline{H}_2$), 1.87–1.75 (m, 4H; $OCH_2C\underline{H}_2$), 0.99 (t, 3H, ^3J = 7.5 Hz; $CH_2C\underline{H}_3$), 0.82 (t, 6H, ^3J = 7.5 Hz; $CH_2C\underline{H}_3$) ppm. ^{13}C NMR (100 MHz, DMSO-d_6): δ = 152.50, 152.46, 152.42 (C=O), 151.27, 150.82, 149.07 (C_{Ar}), 143.23 ($C_{Ar\ Trz}$), 137.29, 137.27, 137.26, 136.22, 134.85, 134.58, 134.55, 134.16, 133.83, 133.51, 133.44, 130.29, 130.28, 130.25 (C_{Ar}), 129.10, 129.08, 128.67, 128.01, 127.61 (CH_{Ar}), 124.82 ($CH_{Ar\ Trz}$), 118.22, 118.17, 118.14, 118.03, 118.00, 117.71 (CH_{Ar}), 76.62, 76.43 ($O\underline{C}H_2CH_2$), 65.84 (OCH_2Trz), 52.56 (NCH_2), 31.07, 30.64 ($ArCH_2Ar$), 22.89, 22.54 ($\underline{C}H_2CH_3$), 20.33 ($PhCH_3$), 10.37, 10.08 ($CH_2\underline{C}H_3$) ppm. ESI-MS *m*/*z*: 1314.6502 $[M+H]^+$ for $C_{79}H_{84}N_{11}O_8$ (1314.6499), 657.8280 $[M+2H]^{2+}$ for $C_{79}H_{85}N_{11}O_8$ (657.8286).

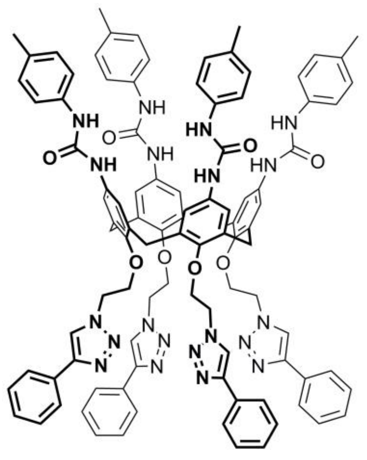

Tetratriazolated tetraureacalix[4]arene ***50*** was prepared as described for compound **47** from calixarene **45** (0.026 g, 0.022 mmol) and *p*-tolyl isocyanate (0.034 mL, 0.270 mmol) in dry toluene (3 mL). Yield 0.025 g (66%), white solid. M.p. 213–215 °C. ^1H NMR (400 MHz, DMSO-d_6): δ = 8.51 (s, 4H; ArH_{Trz}), 8.22 (s, 4H; NH), 8.19 (s, 4H; NH), 7.79–7.73 (m, 8H; ArH_{Ph}), 7.38–7.31 (m, 8H; ArH_{Ph}), 7.30–7.24 (m, 4H; ArH_{Ph}), 7.23–7.18 (m, 8H; ArH_{Tol}), 7.05–6.99 (m, 8H; ArH_{Tol}), 6.79 (s, 8H; ArH), 4.91–4.83 (m, 8H; OCH_2CH_2), 4.32–4.24 (m, 8H; OCH_2CH_2), 4.07 (d, 4H, ^2J = 13.2 Hz; $ArCH_2Ar$), 3.06 (d, 4H, ^2J = 13.2 Hz; $ArCH_2Ar$), 2.21 (s, 12H; $PhCH_3$) ppm. ^{13}C NMR (100 MHz, DMSO-d_6): δ = 152.40 (C=O), 149.82 (C_{Ar}), 146.50 ($C_{Ar\ Trz}$), 137.18, 134.39, 134.02, 130.66, 130.32 (C_{Ar}), 129.06, 128.81, 127.78, 125.10 (CH_{Ar}), 121.51 ($CH_{Ar\ Trz}$), 118.15, 118.05 (CH_{Ar}), 72.32 (OCH_2), 49.50 (NCH_2), 30.29 ($ArCH_2Ar$), 20.32 ($PhCH_3$) ppm. ESI-MS *m*/*z*: 851.8789 $[M+2H]^{2+}$ for $C_{100}H_{94}N_{20}O_8$ (851.8793).

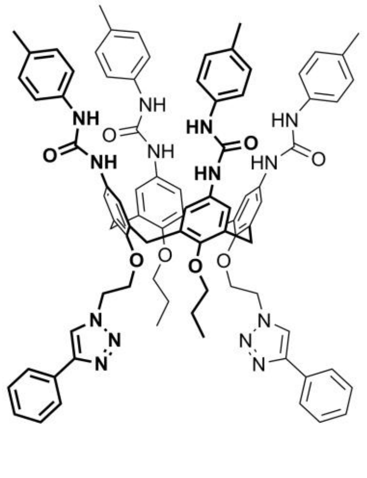

Ditriazolated tetraureacalix[4]arene ***51*** was prepared as described for compound **47** from calixarene **46** (0.041 g, 0.045 mmol) and *p*-tolyl isocyanate (0.068 mL, 0.540 mmol) in dry toluene (4.5 mL). Yield 0.056 g (86%), white solid. M.p. 223–225 °C. ^1H NMR (400 MHz, DMSO-d_6): δ = 8.68 (s, 2H; ArH_{Trz}), 8.38 (s, 2H; NH), 8.36 (s, 2H; NH), 8.13 (s, 2H; NH), 8.06 (s, 2H; NH), 7.90–7.85 (m, 4H; ArH_{Ph}), 7.48–7.42 (m, 4H; ArH_{Ph}), 7.37–7.31 (m, 2H;

ArH_{Ph}), 7.31–7.26 (m, 4H; ArH_{Tol}), 7.19–7.14 (m, 4H; ArH_{Tol}), 7.08–7.03 (m, 4H; ArH_{Tol}), 7.06 (s, 4H; ArH), 7.02–6.97 (m, 4H; ArH_{Tol}), 6.64 (s, 4H; ArH), 5.05–4.98 (m, 4H; NCH_2CH_2), 4.56–4.49 (m, 4H; NCH_2CH_2), 4.27 (d, 4H, ^2J = 13.0 Hz; $ArCH_2Ar$), 3.63–3.57 (m, 4H; OCH_2), 3.16 (d, 4H, ^2J = 13.0 Hz; $ArCH_2Ar$), 2.23 (s, 6H; $PhCH_3$), 2.20 (s, 6H; $PhCH_3$), 1.80–1.68 (m, 4H; $C\underline{H}_2CH_3$), 0.80 (t, 6H, ^3J = 7.4 Hz; $CH_2C\underline{H}_3$) ppm. ^{13}C NMR (100 MHz, DMSO-d_6): δ = 152.54, 152.38 (C=O), 150.52, 150.18 (C_{Ar}), 146.44 ($C_{Ar\ Trz}$), 137.26, 137.22, 135.10, 134.62, 133.52, 133.41, 130.80, 130.39, 130.22 (C_{Ar}), 129.12, 129.04, 128.91, 127.88, 125.14 (CH_{Ar}), 121.53 ($CH_{Ar\ Trz}$), 118.39, 118.13, 118.05, 117.96 (CH_{Ar}), 77.01, 71.73 (OCH_2), 49.16 (NCH_2), 30.70 ($ArCH_2Ar$), 22.64 ($\underline{C}H_2CH_3$), 20.34, 20.31 ($PhCH_3$), 10.26 ($CH_2\underline{C}H_3$) ppm. ESI-MS *m*/*z*: 1465.6641 $[M+Na]^+$ for $C_{86}H_{86}NaN_{14}O_8$ (1465.6645).

NMR spectra of novel compounds

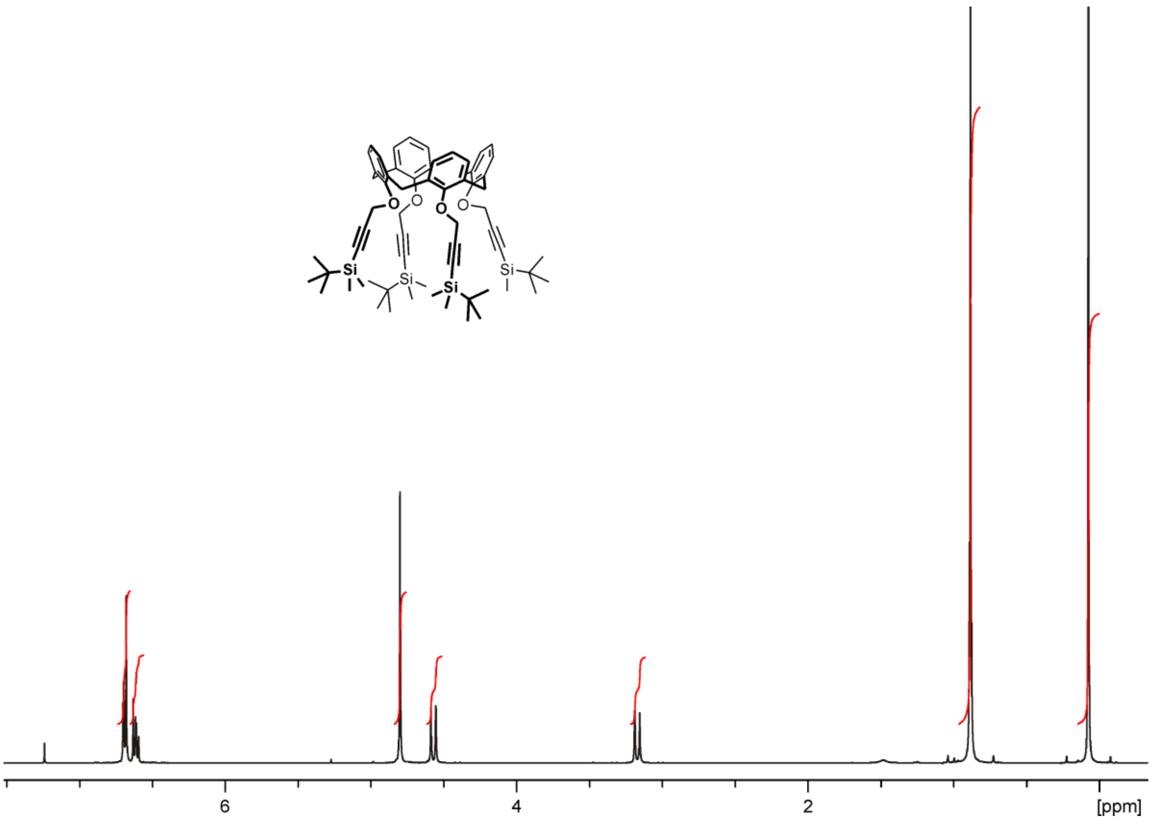

Figure S1. ^{1}H NMR spectrum of calixarene **7** (400 MHz, $CDCl_3$).

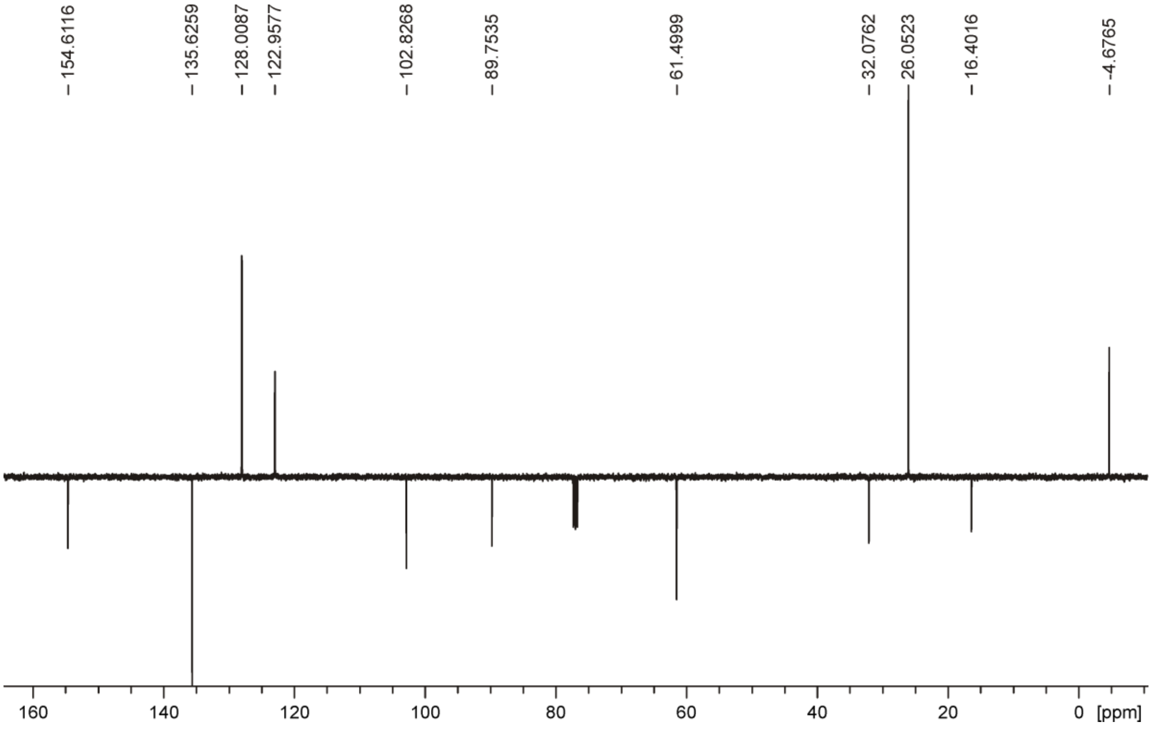

Figure S2. ^{13}C NMR spectrum (APT) of calixarene **7** (100 MHz, $CDCl_3$).

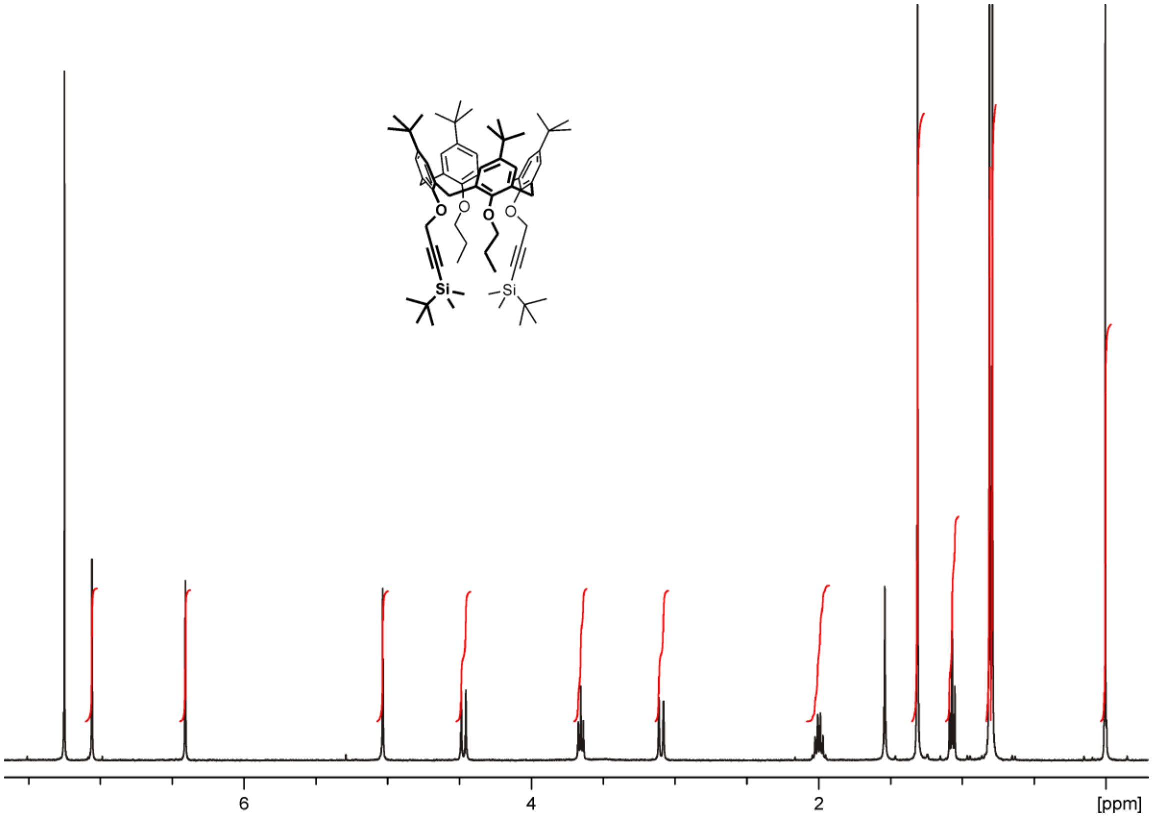

Figure S3. ^{1}H NMR spectrum of calixarene **8** (400 MHz, $CDCl_3$).

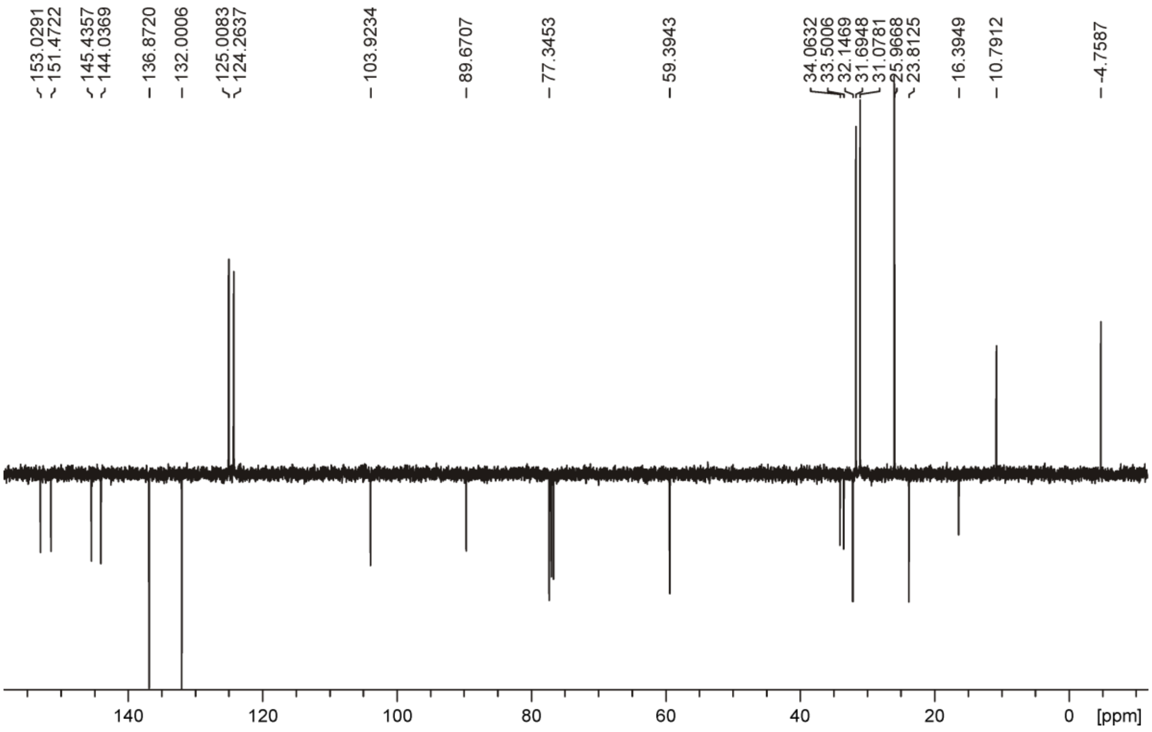

Figure S4. ^{13}C NMR spectrum (APT) of calixarene **8** (100 MHz, $CDCl_3$).

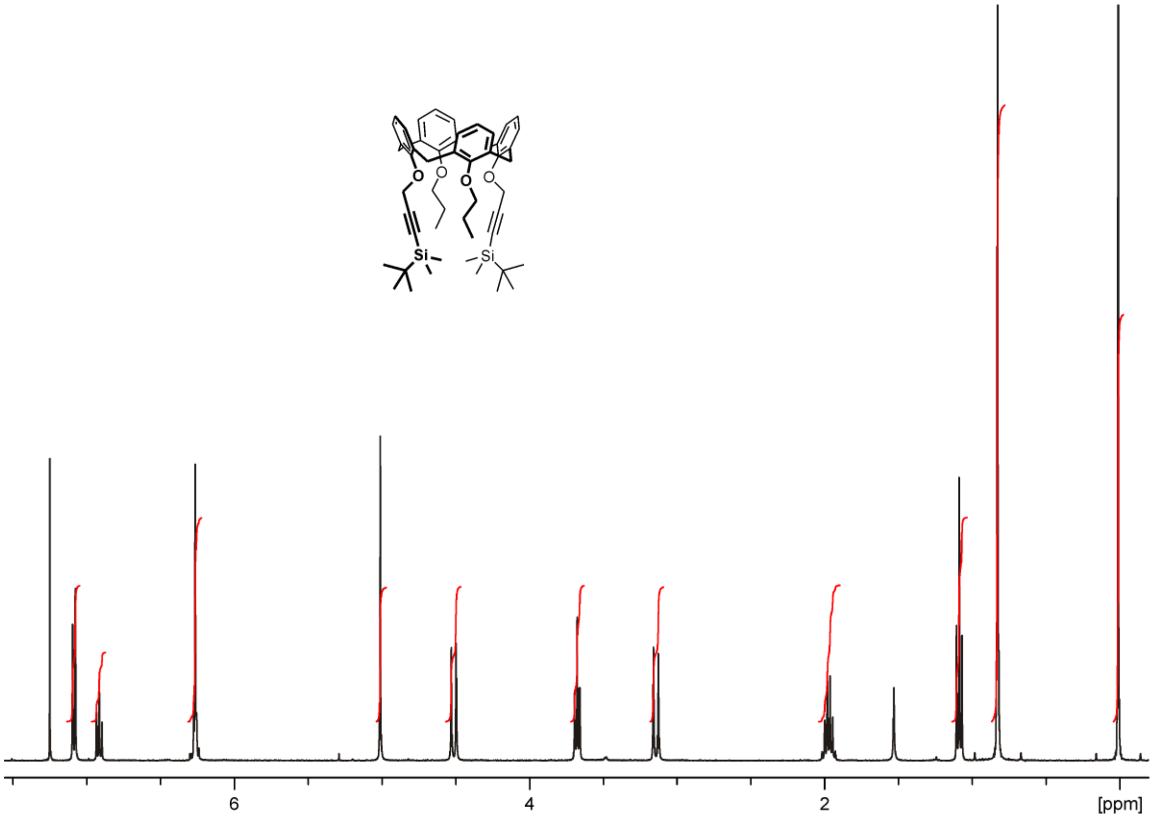

Figure S5. ^{1}H NMR spectrum of calixarene **9** (400 MHz, $CDCl_3$).

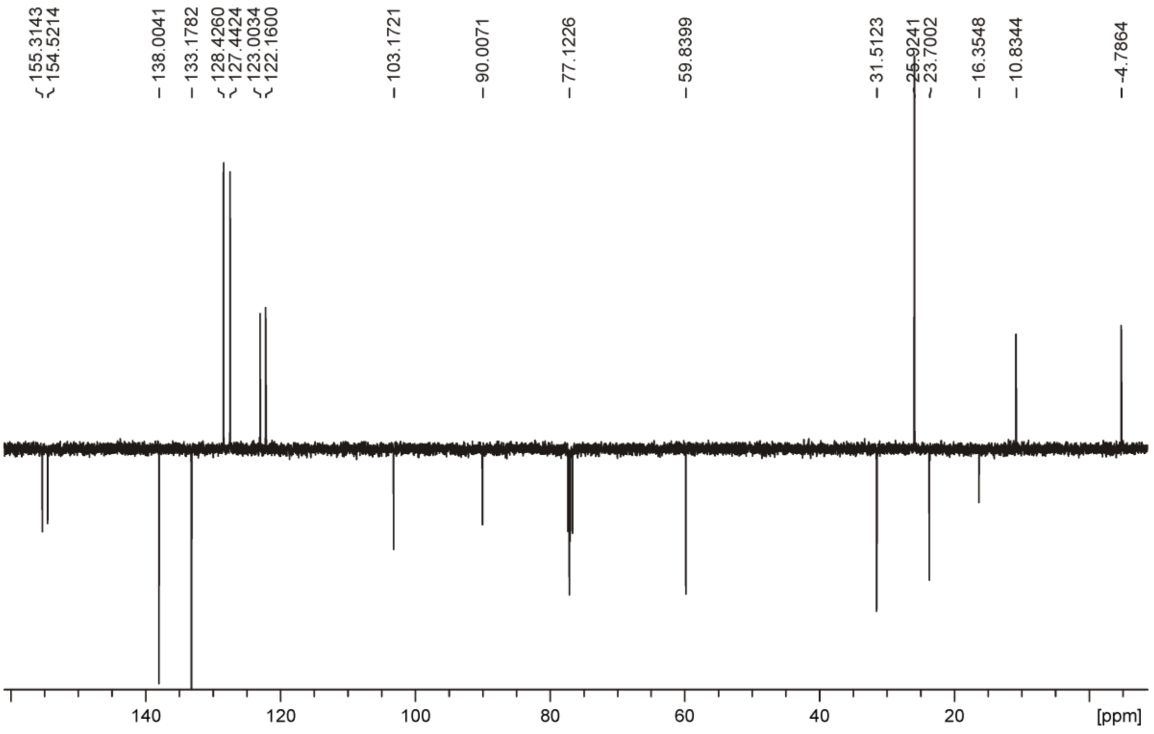

Figure S6. ^{13}C NMR spectrum (APT) of calixarene **9** (100 MHz, $CDCl_3$).

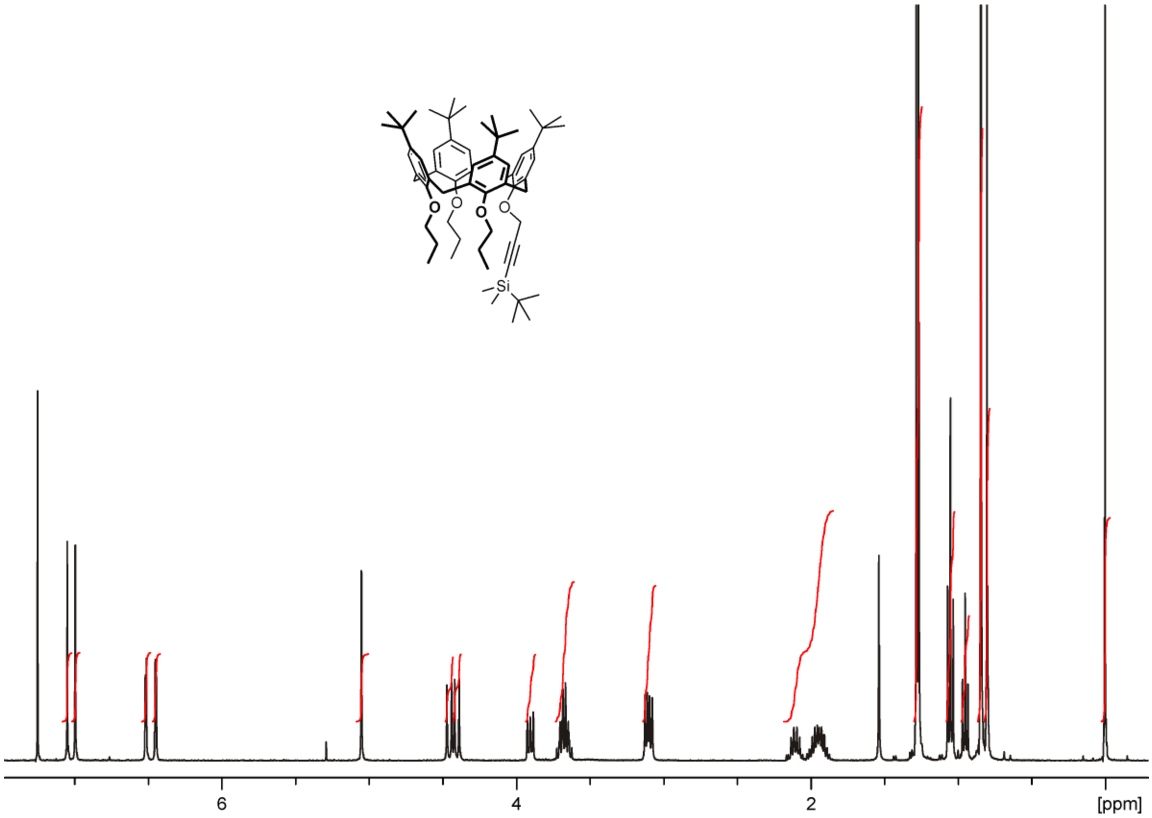

Figure S7. ^{1}H NMR spectrum of calixarene **10** (400 MHz, $CDCl_3$).

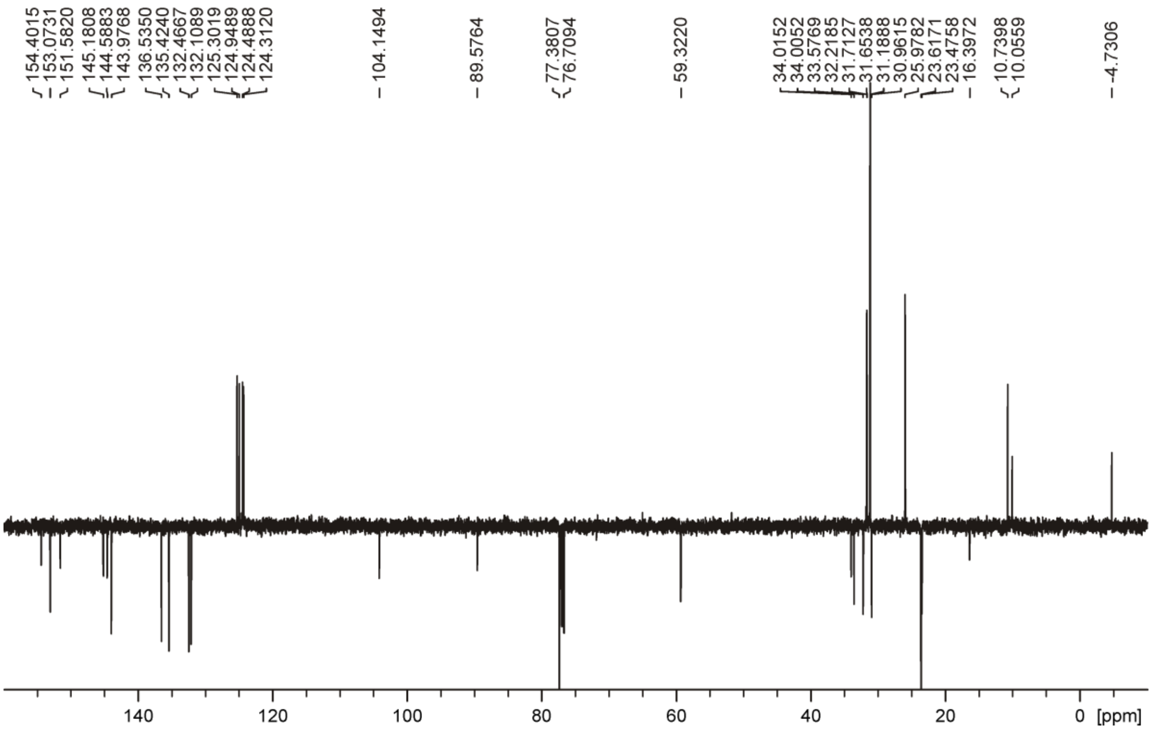

Figure S8. ^{13}C NMR spectrum (APT) of calixarene **10** (100 MHz, $CDCl_3$).

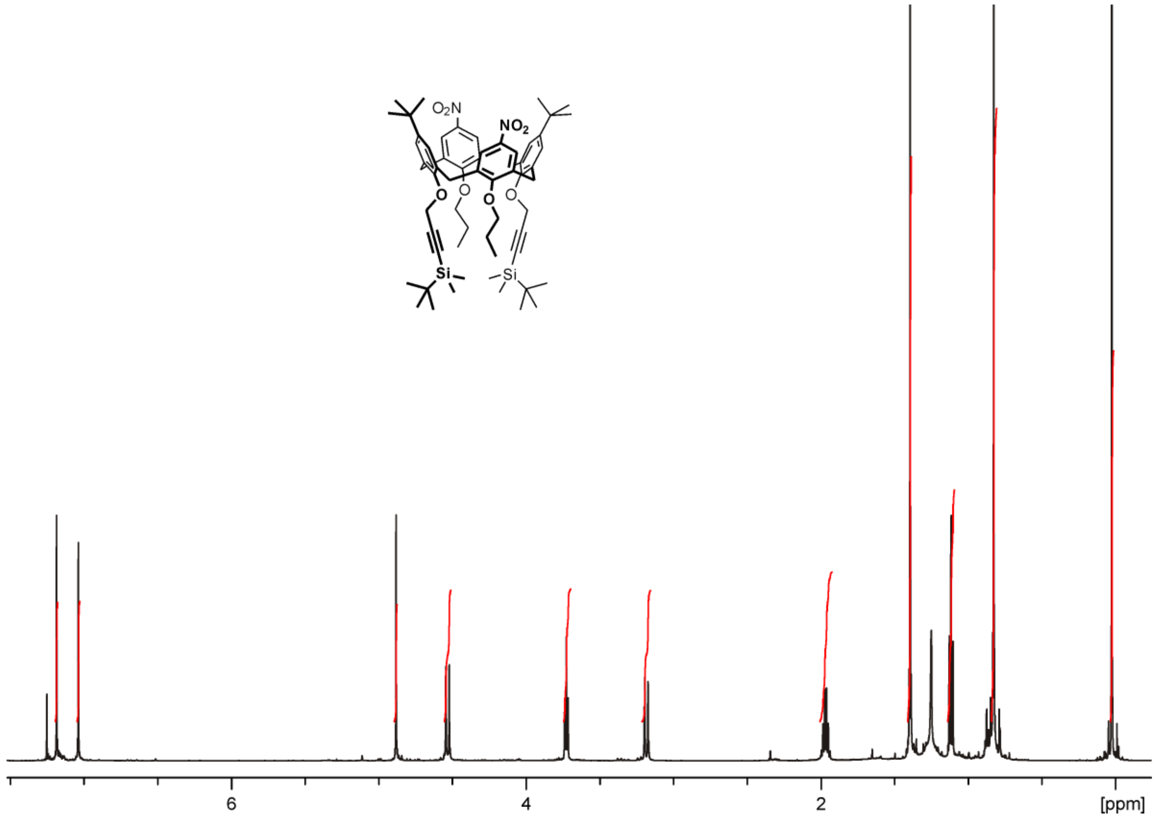

Figure S9. ^{1}H NMR spectrum of calixarene **12** (600 MHz, $CDCl_3$).

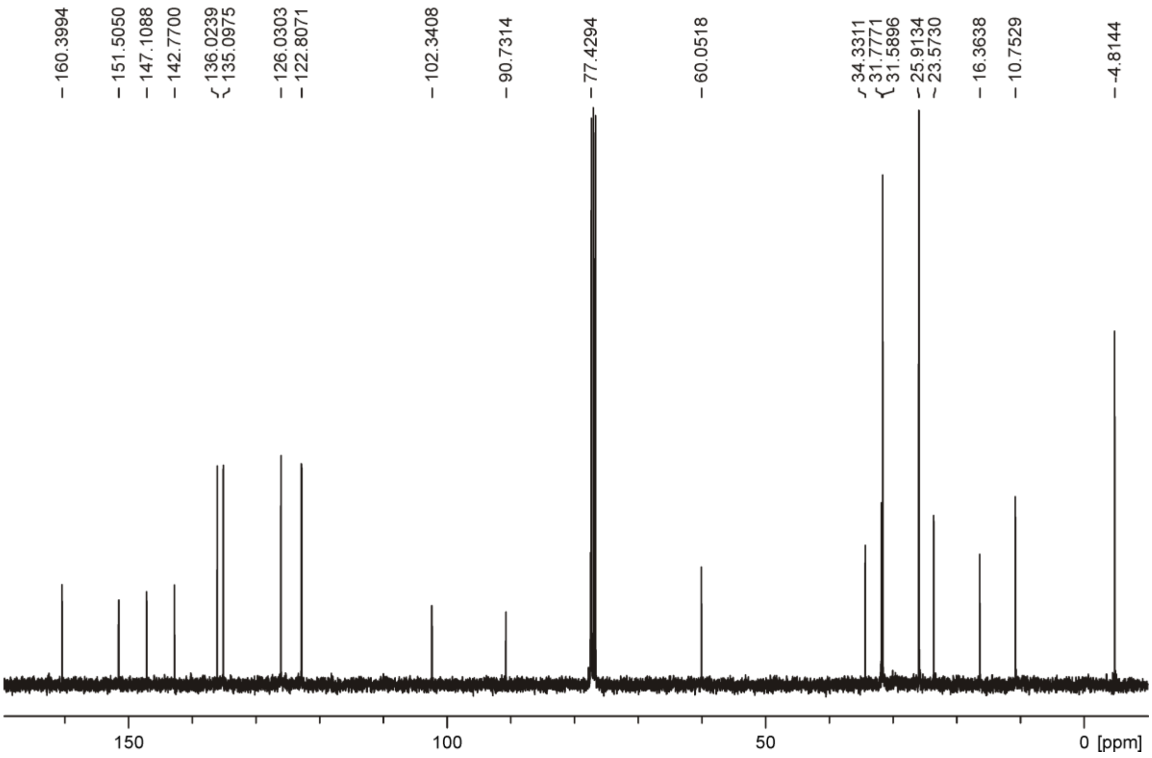

Figure S10. ^{13}C NMR spectrum of calixarene **12** (150 MHz, $CDCl_3$).

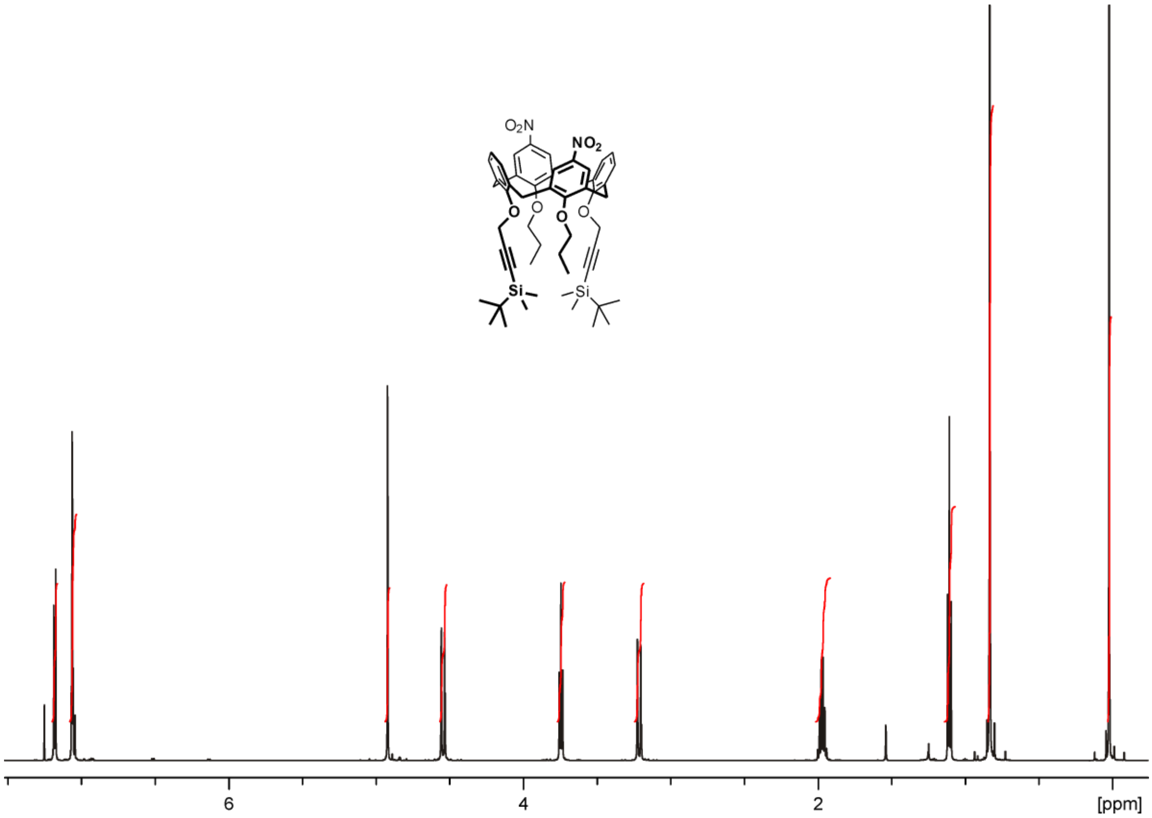

Figure S11. ^{1}H NMR spectrum of calixarene **13** (600 MHz, $CDCl_3$).

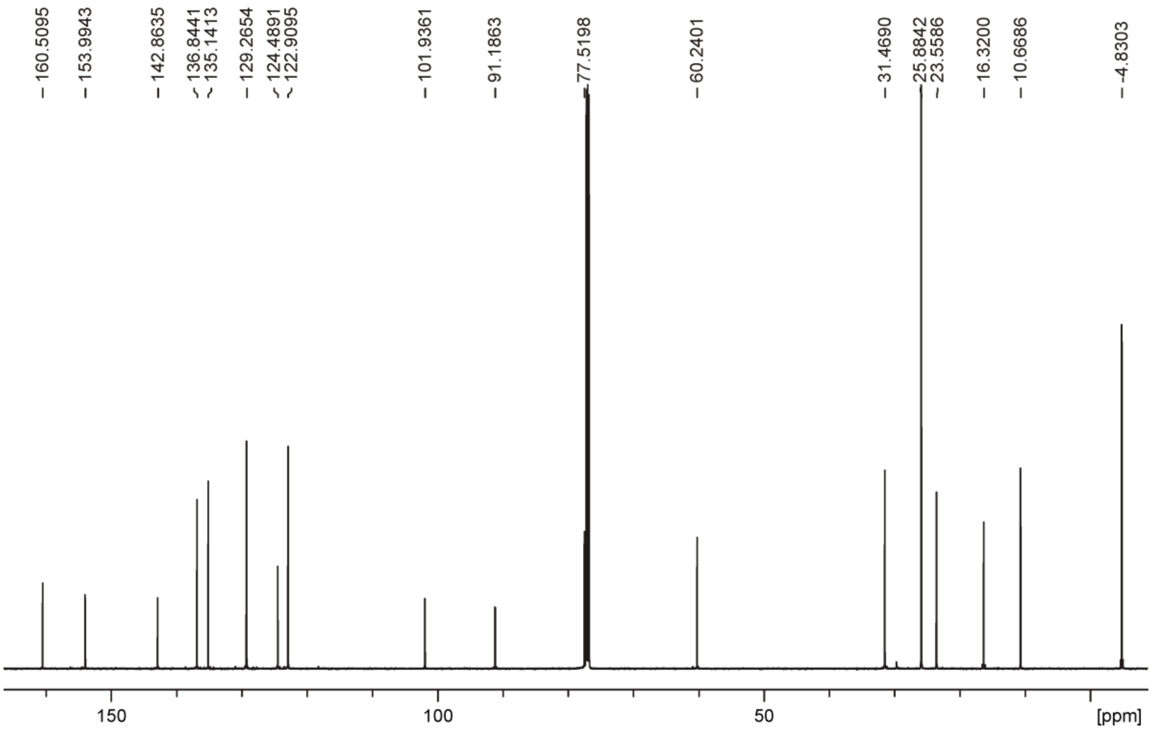

Figure S12. ^{13}C NMR spectrum of calixarene **13** (150 MHz, $CDCl_3$).

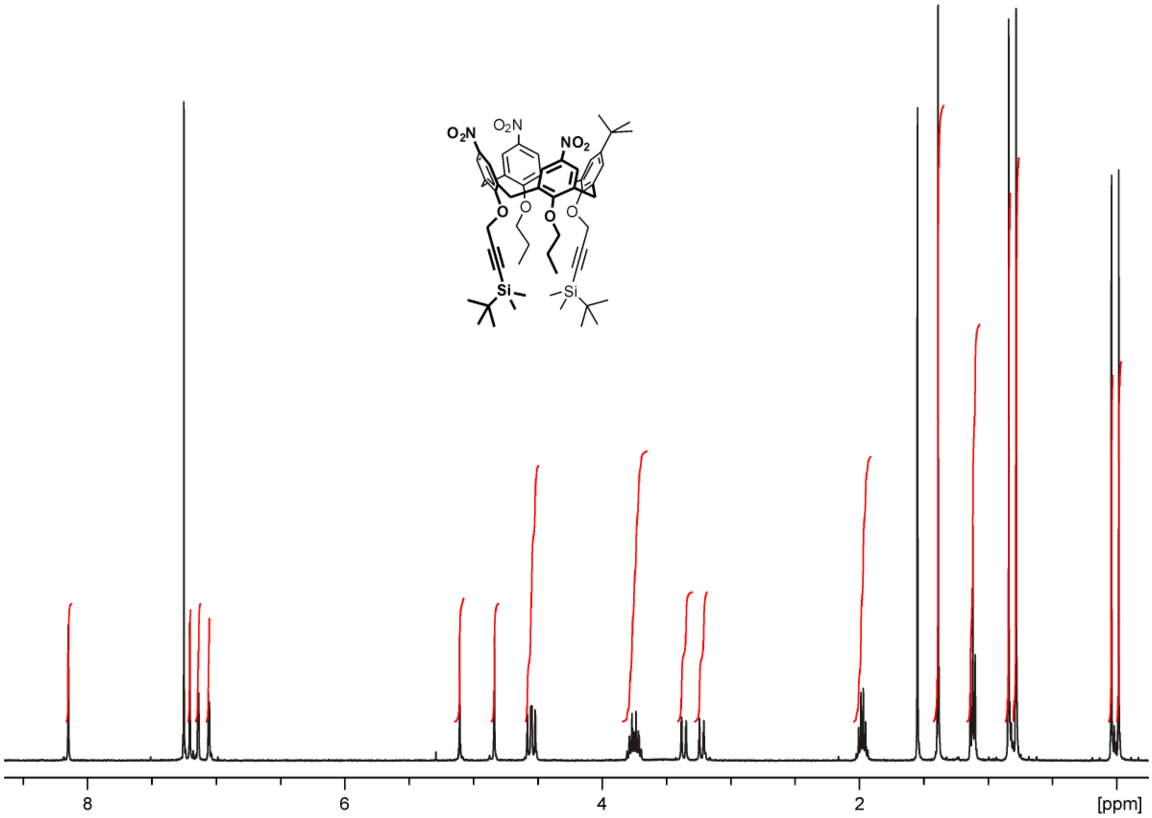

Figure S13. ^{1}H NMR spectrum of calixarene **14** (400 MHz, $CDCl_3$).

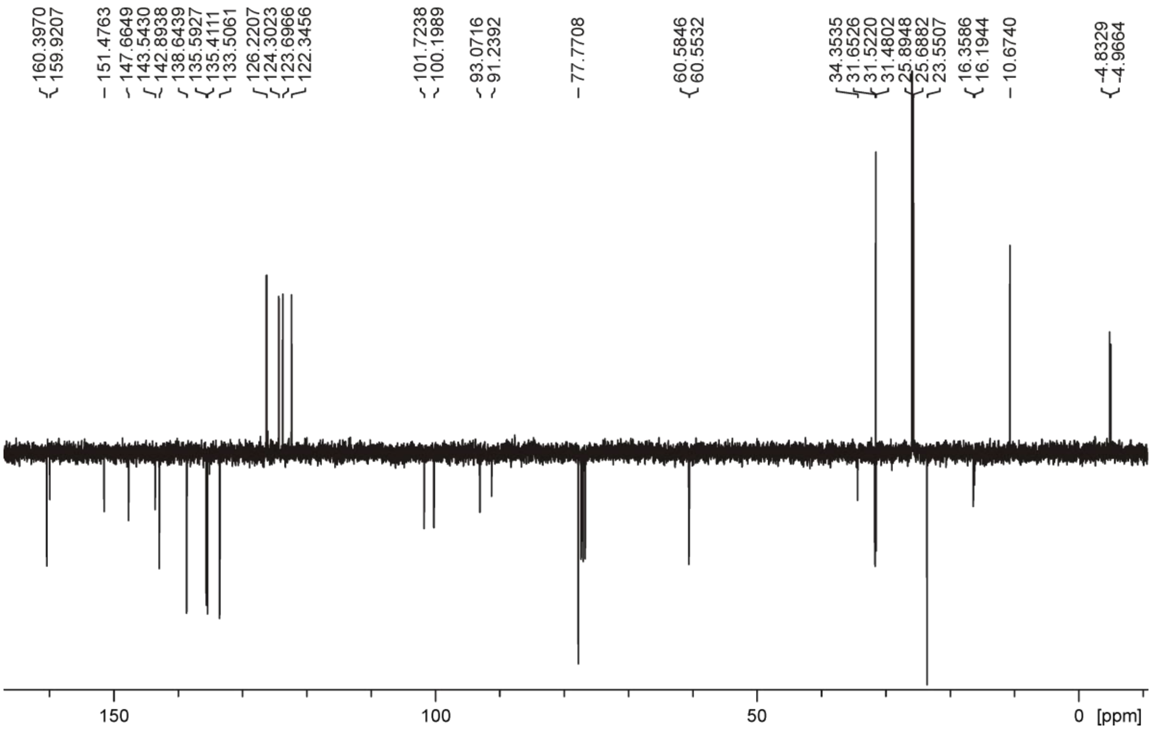

Figure S14. ^{13}C NMR spectrum (APT) of calixarene **14** (100 MHz, $CDCl_3$).

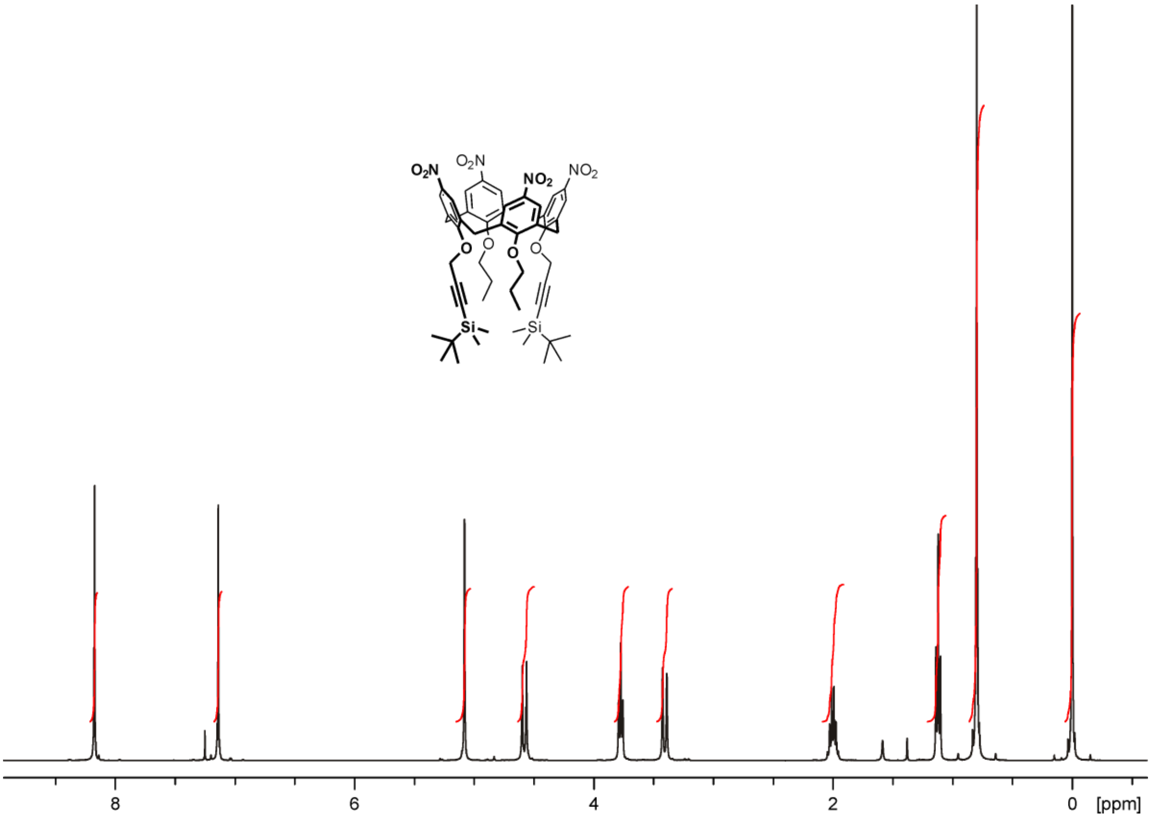

Figure S15. ^{1}H NMR spectrum of calixarene **15** (400 MHz, $CDCl_3$).

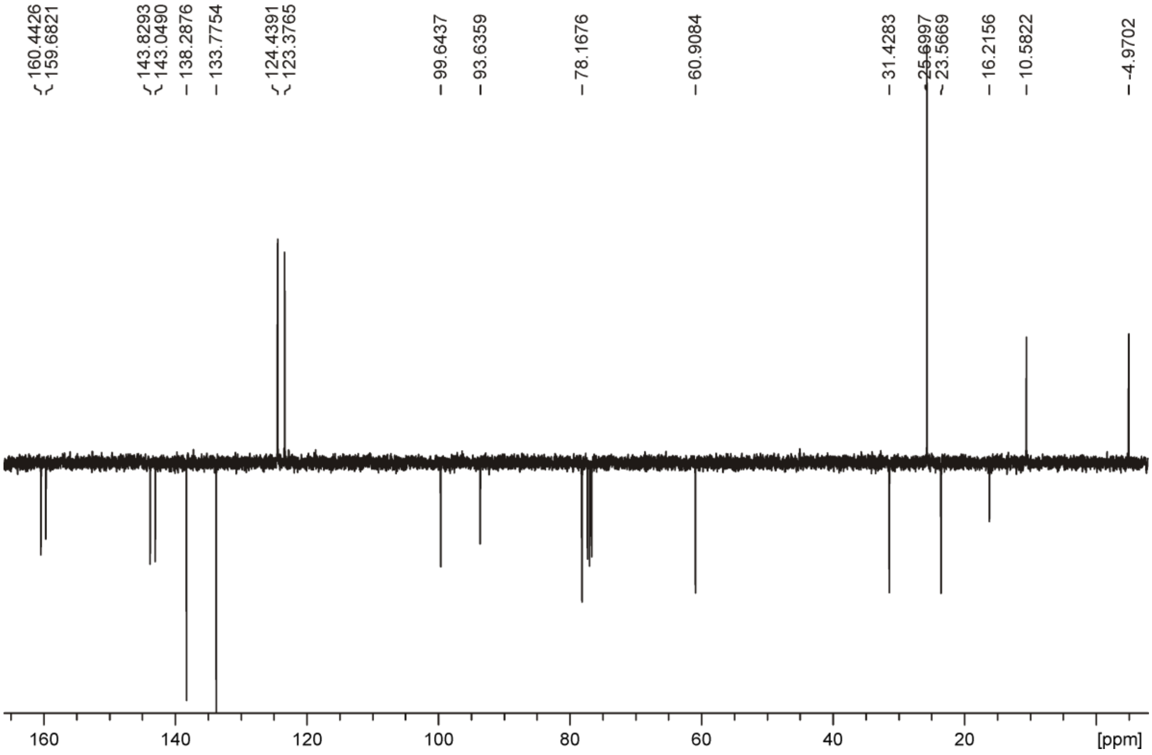

Figure S16. ^{13}C NMR spectrum (APT) of calixarene **15** (100 MHz, $CDCl_3$).

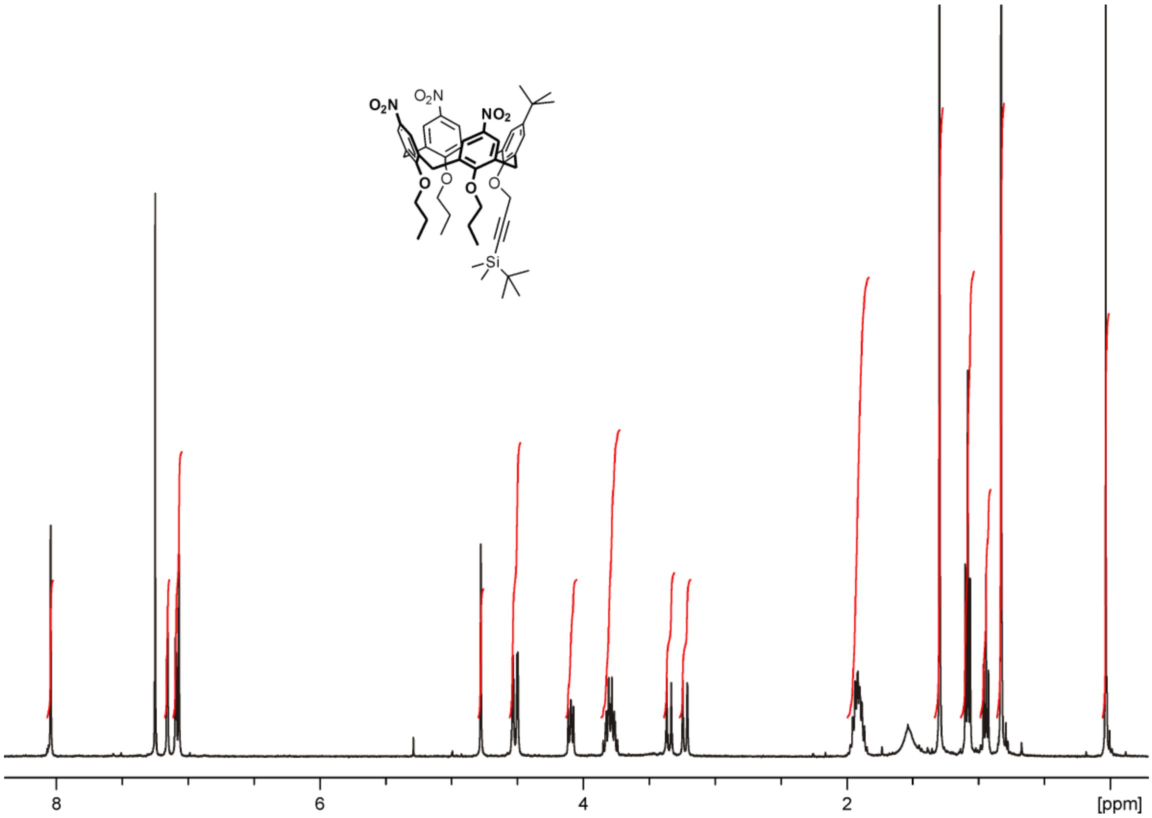

Figure S17. ^{1}H NMR spectrum of calixarene **16** (400 MHz, $CDCl_3$).

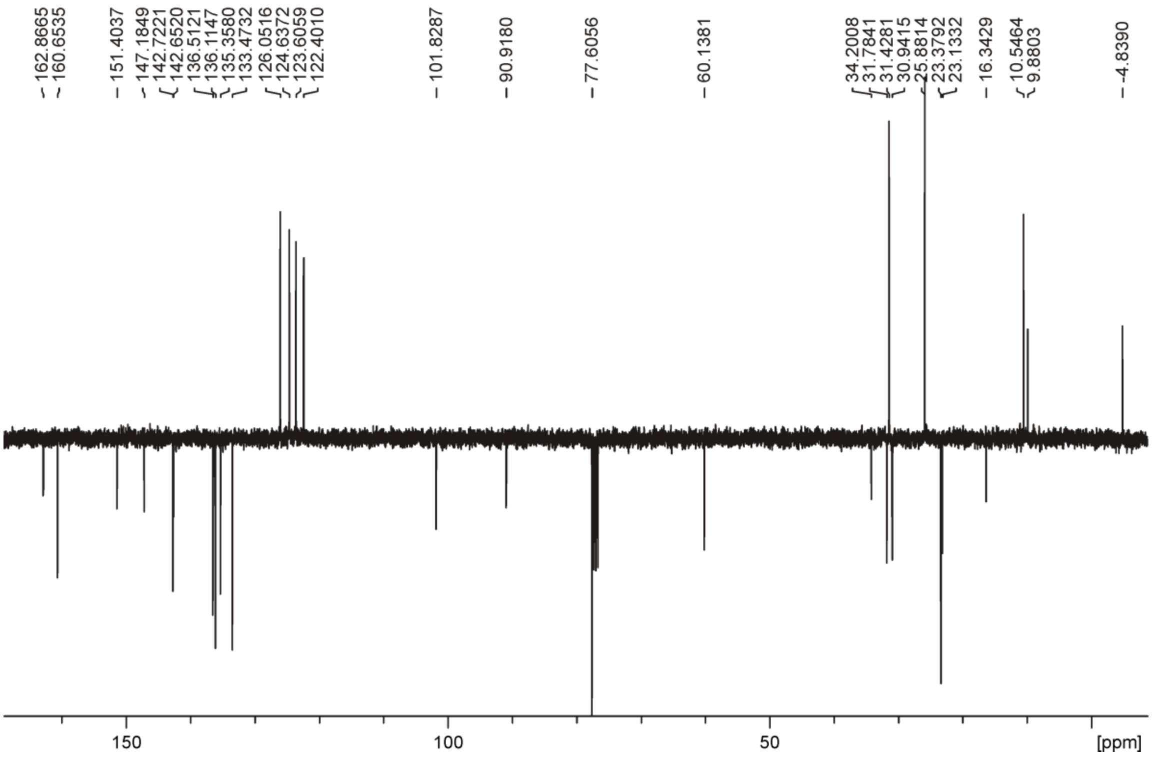

Figure S18. ^{13}C NMR spectrum (APT) of calixarene **16** (100 MHz, $CDCl_3$).

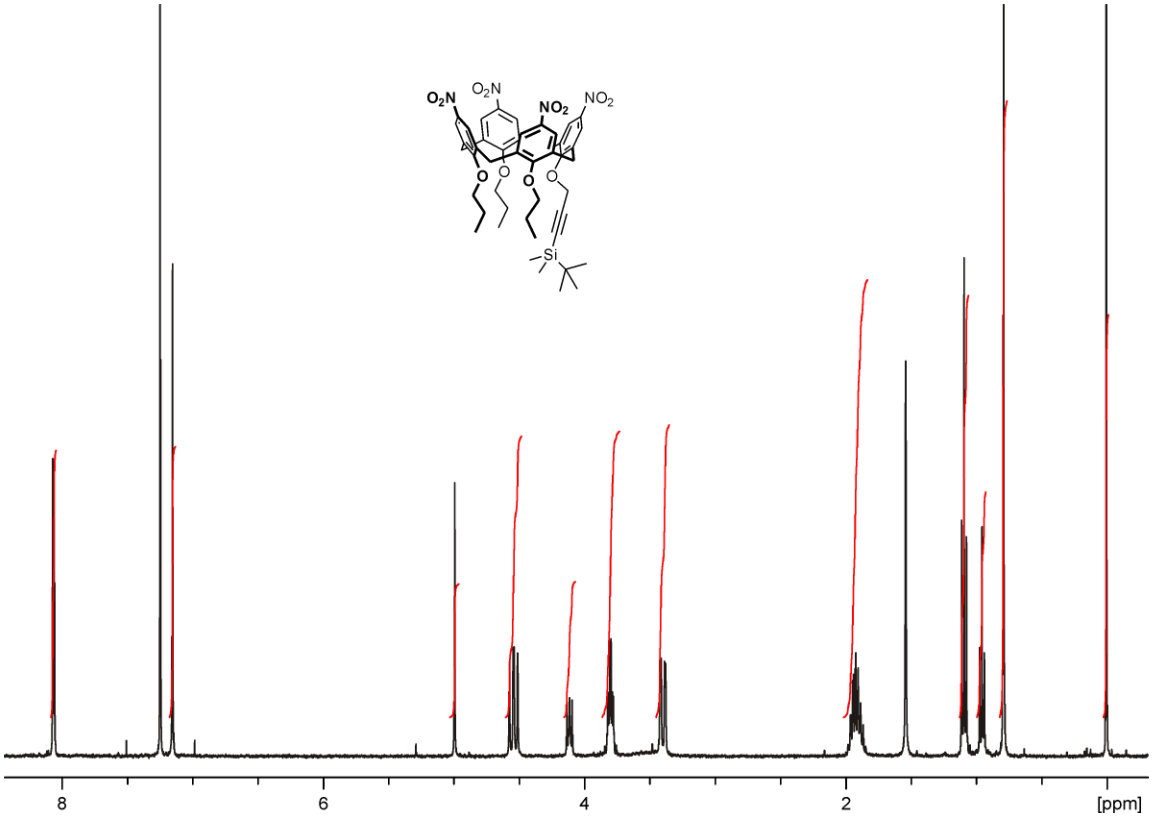

Figure S19. ¹H NMR spectrum of calixarene **17** (400 MHz, $CDCl_3$).

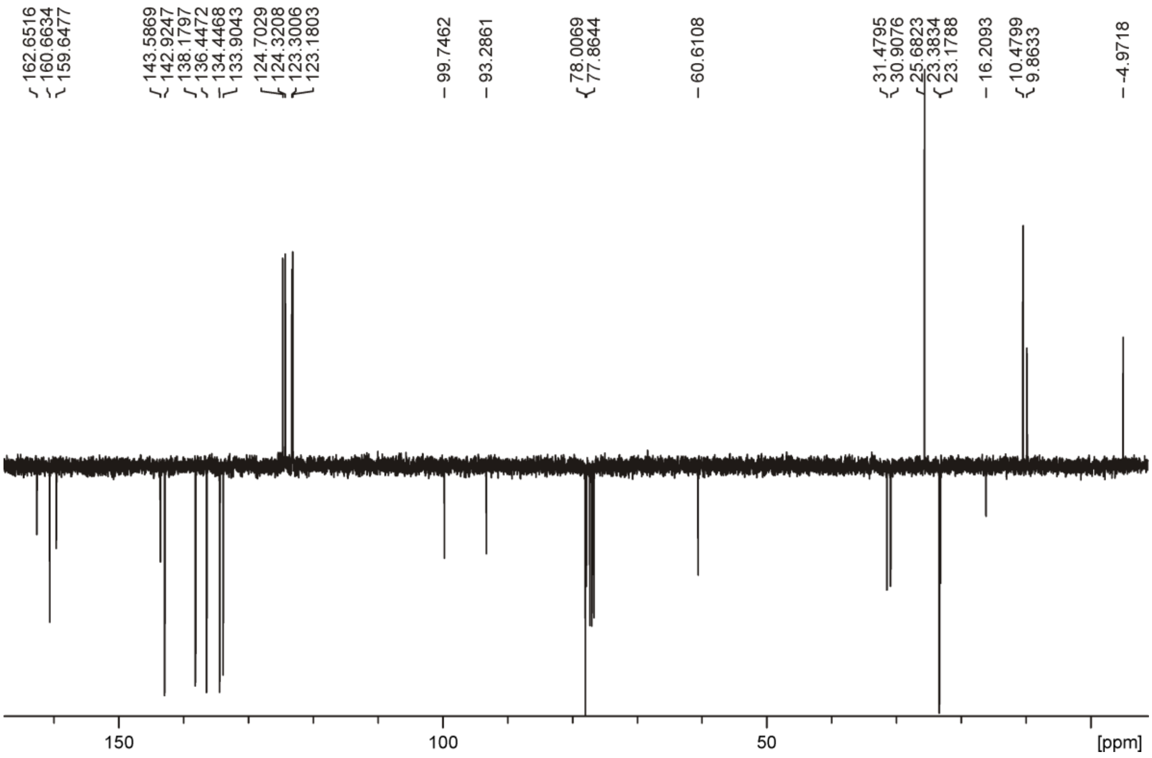

Figure S20. ¹³C NMR spectrum (APT) of calixarene **17** (100 MHz, $CDCl_3$).

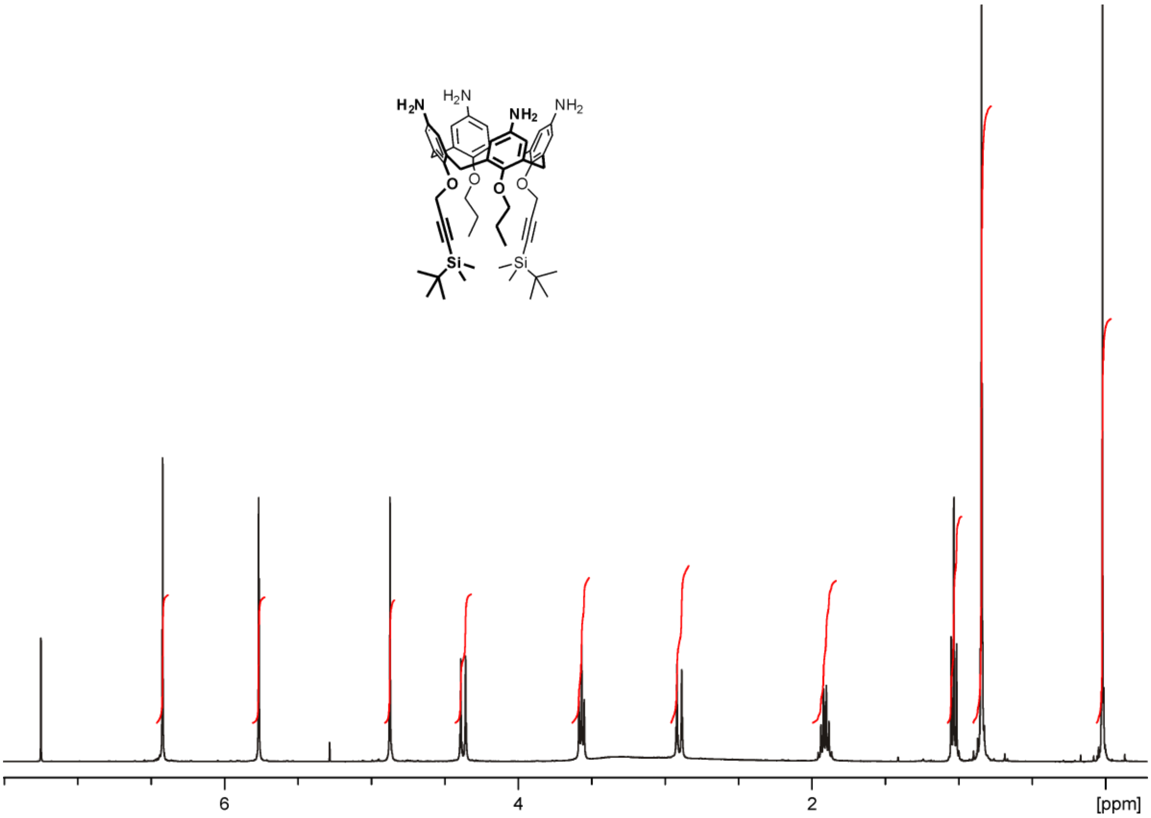

Figure S21. ^{1}H NMR spectrum of calixarene **19** (400 MHz, $CDCl_3$).

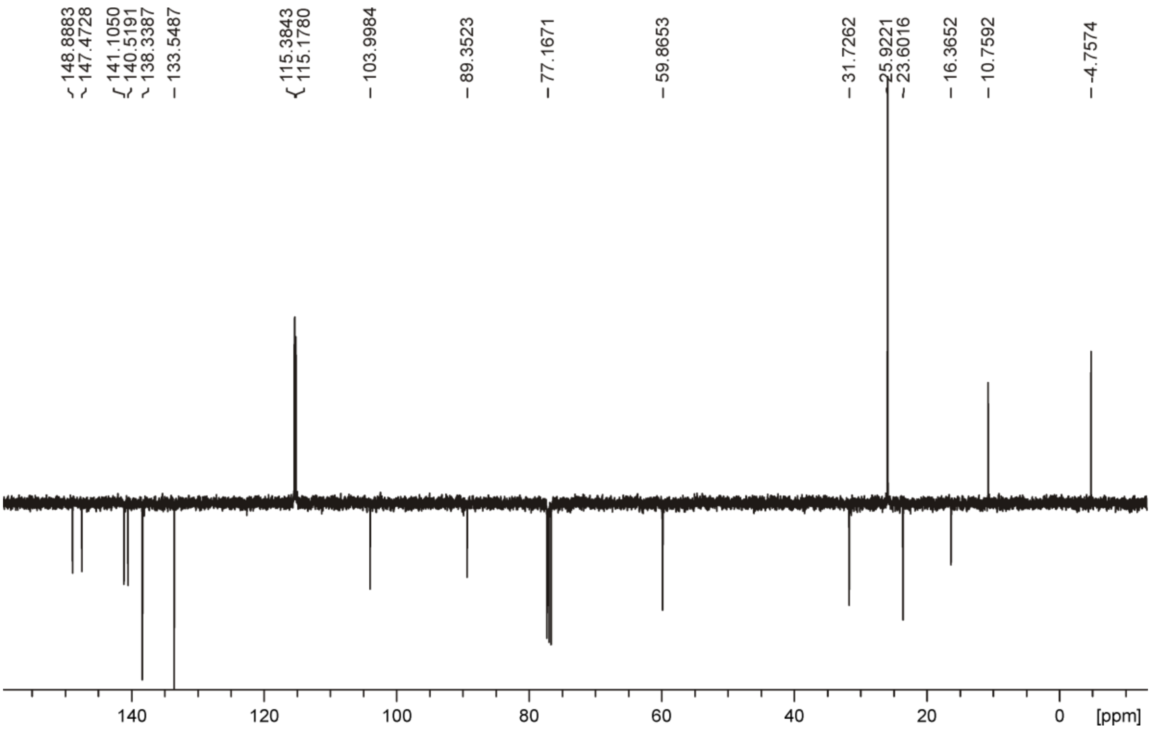

Figure S22. ^{13}C NMR spectrum (APT) of calixarene **19** (100 MHz, $CDCl_3$).

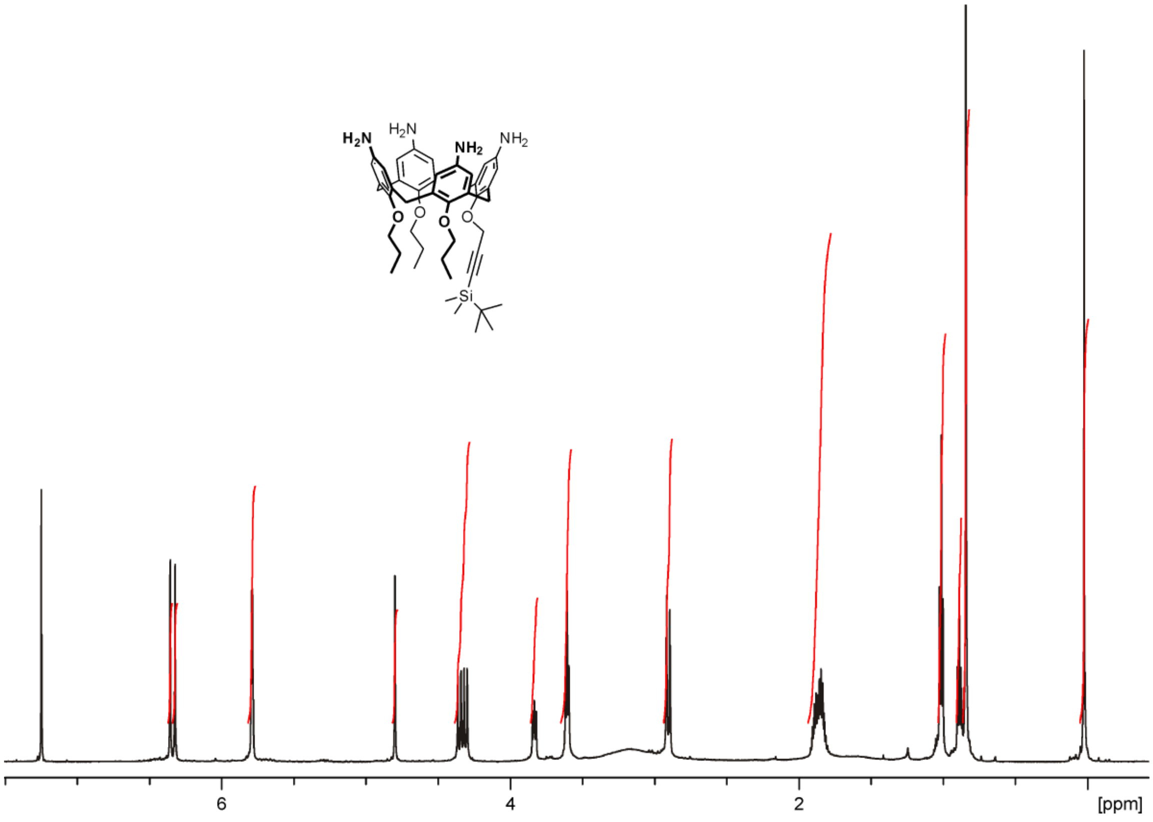

Figure S23. ^{1}H NMR spectrum of calixarene **20** (400 MHz, $CDCl_3$).

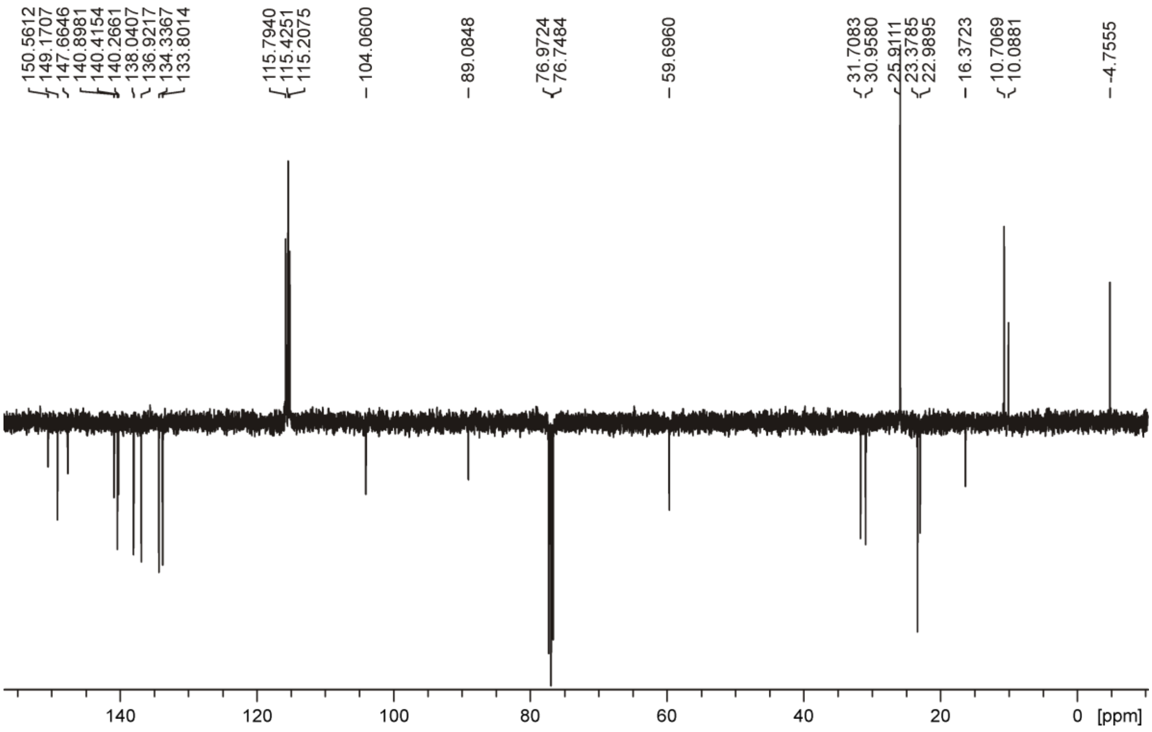

Figure S24. ^{13}C NMR spectrum (APT) of calixarene **20** (100 MHz, $CDCl_3$).

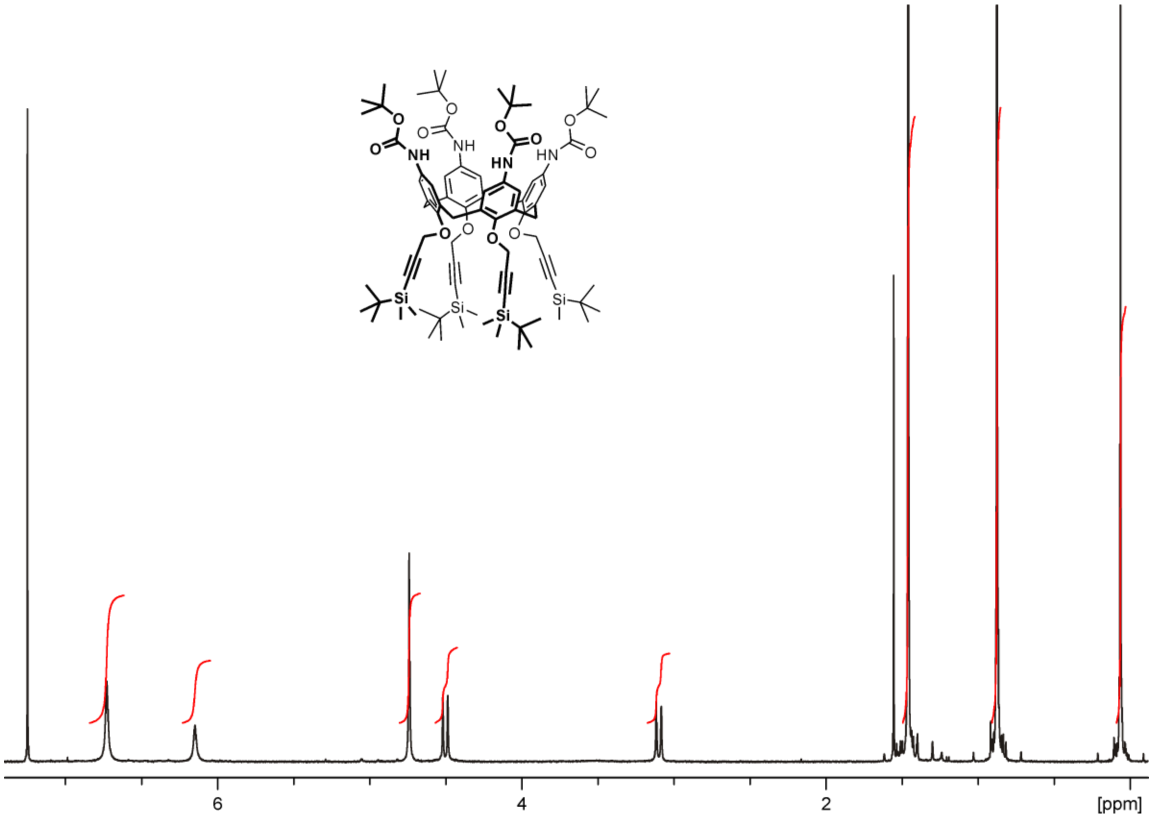

Figure S25. ^{1}H NMR spectrum of calixarene **21** (400 MHz, $CDCl_3$).

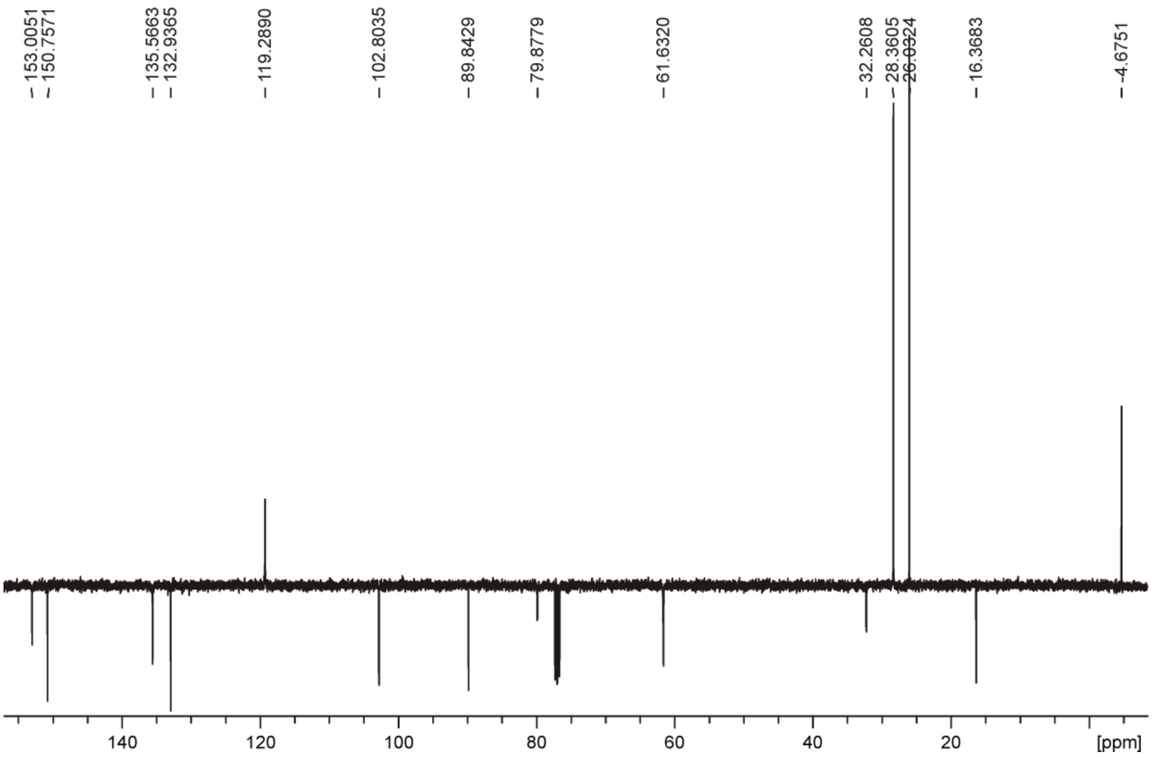

Figure S26. ^{13}C NMR spectrum (APT) of calixarene **21** (100 MHz, $CDCl_3$).

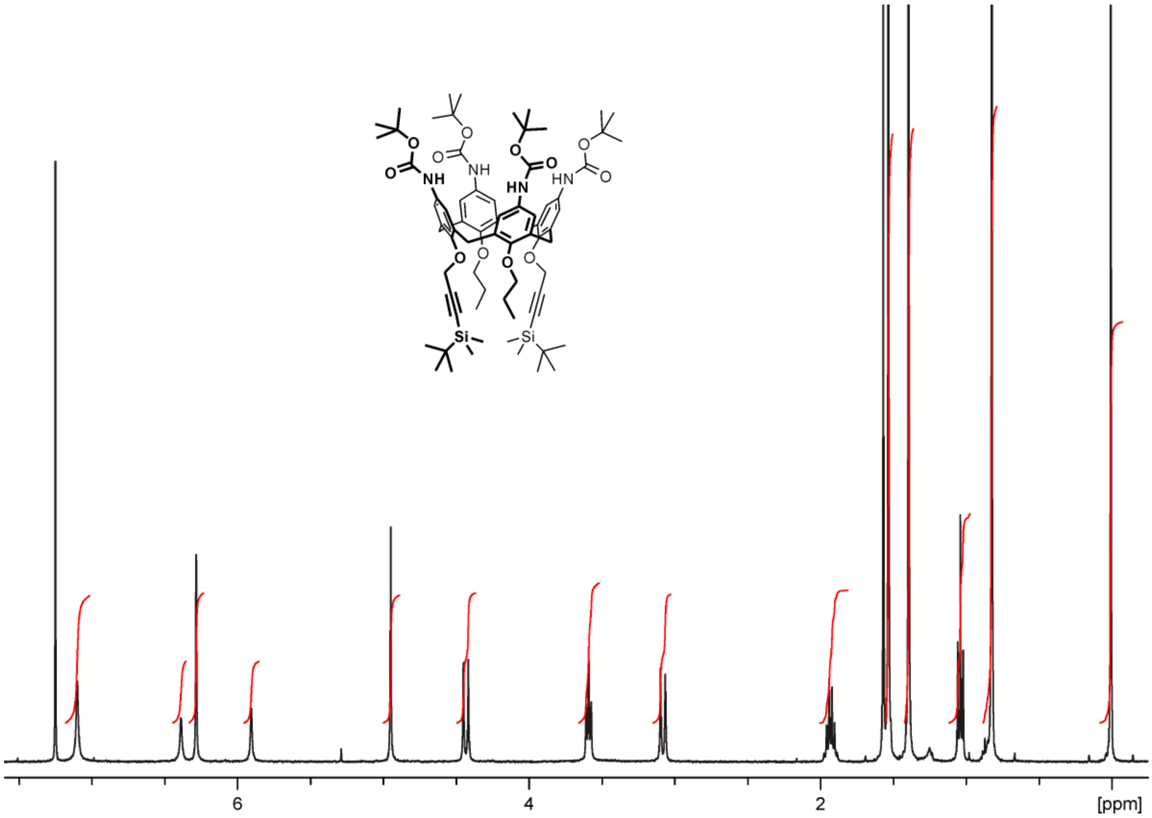

Figure S27. ^{1}H NMR spectrum of calixarene **22** (400 MHz, $CDCl_3$).

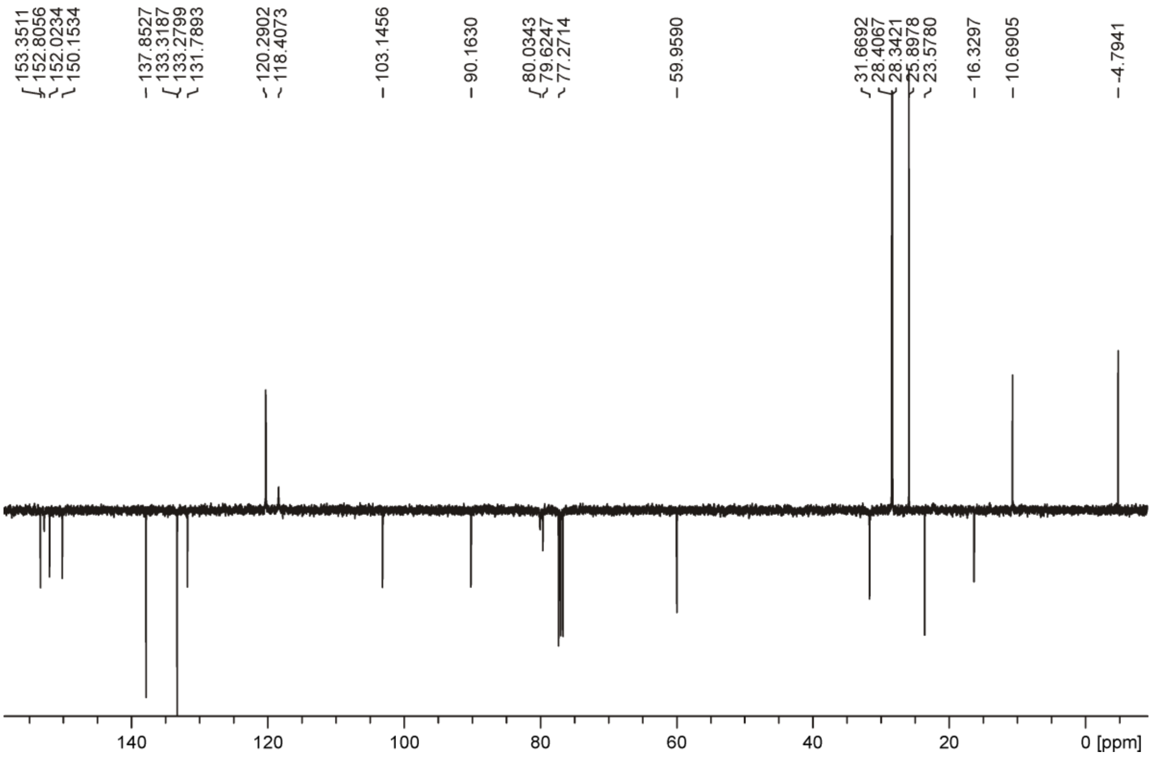

Figure S28. ^{13}C NMR spectrum (APT) of calixarene **22** (100 MHz, $CDCl_3$).

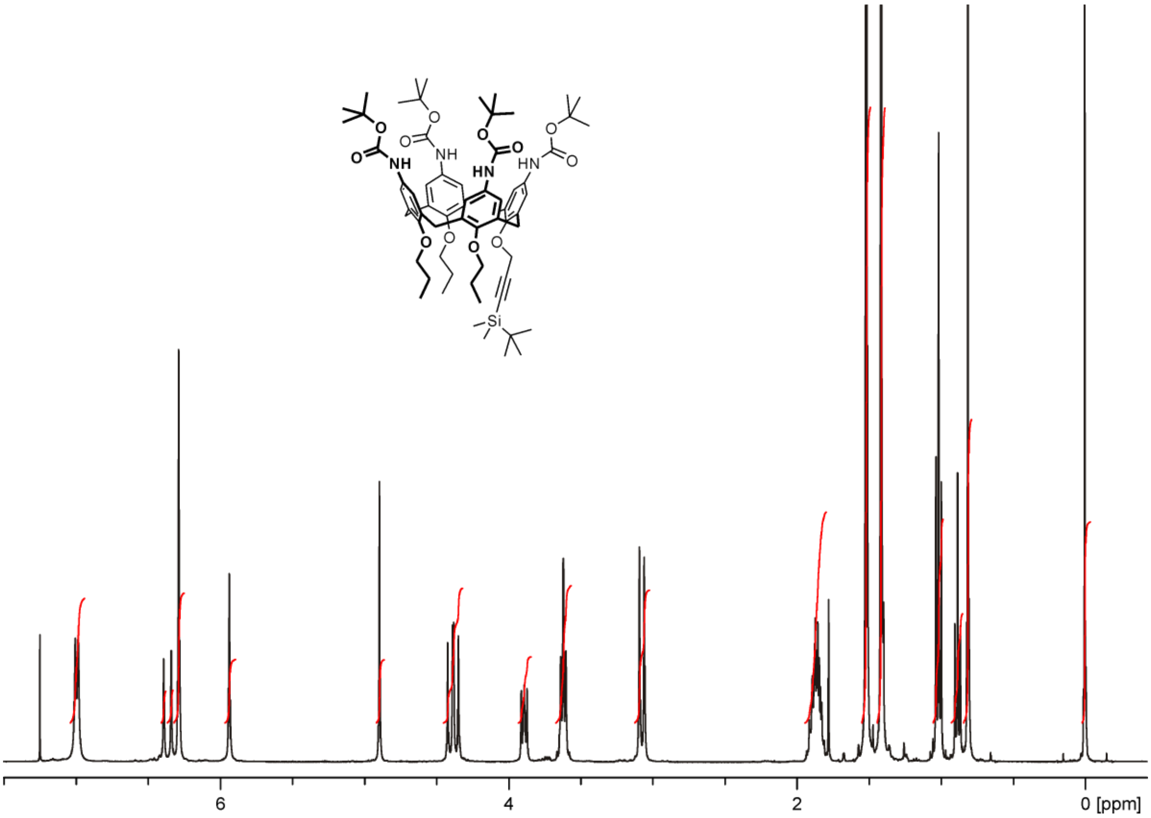

Figure S29. ^{1}H NMR spectrum of calixarene **23** (400 MHz, $CDCl_3$).

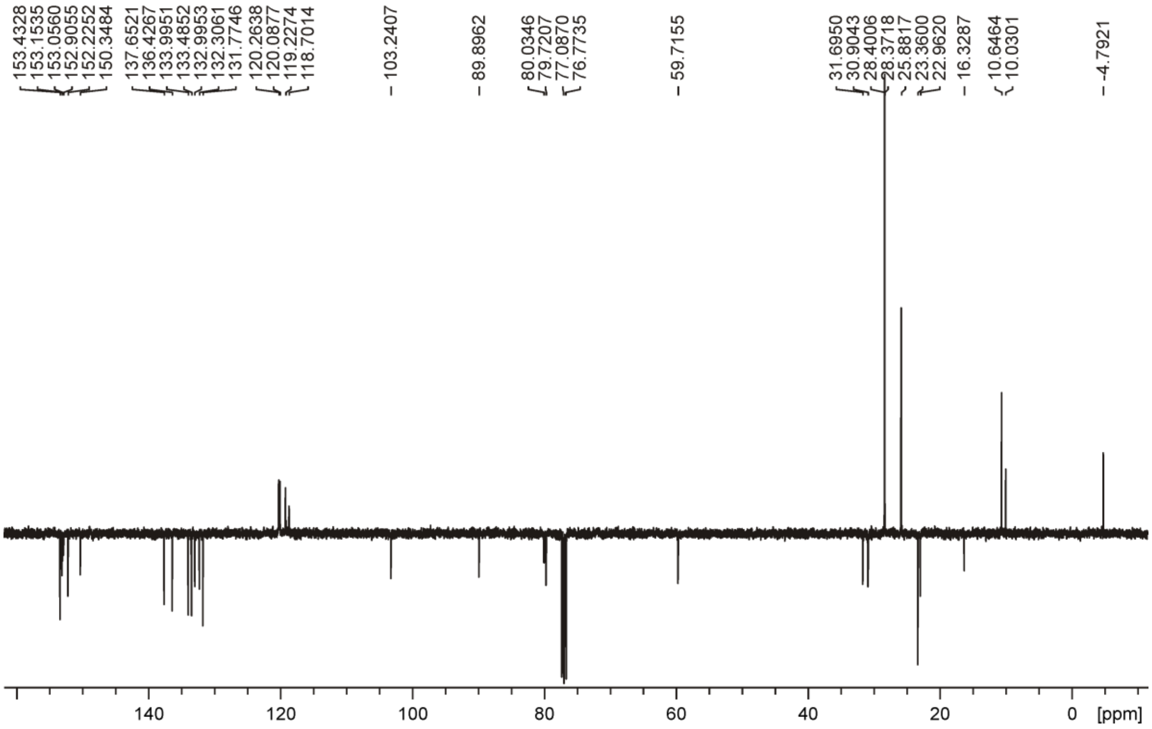

Figure S30. ^{13}C NMR spectrum (APT) of calixarene **23** (100 MHz, $CDCl_3$).

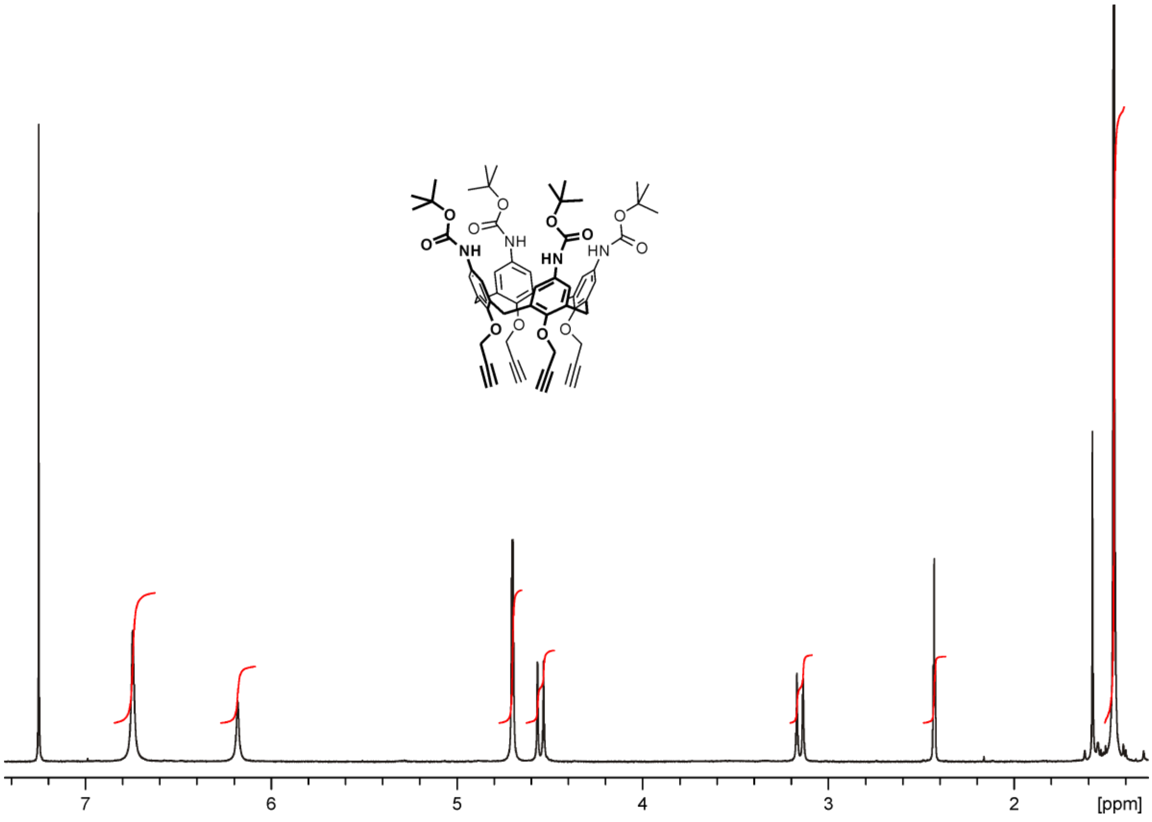

Figure S31. ^{1}H NMR spectrum of calixarene **24** (400 MHz, $CDCl_3$).

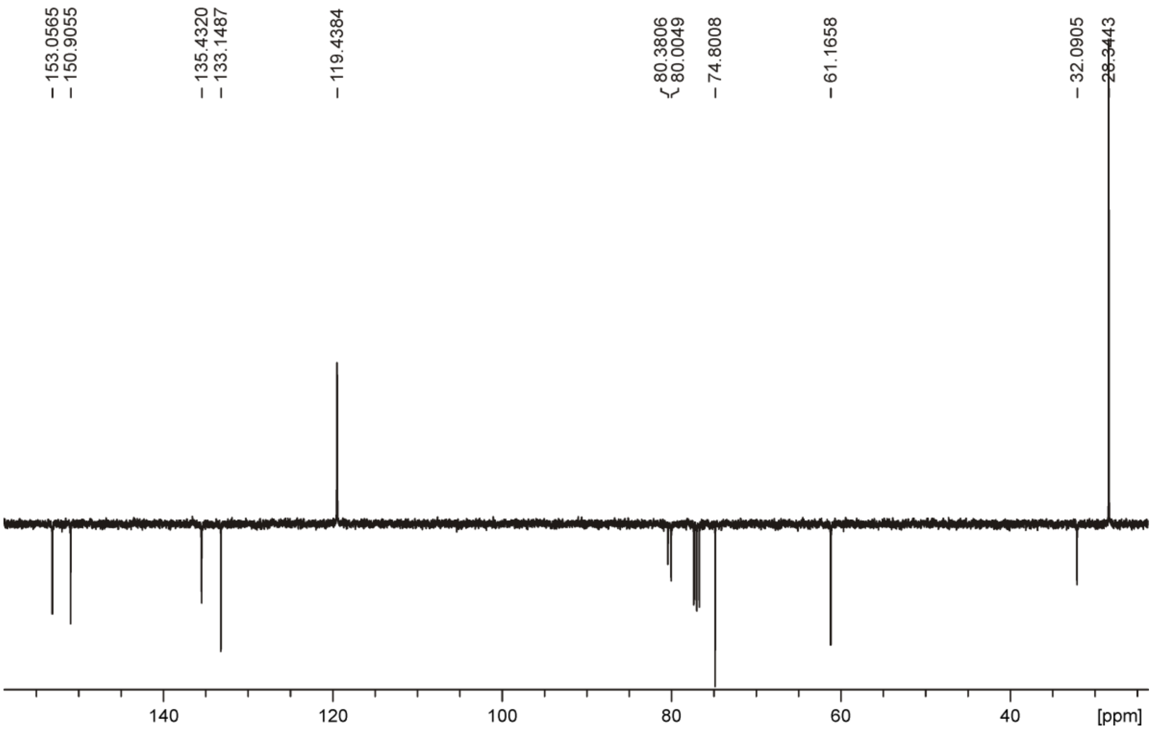

Figure S32. ^{13}C NMR spectrum (APT) of calixarene **24** (100 MHz, $CDCl_3$).

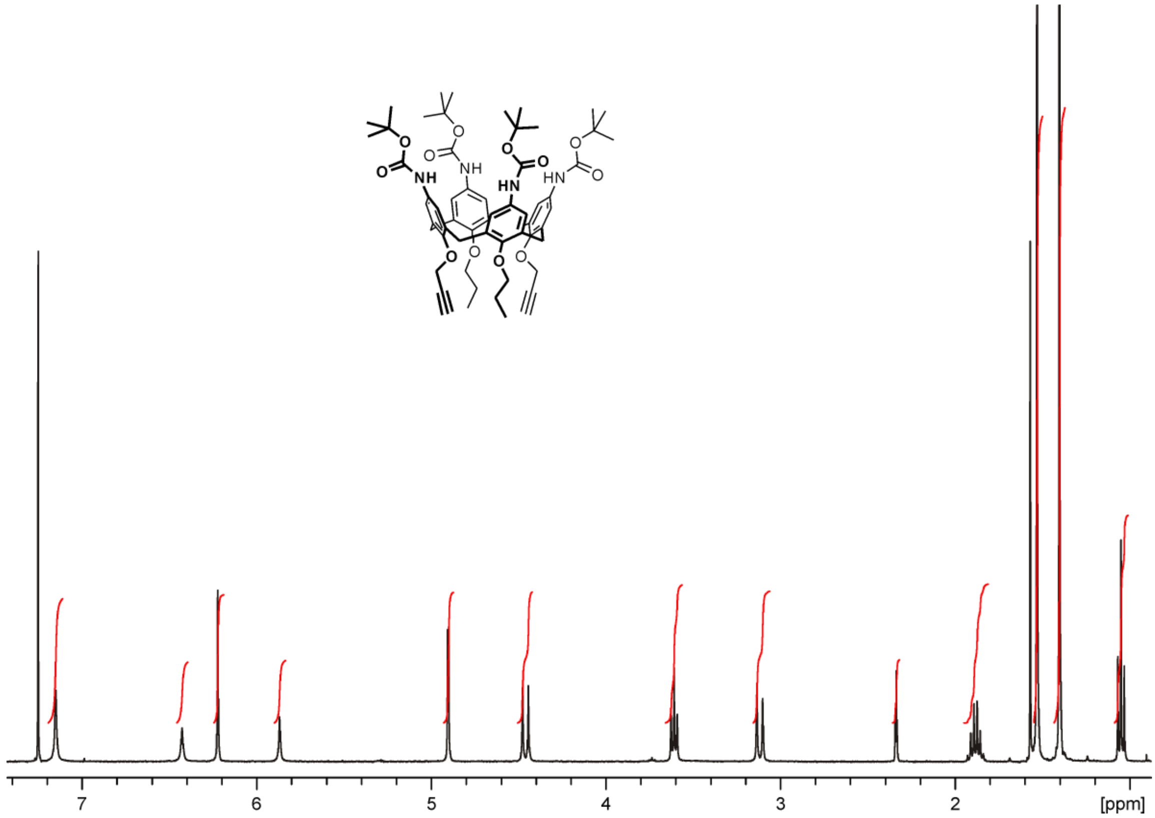

Figure S33. ^{1}H NMR spectrum of calixarene **25** (400 MHz, $CDCl_3$).

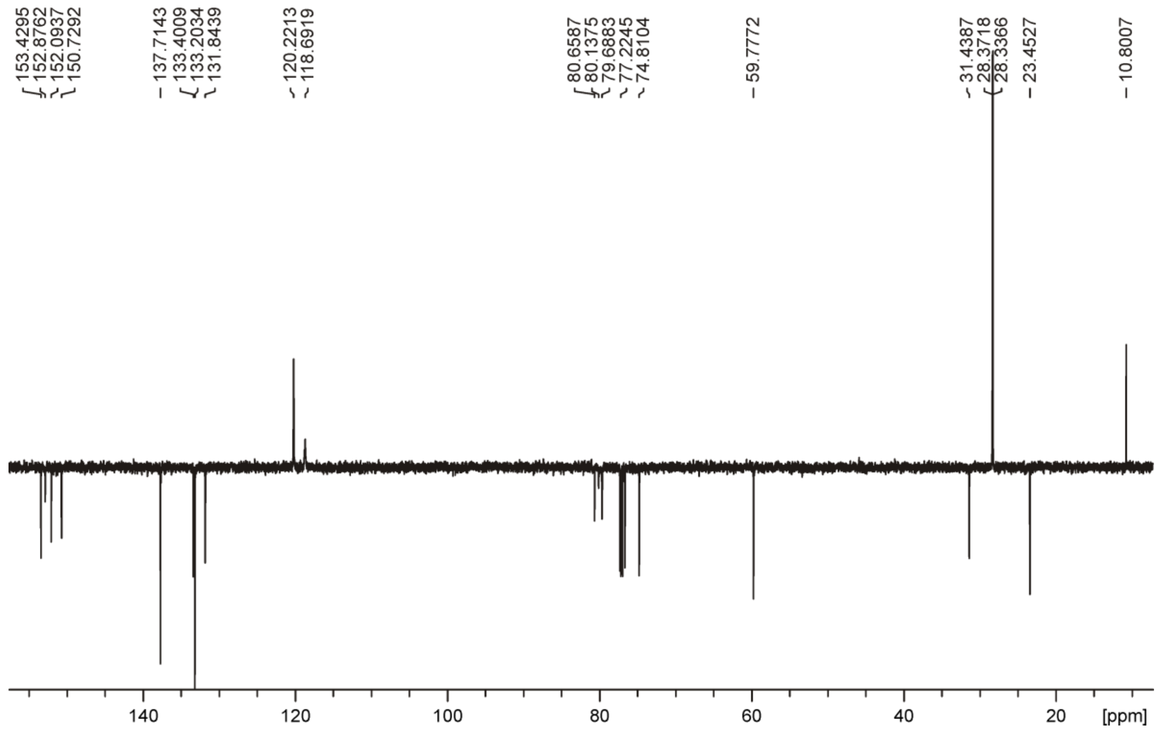

Figure S34. ^{13}C NMR spectrum (APT) of calixarene **25** (100 MHz, $CDCl_3$).

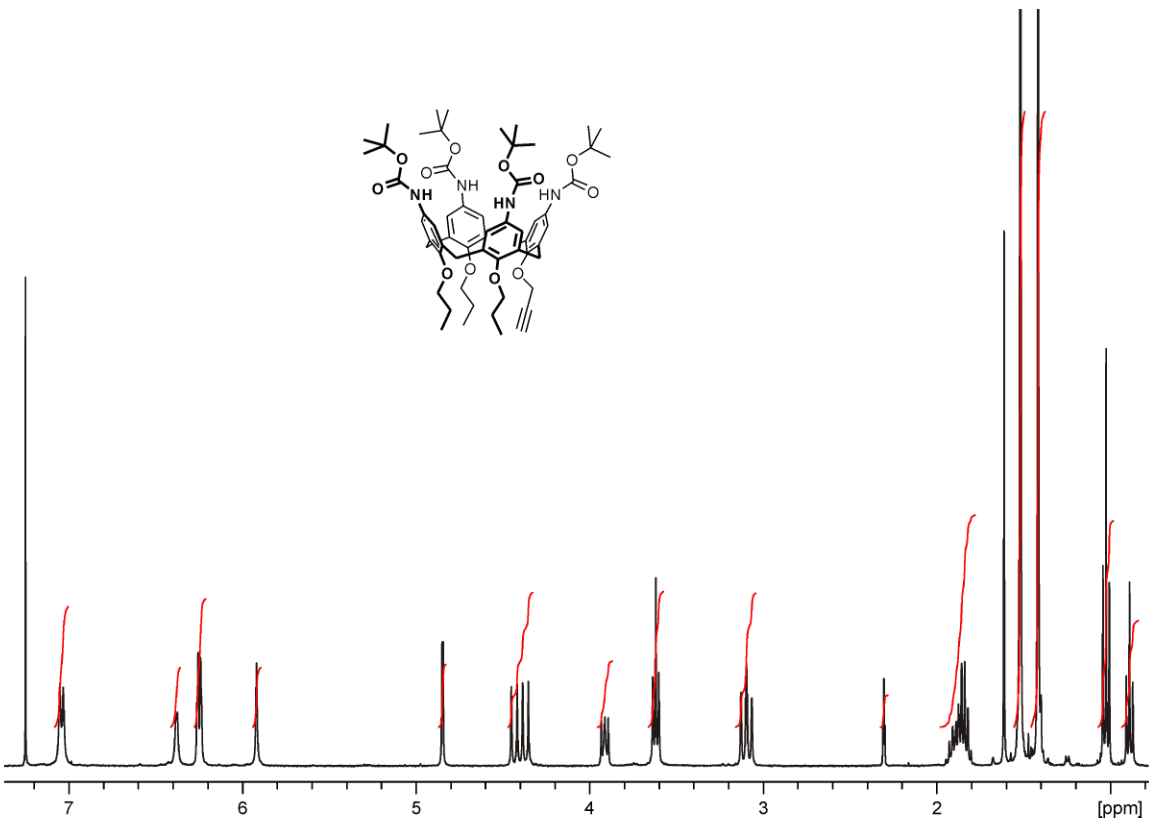

Figure S35. ^{1}H NMR spectrum of calixarene **26** (400 MHz, $CDCl_3$).

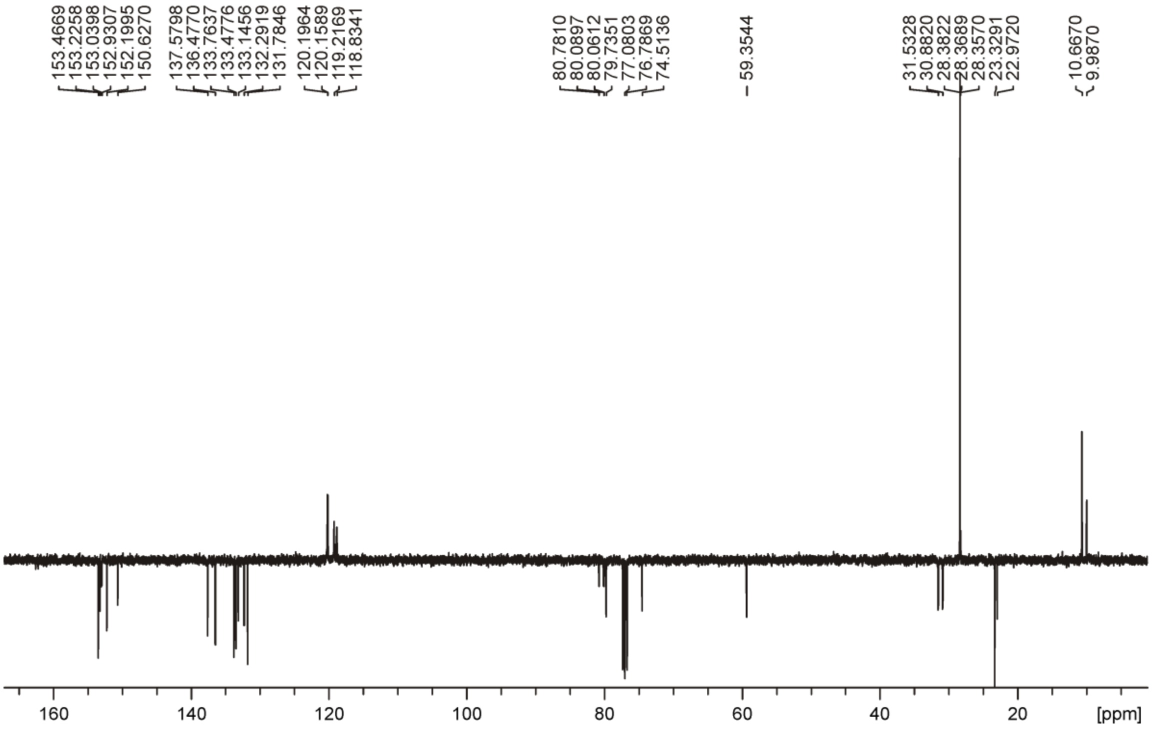

Figure S36. ^{13}C NMR spectrum (APT) of calixarene **26** (100 MHz, $CDCl_3$).

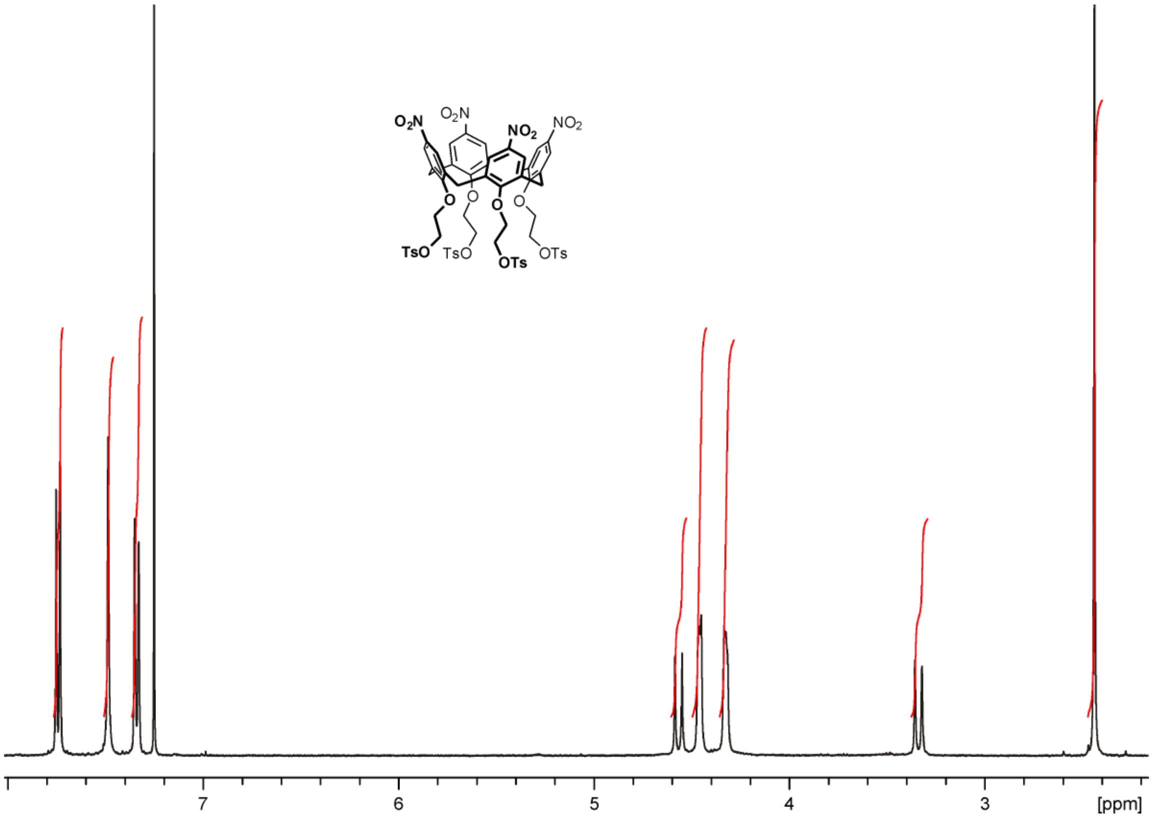

Figure S37. ^{1}H NMR spectrum of calixarene **29** (400 MHz, $CDCl_3$).

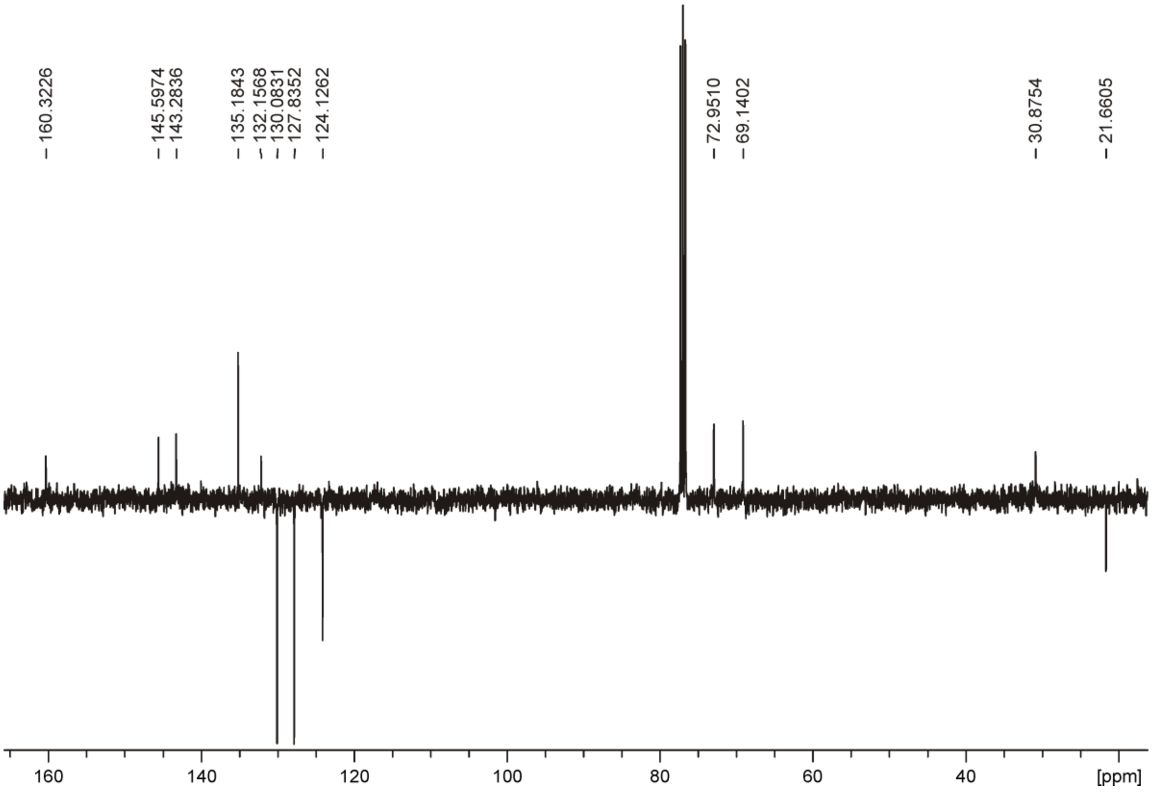

Figure S38. ^{13}C NMR spectrum (APT) of calixarene **29** (100 MHz, $CDCl_3$).

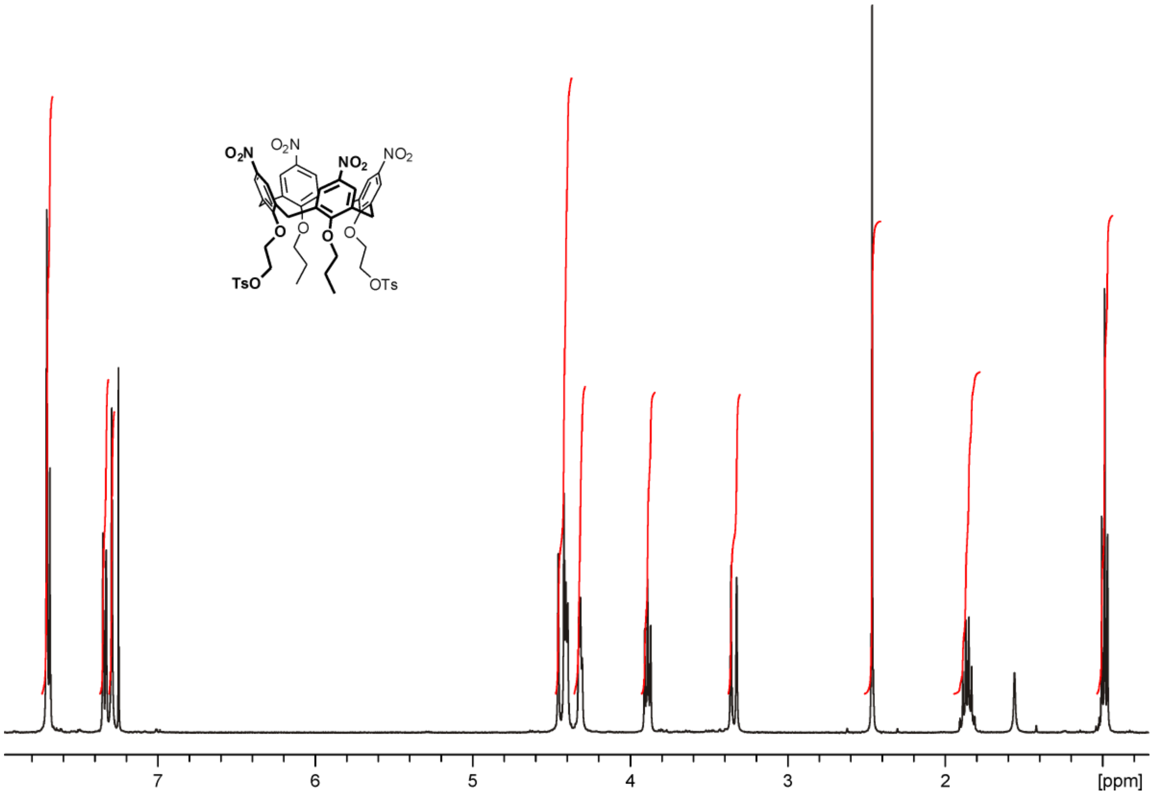

Figure S39. ^{1}H NMR spectrum of calixarene **30** (400 MHz, $CDCl_3$).

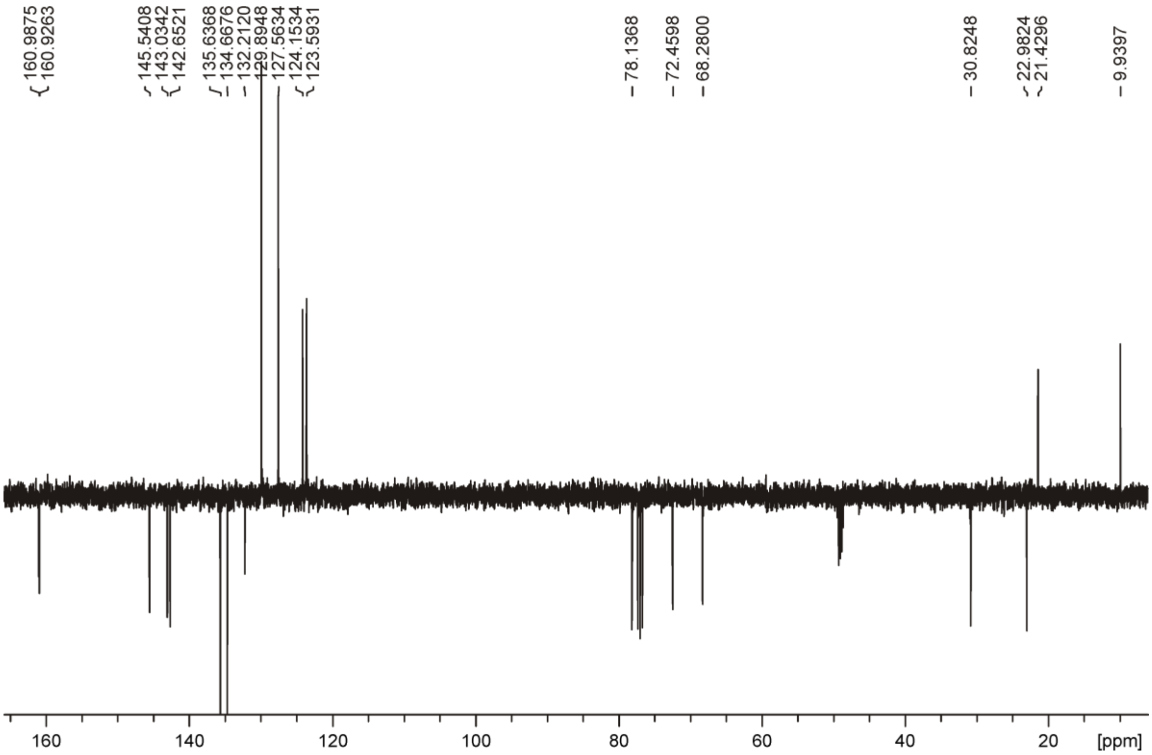

Figure S40. ^{13}C NMR spectrum (APT) of calixarene **30** (100 MHz, $CDCl_3$+CD_3OD).

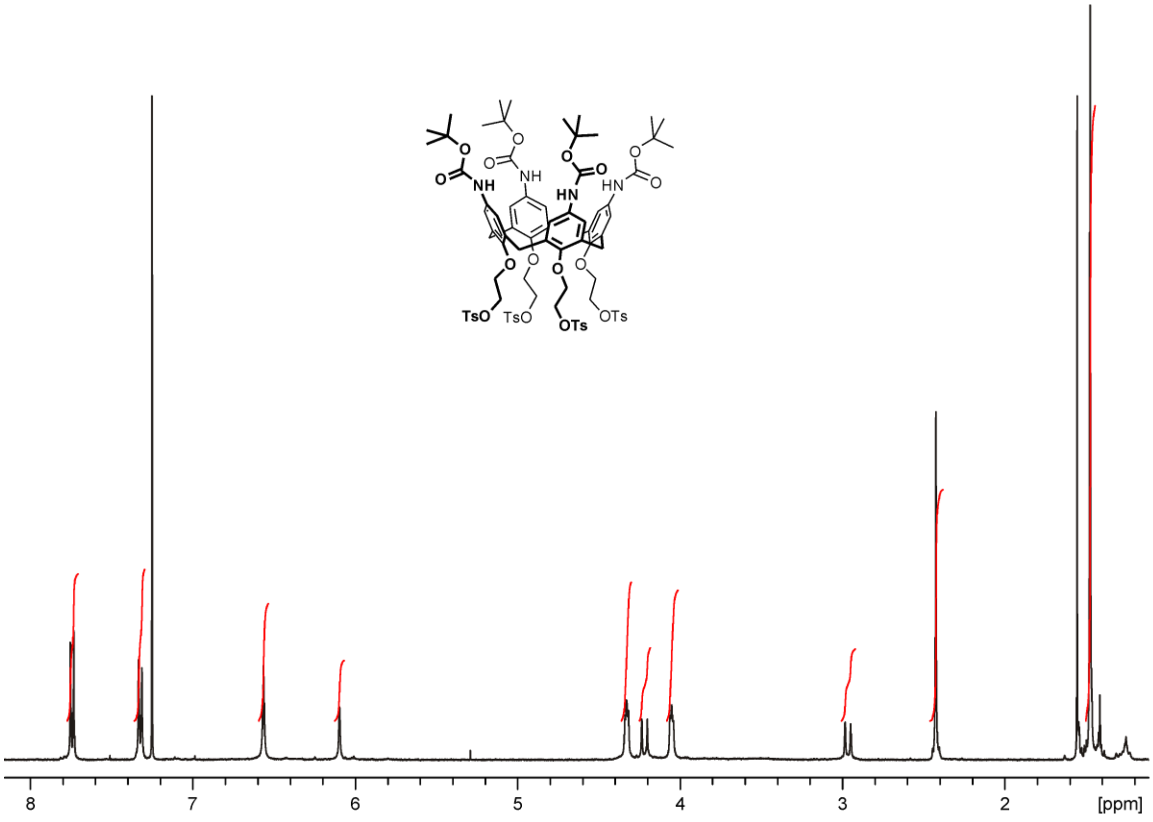

Figure S41. ^{1}H NMR spectrum of calixarene **33** (400 MHz, $CDCl_3$).

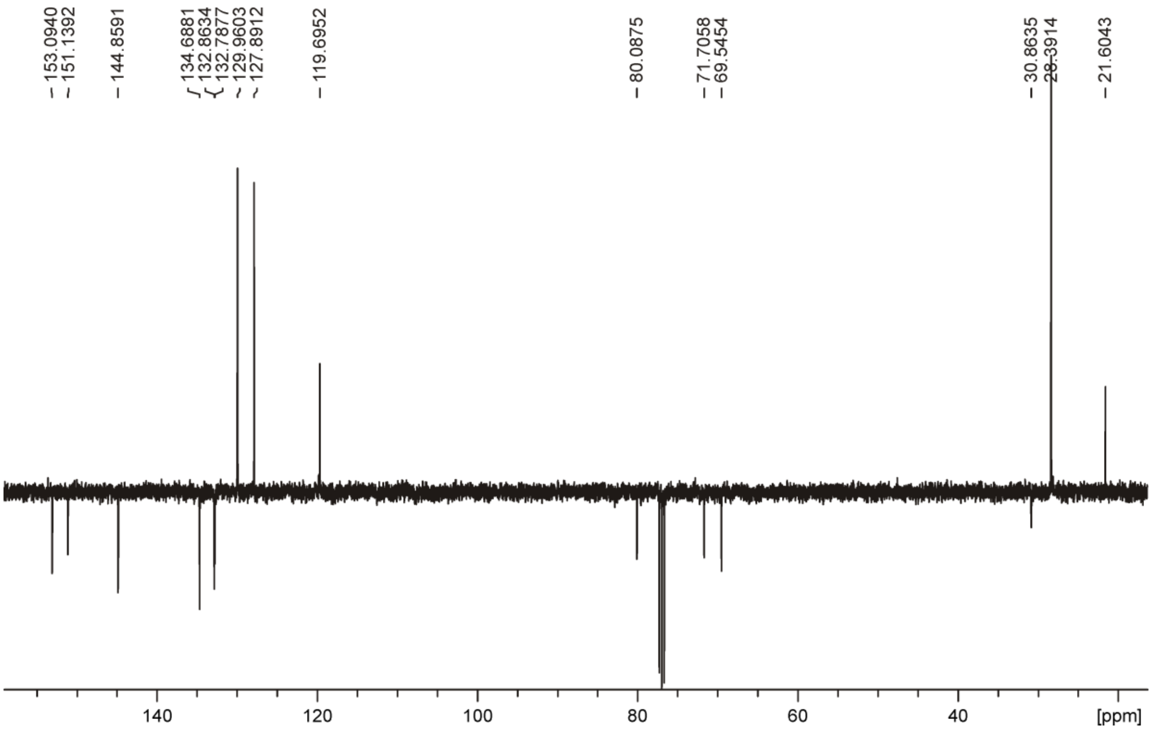

Figure S42. ^{13}C NMR spectrum (APT) of calixarene **33** (100 MHz, $CDCl_3$).

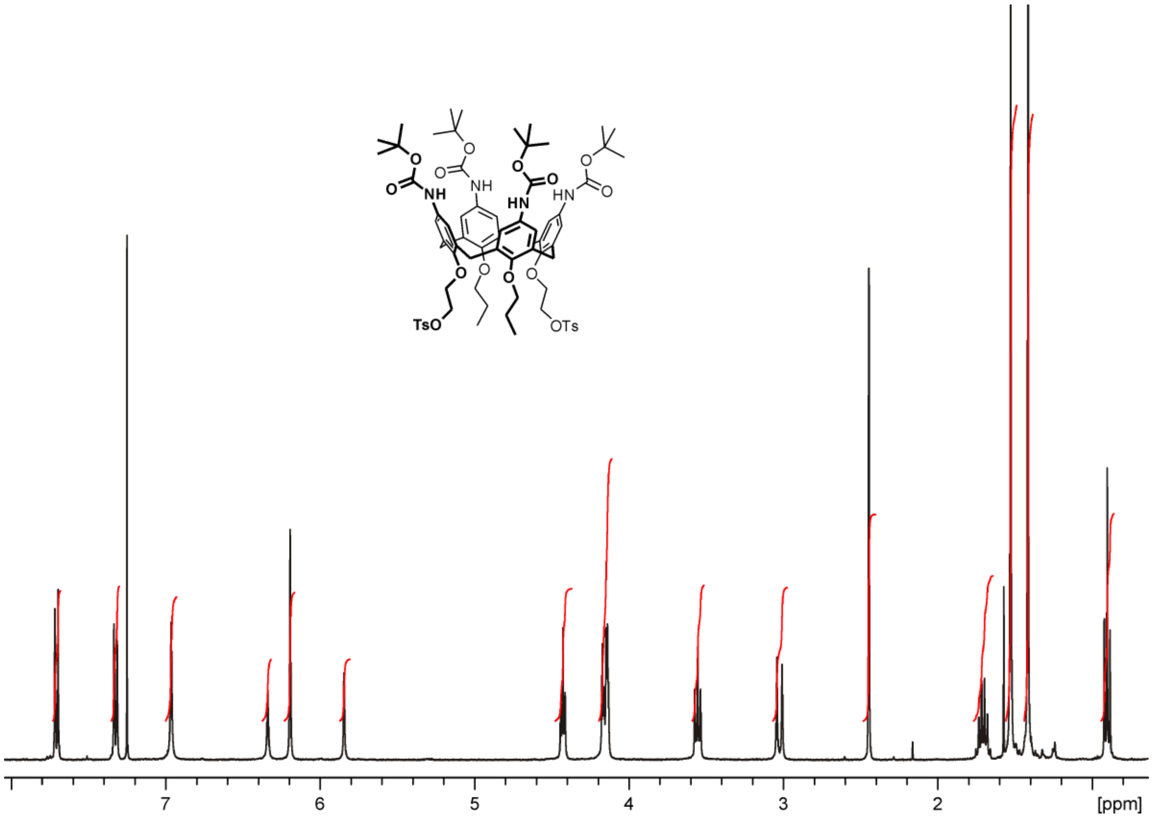

Figure S43. ^{1}H NMR spectrum of calixarene **34** (400 MHz, $CDCl_3$).

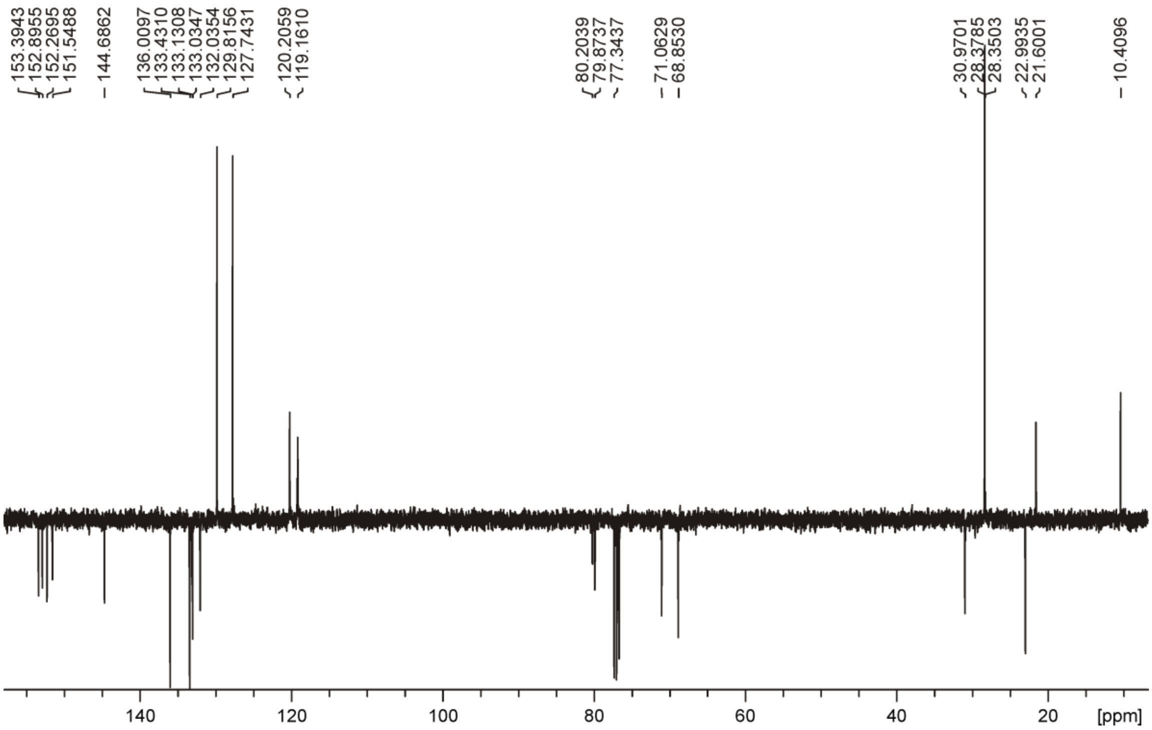

Figure S44. ^{13}C NMR spectrum (APT) of calixarene **34** (100 MHz, $CDCl_3$).

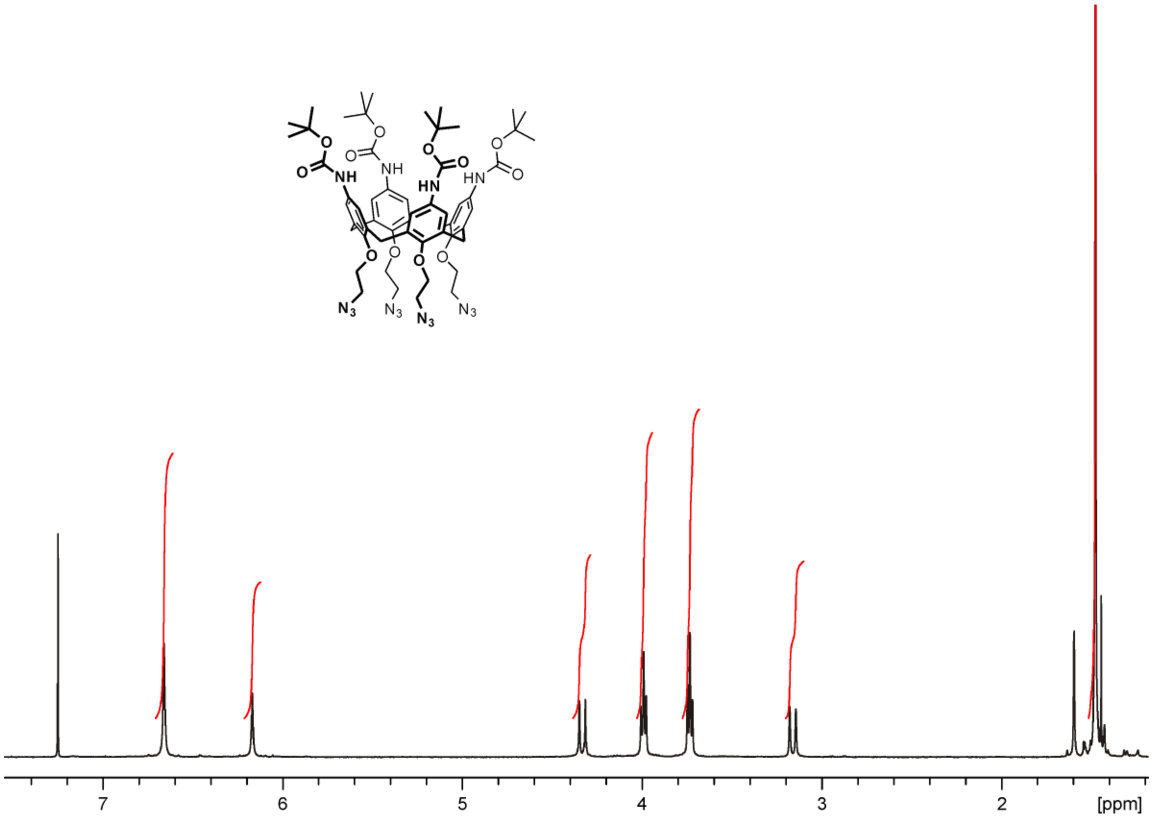

Figure S45. ^{1}H NMR spectrum of calixarene **35** (400 MHz, $CDCl_3$).

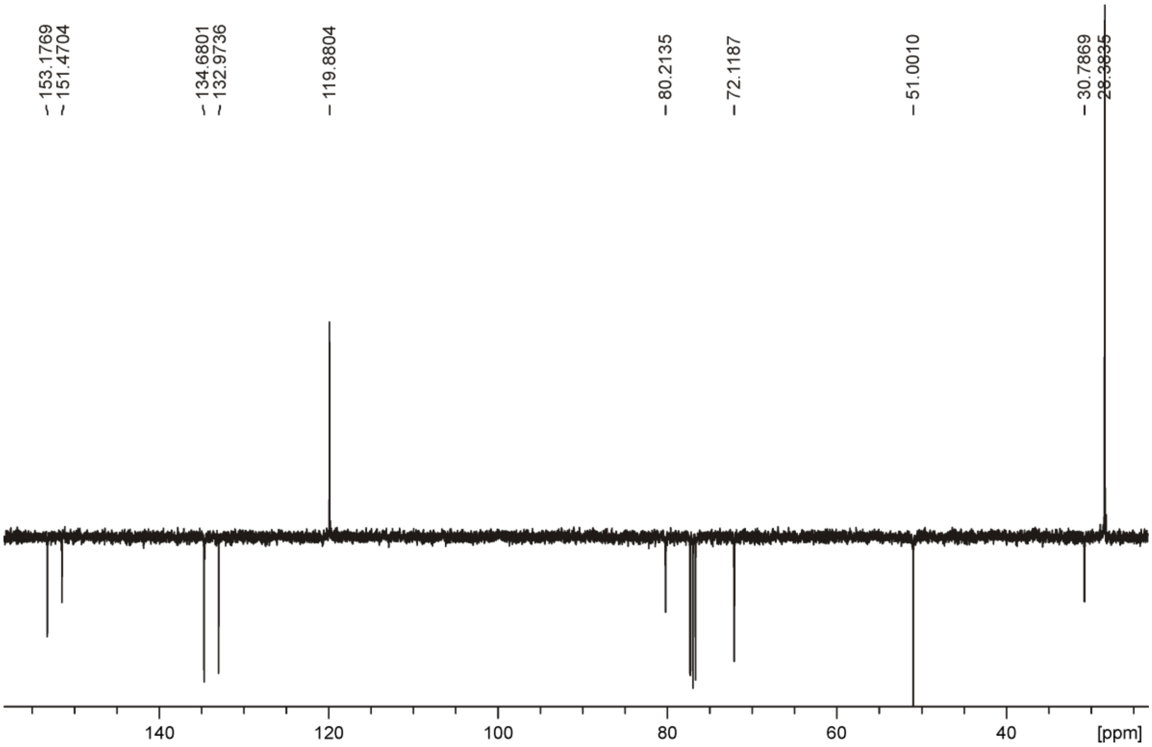

Figure S46. ^{13}C NMR spectrum (APT) of calixarene **35** (100 MHz, $CDCl_3$).

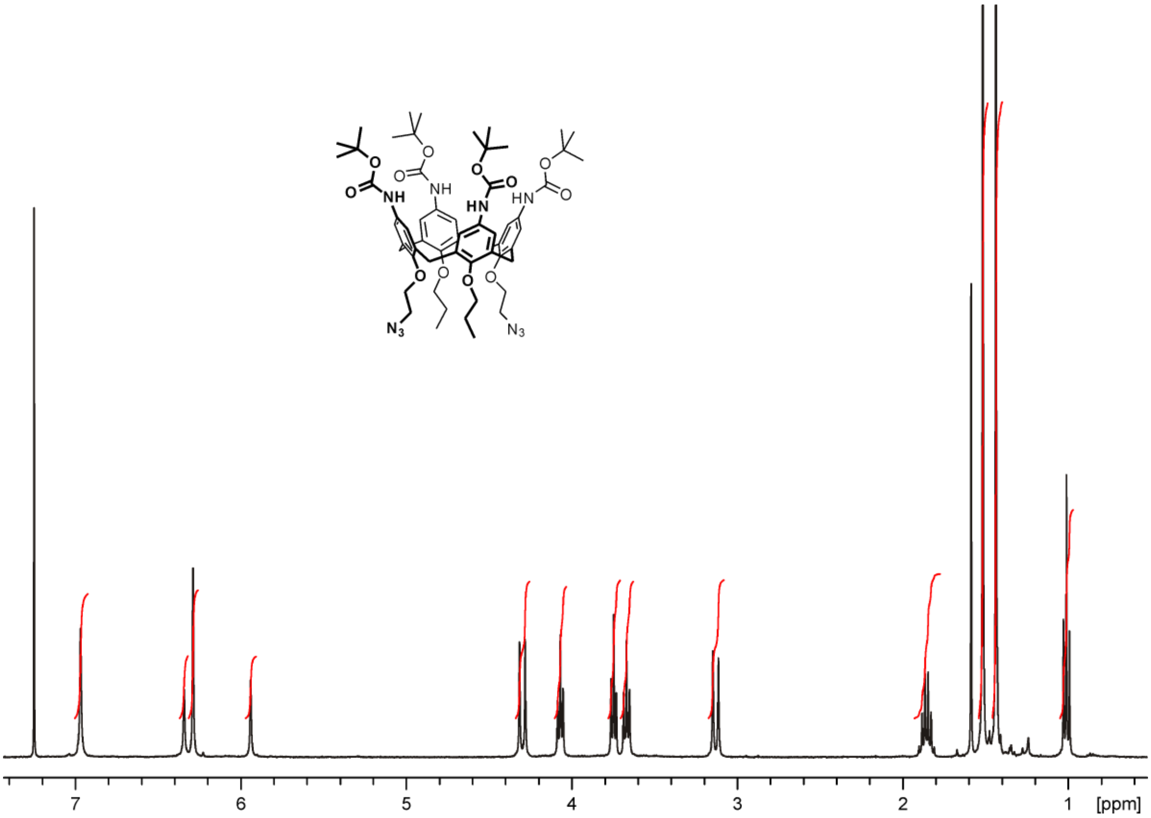

Figure S47. ^{1}H NMR spectrum of calixarene **36** (400 MHz, $CDCl_3$).

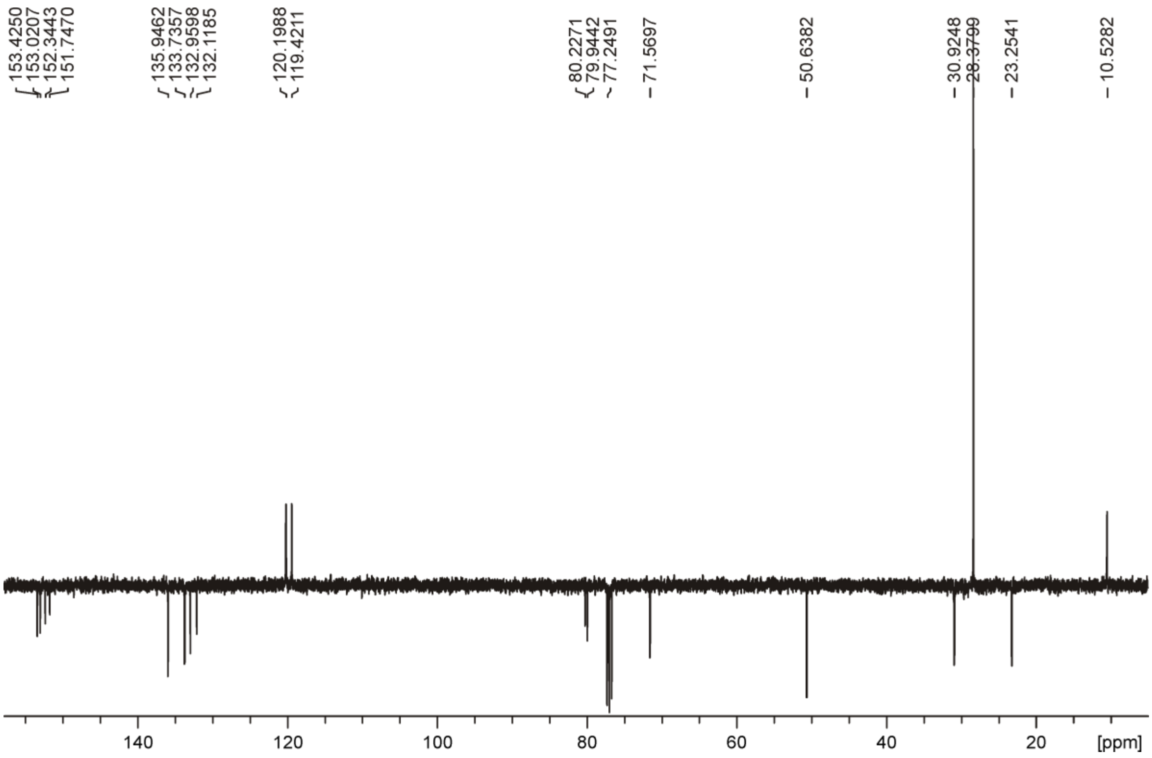

Figure S48. ^{13}C NMR spectrum (APT) of calixarene **36** (100 MHz, $CDCl_3$).

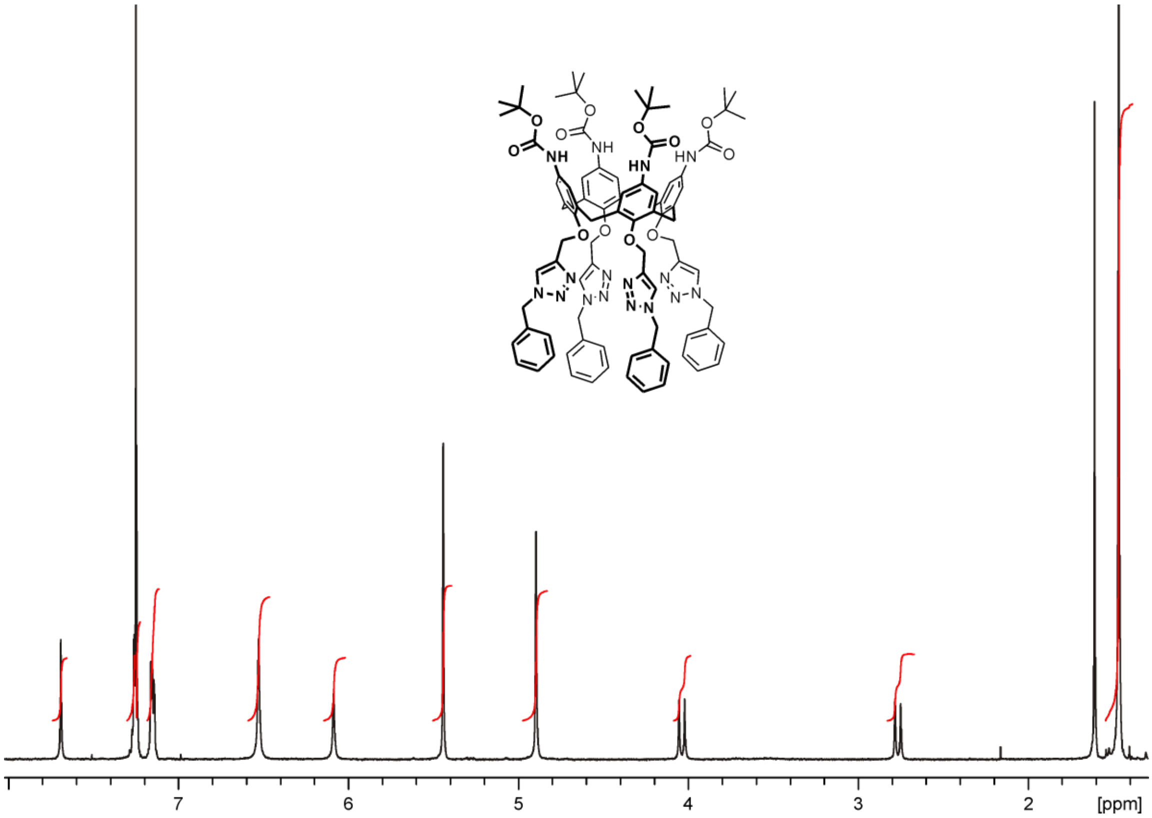

Figure S49. ^{1}H NMR spectrum of calixarene **37** (400 MHz, $CDCl_3$).

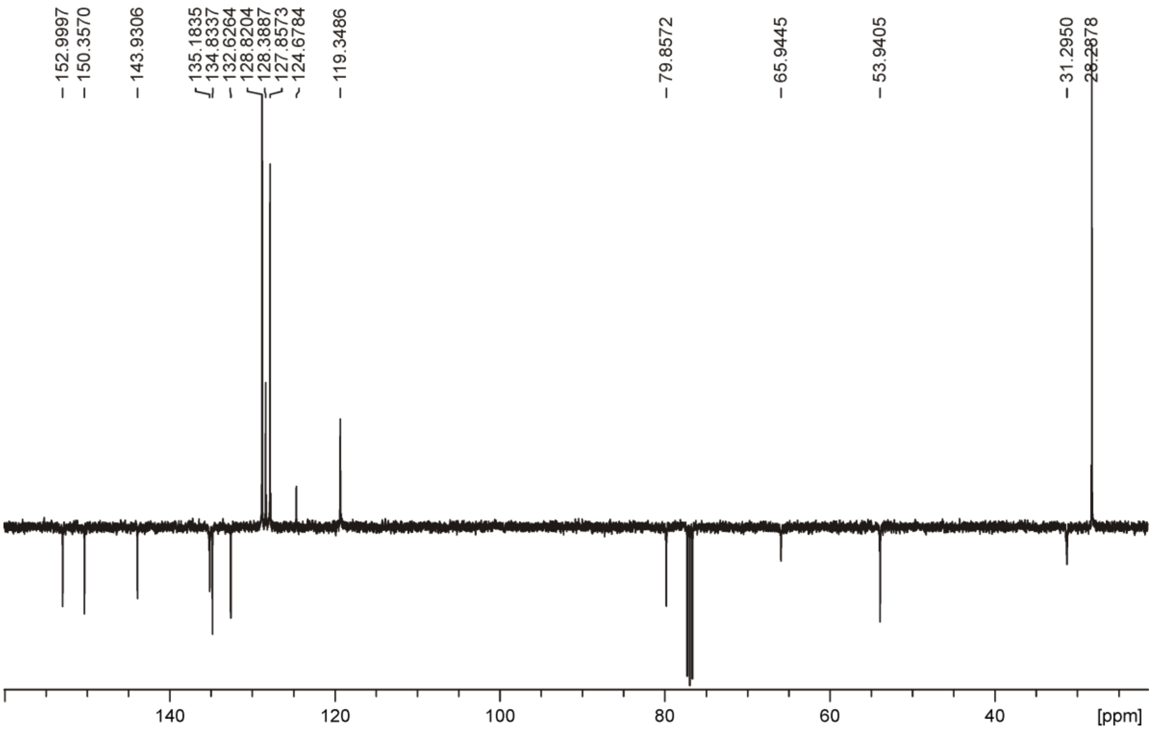

Figure S50. ^{13}C NMR spectrum (APT) of calixarene **37** (100 MHz, $CDCl_3$).

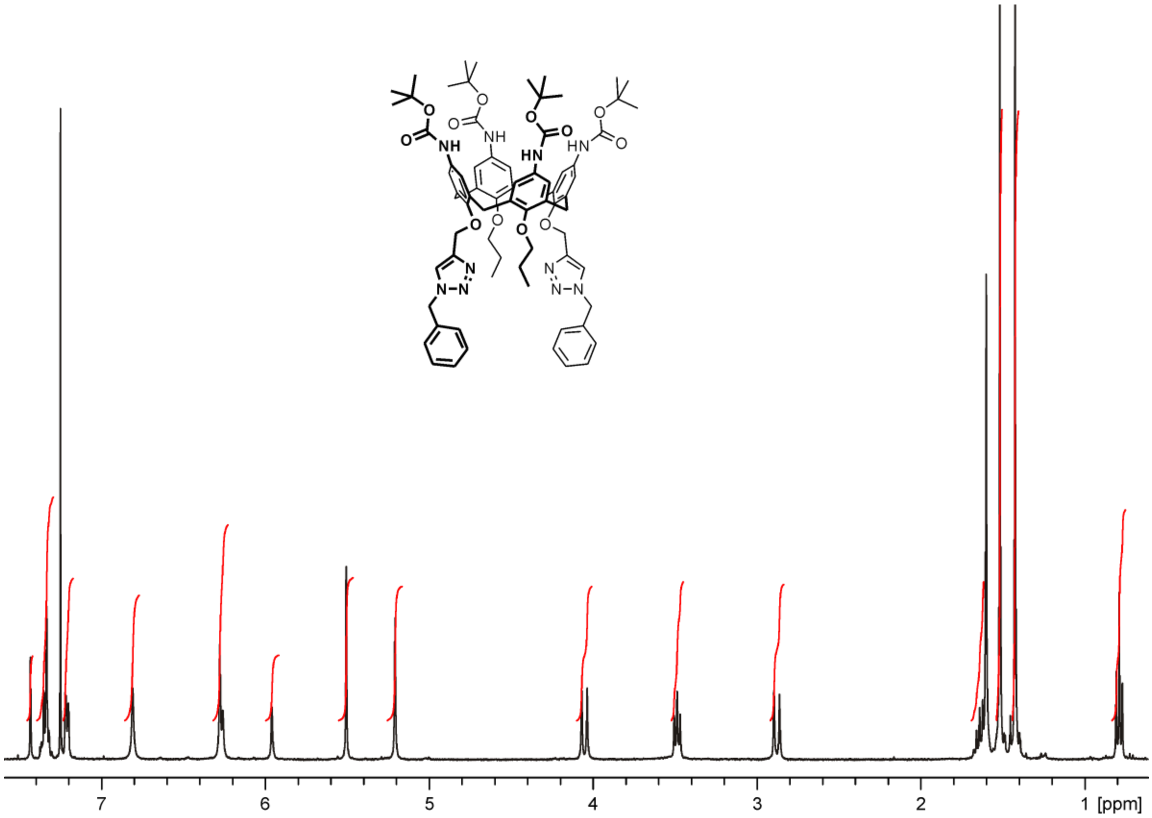

Figure S51. ^{1}H NMR spectrum of calixarene **38** (400 MHz, $CDCl_3$).

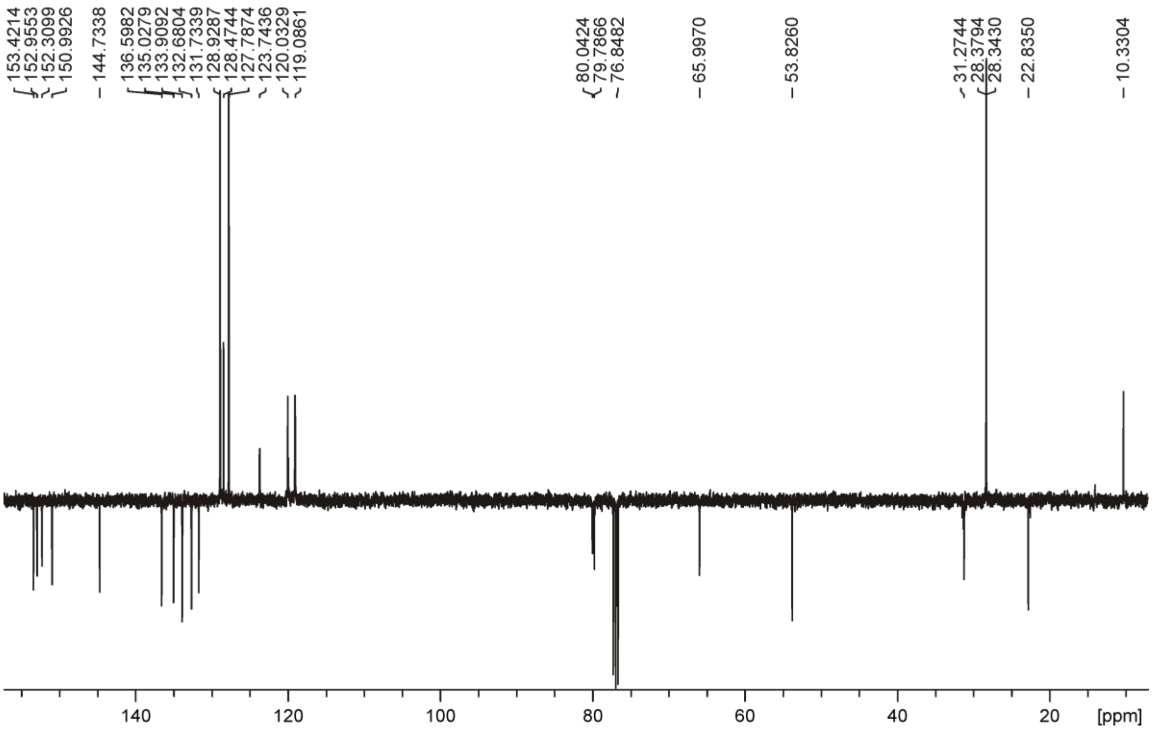

Figure S52. ^{13}C NMR spectrum (APT) of calixarene **38** (100 MHz, $CDCl_3$).

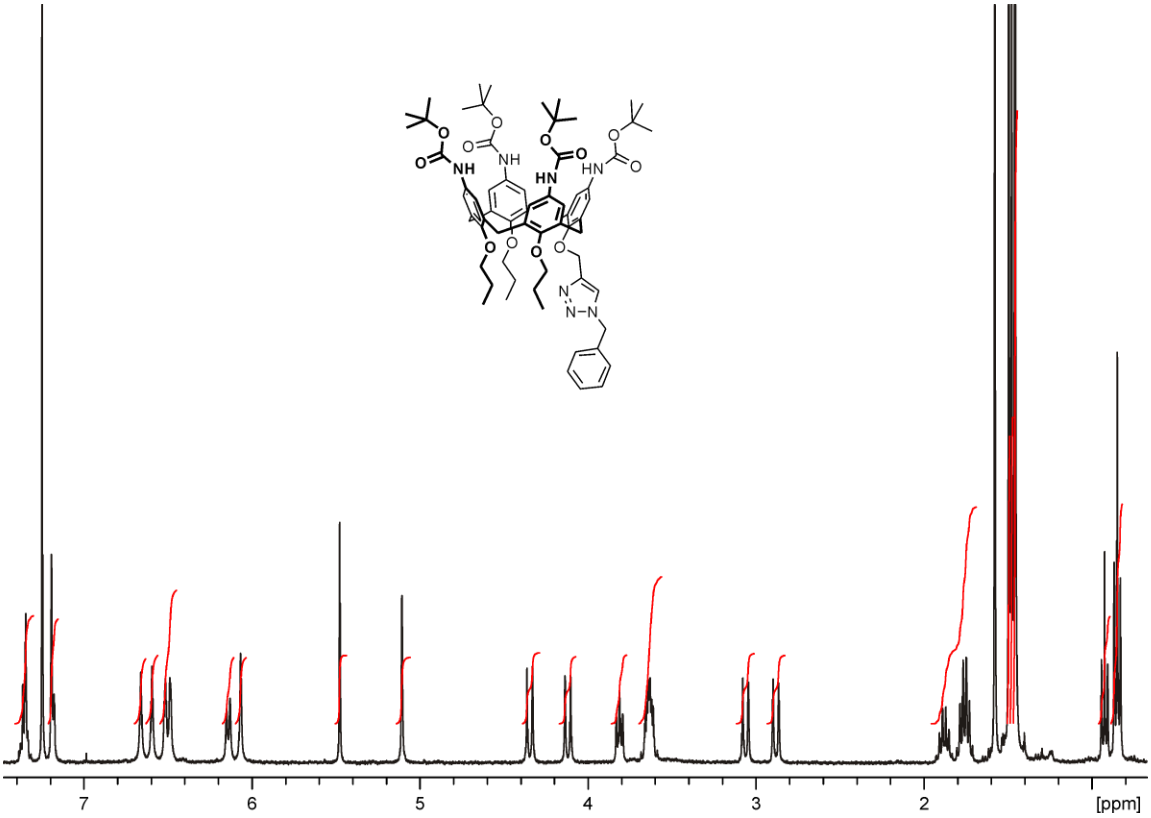

Figure S53. ^{1}H NMR spectrum of calixarene **39** (400 MHz, $CDCl_3$).

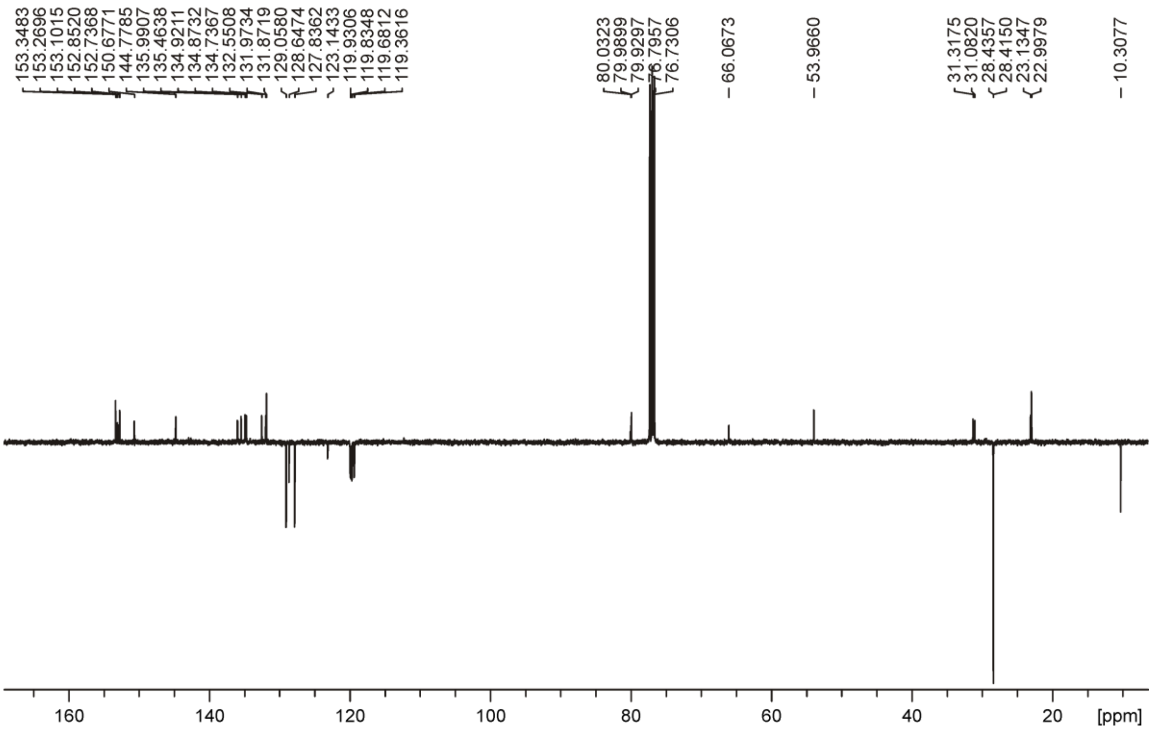

Figure S54. ^{13}C NMR spectrum (APT) of calixarene **39** (100 MHz, $CDCl_3$).

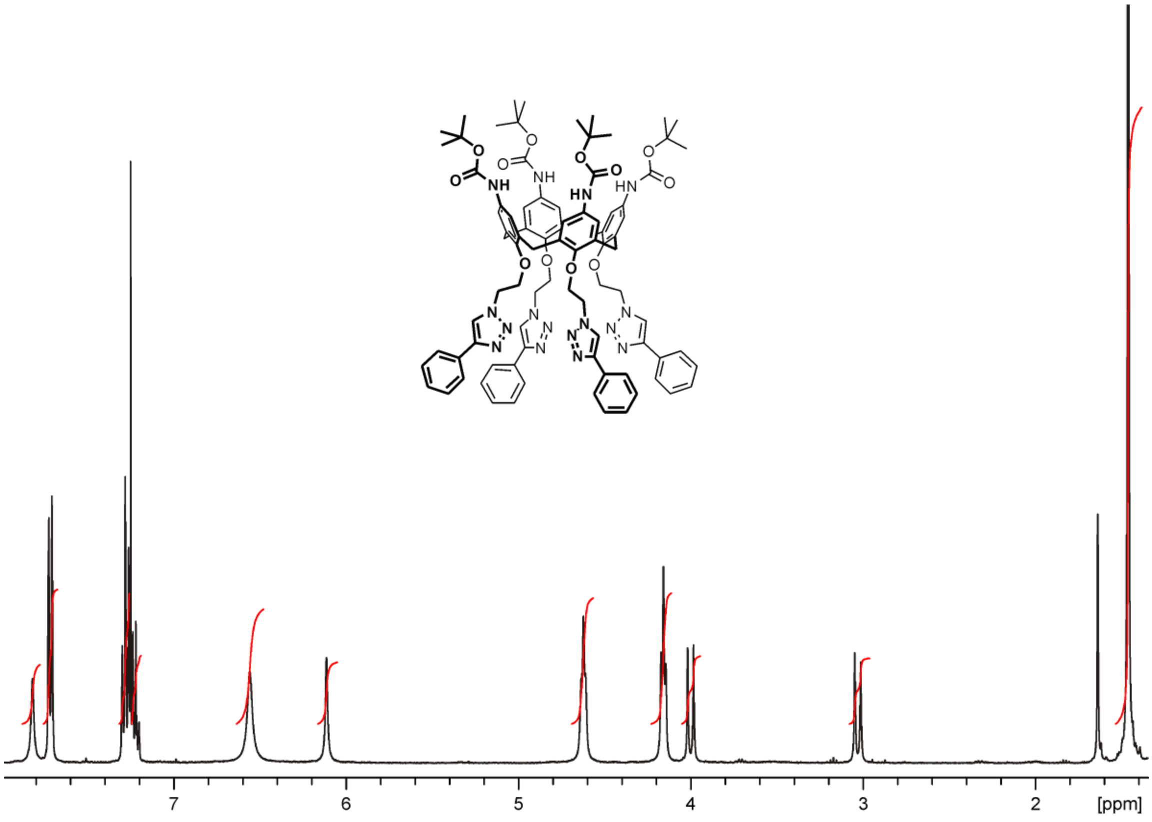

Figure S55. ^{1}H NMR spectrum of calixarene **40** (400 MHz, $CDCl_3$).

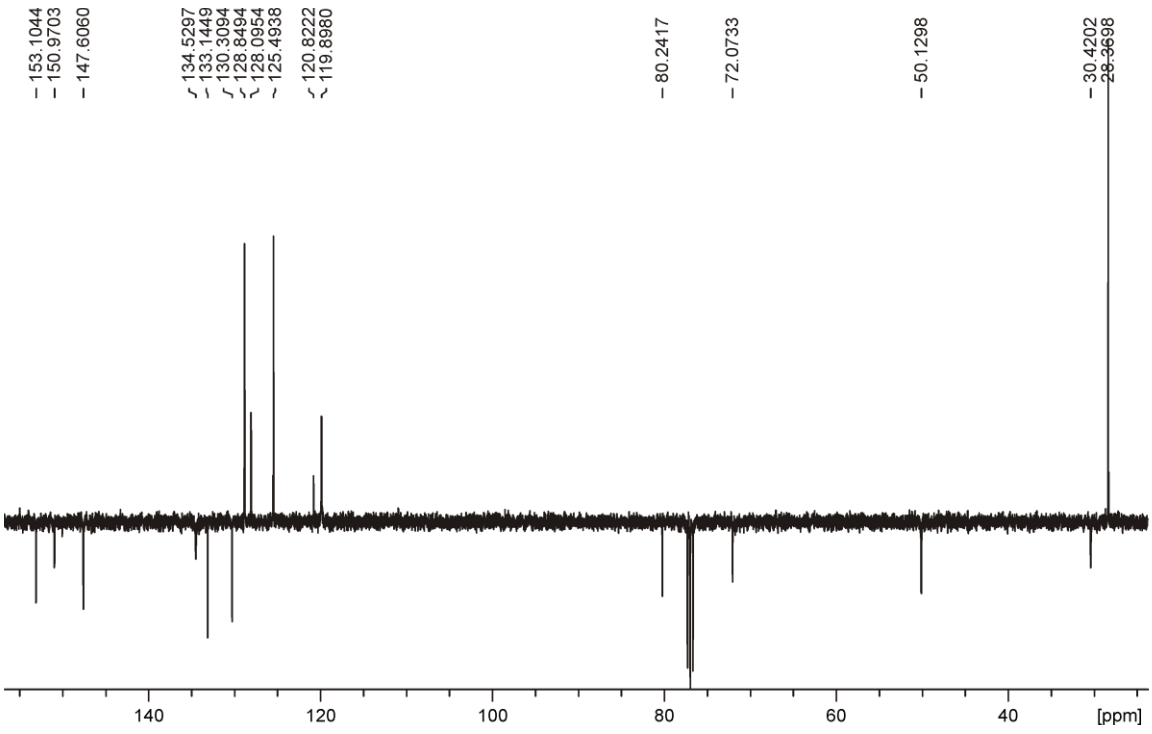

Figure S56. ^{13}C NMR spectrum (APT) of calixarene **40** (100 MHz, $CDCl_3$).

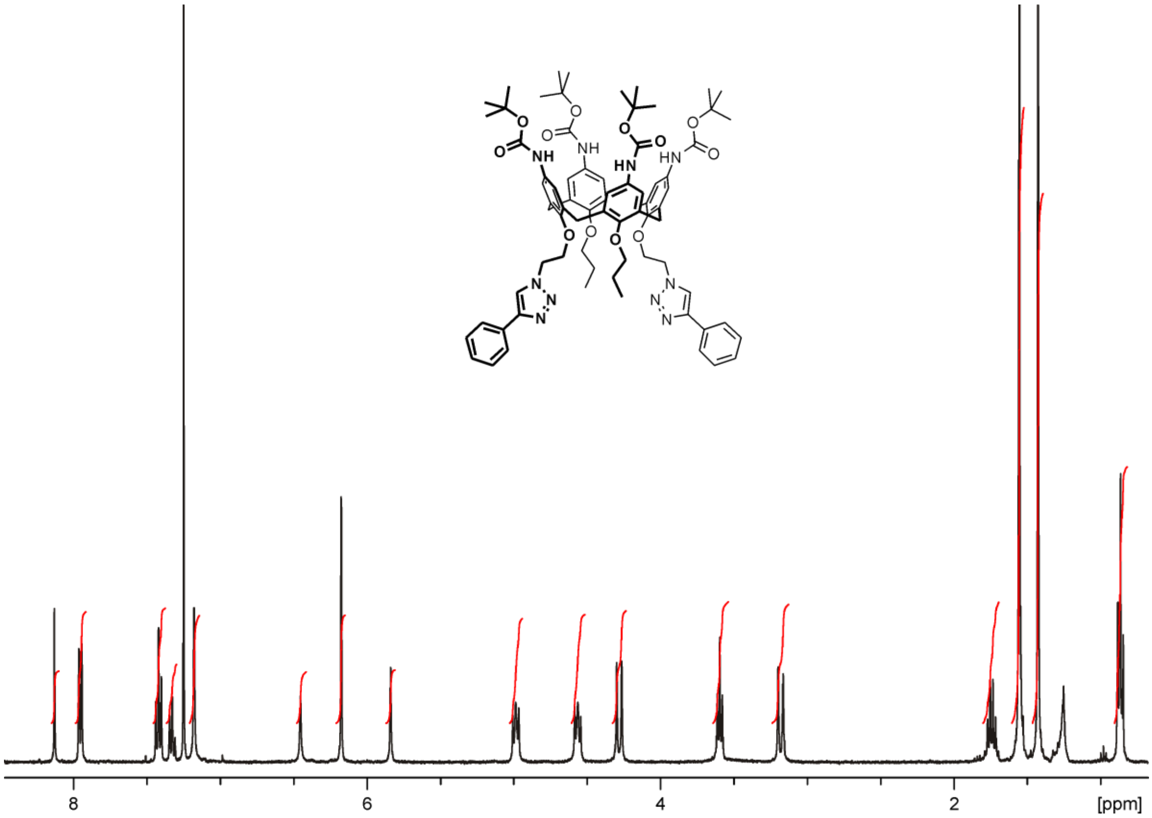

Figure S57. ^{1}H NMR spectrum of calixarene **41** (400 MHz, $CDCl_3$).

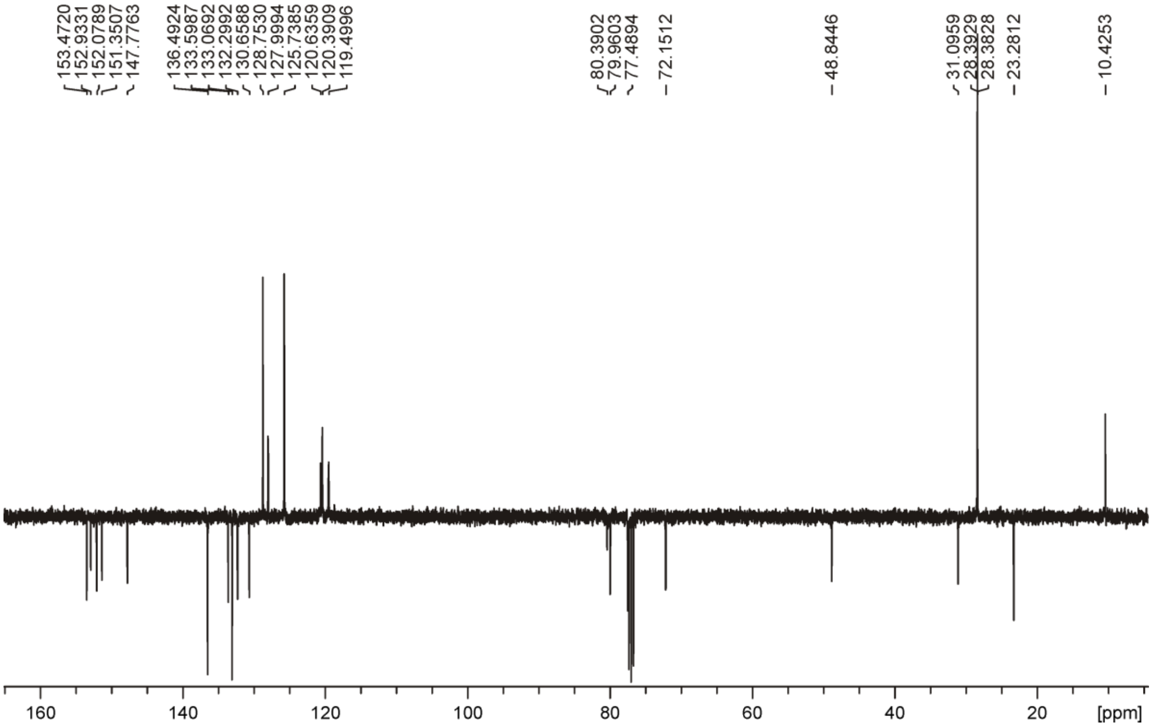

Figure S58. ^{13}C NMR spectrum (APT) of calixarene **41** (100 MHz, $CDCl_3$).

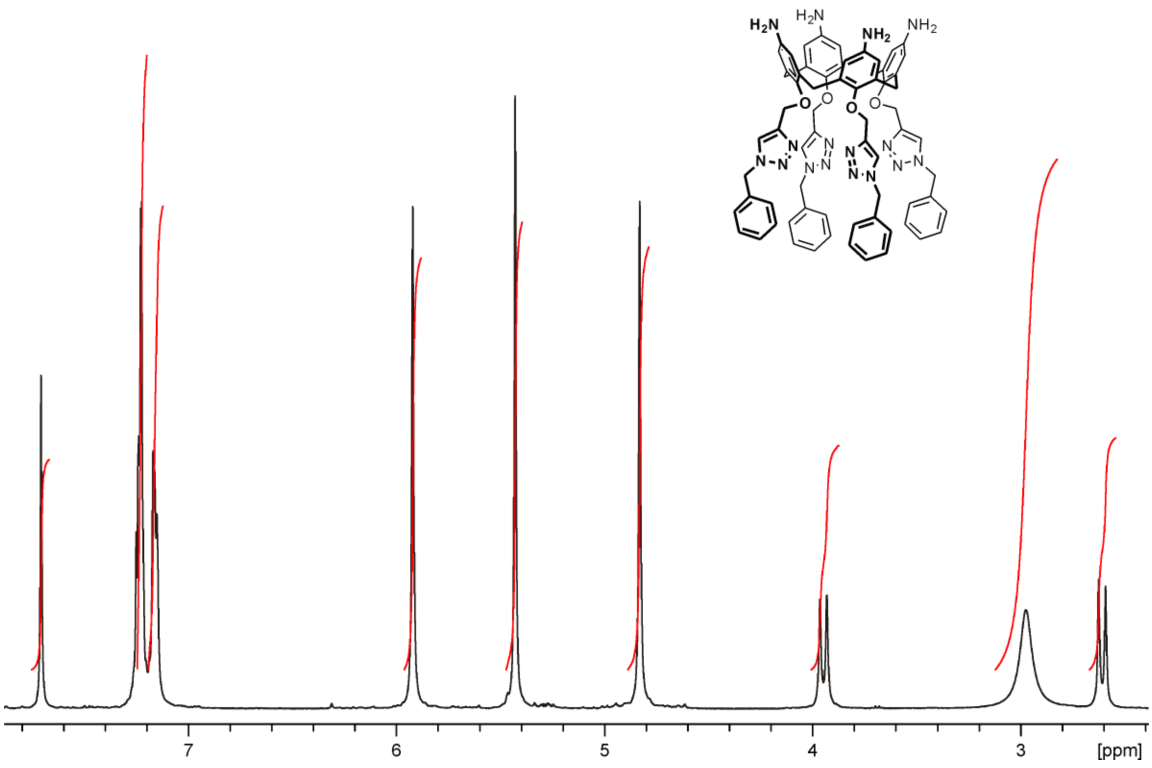

Figure S59. ^{1}H NMR spectrum of calixarene **42** (400 MHz, $CDCl_3$).

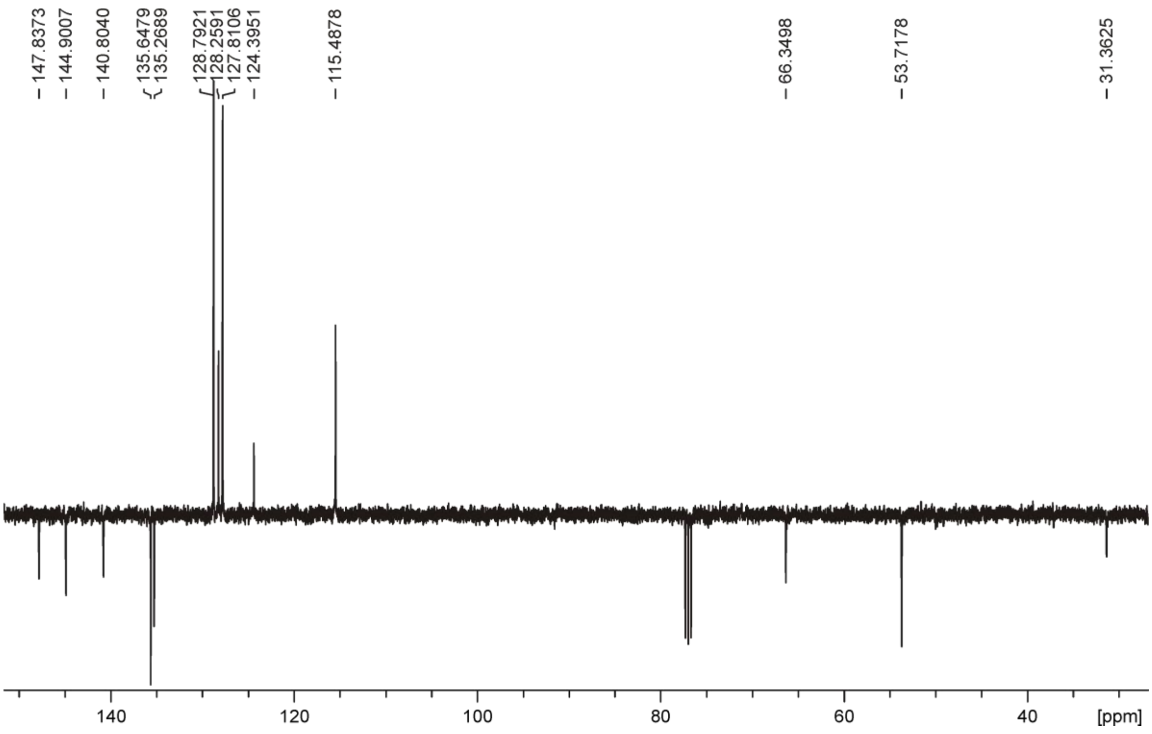

Figure S60. ^{13}C NMR spectrum (APT) of calixarene **42** (100 MHz, $CDCl_3$).

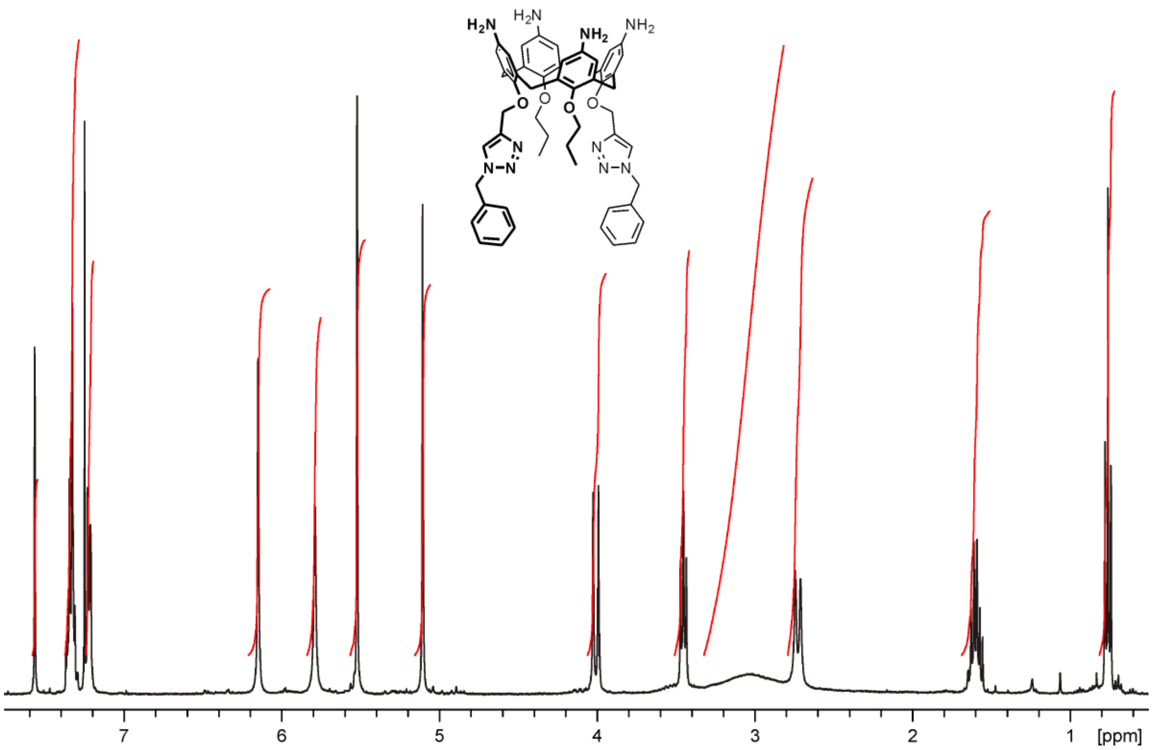

Figure S61. ^{1}H NMR spectrum of calixarene **43** (400 MHz, $CDCl_3$).

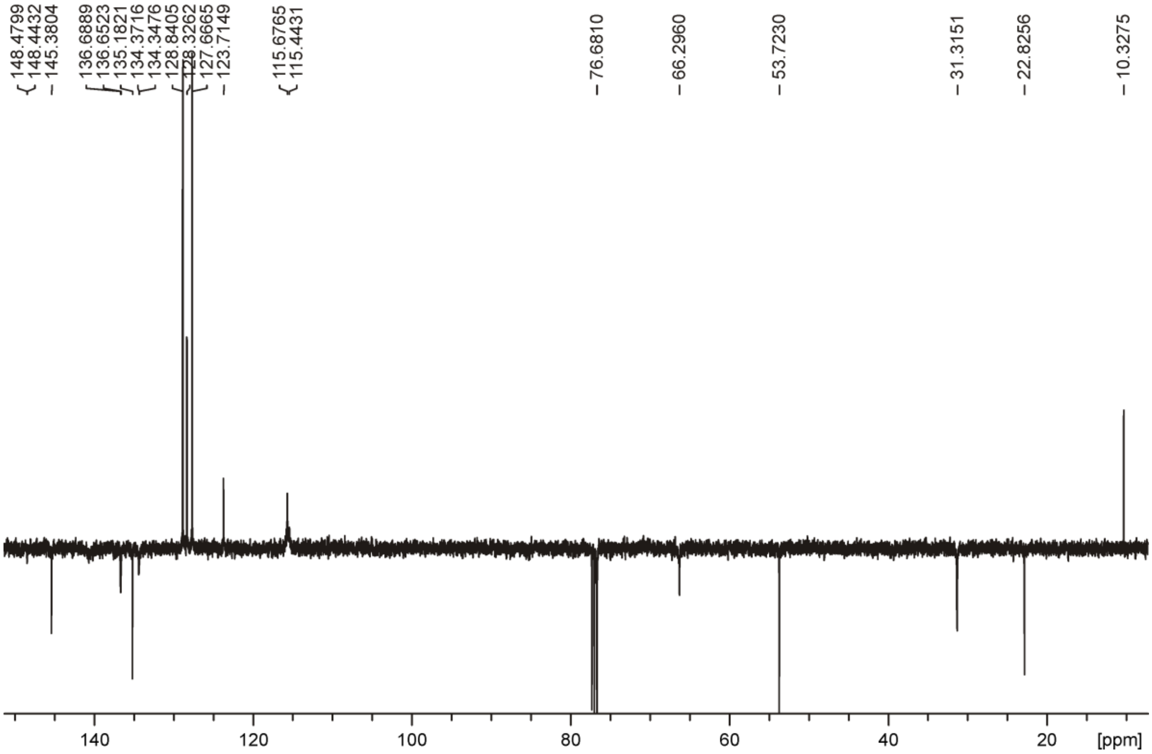

Figure S62. ^{13}C NMR spectrum (APT) of calixarene **43** (100 MHz, $CDCl_3$).

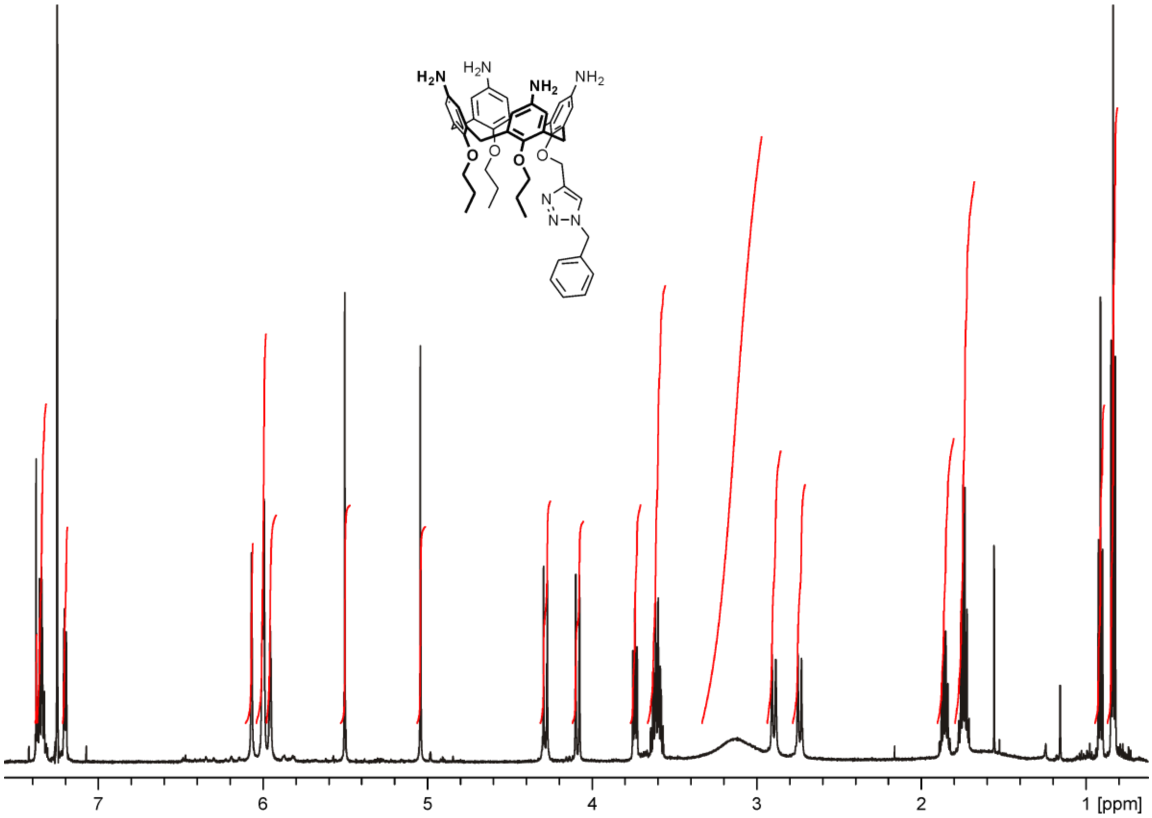

Figure S63. ^{1}H NMR spectrum of calixarene **44** (400 MHz, $CDCl_3$).

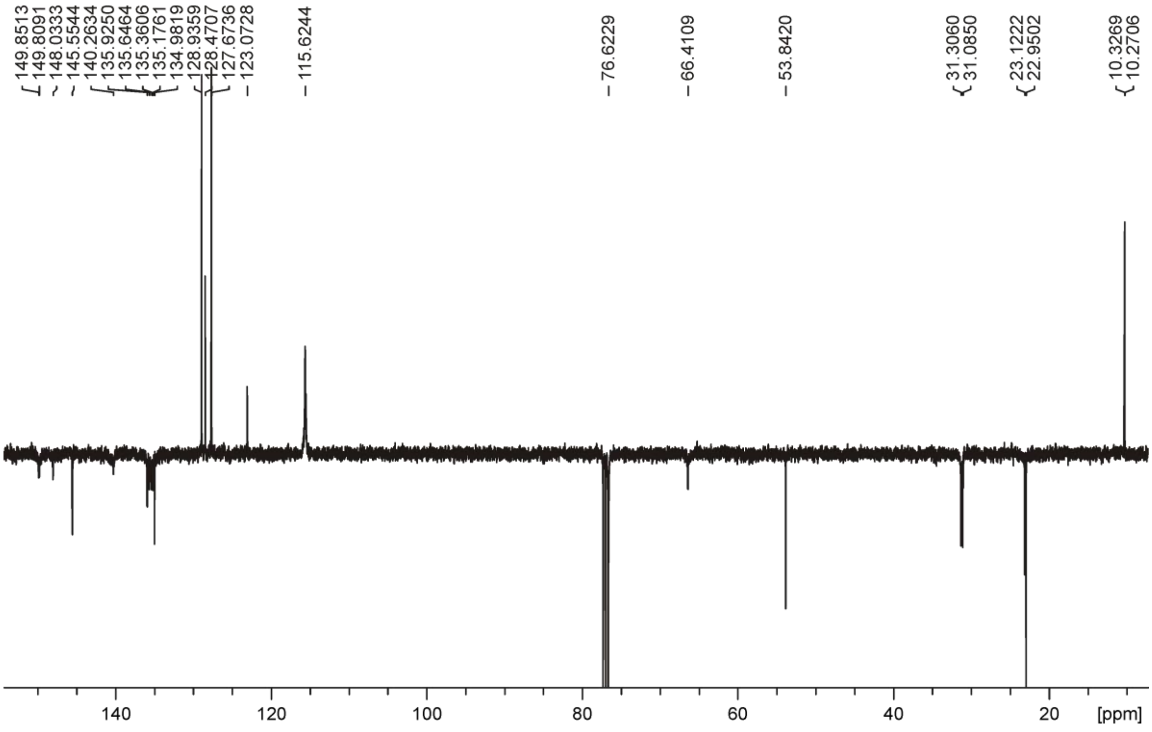

Figure S64. ^{13}C NMR spectrum (APT) of calixarene **44** (100 MHz, $CDCl_3$).

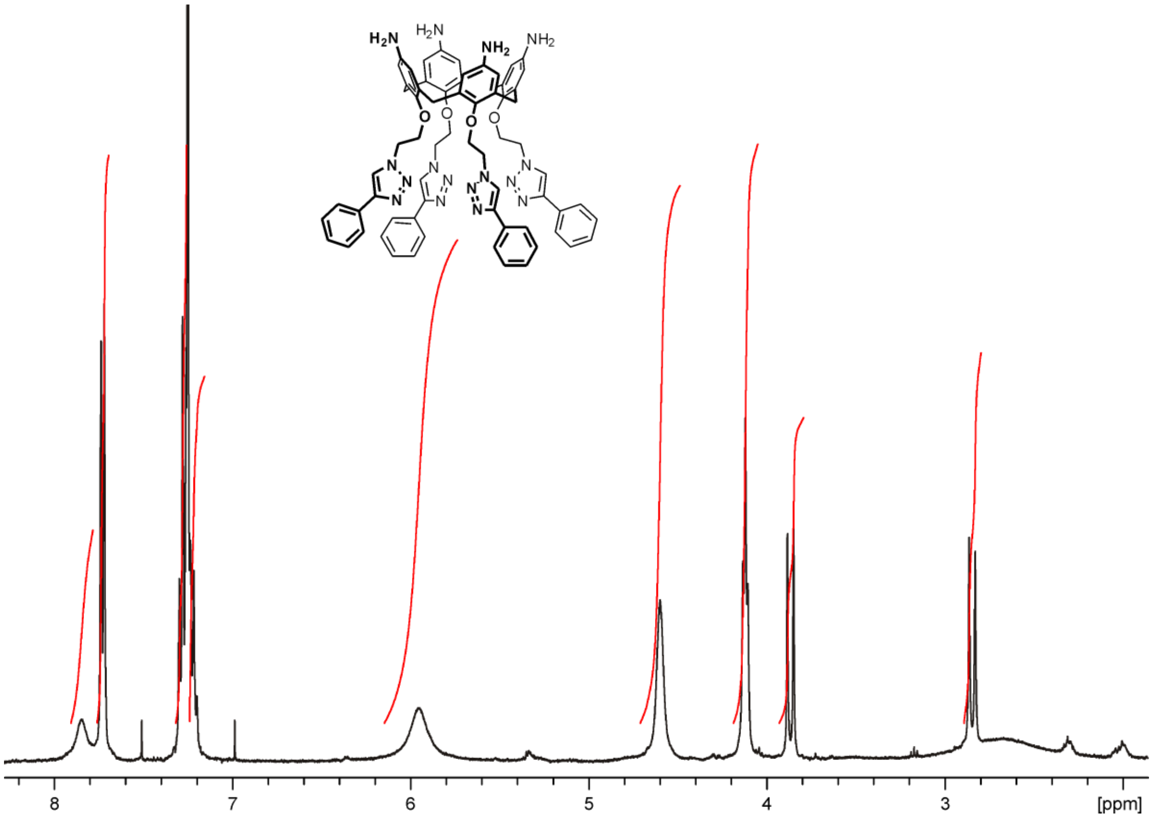

Figure S65. ^{1}H NMR spectrum of calixarene **45** (400 MHz, $CDCl_3$).

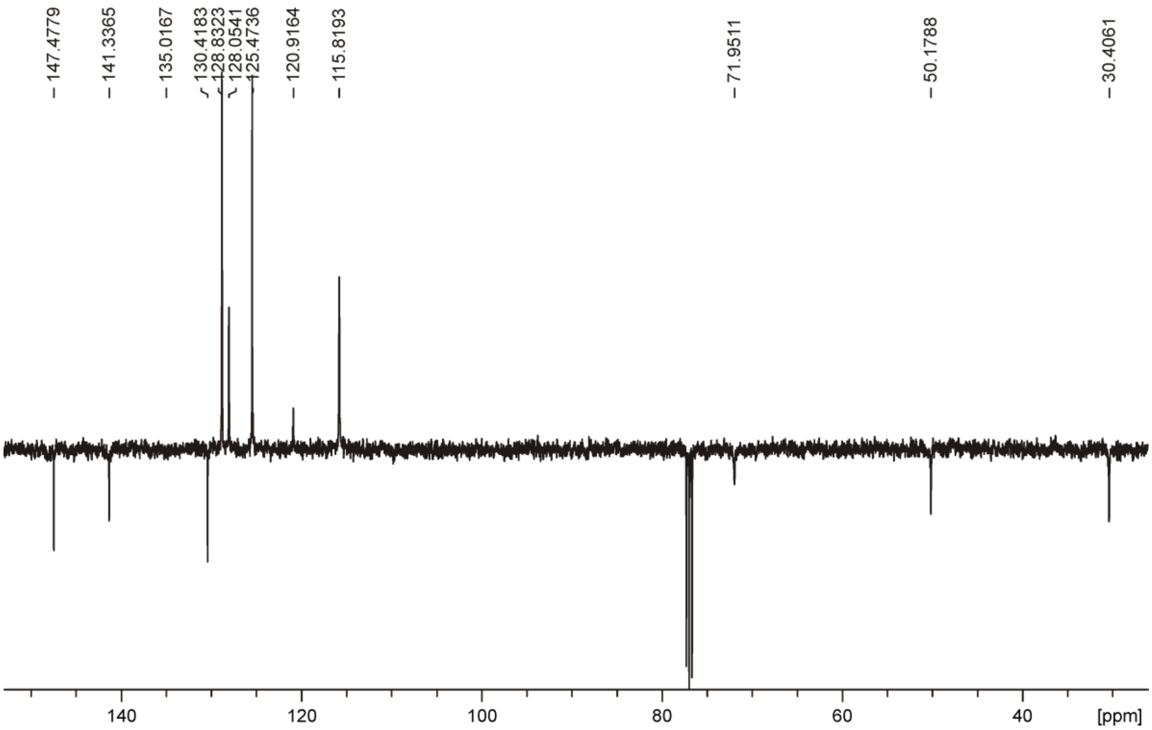

Figure S66. ^{13}C NMR spectrum (APT) of calixarene **45** (100 MHz, $CDCl_3$).

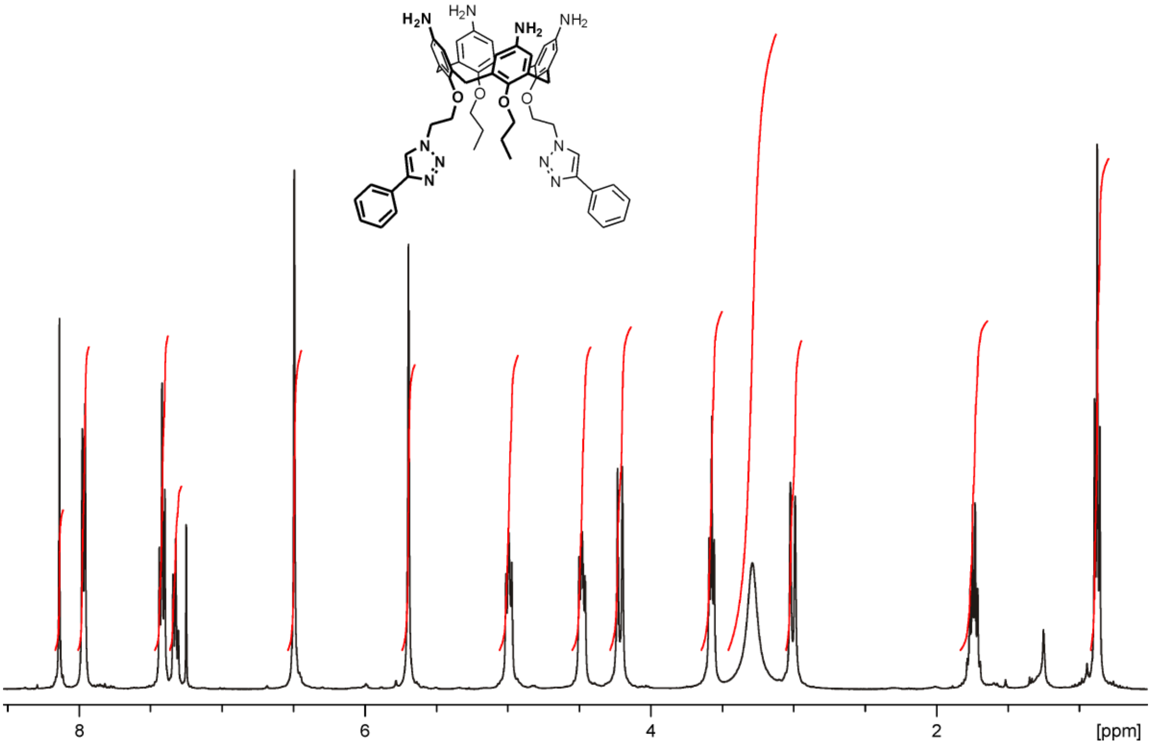

Figure S67. ^{1}H NMR spectrum of calixarene **46** (400 MHz, $CDCl_3$).

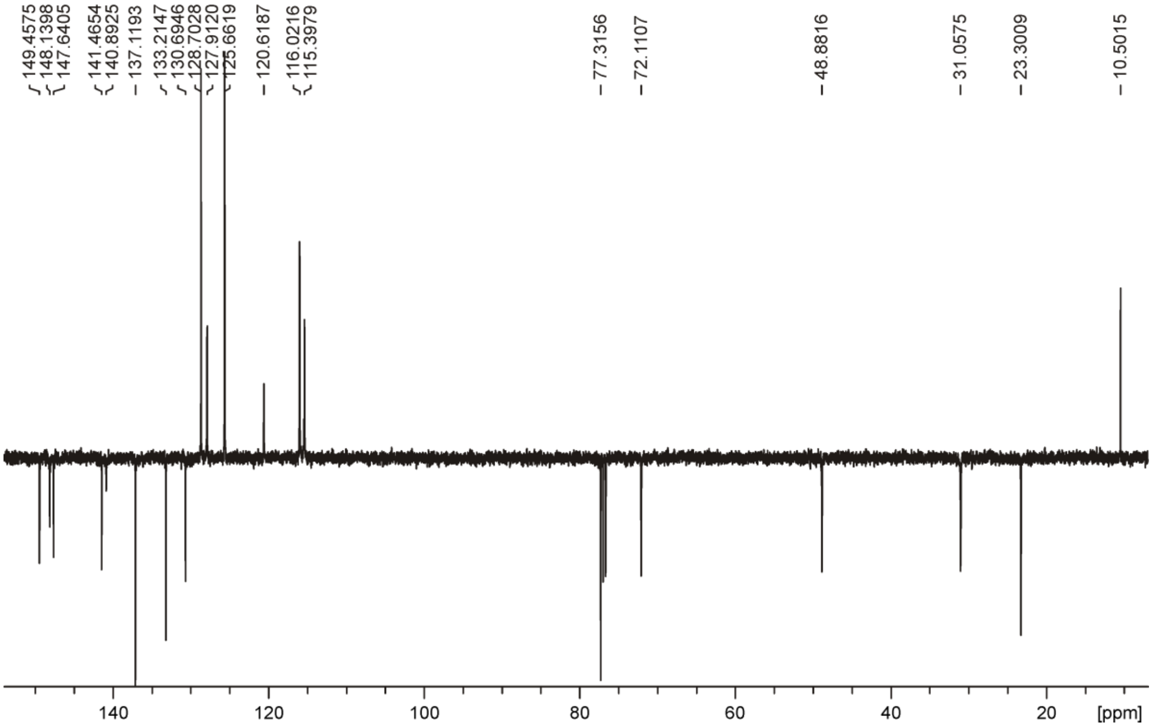

Figure S68. ^{13}C NMR spectrum (APT) of calixarene **46** (100 MHz, $CDCl_3$).

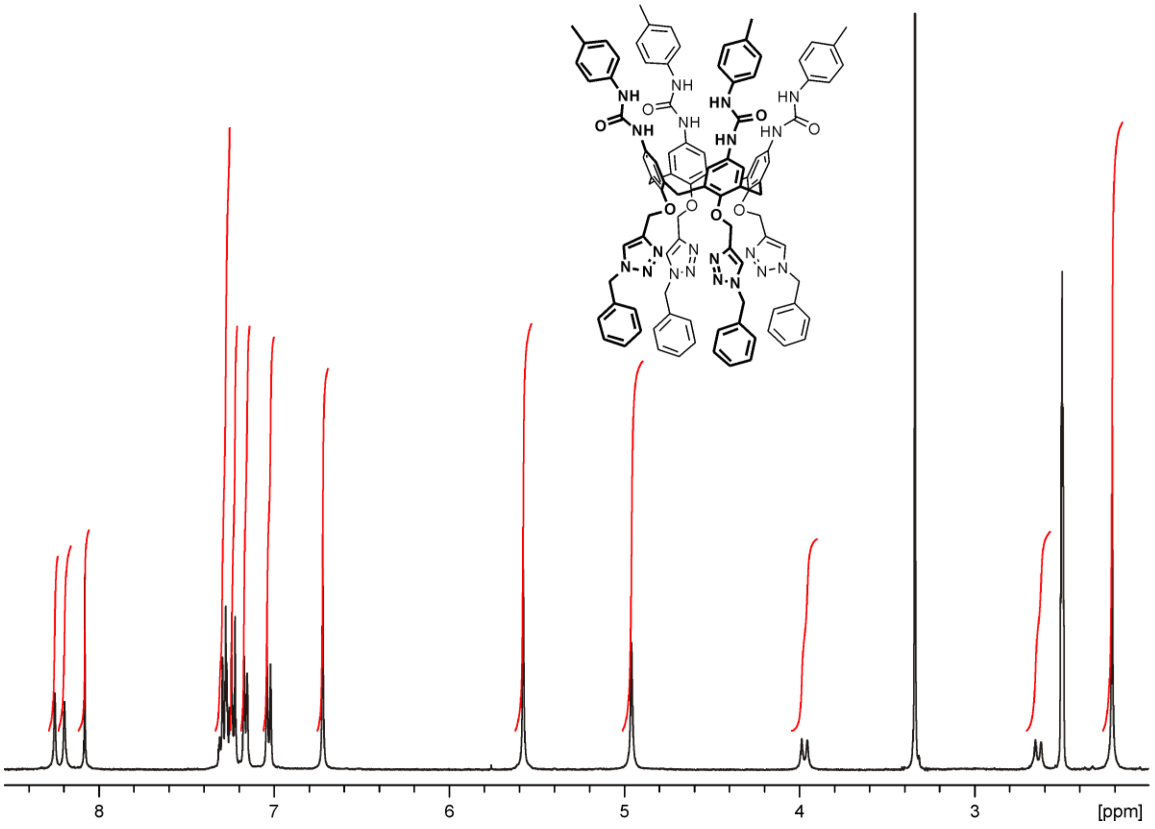

Figure S69. ^{1}H NMR spectrum of calixarene **47** (400 MHz, DMSO-d_6).

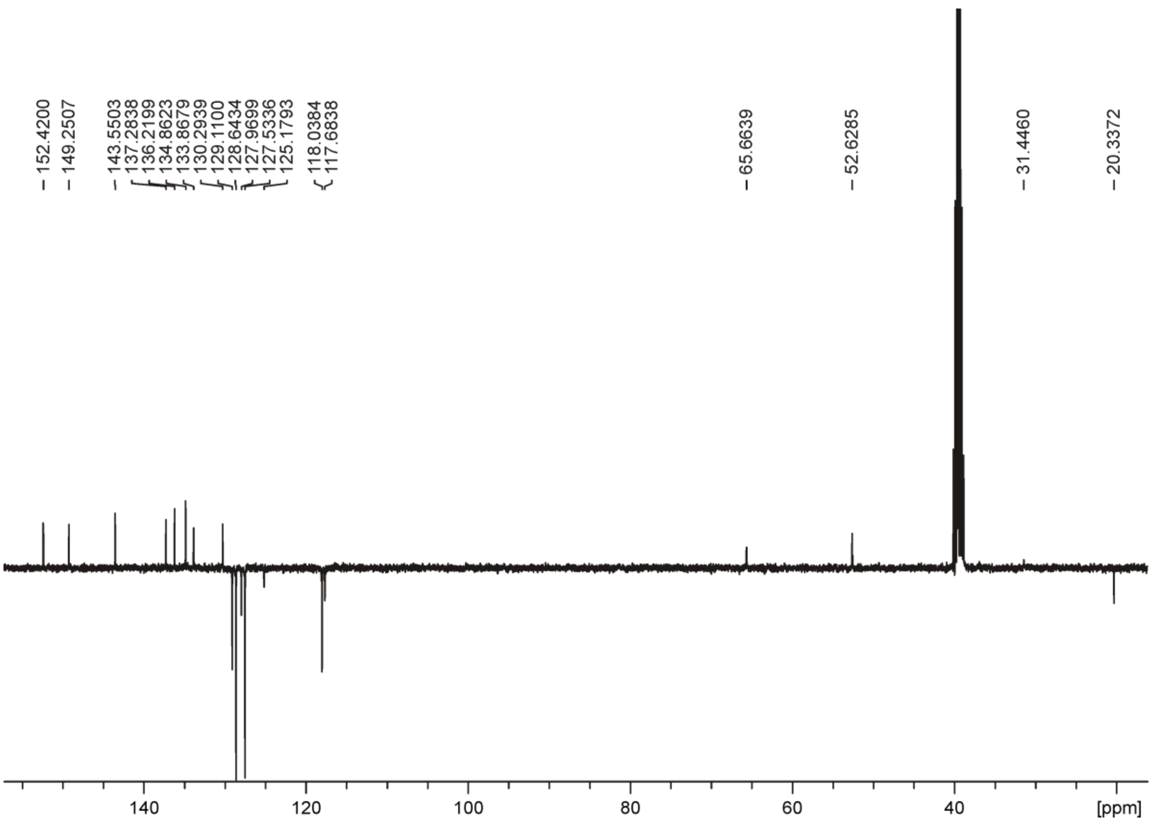

Figure S70. ^{13}C NMR spectrum (APT) of calixarene **47** (100 MHz, DMSO-d_6).

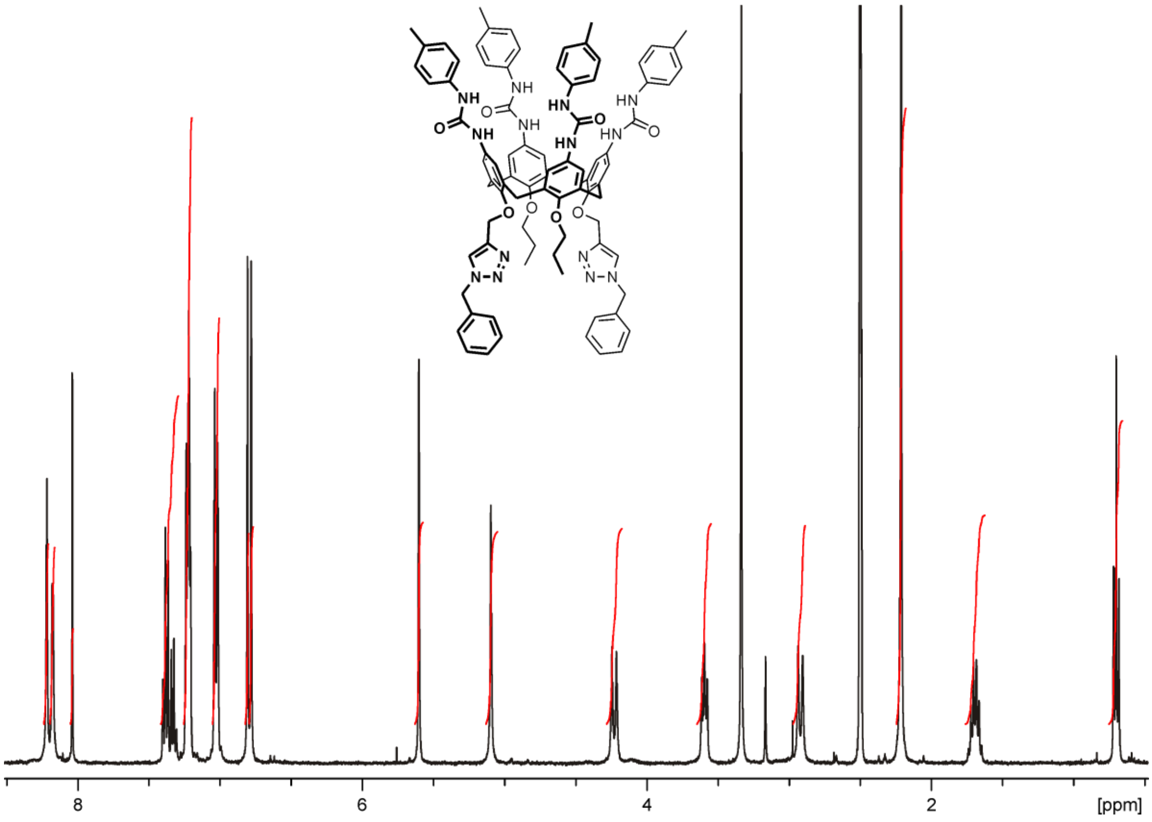

Figure S71. ^{1}H NMR spectrum of calixarene **48** (400 MHz, DMSO-d_6).

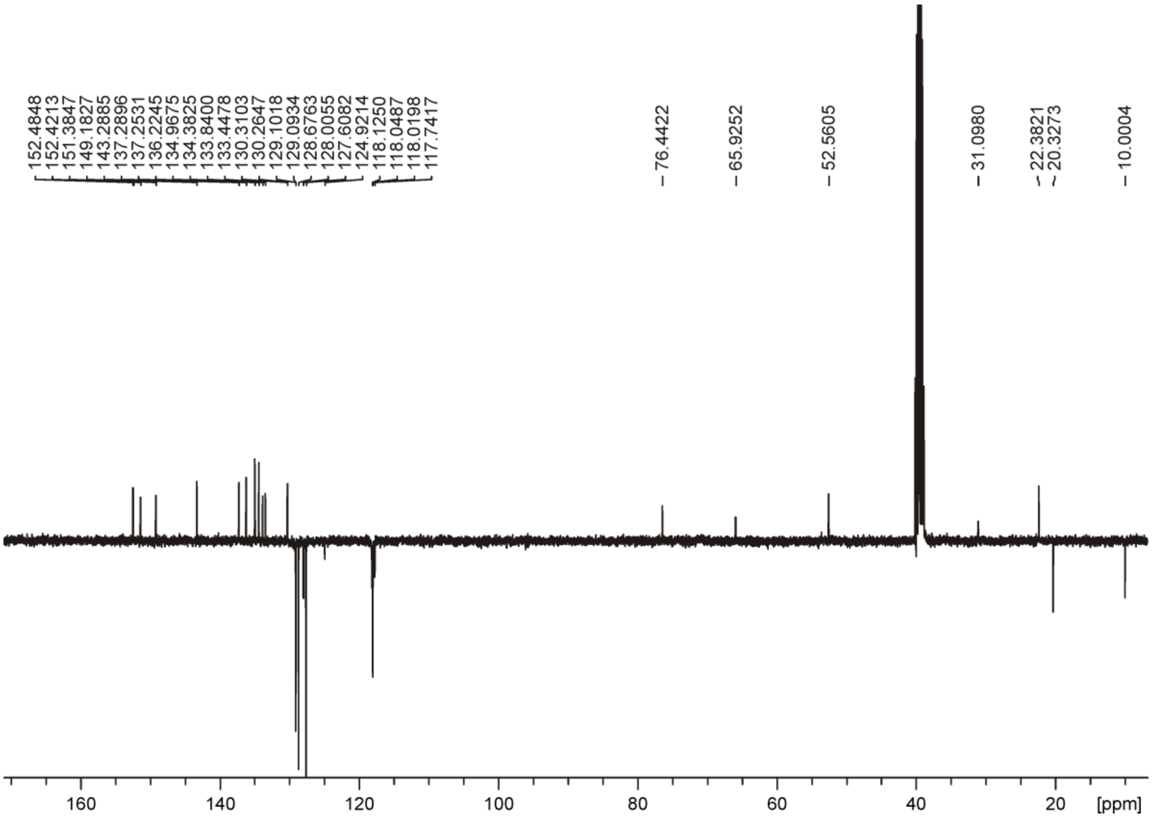

Figure S72. ^{13}C NMR spectrum (APT) of calixarene **48** (100 MHz, DMSO-d_6).

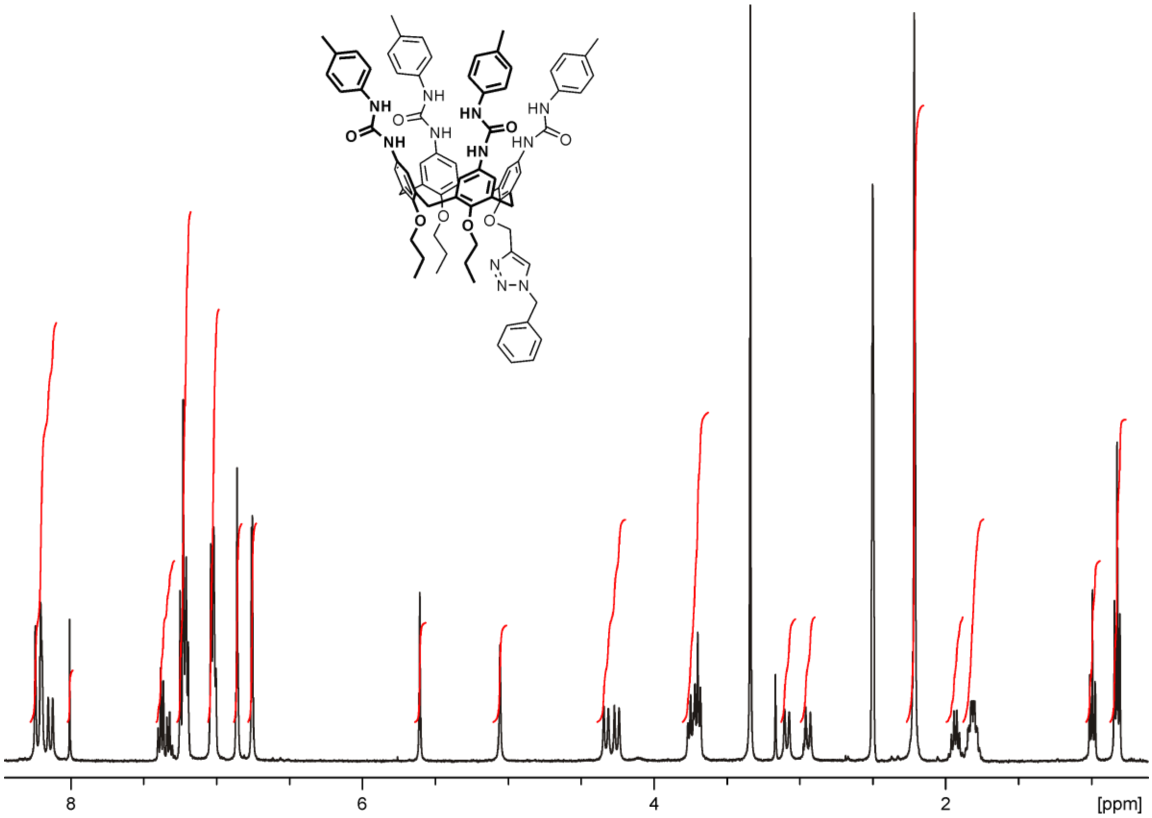

Figure S73. ^{1}H NMR spectrum of calixarene **49** (400 MHz, DMSO-d_6).

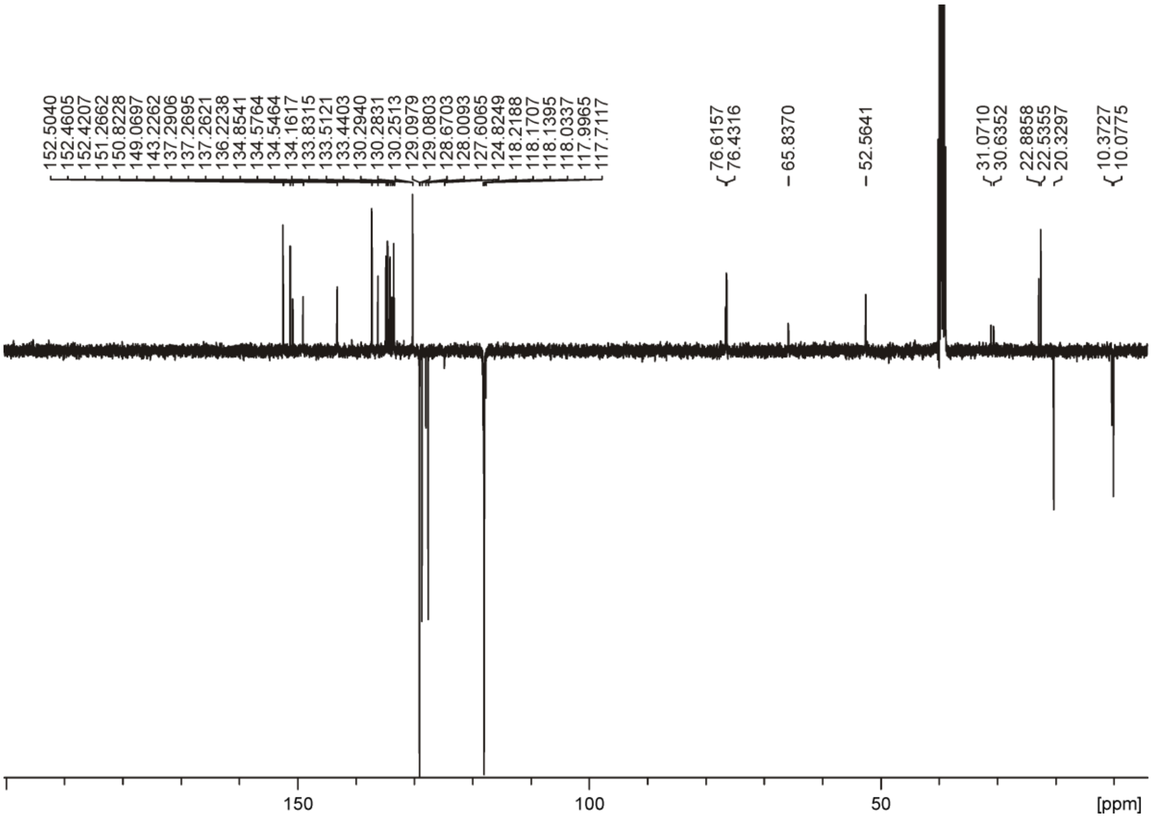

Figure S74. ^{13}C NMR spectrum (APT) of calixarene **49** (100 MHz, DMSO-d_6).

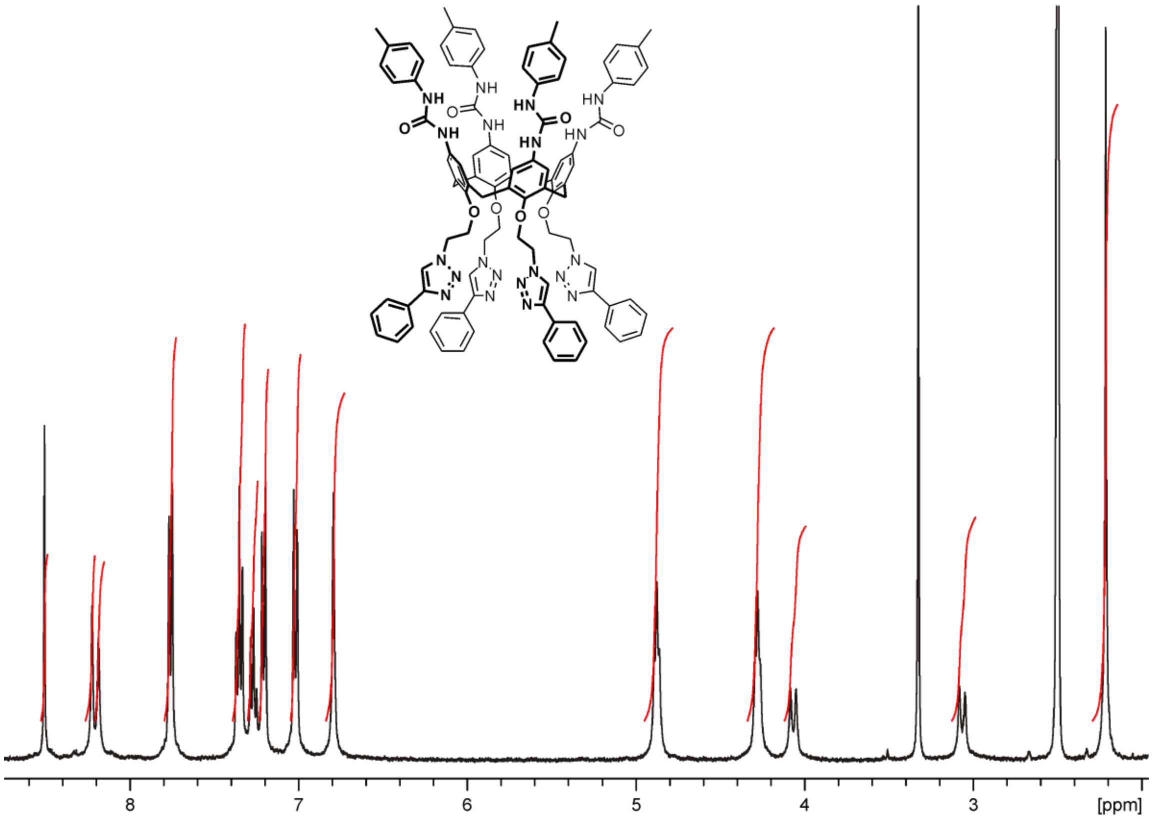

Figure S75. ^{1}H NMR spectrum of calixarene **50** (400 MHz, DMSO-d_6).

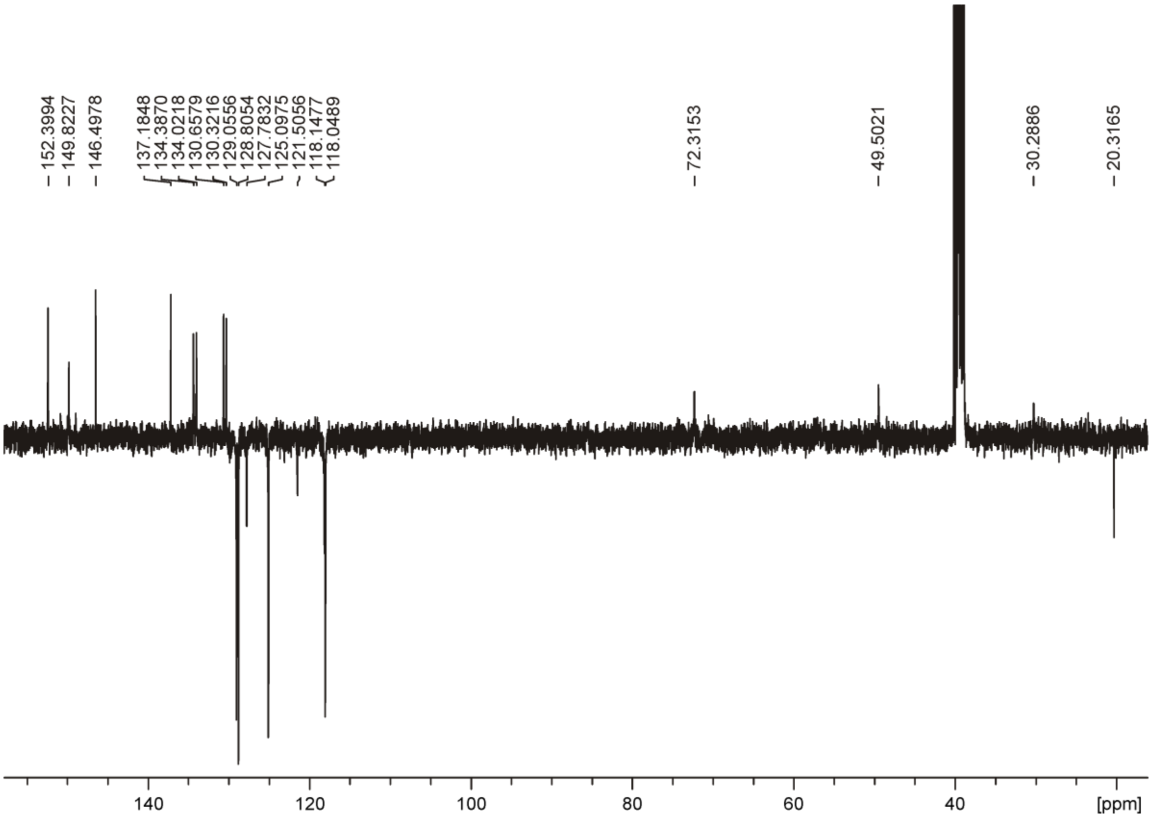

Figure S76. ^{13}C NMR spectrum (APT) of calixarene **50** (100 MHz, DMSO-d_6).

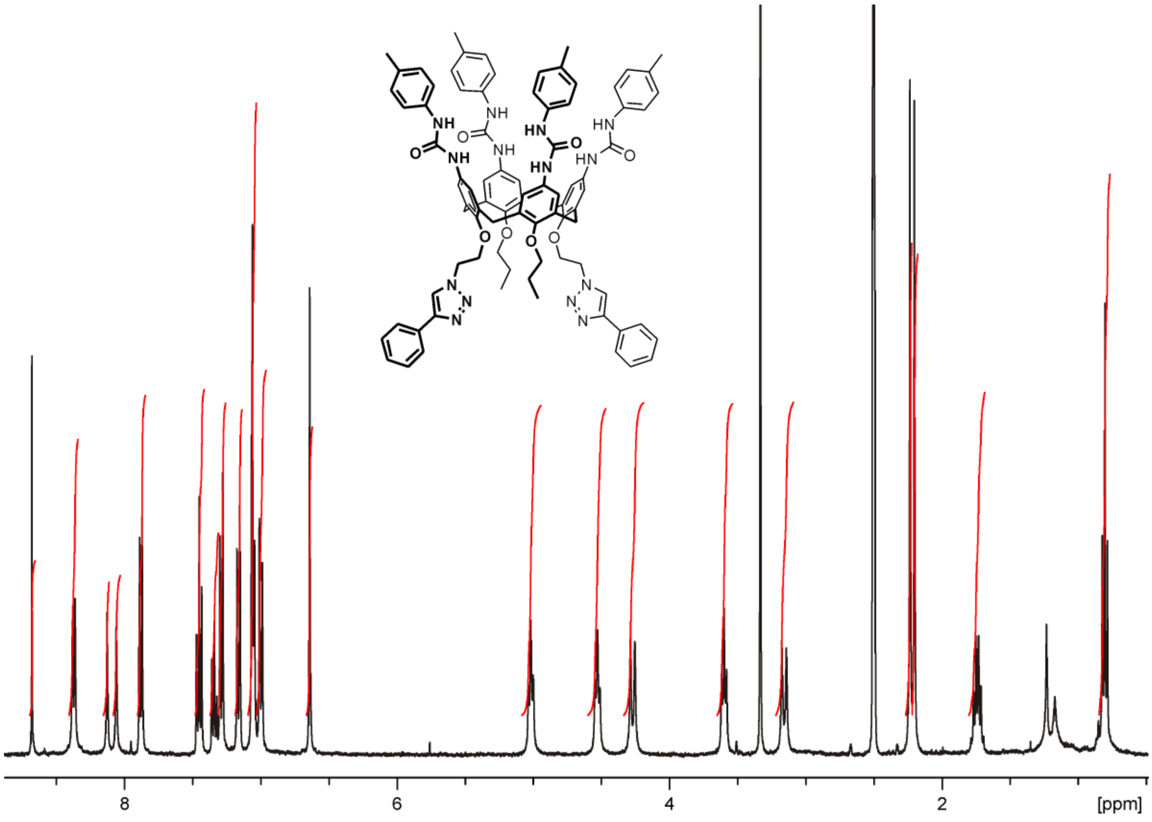

Figure S77. ^{1}H NMR spectrum of calixarene **51** (400 MHz, DMSO-d_6).

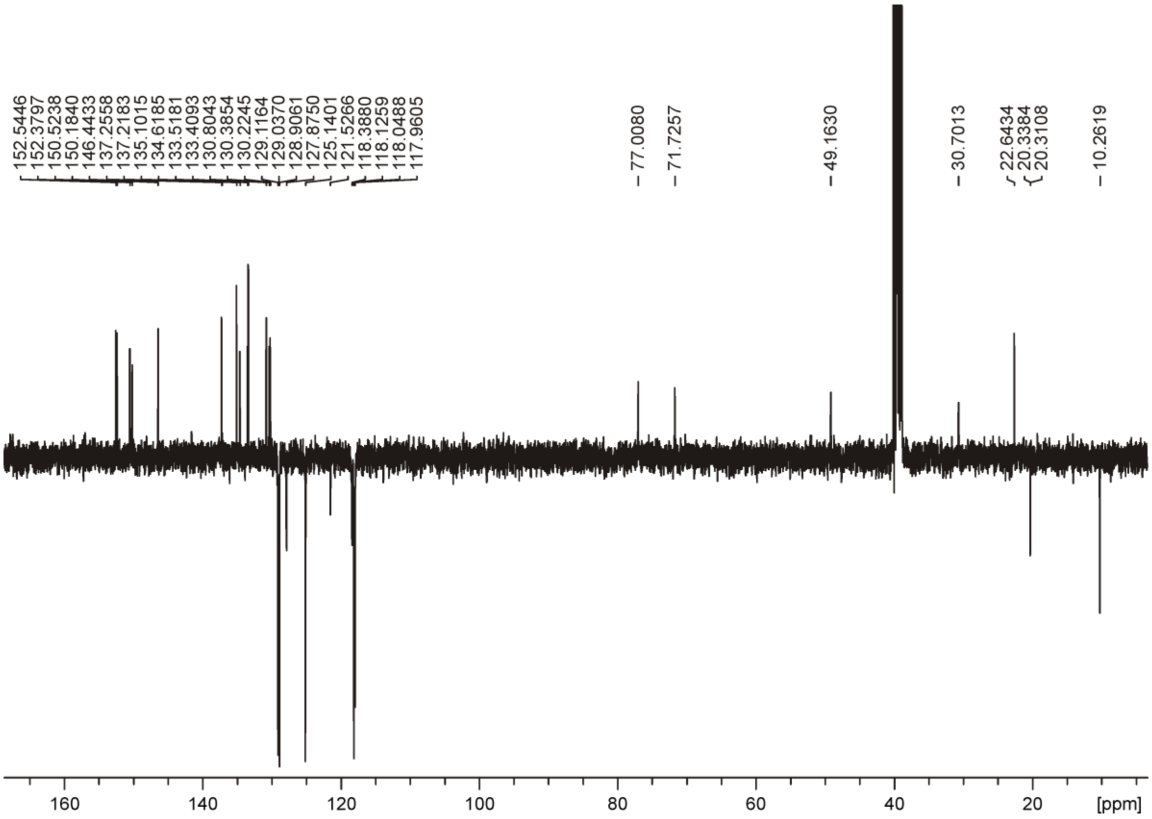

Figure S78. ^{13}C NMR spectrum (APT) of calixarene **51** (100 MHz, DMSO-d_6).

Details of X-ray diffraction measurements

Crystallographic data were collected on a Bruker D8 VENTURE diffractometer using graphite monochromatized Mo Kα radiation (λ = 0.71073 Å) using a ω-scan mode. Absorption correction based on measurements of equivalent reflections was applied.[S9] The structures were solved by direct methods (SHELXT 2018/2)[S10] and refined by full matrix least-squares on F^2 with anisotropic thermal parameters for all non-hydrogen atoms (SHELXL 2018/3)[S11] as implemented in the Olex2 package.[S12] In all the structures, hydrogen atoms were placed in calculated positions and refined using a riding model. In the structure of **15**, two NO_2 groups are disordered over two positions with equal occupancies. Two propoxy groups are also disordered over two positions with equal occupancies. There is also disorder in solvent dichloromethane molecule (occupancy ratio 0.6/0.4). In the structure of **16**, two propoxy groups are positionally disordered with refined occupancy ratios of 0.85–0.15 and 0.6/0.4, respectively. The *tert*-butyl group is disordered with occupancy ratio 0.65/0.35. Solvent methanol molecule is disordered over two positions with occupancy ratio 0.7/0.3. In the structure of **15**, disordered solvent molecules were not located and their contribution was suppressed by the SQUEEZE procedure.[S13] Crystallographic details are presented in Table S1.

Table S1. Details of the X-ray crystal data collection and structure refinement for compounds **15** and **16**.

Compound	**15**	**16**
Formula	$C_{52}H_{64}N_4O_{12}Si_2 \cdot CH_2Cl_2$	$C_{50}H_{63}N_3O_{10}Si_1 \cdot CH_3OH$
M_w	1078.17	926.16
Size (mm)	0.35 × 0.40 × 0.45	0.26 × 0.38 × 0.40
Crystal system	monoclinic	monoclinic
Space group	$P2_1/n$	$P2_1/c$
a (Å)	12.9613(4)	18.4272(10)
b (Å)	16.8169(5)	13.3723(7)
c (Å)	27.4288(7)	21.1077(12)
β (°)	99.0190(10)	104.341(2)
V (Å^3)	5904.7(3)	5039.2(5)
Z	4	4
ρ_{calc} (g·cm^{-3})	1.213	1.221
μ, mm^{-1}	0.210	0.108
F(000)	2280	1984
θ range (deg)	1.86 – 26.02	1.82 – 25.05
Index ranges	$-16 \le h \le 16$<br>$-20 \le k \le 20$<br>$-33 \le l \le 33$	$-21 \le h \le 21$<br>$-15 \le k \le 15$<br>$-25 \le l \le 25$
Reflections collected	93317	48394
Independent reflections	11536	8900
Completeness to θ (%)	99.3	99.9
Parameters	687	620
Goodness of fit on F^2	1.014	1.039
R_1 ($I > 2\sigma(I)$)	0.1027	0.0618
wR_2 (all data)	0.2949	0.1694
ρ_{max}/ρ_{min} (e/Å^3)	2.42 / –0.94	0.94 / –0.80

References

S1. Chetcuti, M. J.; Devoille, A. M. J.; Ben Othman, A.; Souane, R.; Thuéry, P.; Vicens, J. *Dalton Trans.*, **2009**, 2999–3008.

S2. Gullo, M. C.; Baldini, L.; Casnati, A.; Marchiò, L. *Cryst. Growth Des.*, **2020**, *20*, 3611–3616.

S3. Cecioni, S.; Lalor, R.; Blanchard, B.; Praly, J.-P.; Imberty, A.; Matthews, S. E.; Vidal, S. *Chem. – Eur. J.*, **2009**, *15*, 13232–13240.

S4. Chang, K.-C.; Chen, C.-Y.; Hsu, C.-Y.; Lee, L.-W.; Chung, W.-S. *Analyst*, **2022**, *147*, 5105–5112.

S5. Arnaud-Neu, F.; Browne, J. K.; Byrne, D.; Marrs, D. J.; McKervey, M. A.; O'Hagan, P.; Schwing-Weill, M. J.; Walker, A. *Chem. – Eur. J.*, **1999**, *5*, 175–186.

S6. Knoblauch, S.; Falana, O. M.; Nam, J.; Roundhill, D. M.; Hennig, H.; Zeckert, K. *Inorg. Chim. Acta*, **2000**, *300*, 328–332.

S7. Webber, P. R. A.; Cowley, A.; Drew, M. G. B.; Beer, P. D. *Chem. – Eur. J.*, **2003**, *9*, 2439–2446.

S8. Buttress, J. P.; Day, D. P.; Courtney, J. M.; Lawrence, E. J.; Hughes, D. L.; Blagg, R. J.; Crossley, A.; Matthews, S. E.; Redshaw, C.; Bulman Page, P. C.; Wildgoose, G. G. *Langmuir*, **2016**, *32*, 7806–7813.

S9. Sheldrick, G. M. *Acta Crystallogr. A*, **2008**, *64*, 112–122.

S10. Sheldrick, G. M. *Acta Crystallogr. A*, **2015**, *71*, 3–8.

S11. Sheldrick, G. M. *Acta Crystallogr. C*, **2015**, *71*, 3–8.

S12. Dolomanov, O. V.; Bourhis, L. J.; Gildea, R. J.; Howard, J. A. K.; Puschmann, H. *J Appl Crystallogr*, **2009**, *42*, 339–341.

S13. Spek, A. L. *Acta Crystallogr. C*, **2015**, *71*, 9–18.
